# Oral Second-Generation H1-Antihistamine Regimens for Allergic Rhinitis: Systematic Review and Network Meta-Analysis of Effects and Estimability

**DOI:** 10.3390/jcm15187169

**Published:** 2026-09-15

**Authors:** José David Maya Viejo, Fernando María Navarro i Ros, Eva Trillo-Calvo, Luis Richard Rodríguez

**Affiliations:** 1 Unidad de Gestión Clínica de Camas, 41900 Camas, Sevilla, Spain; 2 Centro de Salud Plaza Segovia, 46017 València, Valencia, Spain; fnavarroyros@hotmail.com; 3 Centro de Salud Campo de Belchite, 50130 Belchite, Zaragoza, Spain; etrilloc@salud.aragon.es; 4 Instituto de Investigación Sanitaria Aragón (IIS Aragón), Avenida San Juan Bosco 13, 50009 Zaragoza, Spain; 5 Centro de Salud Puerto de Santa María Sur, 11500 Puerto de Santa María, Cádiz, Spain; luisrichar28@gmail.com

**Keywords:** allergic rhinitis, seasonal allergic rhinitis, perennial allergic rhinitis, persistent allergic rhinitis, second-generation H1-antihistamines, network meta-analysis, comparative effectiveness research, patient-reported outcome measures, adverse events

## Abstract

**Background/Objectives:** Comparative effects of oral second-generation H1-antihistamines vary by rhinitis category, outcome, and treatment period. We assessed evidence structure, estimability, and differentiation among regimens. **Methods:** PubMed, Embase, Web of Science Core Collection, CENTRAL, WHO ICTRP, ClinicalTrials.gov, and the EU Clinical Trials Register were searched on 1–3 July 2026 for records through 30 June 2026. Eligible studies were natural-exposure randomized trials. Continuous outcomes were analyzed using covariance-aware generalized least squares, and binary harms were analyzed using Bayesian arm-level models. RoB 2, CINeMA, and ROB-MEN were applied in duplicate. Registration was retrospective. **Results:** Sixty-one reports generated 66 report–randomization entries from 64 cohorts (34,831 participants); 35 model-ready randomizations informed nine structures. In seasonal allergic rhinitis, six of seven TNSS estimates, two of three ocular estimates, and four RQLQ estimates favored active treatment. Any-adverse-event estimates were imprecise. Under the primary prior, cetirizine yielded a WDAE OR of 0.25 (95% CrI 0.08–0.66), but predictive classification was prior-sensitive. Of 34 continuous active–active comparisons, seven were directly informed and 27 indirect-only. All 147 estimable binary active–active contrasts were reported as secondary derivations: 141 CrIs included one and six excluded one, all indirect-only and not formally appraised with CINeMA. Six of 14 regimen-level nodes contributed only to tolerability structures, precluding balanced regimen-specific benefit–harm assessment. All 89 estimable comparisons within the defined CINeMA appraisal scope were rated very low; two non-estimable effects were not evaluable. **Conclusions:** Predominantly placebo-anchored and sparse evidence established neither a reliable regimen hierarchy nor therapeutic equivalence. Findings were hypothesis-generating, and somnolence remained an evidence gap. No external funding supported conduct; NEXUS PEOPLE, S.L. funded the APC. OSF registration was retrospective: doi:10.17605/OSF.IO/DPT5N.

## 1. Introduction

Allergic rhinitis is a common IgE-mediated inflammatory disorder of the nasal mucosa that often coexists with allergic conjunctivitis and asthma. Beyond nasal itching, sneezing, rhinorrhea, and obstruction, it may cause ocular symptoms and impair sleep, daily functioning, productivity, and health-related quality of life [1,2]. Intranasal corticosteroids generally provide the most effective control of persistent or prominent nasal symptoms, but oral second-generation H1-antihistamines remain widely used for histamine-mediated nasal and ocular symptoms because of their convenience and patient acceptability [3,4,5,6].

The clinically relevant question is therefore not whether oral H1-antihistamines are effective as a class, but whether the available comparative evidence supports selecting one drug–dose regimen over another for a defined rhinitis category, outcome, and treatment period. Agents differ in pharmacology, dosing schedules, sedative potential, interaction profiles, and the quantity and certainty of supporting evidence [6,7,8]; class efficacy therefore does not imply therapeutic interchangeability. Comparative effects are outcome-specific: symptom, quality-of-life, and harm outcomes are distinct constructs that differ in measurement properties and in susceptibility to ascertainment and reporting. A favorable estimate in one domain does not establish overall superiority; statistical non-nullity does not establish clinical importance, and an interval spanning the null does not establish equivalence. Somnolence warrants particular attention because central nervous system effects may impair daily functioning, occupational safety, and driving [7,8], making consistent ascertainment and reporting essential for a valid comparative assessment.

Previous network meta-analyses pooled more broadly and emphasized treatment rankings [9,10], whereas guideline-linked syntheses rated the evidence supporting many oral-treatment comparisons as low or very low certainty [6,10]. Rankings order treatments but do not indicate whether the evidence structure is connected or whether the underlying estimates are precise, credible, and estimable; they may also change after modest alterations in the evidence base or analytical assumptions without materially changing comparative estimates [11]. Because evidence geometry and information content vary by endpoint, a structure may be informative for one outcome but sparse, disconnected, saturated, or limited to direct comparisons for another. These properties must be mapped before comparative estimates or rankings are interpreted.

We therefore conducted an evidence-mapping systematic review and outcome-specific network meta-analysis of oral second-generation H1-antihistamine monotherapy for seasonal, perennial, and persistent allergic rhinitis, with independent randomizations as evidence units and treatment nodes defined by regimen. Total nasal symptom score (TNSS), total ocular symptom score (TOSS), Rhinoconjunctivitis Quality of Life Questionnaire (RQLQ) score, any adverse event, and withdrawal due to adverse events were analyzed in separate rhinitis-category–outcome structures. The review was retrospectively registered; this limitation and the associated methodological audit trail are described in the Methods. We aimed to characterize evidence structure and estimability, quantify outcome-specific comparative effects and uncertainty, and determine whether the evidence supported reliable differentiation among active regimens.

## 2. Materials and Methods

### 2.1. Protocol, Retrospective Registration, and Reporting

This review was not prospectively registered. Study selection, quantitative extraction, evidence appraisal, the principal analyses, implementation-independent computational replication, and figure production preceded registration. On 4 August 2026, a retrospectively reconstructed protocol and statistical analysis plan, verbatim search strategies, a chronology of review conduct, a retrospective-registration statement, and a SHA-256 file manifest were registered in OSF Registries (doi:10.17605/OSF.IO/DPT5N) [12]. This immutable, time-stamped record documents the methods used but does not confer the safeguards of prospective registration. The originally submitted file set was version 1.0 and used the frozen primary analytical release v56.0; the current submission and supporting reproducibility package are version 1.1.

Reporting followed PRISMA 2020, its Explanation and Elaboration document, PRISMA-NMA, and PRISMA-S; the PRISMA Checklist is provided in the Supplementary Materials [13,14,15,16]. Network meta-analysis methods were informed by the Cochrane Handbook [17]; the appraisal frameworks are described in Section 2.8. Evidence units were defined consistently as bibliographic reports, report–randomization entries, distinct non-overlapping randomized cohorts, and model-ready independent randomizations. The final corpus comprised 61 retained reports, 66 report–randomization entries, 64 distinct non-overlapping cohorts, and 35 model-ready independent randomizations. Three duplicate bibliographic representations were recorded as duplicates rather than substantive exclusions, and missing N metadata for the Van Adelsberg et al. (2003) trial [18] were taken from the source patient-disposition information. These source and unit definitions did not alter any comparative-effect or standard-error input, fitted model, or treatment-effect estimate.

The final analytical specification used the randomized-treatment risk period for the Ratner et al. (2017) [19] WDAE result, treated bepotastine 10 mg twice daily and controlled-release bepotastine 20 mg once daily as separate regimen nodes, applied the documented 0.75 transformation to the Okubo et al. (2019) [20] T4NSS scale, used source-verified randomization identifiers and the 17,887 frozen arm-level denominator, and applied the final conservative CINeMA incoherence judgments. Version 1.1 also reports 147 secondary binary active–active contrasts derived by exact within-draw subtraction from joint posterior draws retained in the implementation-independent CmdStanR replication; no model was refitted, and no additional MCMC sampling was performed. These specifications were applied irrespective of effect direction. Tables S1 and S34 summarize protocol status, final analytical specifications, and the relationship between submitted version 1.0 (analytical release v56.0) and current version 1.1.

### 2.2. Information Sources and Search Strategy

PubMed and Embase were searched on 1 July 2026; Web of Science Core Collection and the Cochrane Central Register of Controlled Trials on 2 July; and the World Health Organization International Clinical Trials Registry Platform, ClinicalTrials.gov, and the European Union Clinical Trials Register on 3 July 2026. Searches covered records available from inception through 30 June 2026 and used no language restrictions. Strategies combined controlled vocabulary and free-text terms for allergic rhinitis or rhinoconjunctivitis, oral H1-antihistamines and individual agents, and randomized or controlled trials. The same conceptual framework was applied across sources, with indexing terms, field codes, Boolean operators, and proximity syntax adapted to each platform. Complete strategies, execution dates, and aggregate counts are reported in Table S2 [16].

Reference lists of eligible reports and relevant systematic reviews were examined to verify report linkage, secondary-publication relationships, and trial-family completeness; no additional reports entered the review outside the database/register search flow [13,17]. Deduplication and screening were managed in Rayyan.ai. Records were classified as duplicates when they shared an identical DOI or, in the absence of a reliable DOI match, when similarity in the title or author list, corroborated by equivalent or closely similar available record content, indicated that they represented the same bibliographic report. A total of 6261 duplicate records were removed. Original database exports and the retained record-level decision log were not publicly deposited; the duplicate-cluster log and complete reviewer-specific decision timestamps were not retained.

### 2.3. Eligibility Criteria

We included randomized parallel-group trials involving oral second-generation H1-antihistamine regimens in participants with seasonal (SAR), perennial (PAR), or persistent allergic rhinitis (PER) under natural allergen exposure [21]. The nine principal quantitative structures required an eligible oral second-generation H1-antihistamine monotherapy arm and a valid placebo or other eligible oral H1-antihistamine comparator, together with an outcome, scale, treatment period, risk period, and analysis population compatible with the target estimand. Pediatric-only populations; non-randomized or uncontrolled studies; allergen-chamber or experimental-provocation studies; topical-only interventions; and inseparable populations, interventions, or outcomes were excluded from the relevant quantitative synthesis (Table S3). Review-corpus retention was deliberately distinguished from principal-model eligibility: randomized evidence relevant to the review could remain in the evidence map or a contextual/sensitivity layer when no contrast met all principal-synthesis criteria, provided that its analytical disposition was explicitly recorded.

Eligibility and analytical contribution were therefore assessed hierarchically at report, independent-randomization, arm, and outcome-specific contrast levels. Eligible antihistamine and placebo/comparator arms from trials that also included an ineligible pharmacological class could be retained when randomization among the retained arms remained valid; ineligible arms were omitted from the corresponding quantitative structure, while the wider trial context informed transitivity and partial-arm analyses [17]. Retention of a report or randomized cohort in the review corpus did not imply contribution to a principal network meta-analysis.

Regimen eligibility followed a controlled dictionary defining molecule, dose, frequency, route, and node-defining formulation (Table S4); licensing status at the protocol reference date and operational classification as a second-generation H1-antihistamine are documented in the registered protocol. Eligibility applied to the specified jurisdictions and dates and did not imply contemporaneous authorization in all countries [21].

SAR, PAR, and PER were analyzed separately because allergen-exposure pattern, symptom duration, baseline burden, treatment period, placebo response, and clinical context could modify relative effects. They were treated as reported clinical categories rather than mutually exclusive biological phenotypes: SAR and PAR primarily reflect exposure patterns, whereas PER reflects symptom duration under the ARIA classification [2]. Potential overlap informed applicability and transitivity assessments.

### 2.4. Study Units, Selection, and Data Extraction

The unit of evidence for quantitative synthesis was the independent randomization, not the publication. Reports from the same trial were linked within trial families to prevent double counting, whereas reports containing multiple independent randomizations contributed separate report–randomization entries. Two companion entries in the full-corpus crosswalk mapped to randomizations already represented by primary reports and therefore contributed no additional study or participant denominators. Participant counting was performed at the level of non-overlapping randomized cohorts, with each cohort counted once [17].

Two reviewers independently performed title-and-abstract screening and report-level eligibility assessment in Rayyan.ai. Disagreements or differences in the application of eligibility criteria were resolved by a supervisor. The final inclusion/exclusion ledger was checked against the retained Rayyan export and source reports. Quantitative data were independently extracted in duplicate using structured, version-controlled forms, with disagreements resolved by discussion or supervisor adjudication. Each report-level exclusion decision received one mutually exclusive final classification. Three report-level records were duplicate bibliographic representations of reports already retained and were classified as duplicates rather than substantive exclusions; final decisions and supporting notes are documented in Table S12B [13,17].

A predefined source hierarchy prioritized valid adjusted between-group contrasts from each trial’s prespecified randomized analysis, followed by compatible arm-level change scores, derivable change scores, final-value estimates, and independently verified numerical reconstructions (Table S5). Abstract- or trial-register-only results were retained only when no more complete compatible source existed and were flagged for sensitivity analysis and evidence appraisal [17].

Missing values were not imputed as zero, and participants with missing or unreported binary outcomes were not assumed event-free. Randomized, treated, efficacy-analysis, and safety-analysis denominators were recorded separately; safety denominators were not substituted for efficacy denominators or used to reconstruct continuous-outcome variances. Percentages were converted to integer counts only when the percentage, denominator, and rounding convention yielded a unique mathematically possible value; otherwise, the result was classified as non-model-ready. Source locations, derivations, assumptions, adjudication decisions, and verification status are documented in the audit files [17].

### 2.5. Outcomes, Estimands, and Treatment Periods

Continuous outcomes were the canonical four-item total nasal symptom score (TNSS; 0–12), three-item total ocular symptom score (TOSS; 0–9) [21], and total Rhinoconjunctivitis Quality of Life Questionnaire score (RQLQ; 0–6) [22,23]; lower scores indicated greater benefit. Binary outcomes were the proportions of participants experiencing at least one all-causality adverse event (Any AE) and withdrawing because of an adverse event (WDAE) during randomized treatment [24,25].

The principal framework comprised nine category–outcome structures: TNSS–SAR, TNSS–PAR, TOSS–SAR, RQLQ–SAR, RQLQ–PER, Any AE–SAR, Any AE–PAR, WDAE–SAR, and WDAE–PAR. Other combinations were considered only in explicitly labeled sensitivity or exploratory analyses when compatible data were available.

Eligible results had to match the target estimand in components, direction, respondent, temporal aggregation, recall method, and summary statistic; similar labels did not establish construct equivalence. Period averages, visit endpoints, and reflective and instantaneous assessments were distinguished where recoverable [17,21]. Exact linear scale transformations were permitted only when component structure and weighting were identical and were applied consistently to means, dispersion measures, and interval limits. The source-labeled three-item non-nasal symptom score in Kuna et al. (2009) [26] was accepted as construct-equivalent to the canonical ocular-only TOSS only after source verification showed that it comprised exactly ocular tearing, redness, and itching, each scored 0–3 (total range 0–9).

The continuous estimand was the between-group mean difference attributable to randomized assignment at the target period, oriented so that negative values favored active treatment. The binary estimand was the odds ratio for participants experiencing the outcome during the eligible risk period, with values below 1 favoring active treatment. Event totals were not treated as participant-risk data. All-causality adverse events were preferred to treatment-related events, and periods combining randomized treatment with post-treatment follow-up were excluded [17,24,25].

Target efficacy periods were days 1–14 for SAR and days 1–28 for PAR and PER. Treatment-period averages were preferred; otherwise, the eligible adjusted endpoint nearest the target day was selected. Safety periods matched the corresponding 14- and 28-day randomized-treatment windows; longer randomized exposure was retained only in labeled extended-period analyses. Exact windows and deterministic selection rules are reported in Table S6 [21].

For Okubo et al. (2019) [20], the four-item T4NSS used the same four nasal components and equal weighting as the canonical four-item TNSS but scored each item 0–4 (total range 0–16). Source-scale active-placebo contrasts were −1.084 (SE 0.208) for rupatadine 10 mg and −1.509 (SE 0.215) for rupatadine 20 mg. Effects and standard errors were transformed exactly by multiplying by 0.75, yielding −0.813 (SE 0.156) and −1.132 (SE 0.161) on the 0–12 metric before entry into the primary TNSS-SAR model. No untransformed 0–16 effect entered the synthesis. This linear conversion harmonizes the numerical range, not necessarily the semantic anchors of the 0–4 and 0–3 item categories; that limitation was considered in interpretation.

Somnolence-related outcomes were not synthesized because no sufficiently complete, consistently defined, source-verified, and connected dataset supported the intended estimand. Terminology, ascertainment, denominators, risk periods, and reasons for non-model-readiness for somnolence, sedation, and drowsiness are mapped in Table S7; this was treated as an evidence gap, not evidence of no difference.

The 0.5-point RQLQ change was used only as a contextual benchmark derived from within-patient change, not as a validated between-group minimal important difference or equivalence margin [27,28]. No validated between-group threshold was assumed for harmonized TNSS or TOSS.

### 2.6. Network Structure and Transitivity

Each category–outcome structure was evaluated separately, and network meta-analysis was restricted to connected components. Structures containing only one direct comparison or no independently informed indirect pathway were described as direct evidence structures; a consistency model with zero residual degrees of freedom was described as saturated. Residual degrees of freedom under the consistency model were distinguished from the additional degrees of freedom available to the design-by-treatment interaction model, so heterogeneity could be estimable when global incoherence was not. The term network estimate was reserved for effects supported by a connected comparative structure [11,17,29].

Geometry was characterized by randomized units, arms, regimen-level nodes, direct comparisons, multi-arm designs, connected components, graph cycles, residual degrees of freedom, and evidence distribution (Table S8A,B). Active–active comparisons were directly informed when at least one randomized design contained both regimens and were indirect-only otherwise. Cycle dimensions were partitioned into those induced within multi-arm randomizations and any additional dimensions formed across distinct randomized designs.

Transitivity was assessed before interpreting indirect estimates by comparing documented candidate effect modifiers, including rhinitis category and allergen exposure, baseline burden and age, treatment period, outcome definition and timing, placebo response, cointerventions, trial period, and result-specific risk of bias (Table S9). Pharmacological class membership alone was insufficient to establish exchangeability [11,17,29].

Independent-randomization-level contribution matrices were derived by random-walk evidence-flow decomposition and aggregated by target comparison and randomized unit; each target-comparison column had to sum to 100% within numerical tolerance. Shortest-path reconstruction served only as independent arithmetic validation [17,30].

### 2.7. Statistical Analysis

Treatment effects were parameterized relative to placebo when placebo belonged to the connected component; other pairwise effects were derived from fitted basic parameters within the generalized linear model [29]. The source-primary implementation retained marginal placebo-referenced posterior summaries but not joint posterior draws. However, the archived implementation-independent CmdStanR replication of the four source-primary binary scenarios retained the corresponding native chain files and joint posterior draws. For every estimable pair of active regimens A and B, a secondary contrast was derived at each of 24,000 retained posterior draws as d_A_^(s)^ − d_B_^(s)^ and exponentiated to obtain the active–active odds ratio. No model was refitted, and no additional MCMC sampling was performed. Before deriving these contrasts, all 36 estimable placebo-referenced effects were reproduced within the prespecified numerical tolerances for posterior medians and 95% credible-interval limits. Complete results are reported in Table S31 and Supplementary Data S1 (CSV file: Supplementary_Data_S1_Binary_Active_Active_Effects_v1.1.csv). These secondary contrasts were not multiplicity-adjusted or formally appraised with CINeMA.

Continuous outcomes used contrast-level random-effects generalized least-squares models, with between-study heterogeneity estimated by restricted maximum likelihood where identifiable. Multi-arm sampling covariance was retained: shared-arm covariance was derived from the relevant arm-level sampling variance, whereas the conventional one-half correlation applied only to the multi-arm random-effects component. Results were excluded when valid uncertainty or required covariance could not be recovered [17,30].

Mean differences were reported with 95% confidence intervals, generalized fixed-effect Cochran Q, its residual degrees of freedom and *p*-value, descriptive I^2^ [31], REML τ with profile-likelihood confidence intervals, and prediction intervals where heterogeneity was identifiable. Numerical diagnostics for all five continuous structures are reported in Table S33. Frequentist prediction intervals were plug-in intervals conditional on the REML estimate of τ and did not propagate uncertainty in its estimation, potentially understating predictive uncertainty when heterogeneity was weakly estimated. Bayesian Gaussian sensitivity models integrated over τ under half-normal priors with scales 0.5 and 1.0, where applicable.

Binary harms were analyzed using arm-level Bayesian binomial-logit random-effects models [29], preserving multi-arm randomization and avoiding normal contrast-level approximations in sparse data. Relative effects and study-specific baselines received weakly informative normal priors with standard deviations of 1.5 and 2.5 on the log-odds scale; heterogeneity received a half-normal prior with scale 0.5 in primary analyses and 1.0 in sensitivity analyses. Prior-predictive checks did not identify problematic concentrations of prior mass over implausible risks or relative effects.

No continuity correction was applied. Studies with zero events in every randomized arm, or events in every participant in every arm, were retained descriptively but contributed no relative-effect information; effects supported only by such evidence were classified as non-estimable [17,25,29]. Direct binary contrasts reported separately from the network models used unadjusted odds ratios and two-sided 95% conditional exact confidence intervals obtained by inversion of Fisher’s noncentral hypergeometric distribution.

Binary effects were reported as odds ratios with 95% credible intervals and, where heterogeneity was estimable, prediction intervals. Absolute risks were derived from each posterior draw at standardized baseline risks of 1%, 5%, 10%, and 20%, thereby propagating posterior uncertainty through the transformation [29]. Sensitivity to the heterogeneity prior was evaluated using posterior medians, credible intervals, and prediction intervals; interval classification relative to the null was considered only as a secondary descriptor.

Global incoherence was assessed with the design-by-treatment interaction model where estimable, and local direct–indirect separation only where identifiable [32,33]. Non-estimability informed CINeMA judgments. Ranking probabilities were not the principal basis for interpretation, and no hierarchy across outcomes was constructed [11].

**Interpretive rules.** (i) Parameters or contrasts not identified by the likelihood were reported as non-estimable and were not assigned prior-dominated estimates; non-estimability did not imply absence of effect, homogeneity, or consistency. Failure to detect incoherence likewise did not establish consistency. (ii) A REML heterogeneity estimate at the boundary of zero was considered not informatively estimated rather than evidence of homogeneity; no plug-in prediction interval was reported in that setting. (iii) Analyses were estimation-focused, and no family-wise multiplicity adjustment was applied; intervals excluding the null were not considered independently confirmatory, whereas intervals including the null did not establish equivalence. (iv) Project-specific thresholds and the 0.5-point RQLQ value were used only as contextual benchmarks, not as equivalence margins; estimates below a benchmark in absolute magnitude did not establish clinical unimportance.

### 2.8. Risk of Bias, Confidence, and Missing Evidence

RoB 2, CINeMA, and ROB-MEN appraisals were conducted independently in duplicate, with disagreements resolved by consensus or third-reviewer adjudication. RoB 2 targeted the effect of assignment to intervention for each eligible trial–result pair [34].

CINeMA assessed each comparison–outcome estimate across within-study bias, reporting bias, indirectness, imprecision, heterogeneity, and incoherence [35]. It covered all pairwise comparisons in the five continuous structures and placebo-referenced comparisons in the four binary structures; binary active–active comparisons were reported as not assessed rather than assigned a rating by inference. Judgments reflected the evidence contributing to each estimate rather than the structure as a whole. For estimable effects, domain judgments used the three CINeMA concern levels: no concerns, some concerns, or major concerns; not evaluable was reserved for non-estimable effects. When neither robust local direct–indirect separation nor a final source-verified global test was available, the incoherence domain was conservatively rated major concerns because the absence of a test could not establish consistency. GRADE-NMA guidance informed imprecision and incoherence but did not constitute a separate assessment or modify CINeMA [36,37,38,39,40]. Outcome-specific thresholds for imprecision and heterogeneity were project-specific, were applied under the interpretive rules in Section 2.7, and were not treated as validated equivalence margins.

ROB-MEN assessed bias due to missing evidence [41]. Pairwise judgments on unavailable results, potentially unpublished studies, and the likely direction of missingness were propagated through direct-evidence contribution matrices and interpreted alongside small-study effects and unobserved comparisons. A 15% contribution from evidence with suspected bias served as a project-specific operational indicator, with sensitivity analyses at 10%, 20%, and 25%; none was an official ROB-MEN threshold. In sparse structures, inability to assess small-study effects was recorded as non-assessability rather than absence of bias.

RoB 2 counted trial–result pairs, whereas CINeMA and ROB-MEN counted comparison-level estimates; these appraisal denominators therefore need not correspond arithmetically to participation counts in the modeled structures. Appraisal counts are reported in Section 3.8; participation counts are reported in Section 3.2. RoB 2 provenance is documented in Table S1 and result-specific assessments are reported in Tables S21A and S30.

### 2.9. Sensitivity and Exploratory Analyses

Table S10 reports all sensitivity and exploratory analyses, their rationale, classification, and affected structures. Pre-final-model denotes a decision documented in the frozen internal specification before fitting or inspecting the corresponding final output, not prospective public registration. Pre-final-model robustness analyses examined source completeness, scale compatibility, alternative treatment periods and longer exposure, event definitions and denominators, partial-arm retention, fixed versus random effects, higher-risk-of-bias exclusions, and influential trial families. After source verification, the Kuna et al. ocular NNSS [26] met the prespecified three-item ocular-only 0–9 construct; therefore, no separate construct-exclusion sensitivity was retained as an independent TOSS-SAR analysis. Four individual exploratory evidence-expansion analyses and one combined TNSS-PAR stress test were retained, together with ROB-MEN policy sensitivities.

Analyses introduced after primary-source verification or inspection of the final evidence structure were labeled post hoc. Those expanding the evidence base through assumptions absent from the primary models—including mixed-age eligibility, alternative scale-rater assumptions, per-protocol populations, or assumed covariance where source covariance was unrecoverable—were additionally classified as exploratory evidence-expansion analyses rather than conventional robustness analyses. Leave-one-study-out analyses were undertaken only when the resulting structure and model remained identifiable.

Robustness was evaluated based on changes in effect magnitude, interval width, predictive uncertainty, heterogeneity, estimability, geometry, node composition, and clinical interpretation rather than nominal statistical significance alone.

### 2.10. Computation and Reproducibility

Analyses were conducted in R 4.6.1; Bayesian models were fitted in CmdStan 2.39.0 through CmdStanR 0.9.0 [42,43,44]. Models used four chains and non-centered parameterizations where appropriate; scenario-specific warm-up and post-warm-up schedules are reported in Table S11. Frozen sampling criteria required rank-normalized split $\bar{R}\;$ ≤ 1.01, bulk and tail effective sample sizes ≥ 1000, E-BFMI ≥ 0.30 in every chain, no divergent transitions or maximum-treedepth hits, and successful return codes; trace plots and posterior predictive checks were also reviewed [43,45]. Simulation-based calibration across 30 simulated datasets assessed predefined rank-uniformity and coverage criteria [46] (Table S11).

Implementation-independent replication was performed by L.R.R., who had developed neither the source implementation nor the figure-production pipeline and recoded the models from frozen specifications rather than rerunning source scripts. J.D.M.V. developed the source analytical implementations and figure-production pipeline; L.R.R. developed the independent validation implementation and is the designated human validator for the final analytical outputs. Because the replicator had participated in quantitative extraction and evidence appraisal, the exercise was implementation-independent but not fully outcome-blinded; reference outputs remained inaccessible until the independent fits and continuous reconstructions had been archived. Analytic inputs, frozen specifications, code, priors, seeds, acceptance criteria, software-environment records, machine-readable results, and implementation-independent replication materials are archived in Zenodo version 1.0.0 (doi:10.5281/zenodo.21780525) and in the updated version 1.1 reproducibility package supporting the current submission (doi:10.5281/zenodo.22086455). Replication covered the primary analyses and the sensitivity configurations listed in Table S11.

For binary models, agreement required absolute differences ≤0.10 in posterior median log-odds ratios, ≤0.20 at either 95% interval limit, and ≤0.15 in median τ; continuous estimates, confidence- and prediction-interval limits, and heterogeneity estimates had to agree within 1 × 10^−4^. Interval classification relative to the null was evaluated separately as a secondary criterion. One numerically concordant near-boundary discrepancy prompted a post hoc boundary-indeterminate category without altering any frozen input, fitted model, reference output, or original replication result. Detailed iteration schedules, diagnostic and calibration summaries, numerical comparisons, and boundary-adjudication records are provided in Table S11 and the reproducibility package.

For Supplementary Data S1 (CSV file), L.R.R. independently rederived all 147 binary active–active contrasts from the retained posterior draws and compared them row by row with the reported values. Acceptance required every contrast to agree with its rederived value within a predefined absolute tolerance of 5 × 10^−5^. The executable script, row-level output, summary, and completed validation record are included in the version 1.1 reproducibility package.

Generative AI use in manuscript preparation. After completion of study selection, quantitative extraction, evidence appraisal, statistical analysis, interpretation, and figure production, ChatGPT (OpenAI; model 5.6; version 1.2026.184 [1784145287]) was used solely to assist with English-language editing, concision, readability, and manuscript organization. It did not determine study eligibility, extract or analyze data, assess risk of bias or confidence, or determine scientific conclusions; all outputs were reviewed and edited by the authors. Product and version details are reported in the Acknowledgments.

## 3. Results

### 3.1. Study Selection and Analytical Disposition

The record-, report-, entry-, cohort-, participant-, and analytical-disposition identities are reconciled in Table S12A–C.

**Figure 1:**
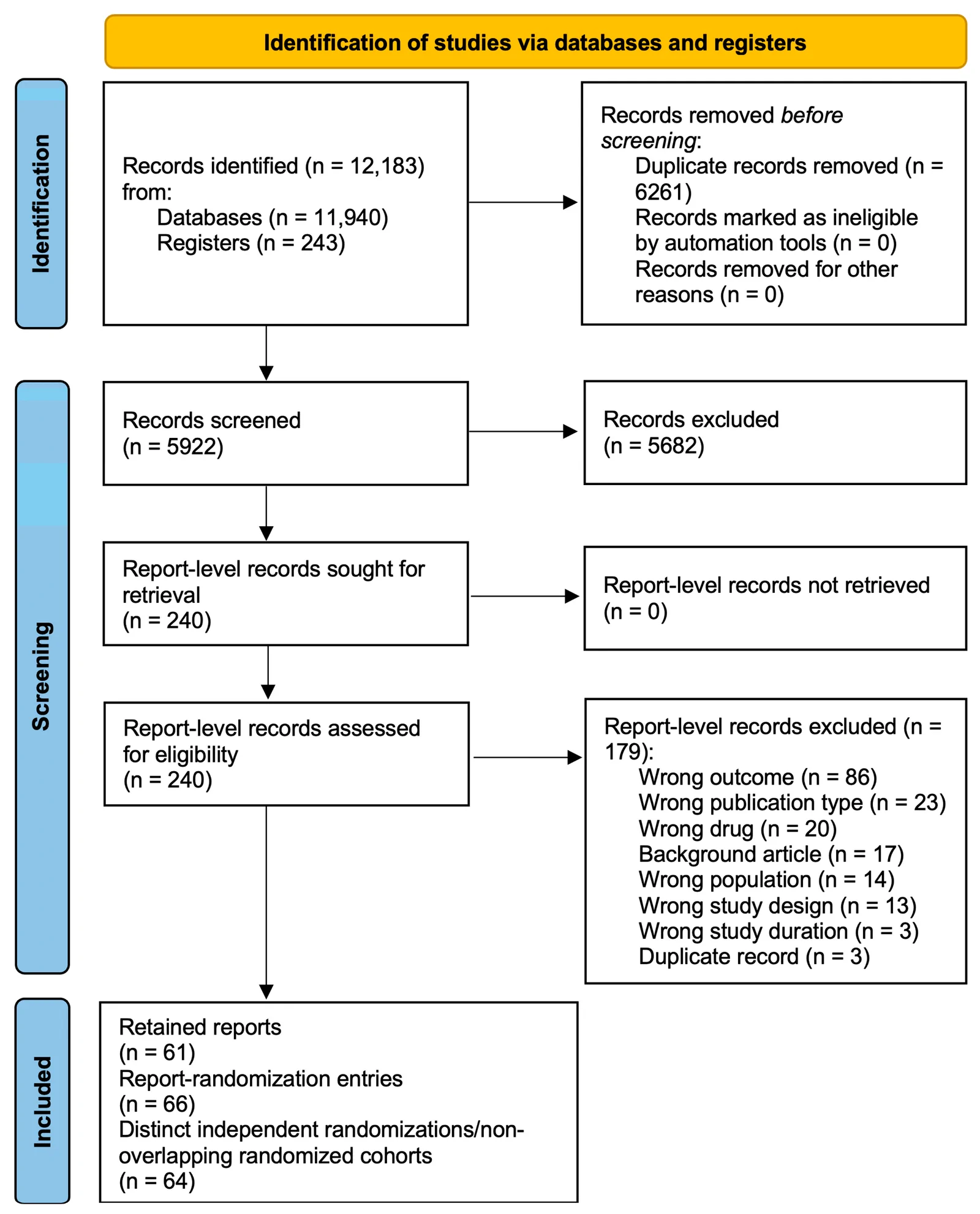
PRISMA 2020 flow diagram [13] (also provided as Supplementary Figure S1). The 240 report-level records assessed for eligibility represented 237 unique reports because three were duplicate bibliographic representations of reports already retained. Thus, the 179 report-level exclusion decisions comprised 176 substantive exclusions and three duplicate-report records, while 61 unique reports were retained (237 = 61 + 176; 240 = 237 + 3). The 61 retained reports generated 66 report–randomization entries; two were non-incremental companion links to randomizations already represented by primary reports, yielding 64 distinct independent randomizations/non-overlapping randomized cohorts comprising 34,831 randomized participants (66 − 2 = 64). Of these 64 cohorts, 35 contributed model-ready data to at least one principal evidence structure, 28 were retained only in the evidence map, and one was contextual/sensitivity-only (64 = 35 + 28 + 1). All 240 report-level records sought for retrieval were retrieved in an evaluable form (not retrieved, *n* = 0); 41 unique reports were available only as abstracts because no more complete report could be identified, and all were excluded after eligibility assessment. No records were identified through other methods (*n* = 0).

The searches identified 12,183 records: 11,940 from bibliographic databases and 243 from trial registers. After 6261 duplicate records were removed, 5922 records underwent title-and-abstract screening, of which 5682 were excluded. All 240 report-level records sought for retrieval were retrieved in an evaluable form (not retrieved, *n* = 0) and assessed for eligibility. These 240 records represented 237 unique reports because three were duplicate bibliographic representations of reports already retained. The unique-report set comprised 61 retained reports and 176 excluded reports. The 179 report-level exclusion decisions comprised 176 substantive exclusions and three duplicate-report records, each assigned one mutually exclusive final classification shown in Figure 1. The 61 retained reports [18,19,20,26,47,48,49,50,51,52,53,54,55,56,57,58,59,60,61,62,63,64,65,66,67,68,69,70,71,72,73,74,75,76,77,78,79,80,81,82,83,84,85,86,87,88,89,90,91,92,93,94,95,96,97,98,99,100,101,102,103] generated 66 report–randomization entries that mapped to 64 distinct independent randomizations/non-overlapping cohorts comprising 34,831 randomized participants. Two companion entries represented randomizations already described by primary reports and therefore did not increase study or participant denominators. The duplicate classification did not alter any comparative-effect or standard-error input, fitted model, treatment-effect estimate, or scientific conclusion.

All 240 report-level records had at least one evaluable source and were therefore classified as retrieved. Forty-one unique reports were available only as abstracts because no more complete report could be identified; none met the eligibility criteria. Their abstract-only status was retained in the eligibility ledger and exclusion audit. Detailed model-ready characteristics and denominator provenance are reported in Table S13.

Thirty-three reports describing 35 distinct randomized cohorts contributed model-ready data to at least one of the nine principal evidence structures. Of the other 29 unique retained cohorts, 28 were evidence-map-only, and one (Prenner et al. [91]; *n* = 1095) was retained as contextual/sensitivity-only evidence; none contributed to the principal NMA. Gutkowski et al. [66] remained an evidence-map-only randomized cohort: its terfenadine–dexchlorpheniramine comparison did not satisfy the principal comparator definition and was not inserted into a principal network. Non-modeling reflected absence of a target result; incompatibility of the population, comparison, construct, scale, treatment period, or analysis population; incomplete or unrecoverable uncertainty/event data; or contextual-only disposition. Decisions were based on estimand compatibility and numerical identifiability, not effect direction, magnitude, or statistical significance. The whole-corpus crosswalk reconciles all 61 retained reports, 66 report–randomization entries, and 64 non-overlapping randomized cohorts. A separate analysis-valid denominator of 34,811 participants reflects the prespecified sensitivity exclusion of 20 participants from one post-randomization center and is not a principal corpus or network denominator (Table S12C).

### 3.2. Composition, Geometry, and Transitivity of the Quantitative Evidence

**Figure 2:**
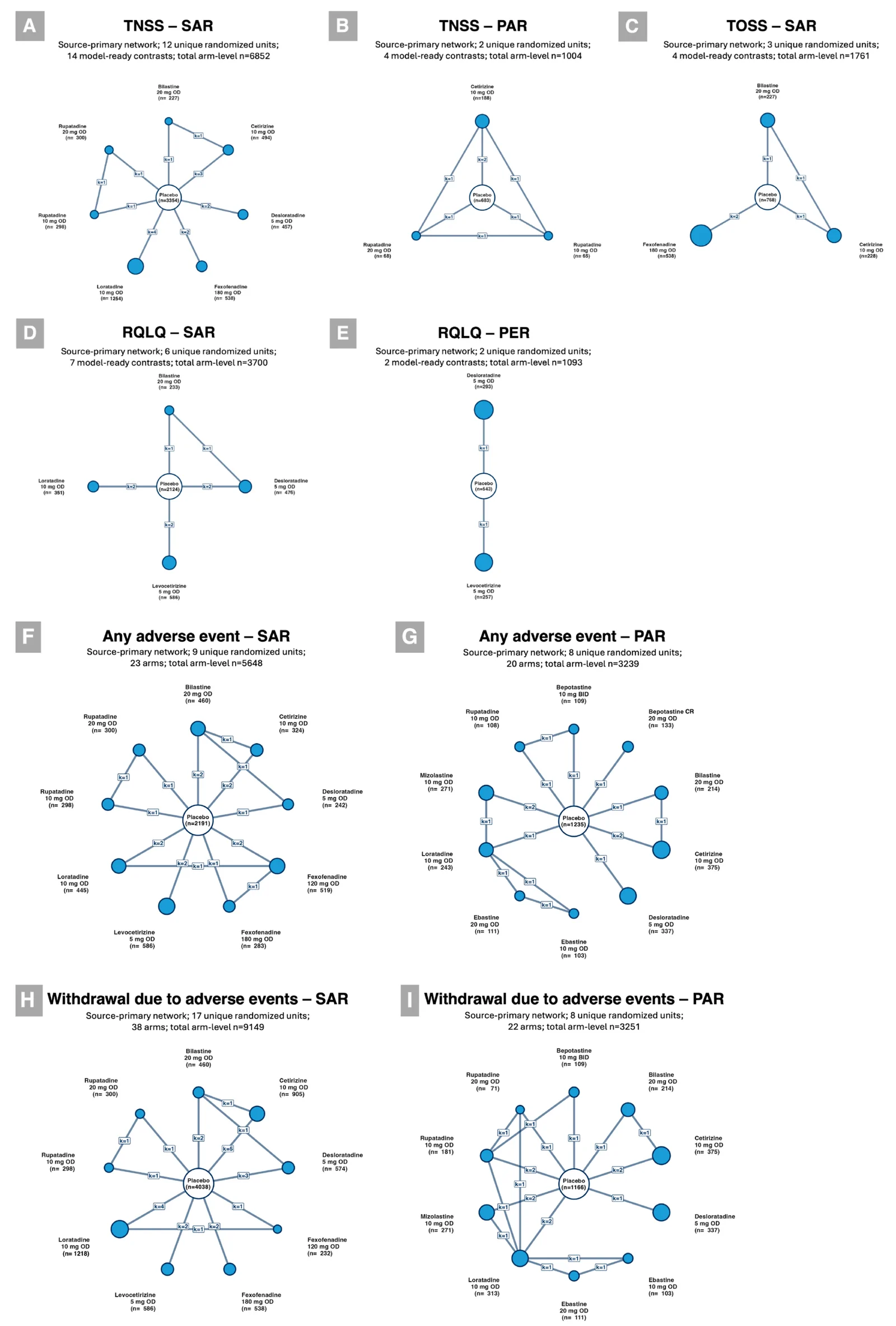
Direct-comparison geometry and structural estimability of the nine principal rhinitis-category–outcome evidence structures: (**A**–**E**) continuous efficacy and quality-of-life structures. Nodes denote regimen-level treatments and edges denote direct randomized comparisons. Node area is proportional to accumulated arm-level denominator; edge labels show the number of unique randomized units containing the comparison. Edge counts are non-additive in multi-arm trials. Geometry represents evidence quantity and connectivity, not effect magnitude, ranking, or confidence. Source-audited node areas for the Van Adelsberg et al. (2003) trial [18] in panels A and D use 451 placebo, 448 montelukast, and 180 loratadine participants; these source-verified values affect node-size metadata only. (**F**–**I**) Binary tolerability structures. In WDAE–SAR, one all-zero three-arm unit (*n* = 900) was retained structurally but excluded from the likelihood; rupatadine 10 mg and 20 mg were therefore non-estimable.

The 64 distinct randomized cohorts arose from placebo- or active-controlled parallel-group trials with at least two arms. Five continuous and four binary category–outcome structures were analyzed; the 35 modeled units generated 67 randomization–structure contributions. All nine structures were connected, but their information content differed substantially: RQLQ–PER was saturated, WDAE–SAR contained two structurally present but non-estimable active nodes, and assessment of incoherence was not feasible or robustly identifiable in several structures. Table 1 summarizes their composition; Figure 2, panels A–E and F–I, shows direct-comparison geometry and structural estimability; and Table S8A,B and Figures S8–S16 provide the corresponding audit and individual network displays.

The nine structures represented all model-ready combinations within the principal framework. No principal model-ready structure was available for TNSS–PER, RQLQ–PAR, Any AE–PER, or WDAE–PER at the target periods; longer-horizon PER harm data entered sensitivity analyses only. TOSS–PAR and TOSS–PER were outside the principal target framework.

Fourteen regimen-level nodes represented ten agents: bilastine, cetirizine, levocetirizine, desloratadine, loratadine, fexofenadine, rupatadine, ebastine, mizolastine, and bepotastine. No structure contained all 14 nodes. Eight nodes contributed to at least one efficacy or quality-of-life structure, whereas six—fexofenadine 120 mg, ebastine 10 and 20 mg, mizolastine 10 mg, bepotastine 10 mg twice daily, and controlled-release (CR) bepotastine 20 mg—appeared only in tolerability structures and therefore lacked corresponding efficacy or quality-of-life estimates.

Candidate effect modifiers were incompletely reported. Rhinitis category, treatment period, outcome definition, and assessment timing were generally recoverable, whereas allergen exposure, baseline symptom burden, placebo response, cointerventions, and some age-distribution characteristics were less consistently reported (Table S9). A quantitative assessment of transitivity was therefore not feasible, and exchangeability was inferred from neither pharmacological class membership nor failure to detect incoherence.

CINeMA indirectness assessments [35] raised some concerns for 81 of the 89 evaluable estimates and major concerns for eight: five of six TNSS–PAR estimates, two of 28 TNSS–SAR estimates, and one of nine Any AE–SAR estimates. The two non-estimable WDAE–SAR effects were not evaluable. These judgments informed comparison-specific confidence ratings and interpretation of indirect and active–active estimates.

### 3.3. Nasal and Ocular Symptom Outcomes

**Figure 3:**
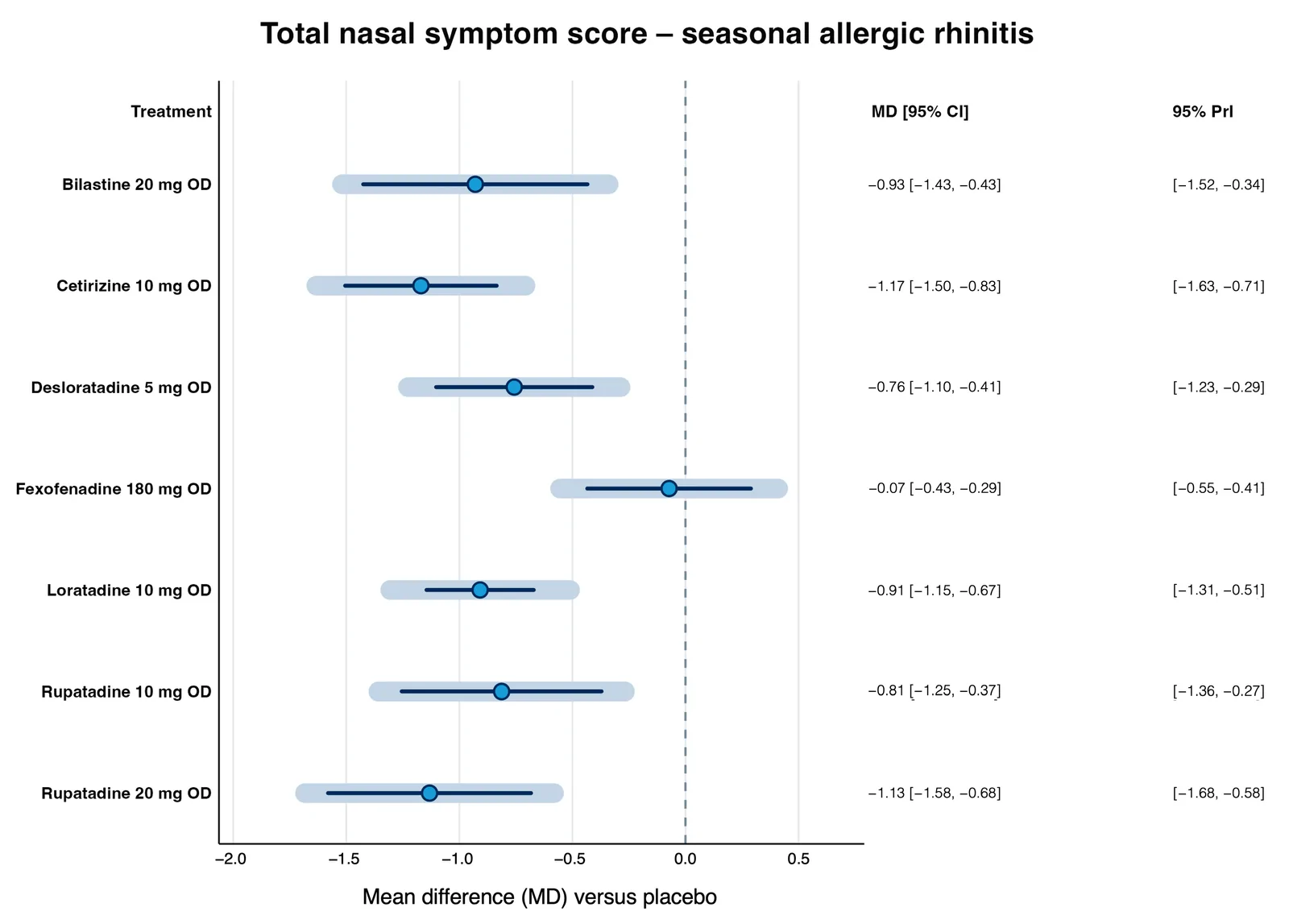
Mean differences (MDs) versus placebo for total nasal symptom score (TNSS) in SAR. The vertical dashed line indicates the null value (MD = 0). Negative values favor active treatment. Dark lines represent 95% confidence intervals; pale lines represent plug-in 95% prediction intervals conditional on the restricted maximum likelihood (REML) estimate of τ.

*Total nasal symptom score.* In SAR, six of seven placebo-referenced estimates had 95% confidence intervals entirely below zero, ranging from cetirizine 10 mg (MD −1.17, 95% CI −1.50 to −0.83) to desloratadine 5 mg (MD −0.76, 95% CI −1.10 to −0.41); their plug-in prediction intervals, conditional on the REML estimate of τ, also remained below zero. Fexofenadine 180 mg was close to the null and imprecise (MD −0.07, 95% CI −0.43 to 0.29; prediction interval −0.55 to 0.41) (Figure 3). Estimated heterogeneity was τ = 0.163 (profile-likelihood 95% CI 0–0.482; descriptive I^2^ = 41.3%).

In PAR, estimates were below zero for rupatadine 20 mg (MD −1.06, 95% CI −1.83 to −0.30), rupatadine 10 mg (MD −0.80, 95% CI −1.53 to −0.07), and cetirizine 10 mg (MD −0.50, 95% CI −0.87 to −0.12) (Figure S2). The heterogeneity estimate (τ = 0.108) and prediction intervals were weakly informed by only two randomized units.

*Total ocular symptom score*. In SAR, estimates were below zero for cetirizine 10 mg (MD −0.85, 95% CI −1.30 to −0.40) and bilastine 20 mg (MD −0.74, 95% CI −1.18 to −0.30), whereas the fexofenadine 180 mg interval crossed the null (MD −0.07, 95% CI −0.36 to 0.23) (Figure S3). Heterogeneity (τ = 0.132; descriptive I^2^ = 39.0%) and prediction intervals were weakly informed by three randomized units. Complete placebo-referenced continuous estimates are reported in Table S15; generalized Q, residual df, *p*-values, τ profile intervals, and descriptive I^2^ for all continuous structures are reported in Table S33.

### 3.4. Disease-Specific Quality of Life

In SAR, estimates were below zero for desloratadine 5 mg (MD −0.38, 95% CI −0.55 to −0.22), bilastine 20 mg (MD −0.34, 95% CI −0.55 to −0.14), loratadine 10 mg (MD −0.31, 95% CI −0.44 to −0.18), and levocetirizine 5 mg (MD −0.17, 95% CI −0.32 to −0.03) (Figure S4). All point estimates were smaller in absolute magnitude than the contextual 0.5-point benchmark; the lower confidence limits for desloratadine and bilastine were −0.55.

The REML estimate of τ was at the boundary of zero and was not informatively estimated; no plug-in prediction interval was reported. Bayesian sensitivity analyses nevertheless retained material uncertainty about heterogeneity.

In PER, mean differences were −0.48 for levocetirizine 5 mg (95% CI −0.67 to −0.29) and −0.30 for desloratadine 5 mg (95% CI −0.49 to −0.11) (Figure S5). This two-unit structure was saturated, precluding estimation of heterogeneity, prediction intervals, and incoherence.

### 3.5. Tolerability Outcomes

Binary estimates, heterogeneity diagnostics, and source/prior sensitivities are reported in Tables S16–S20.

**Figure 4:**
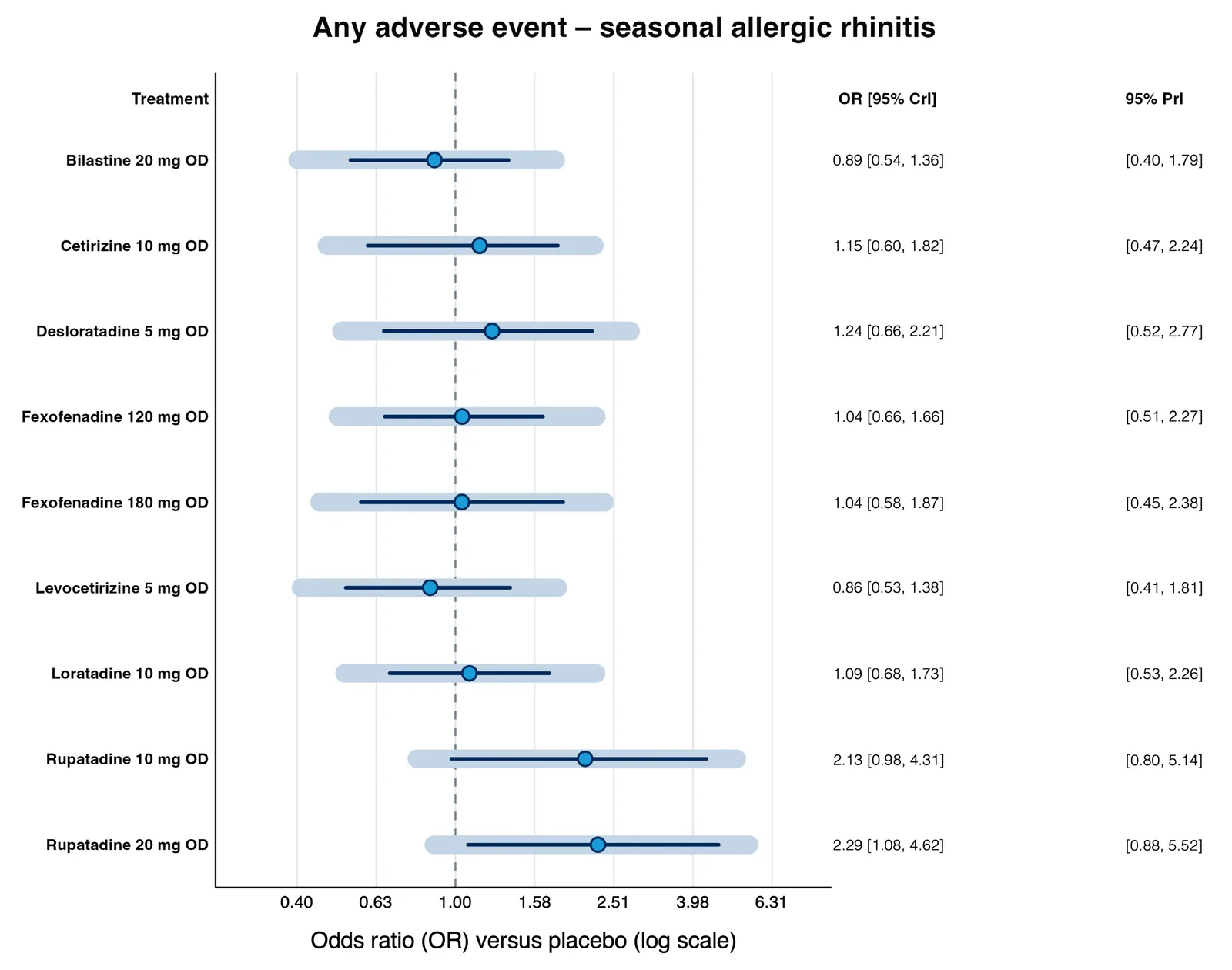
Posterior odds ratios (ORs) versus placebo for any adverse event in SAR under the primary half-normal heterogeneity prior with scale 0.5. The vertical dashed line indicates the null value (OR = 1). Dark and pale lines represent 95% credible and prediction intervals, respectively.

*Any adverse event.* In SAR, 1351 of 5648 participants experienced at least one event. Under the primary half-normal heterogeneity prior with scale 0.5, most estimates were imprecise, and all 95% prediction intervals included one (Figure 4). Rupatadine 20 mg was the only regimen whose credible interval excluded one (OR 2.29, 95% CrI 1.08–4.62), although its prediction interval included one (0.88–5.52). Under the wider half-normal heterogeneity prior with scale 1.0, its posterior median was similar, but the credible interval included one (OR 2.26, 95% CrI 0.99–4.70). Under the primary prior, rupatadine 10 mg yielded an OR of 2.13 (95% CrI 0.98–4.31). The direct 20 mg versus 10 mg contrast was imprecise (unadjusted OR 1.08, two-sided 95% conditional exact CI 0.67–1.74). Posterior median τ under the primary prior was 0.182 (95% CrI 0.010–0.606).

In PAR, 1080 of 3239 participants experienced at least one event. Posterior median ORs ranged from 0.54 to 1.65; all credible and prediction intervals included one. Posterior median τ was 0.202 (95% CrI 0.012–0.656) (Figure S6).

**Figure 5:**
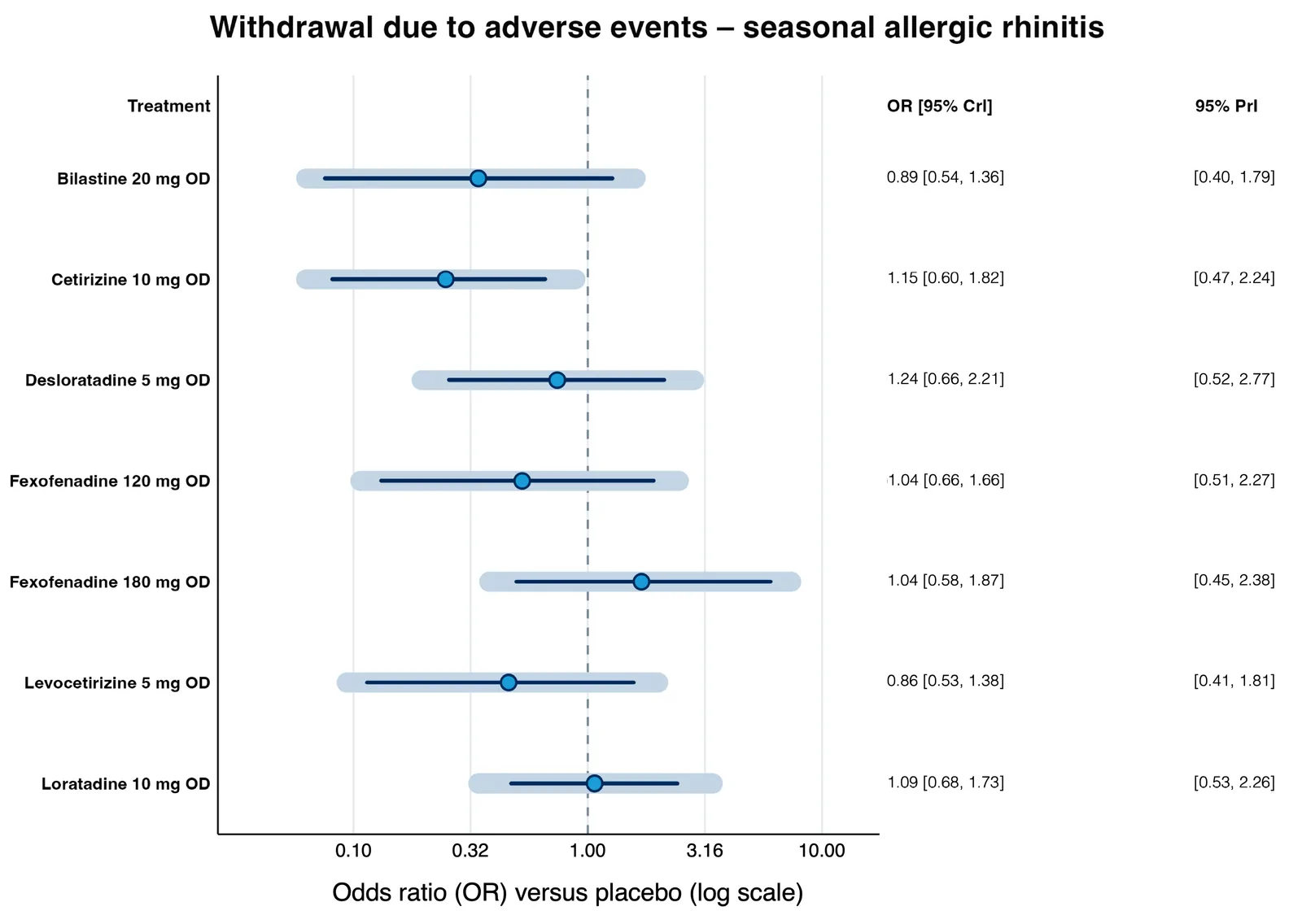
Posterior odds ratios (ORs) versus placebo for withdrawal due to adverse events (WDAE) in SAR under the primary half-normal heterogeneity prior with scale 0.5. The vertical dashed line indicates the null value (OR = 1). Dark and pale lines represent 95% credible intervals and 95% prediction intervals, respectively. Rupatadine 10 mg and 20 mg were non-estimable because their only contributing randomized unit had zero withdrawals in every arm.

*Withdrawals due to adverse events.* In SAR, 83 withdrawals occurred among 9149 participants from 17 randomized units. One three-arm unit had zero withdrawals in every arm and contributed no relative-effect information, leaving 16 units and 8249 participants in the likelihood; rupatadine 10 mg and 20 mg were consequently non-estimable (Table 1). Under the primary heterogeneity prior, cetirizine 10 mg was the only estimable regimen with both its credible and prediction intervals below one (OR 0.25, 95% CrI 0.08–0.66; 95% prediction interval 0.06–0.89) (Figure 5). Under the wider half-normal heterogeneity prior with scale 1.0, its posterior median and credible interval were similar (OR 0.25, 95% CrI 0.08–0.69), but the prediction interval included one (0.05–1.10). Credible intervals for all other estimable regimens included 1 under the primary prior. Posterior median τ under that prior was 0.271 (95% CrI 0.013–0.862).

In PAR, 55 of 3251 participants withdrew because of an adverse event. Posterior median ORs ranged from 0.35 to 1.69; all credible and prediction intervals included one. Posterior median τ was 0.272 (95% CrI 0.012–0.903) (Figure S7). One unit used uniquely reconstructed event counts (11/337 and 7/339), retained with explicit derivation flags. Table S14 reports absolute-risk estimates for all four binary structures, and Table S10 reports the heterogeneity-prior sensitivities.

### 3.6. Active–Active Evidence and Incoherence

Of the 34 continuous active–active comparisons, seven were directly informed, and 27 were indirect only through placebo; none had independently estimable direct and indirect components. None reached moderate or high CINeMA confidence [35], and intervals were generally wide relative to plausible between-regimen differences. Complete continuous active–active estimates, evidence routes, component-estimability status, and comparison-specific appraisals are reported in Tables S26 and S27. For the binary structures, Table S27 reports all 36 estimable placebo-referenced ORs and 95% CrIs from the frozen source-primary file used for Tables S16–S20 and Figure 4 and Figure 5; two WDAE–SAR effects remained non-estimable.

The retained implementation-independent posterior draws yielded all 147 estimable binary active–active contrasts: 36 for Any AE–SAR, 45 for Any AE–PAR, 21 for WDAE–SAR, and 45 for WDAE–PAR. Overall, 141 of 147 estimates had 95% CrIs including one and six excluded one; all six were indirect-only. Because these secondary estimates were not multiplicity-adjusted or formally appraised with CINeMA, none were considered confirmatory or used to support treatment ranking, superiority, equivalence, or preferential tolerability. Complete estimates and evidence-route classifications are reported in Table S31 and Supplementary Data S1 (CSV file).

All graph-cycle dimensions arose within multi-arm randomizations—one per three-arm and three per four-arm designs—and no additional cycle dimension spanned distinct randomized designs.

Global design-by-treatment interaction tests [32,33] were estimable for Any AE–SAR, RQLQ–SAR, TNSS–PAR, and TNSS–SAR, with *p*-values of 0.984, 0.279, 0.317, and 0.065, respectively. The first three provided little indication of material incoherence. TNSS–SAR was least reassuring, although its structure was sparse and weakly replicated, and all tests had limited power. These global tests can be estimable through treatment-by-design differences and design replication even when no local direct–indirect contrast is separately identifiable (Table S9).

RQLQ–PER was saturated, and TOSS–SAR had zero additional degrees of freedom for the global design-by-treatment test. In Any AE–PAR, WDAE–SAR, and WDAE–PAR, local direct–indirect separation was not robustly identifiable, and a global test was not estimated after the final source-verified freeze. For the 27 estimable placebo-referenced effects in these structures, the CINeMA incoherence domain was therefore conservatively rated as major concerns; the two non-estimable WDAE–SAR effects remained not evaluable. This domain judgment reflected unresolved incoherence evaluability rather than evidence that incoherence was present, and absence of a test was not interpreted as evidence of consistency (Tables S9 and S27).

### 3.7. Sensitivity and Exploratory Analyses

Restrictions based on source completeness and scale compatibility materially reduced several structures: TNSS–SAR to two randomized units and a saturated three-contrast structure, TNSS–PAR to one multi-arm unit, and RQLQ–PER to one direct comparison. Source verification confirmed that the primary Kuna et al. score [26] comprised only tearing, ocular redness, and ocular itching, each scored 0–3 (total 0–9), so it satisfied the prespecified ocular-only TOSS rule. No separate construct-restriction sensitivity was retained after this verification. Exploratory TOSS–PAR was appraisal-only and did not enter a treatment-effect model. Detailed scenarios are reported in Table S10.

Alternative treatment periods, event definitions and denominators, partial-arm exclusions, model assumptions, and heterogeneity priors affected estimates and uncertainty. Widening the binary heterogeneity prior had little effect on posterior medians but altered different interval components: for cetirizine in WDAE–SAR, the credible interval remained below one while the prediction interval widened to include one; for rupatadine 20 mg in Any AE–SAR, the credible interval itself widened to include one. Interval-based descriptors were therefore sensitive to heterogeneity assumptions in these sparse structures (Section 3.5; Table S10).

Four individual exploratory evidence-expansion analyses examined mixed-age eligibility, broader scale/rater compatibility, a per-protocol population, and inclusion of the four-arm, placebo-controlled trial by Kowalski et al. (2009) [71]. Adding that trial introduced the loratadine node; because covariance among its adjusted contrasts was unreported, it was approximated as zero, changing the rupatadine 10 mg estimate from −0.80 to −0.91. A separate combined TNSS–PAR stress test—not counted as a fifth individual analysis—jointly applied the Bachert, Kowalski, and Mo assumptions. Because these scenarios altered the evidence composition and at least one eligibility, population, estimand, scale-compatibility, or covariance assumption, they were treated as exploratory evidence expansion rather than conventional robustness analyses. None yielded a stable, outcome-independent treatment hierarchy (Tables S10 and S18).

The four additional TNSS–PAR RoB 2 records corresponded to result-specific exploratory or sensitivity layers from Bachert et al. (1998) [49], Davies et al. (1998) [60], Kowalski et al. (2009) [71], and Mo et al. (2021) [80]. One additional appraisal-only exploratory TOSS–PAR result from Bachert et al. (1998) [49] was retained in the RoB 2 panel; it was not a principal or pooled quantitative synthesis and is documented in Tables S10 and S21A.

### 3.8. Risk of Bias, Missing Evidence, and Confidence

Of 73 result-specific RoB 2 assessments [34], 49 raised some concerns, and 24 were at high risk; none were at low risk. Counts are result-specific rather than unique primary-model randomizations: TNSS–SAR includes one additional sensitivity/exploratory result, TNSS–PAR four additional results, and TOSS–PAR one appraisal-only exploratory result (Table S21A; Figure S17). Full domain-level assessments, actual risk windows, domain evidence summaries, and overall-judgment rationales are reported in Table S30 and *RoB2_Result_Level_Assessments_v1.1.csv*.

Of 91 ROB-MEN judgments [41], 85 raised some concerns, four were at low risk, and two were not rateable because the corresponding effects were non-estimable. The four low-risk judgments were RQLQ–SAR levocetirizine versus placebo, Any AE–SAR levocetirizine versus placebo, and WDAE–SAR cetirizine and levocetirizine versus placebo. This 85/4/2 distribution was unchanged across the 10%, 15%, 20%, and 25% project-specific contribution indicators. Under a separate conservative small-study-effect policy, those four judgments were classified as some concerns, yielding 89 some concerns and two not-rateable judgments (Table S27; Figures S19 and S31).

All 89 estimable comparisons within the defined CINeMA appraisal scope were rated very low confidence; the two non-estimable WDAE–SAR effects were not evaluable (Table S21; Figures S18 and S29). The 147 secondary binary active–active contrasts were not formally appraised with CINeMA and are not included in these counts. The incoherence domain comprised 25 no-concerns, 28 some-concerns, 36 major-concerns, and two not-evaluable judgments. Heterogeneity-domain judgments were comparison-specific rather than uniform by evidence structure and followed the project rule based on estimability and whether predictive uncertainty altered the comparison-specific clinical interpretation, not on the numerical magnitude of τ alone. Within TNSS–SAR, 23 comparisons had no concerns, one had some concerns, and four had major concerns; the fexofenadine 180 mg versus placebo comparison was the single some-concerns judgment. All 10 RQLQ–SAR comparisons had some concerns because τ lay at a non-informative zero boundary, no plug-in prediction interval was supportable, and Bayesian τ uncertainty persisted. Continuous numerical heterogeneity diagnostics are reported in Table S33.

The automated consistency audit linking Tables S16–S20 and S27 with Figure 4 and Figure 5 passed for all 38 binary rows. Table S27 reports the source-primary binary ORs and 95% CrIs, and Table S32 reports the canonical treatment identifiers, Table S20 boundary classifications, and row-level display checks. No discrepancy was identified among the linked tables and figures (Table S32).

The binary-specific and all-structure contribution-matrix audits passed projection, conservation, aggregation, and column-sum checks, confirming arithmetic consistency only and not altering confidence ratings (Table S22A,B and Figures S20–S28).

### 3.9. Computational Replication

All 12 binary scenarios satisfied the frozen sampling-diagnostic criteria, and all 12 heterogeneity comparisons met the predefined numerical tolerance. Continuous results were reproduced across ten GLS/REML scenarios and three direct components; all four primary binary source–replication comparisons also met the numerical tolerances (Table S23).

Interval classifications relative to the null agreed in three of the four primary binary comparisons. The sole label discrepancy involved a numerically concordant result near the null boundary and was classified post hoc as boundary-indeterminate. This classification altered no frozen input, fitted model, reference output, or original replication result; exact-label agreement therefore remained three of four. All 30 simulation-based calibration replicates met the predefined aggregate acceptance criteria [46].

Final row-level validation by L.R.R. confirmed that all 147 secondary binary active–active contrasts agreed with the independently rederived values within the predefined tolerance (maximum absolute difference, 5.0000049 × 10^−10^); no model was refitted, and no additional MCMC sampling was performed.

## 4. Discussion

### 4.1. Principal Findings

This review neither established therapeutic equivalence nor identified a reliable efficacy or tolerability hierarchy among oral second-generation H1-antihistamine regimens. Several regimens had favorable placebo-referenced estimates, but effect magnitude, uncertainty, predictive distributions, structural support, and confidence varied by outcome. The evidence was predominantly placebo-anchored, active–active evidence was sparse and weakly replicated, and several structures contained only two or three randomized units. Within the defined CINeMA appraisal scope, all 89 estimable comparisons were rated very low confidence, while two non-estimable effects were not evaluable [35]; the 147 secondary binary active–active contrasts were not formally appraised with CINeMA. The results therefore identify where comparative effects are estimable and how uncertain they remain, rather than supporting global treatment differentiation.

### 4.2. Outcome-Specific Patterns and Harms

Cetirizine 10 mg was the only regimen represented in all three principal symptom structures—TNSS–SAR, TNSS–PAR, and TOSS–SAR—and had favorable placebo-referenced estimates in each; it also had a favorable WDAE–SAR estimate under the primary model. The TOSS–SAR structure used the source-labeled three-item ocular non-nasal score from Kuna et al. (2009) [26], verified as tearing, redness, and itching scored 0–3 and therefore construct-equivalent to the prespecified 0–9 TOSS. This was the broadest favorable cross-structure pattern observed, but not a formally estimated overall treatment profile. Bilastine 20 mg had favorable estimates in each of the three SAR benefit structures in which it was represented—TNSS, TOSS, and RQLQ. Rupatadine, particularly 20 mg, yielded among the larger nasal-symptom point estimates in SAR and PAR after the Okubo et al. 0–16 T4NSS [20] was exactly transformed to the common 0–12 scale; its tolerability evidence remained inconclusive. Desloratadine, loratadine, and levocetirizine also had favorable RQLQ estimates.

All SAR RQLQ point estimates were smaller in absolute magnitude than the contextual 0.5-point benchmark. Because this value was derived for within-patient change rather than between-group effects, it establishes neither clinical unimportance nor an expected perceptible benefit for individual patients; no validated between-group threshold is available [27,28]. These cross-structure observations are descriptive and conditional on unequal outcome coverage. They arose from separately fitted, predominantly placebo-anchored structures involving different studies, populations, and treatment periods and were neither defined by a prespecified cross-outcome composite nor evaluated in a joint multivariate or benefit–harm model. Given the very-low-confidence evidence within the CINeMA-appraised comparisons, they do not establish active–active superiority, an overall benefit–harm advantage, or a treatment ranking. Likewise, fexofenadine 180 mg estimates close to the null in the eligible SAR nasal and ocular structures indicate imprecise average effects, not pharmacological inactivity or equivalence to placebo.

Evidence on harms was less informative. Any AE combines events differing in severity, duration, attribution, background frequency, and importance to patients and is sensitive to ascertainment and reporting [24,25]. For rupatadine 20 mg in Any AE–SAR, the credible interval excluded one only under the primary heterogeneity prior, the prediction interval included one under both priors, and the direct 20 mg versus 10 mg comparison was imprecise; the rupatadine WDAE–SAR effects were non-estimable. For cetirizine in WDAE–SAR, both intervals were below one under the primary prior, but the prediction interval included one under the wider prior. Posterior medians changed little, indicating sensitivity of interval classification rather than of the central estimates. Given the 83 withdrawals and very low confidence of the formally appraised evidence, these findings establish neither a robust safety signal nor a tolerability hierarchy.

### 4.3. Evidence Structure, Active–Active Comparisons, and Rankings

Of the 34 active–active comparisons in the continuous structures, 27 were indirect-only through placebo, and seven were directly informed within multi-arm randomizations. None had independently estimable direct and indirect components or reached moderate or high CINeMA confidence [35]. The 147 secondary binary active–active contrasts were not formally appraised with CINeMA or multiplicity-adjusted: 141 had 95% credible intervals including one, and six excluded one, all six being indirect-only. These findings were therefore not considered confirmatory. Differences between placebo-referenced estimates, or isolated non-null secondary active–active contrasts, cannot support reliable claims of superiority, equivalence, or preferential tolerability between active regimens.

The evidence map was also asymmetric across benefit and harm domains: six of the 14 regimen-level nodes contributed only to tolerability structures (Section 3.2). Their harm estimates cannot support a balanced regimen-specific benefit–harm assessment, and absent efficacy or quality-of-life estimates do not imply a lack of efficacy. A universal ranking would therefore overstate the evidence’s capacity to discriminate among regimens. Rankings are conditional on the included studies, nodes, outcomes, and modeling assumptions and do not convey non-estimability, sparse replication, missing benefit or harm domains, or confidence in the underlying comparisons [11].

### 4.4. Relation to Previous Evidence and Clinical Context

Previous network meta-analyses pooled more broadly and reported comparative rankings [9,10]. Four design features help explain the different conclusions. First, this review used independent randomization rather than publication as the evidence unit and linked reports within trial families, reducing double counting and underestimation of uncertainty. Second, treatment nodes were regimen-specific: dose-specific regimens were retained separately because their evidence bases and outcome coverage differed, without assuming interchangeability. Third, eligibility required compatibility of construct, components, respondent, temporal aggregation, summary statistic, rhinitis category, and treatment period, avoiding apparent precision gained by pooling non-equivalent information. Fourth, sparse binary outcomes were analyzed with arm-level binomial-logit models without continuity correction, preserving the distinction between a small effect and an unidentified one. In the all-zero rupatadine WDAE–SAR unit, no relative-effect information entered the likelihood; a prior could not create empirical comparative evidence where none existed [17,29], whereas contrast-level normal approximations with added pseudo-counts would have produced apparently informative estimates.

Preserving multi-arm covariance and accepting event counts only when denominators and risk periods were source-verified or uniquely reconstructable likewise reduced the modeled evidence and widened intervals. This review therefore does not directly refute earlier rankings; it finds the available evidence insufficient to justify one. The ARIA–EAACI evidence program similarly rated many oral-treatment comparisons as having low or very low certainty [6,10].

This analysis concerns differentiation within oral H1-antihistamine monotherapy, not oral versus intranasal treatment. Intranasal corticosteroids generally provide stronger control of persistent or prominent nasal symptoms, particularly obstruction [3,4,5,6,104], whereas oral antihistamines remain reasonable when histamine-mediated nasal or ocular symptoms, convenience, or patient preference predominate [3,4,5,6,7,8].

Fixed cetirizine–pseudoephedrine combinations were excluded because they constitute a distinct intervention and benefit–harm estimand. According to the Spanish product information, the prolonged-release formulation is indicated for allergic rhinitis with nasal congestion when both antihistaminic and decongestant effects are required; it is administered twice daily for a limited symptomatic period, generally not exceeding 2–3 weeks [105]. Any incremental benefit over cetirizine alone reflects the pseudoephedrine component and must be weighed against its adverse effects, contraindications, and restrictions on systemic decongestant use [3,4,106,107]. Findings from combination arms therefore provide no additional evidence for, and do not alter, the cetirizine-monotherapy estimates synthesized here.

### 4.5. Implications for Practice

Within oral second-generation H1-antihistamine monotherapy, the principal clinical implication is restraint: comparative trial evidence does not reliably identify a preferred active regimen for any outcome examined, nor does it establish therapeutic equivalence. Selection cannot therefore rely on comparative evidence alone. Acquisition cost, dosing convenience, formulation, interaction potential, renal or hepatic considerations, occupational and driving requirements, previous response, patient preference, and relevant pharmacological evidence may reasonably guide choice.

Claims of superiority between active regimens remain based largely on indirect comparisons or secondary contrasts that do not provide sufficiently robust comparative evidence. This review cannot support comparative conclusions about sedation because somnolence, psychomotor performance, and driving safety were too inconsistently reported for synthesis; dedicated pharmacological and experimental evidence should inform these considerations [7,8]. Although RQLQ estimates favored active treatment, their average magnitudes cannot be translated into an expected individual benefit because the 0.5-point benchmark concerns within-patient change and no validated between-group threshold exists (Section 4.2).

Clinical selection should therefore begin with the patient’s dominant symptoms, comorbidities, safety constraints, previous response, and preferences rather than a numerical rank [3,4,5,6,7,8]. Outcome-specific estimates may inform but cannot replace individualized shared decision-making.

### 4.6. Strengths and Limitations

Strengths included report mapping to independent randomizations, retrospective whole-corpus audit, independent duplicate quantitative extraction, outcome-specific estimands, regimen-level nodes, preservation of multi-arm covariance, and explicit handling of non-estimability. Documented eligibility, result-selection, and estimand-compatibility rules constrained analytic discretion irrespective of effect direction. Risk-of-bias, confidence, and missing-evidence appraisals were conducted independently in duplicate with third-reviewer adjudication; models were independently re-implemented from frozen specifications, and no global treatment ranking was constructed. The 147 secondary binary active–active contrasts were independently rederived and validated row by row from the retained implementation-independent posterior draws, without model refitting or additional MCMC sampling. These safeguards reduced double counting, artificial connectivity, prior-dominated inference, and computational transcription error [13,14,17].

The review was registered retrospectively rather than prospectively. The OSF record documents the reconstructed protocol and methodological audit trail but cannot provide prospective safeguards [12,13,14,15,16]. Original search exports and reviewer-specific screening records were not publicly deposited; however, study selection and report-level eligibility assessment were performed independently by two reviewers, with supervisor adjudication of conflicts or differences in eligibility criteria. The final report-level inclusion/exclusion ledger and report-to-randomization crosswalk were reconstructed and verified from the retained Rayyan export and source reports. Three duplicate bibliographic representations of retained reports were identified; their classification did not alter the retained corpus, quantitative evidence, or conclusions. The Van Adelsberg et al. (2003) trial [18] patient-disposition figure resolved an internal participant-count conflict, affecting only represented-N and node-size metadata. Forty-one of the 240 reports assessed for eligibility were available only as abstracts because no more complete report could be identified; all were excluded, and trial-register sources were handled separately. Limited abstract information may have affected eligibility decisions and the reliability of the corresponding exclusion classifications, although not the characterization of included outcomes.

Only 35 of the 64 retained distinct randomized cohorts contributed model-ready data to a principal evidence structure. Because quantitative completeness may not have been random, selection into the modeled evidence may have influenced its composition and apparent precision. Several structures were small, minimally replicated, predominantly placebo-anchored, or saturated, limiting evaluation of the common heterogeneity assumption. Frequentist prediction intervals were conditional on plug-in REML estimates, and several heterogeneity estimates were not informative. The exact ×0.75 transformation of Okubo T4NSS [20] harmonized the numerical range but cannot establish semantic equivalence between the 0–4 item anchors, which include a “very severe” category, and the canonical 0–3 anchors.

The binary active–active contrasts were secondary derivations from joint posterior draws retained by the implementation-independent replication rather than the original source-primary implementation. Although the corresponding placebo-referenced effects reproduced the source-primary summaries within the predefined numerical tolerances and all 147 secondary contrasts passed independent row-level validation, these contrasts were not multiplicity-adjusted or formally appraised with CINeMA. The three global incoherence tests not re-estimated after the final source-verified freeze remain a separate limitation. Zenodo version 1.0.0 corresponds to submitted version 1.0; the updated version 1.1 reproducibility package supporting the current submission is publicly available at doi:10.5281/zenodo.22086455.

Outcome harmonization required judgments about constructs, components, recall periods, temporal aggregation, endpoints versus change scores, scale transformations, and treatment periods; stricter source-and-scale restrictions materially reduced several structures. Exact outcome-specific analyzed denominators were unavailable for some continuous results. Because SAR, PAR, and PER were treated as reported clinical categories rather than mutually exclusive phenotypes, population overlap may complicate applicability and transitivity. Placebo response, a potentially important effect modifier in placebo-anchored structures, was inconsistently reported.

Harms reporting was incomplete. Somnolence, psychomotor performance, driving safety, serious and treatment-related adverse events, anticholinergic effects, and cardiac safety were not synthesized because definitions and reporting were insufficiently standardized; future studies should use prespecified, consistent definitions and outcome-specific reporting for these domains [7,8,24,25]. Aggregate data also precluded assessment of effect modification by baseline severity, age, sensitization, exposure, comorbidity, or previous response.

Finally, implementation-independent replication and row-level validation support computational fidelity to the frozen analytic data and specifications but cannot address trial conduct, risk of bias, missing evidence, or outcome harmonization.

### 4.7. Implications for Research

Future trials should prioritize clinically relevant active–active comparisons powered for realistic between-regimen differences. Reports should provide exact randomized and analyzed denominators, complete outcome definitions and component scores, prespecified assessment times, covariance information for multi-arm or adjusted analyses, and participant-level counts for all-causality, treatment-related, serious, sedating, and discontinuation-related harms [13,14,17,21,24,25]. Standardizing TNSS and TOSS components, recall periods, averaging rules, and endpoint selection would materially improve comparability [21].

Dedicated comparative syntheses are needed for somnolence, psychomotor performance, driving safety, and cardiac outcomes [7,8,24,25]. Antihistamine–pseudoephedrine combinations should remain analytically separate from monotherapy, with explicit evaluation of congestion-predominant populations and the benefits and harms attributable to the decongestant component [3,4,106,107]. Individual-participant-data meta-analysis could examine effect modification, while a prospectively registered living update with publicly deposited search and screening records could incorporate new trials without losing the present audit trail [12,13,14,15,16,17,108].

## 5. Conclusions

Comparative effects of oral second-generation H1-antihistamine monotherapy varied by rhinitis category, regimen, outcome, and treatment period. Across nine sparse, predominantly placebo-anchored evidence structures, favorable placebo-referenced estimates did not support a reliable active-regimen hierarchy, and the evidence did not establish therapeutic equivalence. Direct active–active evidence was limited, estimates were often imprecise, and all estimable comparisons within the defined CINeMA appraisal scope were rated very low confidence; the secondary binary active–active contrasts were not formally appraised using CINeMA. Some regimens had favorable estimates in more than one outcome domain, but these patterns arose from distinct studies and evidence structures, were not evaluated in a joint benefit–harm model, and remain hypothesis-generating. Somnolence and other functionally important harms were too inconsistently reported for reliable synthesis, and six of the 14 regimen-level nodes contributed only to tolerability structures. Treatment selection should therefore remain outcome-specific and patient-centered; adequately powered, transparently reported head-to-head trials are needed to determine whether clinically meaningful differences exist between active regimens.

## Figures and Tables

**Table 1:** Composition of the nine principal rhinitis-category–outcome evidence structures.

Outcome/ Category	Reports/Units	Arms	Information Count and Type	Represented N	Events	Active Nodes	Structural Status
TNSS–SAR	11/12	26	14 contrasts	6852 *	—	7	Connected; global incoherence estimable (*p* = 0.065)
TNSS–PAR	2/2	6	4 contrasts	1004 *	—	3	Connected; global incoherence estimable (*p* = 0.317)
TOSS–SAR	2/3	7	4 contrasts	1761 *	—	3	Connected; global incoherence non-estimable (0 additional df)
RQLQ–SAR	6/6	13	7 contrasts	3700 *	—	4	Connected; global incoherence estimable (*p* = 0.279); heterogeneity not informatively estimated
RQLQ–PER	2/2	4	2 contrasts	1093 *	—	2	Saturated; heterogeneity and global incoherence non-estimable
Any AE–SAR	9/9	23	14 likelihood dimensions	5648	1351	9	Connected; global incoherence estimable (*p* = 0.984)
Any AE–PAR	8/8	20	12 likelihood dimensions	3239	1080	10	Connected; global test not estimated; CINeMA incoherence domain: major concerns
WDAE–SAR, structural	15/17	38	21 structural contrasts	9149	83	9	Connected; CINeMA incoherence domain: major concerns for seven estimable effects; two active nodes non-estimable/not evaluable
WDAE–SAR, likelihood-informative	—/16	35	19 likelihood dimensions	8249	83	7	Connected likelihood subset; CINeMA incoherence domain: major concerns for seven estimable effects; rupatadine 10/20 mg non-estimable/not evaluable
WDAE–PAR	8/8	22	14 likelihood dimensions	3251	55	10	Connected; global test not estimated; CINeMA incoherence domain: major concerns

**Note:** Randomized-unit counts are unique within, but not across, structures. The 35 modeled units generated 67 randomization–structure contributions; neither these counts nor participant denominators should be summed across structures. Information counts denote model-ready contrasts for continuous outcomes, likelihood dimensions for binary models, or structural contrasts retained in the geometry despite contributing no relative-effect information. For continuous outcomes (*), represented N is the accumulated arm-level denominator used to characterize geometry and may differ from the exact analyzed sample; for binary likelihood-informative rows, it entered the arm-level likelihood. Structural totals may additionally include non-informative all-zero designs. Saturated denotes zero residual degrees of freedom under the consistency model. Degrees of freedom for the design-by-treatment interaction test were assessed separately: TOSS–SAR retained one residual degree of freedom for descriptive heterogeneity estimation but none for global incoherence, whereas RQLQ–PER was saturated for both. Global incoherence estimable indicates that the design-by-treatment test was identifiable, although power remained limited in sparse structures. WDAE–SAR is presented in structural and likelihood-informative rows because one three-arm unit with zero withdrawals in every arm (*n* = 900) contributed to the geometry but not to relative-effect estimation. Its exclusion left 16 units, 35 arms, *N* = 8249, and seven estimable active nodes; rupatadine 10 mg and 20 mg remained structurally present but non-estimable. Reports cannot be separately counted for this subset because report-to-unit mapping is not one-to-one.

## Data Availability

The retrospectively registered protocol and methodological audit trail are publicly available in OSF Registries (doi:10.17605/OSF.IO/DPT5N). Zenodo version 1.0.0 (doi:10.5281/zenodo.21780525) corresponds to the submitted version 1.0 and the frozen primary analytical release v56.0. The updated version 1.1 reproducibility package supporting the current submission is publicly available on Zenodo (doi:10.5281/zenodo.22086455). The source-primary implementation retained marginal placebo-referenced posterior summaries but not joint posterior draws. The archived implementation-independent CmdStanR replication of the four source-primary binary scenarios retained the native chain CSV files, treatment maps, fit metadata, model inputs, Stan specification, and joint posterior draws used for the secondary active–active derivations. The version 1.1 repository package contains the derivation code, all 147 secondary active–active contrast estimates, the 36-effect placebo-referenced concordance audit, source-file manifests, and the independent validation script, row-level output, and completed validation record. L.R.R. is the designated validator in the Author Contributions. Original search exports, the duplicate-cluster log, and reviewer-specific screening timestamps were not retained. Copyrighted source reports are not redistributed.

## Supplementary Materials

The following supporting information can be downloaded at: https://www.mdpi.com/article/10.3390/jcm15187169/s1.

## Abbreviations

The following abbreviations are used in this manuscript:

AE	Adverse event
ARIA	Allergic Rhinitis and its Impact on Asthma
BID	*Bis in die*; twice daily
CI	Confidence interval
CINeMA	Confidence in Network Meta-Analysis
COPD	Chronic obstructive pulmonary disease
CR	Controlled release
CrI	Credible interval
df	Degree of freedom
EAACI	European Academy of Allergy and Clinical Immunology
GLS	Generalized least squares
GRADE-NMA	Grading of Recommendations Assessment, Development and Evaluation approach for network meta-analysis
H1	Histamine H1 receptor
I^2^	Percentage of total variation in observed effect estimates attributable to between-study heterogeneity rather than sampling error
MD	Mean difference
NMA	Network meta-analysis
OD	Once daily
OR	Odds ratio
OSF	Open Science Framework
PAR	Perennial allergic rhinitis
PER	Persistent allergic rhinitis
PrI	Prediction interval
PRISMA	Preferred Reporting Items for Systematic Reviews and Meta-Analyses
PRISMA-NMA	Preferred Reporting Items for Systematic Reviews and Meta-Analyses extension for network meta-analyses
PRISMA-S	Preferred Reporting Items for Systematic Reviews and Meta-Analyses extension for reporting literature searches in systematic reviews
REML	Restricted maximum likelihood
ROB-MEN	Risk of Bias due to Missing Evidence in Network Meta-Analysis
RoB 2	Revised Cochrane risk-of-bias tool for randomized trials
RQLQ	Rhinoconjunctivitis Quality of Life Questionnaire
SAR	Seasonal allergic rhinitis
TNSS	Total nasal symptom score
TOSS	Total ocular symptom score
WDAE	Withdrawal due to adverse events
