# Supplementary material for: Oral Second-Generation H1-Antihistamine Regimens for Allergic Rhinitis: Systematic Review and Network Meta-Analysis of Effects and Estimability
Source: J Clin Med. 2026 Sep 15;15(18):7169. doi: 10.3390/jcm15187169 (PMC13607989; doi:10.3390/jcm15187169)
Supplement: Supplementary file 1 [file jcm-15-07169-s001.zip › jcm-4519754_Supplementary_Materials_v1.1_for_2nd_proofreading.pdf]

Supplementary Materials

Oral Second-Generation H1-Antihistamine Regimens for Allergic Rhinitis: Systematic Review and Network Meta-Analysis of Effects and Estimability

Submission version 1.1

These Supplementary Materials provide the supplementary methods, complete numerical results, sensitivity analyses, evidence appraisals, and computational-validation details cited in the main manuscript.

Outcome definitions, category-specific estimands, regimen nodes, and treatment periods are identical to those in the main manuscript. Submitted version 1.0 used the frozen primary analytical release v56.0. Submission version 1.1 supersedes the version 1.0 file set for editorial processing while retaining v56.0 as the frozen primary analytical release.

Version element	Status
Submitted file set	Version 1.0 - replaced by version 1.1
Frozen primary analytical release	v56.0 - primary fitted models and placebo-referenced estimates
Current file set	Version 1.1 - current complete submission
Public repository	Zenodo v1.1; doi:10.5281/zenodo.22086455. Zenodo v1.0.0 (doi:10.5281/zenodo.21780525) corresponds to submission version 1.0.
Secondary binary contrasts	147 exact within-draw derivations from retained implementation-independent posterior draws; no refit or new MCMC

Contents

Section or item	Page
S1. Review conduct, protocol status, and analytical governance	5
S2. Information sources, platforms, search dates, record counts, and complete search strategies	6
S3. Eligibility criteria	14
S4. Regimen-level treatment-node dictionary	15
S5. Source hierarchy, extraction, and numerical reconstruction	16
S6. Outcomes, estimands, treatment periods, and deterministic result selection	17
S7. Somnolence, sedation, and other unsynthesized harms	18
S8. Network geometry, structural estimability, and active-active audit	19
S9. Transitivity, indirectness, and incoherence support	21
S10. Sensitivity and exploratory analysis map	23
S11. Computation, diagnostics, calibration, and replication	24
S12. Full-corpus crosswalk, eligibility disposition, and denominator reconciliation	25
S13. Characteristics of model-ready quantitative evidence	42
S14. Standardized absolute-risk scenarios for binary outcomes	45
S15. Primary continuous comparative effects	50
S16. Primary binary effects and prediction intervals	51
S17. Binary heterogeneity and diagnostic status	53
S18. Continuous sensitivity and exploratory effect estimates	54
S19. WDAE-SAR source and prior sensitivities	55
S20. Rupatadine Any AE-SAR null-boundary audit	56
S21. RoB 2, CINeMA, and ROB-MEN assessments	57
S22. Contribution-matrix arithmetic and graphical quality assurance	58
S23. Implementation-independent computational replication	60
S24. Supplementary figure inventory and generation provenance	61
S25. Reproducibility package, availability, and interpretive scope	62
S26. Complete active-active comparative effects	63
S27. Comparison-level CINeMA and ROB-MEN appraisals	65
S28. Model inputs and individual-study/arm reporting	72
S29. Characteristics of model-ready studies and participants	79
S30. Result-specific RoB 2 domain assessments	82

Section or item	Page
S31. Binary active-active secondary contrasts	88
S32. Binary effect cross-table consistency audit	89
S33. Continuous heterogeneity and generalized Cochran Q diagnostics	91
S34. Submission version and repository status	92
Supplementary abbreviations	93
Supplementary Figures S1-S32	94-125

Main-manuscript crosswalk

Crosswalk between manuscript version 1.1 and the Supplementary Materials.

Main location	Supplement item	Content
Methods 2.1	Tables S1 and S34	Retrospective registration, final analytical specifications, and version 1.0-to-1.1 file-set relationship
Methods 2.2	Table S2	Sources, search coverage, dates, complete strategies, deduplication, and qualifications
Methods 2.3	Tables S3-S4	Hierarchical retention, synthesis eligibility, partial-arm retention, and node dictionary
Methods 2.4	Table S5	Source hierarchy, duplicate extraction, reconstruction rules, and provenance
Methods 2.5	Tables S6-S7 and S28	Outcomes, estimands, periods, result selection, unsynthesized harms, and inputs
Methods 2.6 / Results 3.2	Tables S8A-S9; Figures S8-S16	Geometry, structural estimability, transitivity, effect modifiers, and incoherence
Results 3.6	Tables S26-S27/S31-S33	Continuous and binary active-active effects, appraisals, and heterogeneity
Methods 2.9 / Results 3.7	Tables S10/S18-S20	Sensitivity and exploratory analyses, priors, source/event analyses, and boundary audit
Methods 2.10 / Results 3.9	Tables S11/S23; Figure S32	Computation, diagnostics, calibration, and implementation-independent replication
Results 3.1	Tables S12-S13	Selection, corpus mapping, analytical disposition, characteristics, and denominators
Results 3.3-3.5	Tables S14-S20/S28; Figures S2-S7	Continuous and binary effects, absolute risks, prediction intervals, and inputs
Results 3.8	Tables S21/S27/S30/S32-S33	RoB 2, CINeMA, ROB-MEN, domain distributions, binary display QA, and continuous heterogeneity
Data Availability	Tables S22-S34	Contribution matrices, figures, current files, results, appraisals, characteristics, reproducibility, aliases, and version status

Version 1.1 replaces submitted version 1.0. Primary fitted models remain those of analytical release v56.0; the 147 secondary binary contrasts use retained implementation-independent posterior draws.

S1. Review Conduct, Protocol Status, and Analytical Governance

Table S1. Review conduct, protocol status, and analytical governance.

Domain	Final status	Interpretation
Registration	Retrospective OSF registration on 4 August 2026	Study selection, extraction, appraisals, principal analyses, independent replication, and figures preceded registration; no prospective safeguard is claimed.
Search	Seven sources; records available through 30 June 2026	Exact strategies and dates are reported in S2.
Study selection	Independent dual review with supervisor adjudication	Final report-level decisions are retained in the screening ledger.
Quantitative extraction	Independent duplicate extraction	Source hierarchy, denominators, derivations, and verification status are documented.
Evidence unit	Independent randomization	61 reports map to 66 report-randomization entries and 64 non-overlapping cohorts; 35 were model-ready.
Primary analytical source	Frozen release v56.0	Primary fitted models and placebo-referenced treatment effects remain unchanged in version 1.1.
Current submission	Version 1.1	Complete replacement for submitted version 1.0.

Specification	Final implementation	Qualification
Ratner WDAE risk period	Randomized-treatment risk period	Avoids mixing randomized treatment with run-out follow-up.
Bepotastine nodes	10 mg twice daily and CR 20 mg once daily treated separately	Regimen-specific node definition.
Okubo T4NSS	Raw 0-16 values multiplied by 0.75 for the 0-12 analysis scale	Exact linear range transformation; semantic equivalence is not assumed.
Zero-event studies	Retained descriptively but contribute no relative-effect information	No continuity correction; unsupported effects remain non-estimable.
Binary active-active contrasts	147 exact within-draw differences from retained implementation-independent posterior draws	No model refit or new MCMC sampling; no multiplicity adjustment; not formally appraised with CINeMA.
Human validation	L.R.R. is the designated validator	Completed by L.R.R.: 147/147 PASS; max difference $5.0000049 \times 10^{-10}$; full conformity and approval recorded.
Incoherence	Three global tests remain not re-estimated after the final source-verified freeze	Reported as a limitation; absence of a test is not evidence of consistency.
Version relationship	Submitted version 1.0 / analytical release v56.0 -> current version 1.1	Replace all version 1.0 files with the version 1.1 file set.

S2. Information sources, platforms, search dates, record counts, and complete search strategies

All information sources were searched separately from inception to the prespecified review coverage cutoff of 30 June 2026. PubMed and Embase were searched on 1 July 2026; Web of Science Core Collection and CENTRAL on 2 July 2026; and WHO ICTRP, ClinicalTrials.gov, and the EU Clinical Trials Register on 3 July 2026. No language restrictions were applied, and no subsequent search update, rerun, or alert was conducted.

Table S2. Information sources and records identified before deduplication.

Information source	Source type	Platform/interface and provider	Date last searched	Review coverage cutoff	Records identified before deduplication
PubMed	Bibliographic database	PubMed public interface; U.S. National Library of Medicine	1 July 2026	30 June 2026	1,759
Embase	Bibliographic database	Embase.com; Elsevier	1 July 2026	30 June 2026	3,941
Web of Science Core Collection	Bibliographic database	Web of Science platform; Clarivate; institutional access through FECYT	2 July 2026	30 June 2026	3,114
Cochrane Central Register of Controlled Trials (CENTRAL)	Bibliographic database	Cochrane Library Search Manager; Cochrane	2 July 2026	30 June 2026	3,126
WHO International Clinical Trials Registry Platform (ICTRP)	Trial register	WHO ICTRP Trial Search Portal	3 July 2026	30 June 2026	36
ClinicalTrials.gov	Trial register	Public web interface; U.S. National Library of Medicine	3 July 2026	30 June 2026	31
European Union Clinical Trials Register (EU CTR)	Trial register	Public web interface; European Medicines Agency	3 July 2026	30 June 2026	176
Bibliographic-database subtotal					11,940
Trial-register subtotal					243
Total					12,183

Note: Counts are records identified before deduplication. The bibliographic-database subtotal of 11,940 and the trial-register subtotal of 243 yielded 12,183 records overall. After removal of 6,261 duplicate records, 5,922 records remained for title-and-abstract screening. No records were removed by automation tools or for other reasons before screening. The strategies were developed specifically for this review; no adaptation from a previously published review strategy was reported. They combined controlled vocabulary, where available, with free-text terms for allergic rhinitis or rhinoconjunctivitis, oral H1-antihistamines and individual agents, and randomized or controlled trials. No published or externally validated methodological search filter was used, and no automated tool was used to translate strategies between sources. Indexing terms, field codes, Boolean operators, and proximity syntax were adapted to each platform. PRESS peer review was not performed. All records originated from the seven sources listed above. No records were supplied by the authors or identified through a separate other-methods route. Reference lists of eligible reports and relevant systematic reviews were checked only to verify report linkage, secondary-publication relationships, and trial-family completeness; this process added no records to the documented database/register flow. No forward-citation search, citation-alert service, study-investigator contact, expert contact, manufacturer contact, or search for unpublished data outside the listed trial registers was undertaken. Deduplication and screening management were performed in Rayyan.ai web. Records were considered duplicates when they shared an identical DOI or, in the absence of a reliable DOI match, when similarity in title or author list - corroborated by equivalent or closely similar available record content - indicated the same bibliographic report rather than distinct reports from the same independent randomization. The record-level duplicate-cluster log was not retained. Rayyan.ai web filters were used only to organize records for manual assessment; no automation tool made eligibility decisions. The complete strategies below are reproduced as executed. Only typographical line wrapping and formatting have been altered; no search terms, field tags, operators, limits, or logical relationships have been changed. The following syntax reproduces the platform-specific search strategies. Formatting changes are typographical only and do not alter the search logic.

Table S2 (continued). PubMed search strategy.

PubMed via the PubMed public interface, U.S. National Library of Medicine; searched 1 July 2026; review coverage cutoff 30 June 2026; records identified before deduplication, n = 1,759.

("Rhinitis, Allergic"[Mesh] OR "Rhinitis, Allergic, Seasonal"[Mesh] OR "Rhinitis, Allergic, Perennial"[Mesh] OR "allergic rhinitis"[tiab] OR "seasonal allergic rhinitis"[tiab] OR "perennial allergic rhinitis"[tiab] OR "allergic rhinoconjunctivitis"[tiab] OR rhinoconjunctivitis[tiab] OR pollinosis[tiab] OR "hay fever"[tiab] OR hayfever[tiab] OR "pollen allergy"[tiab] OR "pollen-induced"[tiab] OR ragweed[tiab] OR "grass pollen"[tiab] OR "Japanese cedar pollinosis"[tiab]) AND ("Histamine H1 Antagonists"[Mesh] OR antihistamin*[tiab] OR "H1 antihistamin*"[tiab] OR "H1-antihistamin*"[tiab] OR "H1 antagonist*"[tiab] OR "H1 receptor antagonist*"[tiab] OR "oral antihistamin*"[tiab] OR "oral H1"[tiab] OR OAH[tiab] OR cetirizine[tiab] OR cetrizine[tiab] OR levocetirizine[tiab] OR loratadine[tiab] OR desloratadine[tiab] OR fexofenadine[tiab] OR bilastine[tiab] OR rupatadine[tiab] OR ebastine[tiab] OR terfenadine[tiab] OR astemizole[tiab] OR mizolastine[tiab] OR mequitazine[tiab] OR acrivastine[tiab] OR bepotastine[tiab] OR ketotifen[tiab] OR olopatadine[tiab] OR oxatomide[tiab] OR epinastine[tiab] OR azelastine[tiab] OR levocabastine[tiab] OR chlorpheniramine[tiab] OR chlorphenamine[tiab] OR dexchlorpheniramine[tiab] OR dexchlorphenamine[tiab] OR brompheniramine[tiab] OR bromphenamine[tiab] OR dexbrompheniramine[tiab] OR pheniramine[tiab] OR triprolidine[tiab] OR hydroxyzine[tiab] OR meclizine[tiab] OR meclozine[tiab] OR clemastine[tiab] OR carbinoxamine[tiab] OR diphenhydramine[tiab] OR dimenhydrinate[tiab] OR doxylamine[tiab] OR promethazine[tiab] OR alimemazine[tiab] OR trimeprazine[tiab] OR cyproheptadine[tiab] OR antazoline[tiab] OR quifenadine[tiab] OR dimethindene[tiab] OR dimetindene[tiab] OR oxomemazine[tiab]) AND (randomized controlled trial[pt] OR controlled clinical trial[pt] OR random*[tiab] OR randomly[tiab] OR trial[tiab] OR placebo[tiab] OR "placebo-controlled"[tiab] OR "double blind"[tiab] OR double-blind[tiab] OR "single blind"[tiab] OR single-blind[tiab] OR "parallel group"[tiab] OR parallel-group[tiab] OR multicenter[tiab] OR multicentre[tiab] OR "clinical trial"[tiab]) NOT (animals[mh] NOT humans[mh])

Table S2 (continued). Embase search strategy.

Embase via Embase.com, Elsevier; searched 1 July 2026; review coverage cutoff 30 June 2026; records identified before deduplication, n = 3,941.

(('allergic rhinitis'/exp OR 'seasonal allergic rhinitis'/exp OR 'perennial allergic rhinitis'/exp OR 'hay fever'/exp OR 'rhinoconjunctivitis'/exp OR 'allergic rhinitis':ti,ab,kw OR 'seasonal allergic rhinitis':ti,ab,kw OR 'perennial allergic rhinitis':ti,ab,kw OR 'allergic rhinoconjunctivitis':ti,ab,kw OR rhinoconjunctivitis:ti,ab,kw OR pollinosis:ti,ab,kw OR 'hay fever':ti,ab,kw OR hayfever:ti,ab,kw OR 'pollen allergy':ti,ab,kw OR 'pollen-induced':ti,ab,kw OR ragweed:ti,ab,kw OR 'grass pollen':ti,ab,kw OR 'Japanese cedar pollinosis':ti,ab,kw) AND ('histamine h1 receptor antagonist'/exp OR antihistamin*:ti,ab,kw OR 'h1 antihistamin*':ti,ab,kw OR 'h1-antihistamin*':ti,ab,kw OR 'h1 antagonist*':ti,ab,kw OR 'h1 receptor antagonist*':ti,ab,kw OR 'oral antihistamin*':ti,ab,kw OR oah:ti,ab,kw OR 'cetirizine'/exp OR cetirizine:ti,ab,kw OR cetrizine:ti,ab,kw OR 'levocetirizine'/exp OR levocetirizine:ti,ab,kw OR 'loratadine'/exp OR loratadine:ti,ab,kw OR 'desloratadine'/exp OR desloratadine:ti,ab,kw OR 'fexofenadine'/exp OR fexofenadine:ti,ab,kw OR 'bilastine'/exp OR bilastine:ti,ab,kw OR 'rupatadine'/exp OR rupatadine:ti,ab,kw OR 'ebastine'/exp OR ebastine:ti,ab,kw OR 'terfenadine'/exp OR terfenadine:ti,ab,kw OR 'astemizole'/exp OR astemizole:ti,ab,kw OR 'mizolastine'/exp OR mizolastine:ti,ab,kw OR 'mequitazine'/exp OR mequitazine:ti,ab,kw OR 'acrivastine'/exp OR acrivastine:ti,ab,kw OR 'bepotastine'/exp OR bepotastine:ti,ab,kw OR 'ketotifen'/exp OR ketotifen:ti,ab,kw OR 'olopatadine'/exp OR olopatadine:ti,ab,kw OR 'oxatomide'/exp OR oxatomide:ti,ab,kw OR 'epinastine'/exp OR epinastine:ti,ab,kw OR 'azelastine'/exp OR azelastine:ti,ab,kw OR 'levocabastine'/exp OR levocabastine:ti,ab,kw OR 'chlorphenamine'/exp OR chlorphenamine:ti,ab,kw OR chlorpheniramine:ti,ab,kw OR 'dexchlorpheniramine'/exp OR dexchlorpheniramine:ti,ab,kw OR dexchlorphenamine:ti,ab,kw OR 'brompheniramine'/exp OR brompheniramine:ti,ab,kw OR bromphenamine:ti,ab,kw OR dexbrompheniramine:ti,ab,kw OR 'pheniramine'/exp OR pheniramine:ti,ab,kw OR 'triprolidine'/exp OR triprolidine:ti,ab,kw OR 'hydroxyzine'/exp OR hydroxyzine:ti,ab,kw OR 'meclizine'/exp OR meclizine:ti,ab,kw OR meclozine:ti,ab,kw OR 'clemastine'/exp OR clemastine:ti,ab,kw OR 'carbinoxamine'/exp OR carbinoxamine:ti,ab,kw OR 'diphenhydramine'/exp OR diphenhydramine:ti,ab,kw OR 'dimenhydrinate'/exp OR dimenhydrinate:ti,ab,kw OR 'doxylamine'/exp OR doxylamine:ti,ab,kw OR 'promethazine'/exp OR promethazine:ti,ab,kw OR 'alimemazine'/exp OR alimemazine:ti,ab,kw OR trimeprazine:ti,ab,kw OR 'cyproheptadine'/exp OR cyproheptadine:ti,ab,kw OR 'antazoline'/exp OR antazoline:ti,ab,kw OR quifenadine:ti,ab,kw OR dimethindene:ti,ab,kw OR dimetindene:ti,ab,kw OR oxomemazine:ti,ab,kw OR pyrilamine:ti,ab,kw OR methapyrilene:ti,ab,kw OR tripelennamine:ti,ab,kw) AND ('randomized controlled trial'/exp OR 'controlled clinical trial'/exp OR 'randomization'/exp OR 'double blind procedure'/exp OR 'single blind procedure'/exp OR 'placebo'/exp OR random*:ti,ab,kw OR randomly:ti,ab,kw OR trial:ti,ab,kw OR placebo:ti,ab,kw OR 'placebo-controlled':ti,ab,kw OR 'double blind':ti,ab,kw OR 'double-blind':ti,ab,kw OR 'single blind':ti,ab,kw OR 'single-blind':ti,ab,kw OR 'parallel group':ti,ab,kw OR 'parallel-group':ti,ab,kw OR multicenter:ti,ab,kw OR multicentre:ti,ab,kw OR 'clinical trial':ti,ab,kw)) NOT ('animal'/exp NOT 'human'/exp)

Table S2 (continued). Web of Science Core Collection search strategy.

Web of Science Core Collection via the Web of Science platform, Clarivate, accessed through FECYT; searched 2 July 2026; review coverage cutoff 30 June 2026; records identified before deduplication, n = 3,114.

TS=(("allergic rhinitis" OR "seasonal allergic rhinitis" OR "perennial allergic rhinitis" OR "allergic rhinoconjunctivitis" OR rhinoconjunctiviti* OR pollinosis OR "hay fever" OR hayfever OR "pollen allergy" OR "pollen-induced" OR ragweed OR "grass pollen" OR "Japanese cedar pollinosis") AND (antihistamin* OR "oral antihistamin*" OR (H1 NEAR/2 antihistamin*) OR (H1 NEAR/2 antagonist*) OR "histamine H1 antagonist*" OR "histamine H1 receptor antagonist*" OR cetirizine OR cetrizine OR levocetirizine OR loratadine OR desloratadine OR fexofenadine OR bilastine OR rupatadine OR ebastine OR terfenadine OR astemizole OR mizolastine OR mequitazine OR acrivastine OR bepotastine OR ketotifen OR olopatadine OR oxatomide OR epinastine OR azelastine OR levocabastine OR chlorpheniramine OR chlorphenamine OR dexchlorpheniramine OR dexchlorphenamine OR brompheniramine OR bromphenamine OR dexbrompheniramine OR pheniramine OR triprolidine OR hydroxyzine OR meclizine OR meclozine OR clemastine OR carbinoxamine OR diphenhydramine OR dimenhydrinate OR doxylamine OR promethazine OR alimemazine OR trimeprazine OR cyproheptadine OR antazoline OR quifenadine OR dimethindene OR dimetindene OR oxomemazine OR pyrilamine OR methapyrilene OR tripelennamine) AND (random* OR randomly OR trial* OR placebo* OR "placebo controlled" OR placebo-controlled OR "controlled trial" OR "clinical trial" OR "randomized controlled trial" OR "randomised controlled trial" OR "double blind" OR double-blind OR "single blind" OR single-blind OR "parallel group" OR parallel-group OR multicenter OR multicentre))

Table S2 (continued). CENTRAL search strategy.

Cochrane Central Register of Controlled Trials via the Cochrane Library Search Manager; searched 2 July 2026; review coverage cutoff 30 June 2026; records identified before deduplication, n = 3,126.

(([mh "Rhinitis, Allergic"] OR [mh "Rhinitis, Allergic, Seasonal"] OR [mh "Rhinitis, Allergic, Perennial"] OR [mh "Conjunctivitis, Allergic"] OR "allergic rhinitis":ti,ab,kw OR "seasonal allergic rhinitis":ti,ab,kw OR "perennial allergic rhinitis":ti,ab,kw OR "allergic rhinoconjunctivitis":ti,ab,kw OR rhinoconjunctiviti*:ti,ab,kw OR pollinosis:ti,ab,kw OR "hay fever":ti,ab,kw OR hayfever:ti,ab,kw OR "pollen allergy":ti,ab,kw OR "pollen-induced":ti,ab,kw OR ragweed:ti,ab,kw OR "grass pollen":ti,ab,kw OR "Japanese cedar pollinosis":ti,ab,kw) AND ([mh "Histamine H1 Antagonists"] OR antihistamin*:ti,ab,kw OR "oral antihistamin*":ti,ab,kw OR "H1 antihistamin*":ti,ab,kw OR "H1-antihistamin*":ti,ab,kw OR "H1 antagonist*":ti,ab,kw OR "H1 receptor antagonist*":ti,ab,kw OR "histamine H1 antagonist*":ti,ab,kw OR "histamine H1 receptor antagonist*":ti,ab,kw OR cetirizine:ti,ab,kw OR cetrizine:ti,ab,kw OR levocetirizine:ti,ab,kw OR loratadine:ti,ab,kw OR desloratadine:ti,ab,kw OR fexofenadine:ti,ab,kw OR bilastine:ti,ab,kw OR rupatadine:ti,ab,kw OR ebastine:ti,ab,kw OR terfenadine:ti,ab,kw OR astemizole:ti,ab,kw OR mizolastine:ti,ab,kw OR mequitazine:ti,ab,kw OR acrivastine:ti,ab,kw OR bepotastine:ti,ab,kw OR ketotifen:ti,ab,kw OR olopatadine:ti,ab,kw OR oxatomide:ti,ab,kw OR epinastine:ti,ab,kw OR azelastine:ti,ab,kw OR levocabastine:ti,ab,kw OR chlorpheniramine:ti,ab,kw OR chlorphenamine:ti,ab,kw OR dexchlorpheniramine:ti,ab,kw OR dexchlorphenamine:ti,ab,kw OR brompheniramine:ti,ab,kw OR bromphenamine:ti,ab,kw OR dexbrompheniramine:ti,ab,kw OR pheniramine:ti,ab,kw OR triprolidine:ti,ab,kw OR hydroxyzine:ti,ab,kw OR meclizine:ti,ab,kw OR meclozine:ti,ab,kw OR clemastine:ti,ab,kw OR carbinoxamine:ti,ab,kw OR diphenhydramine:ti,ab,kw OR dimenhydrinate:ti,ab,kw OR doxylamine:ti,ab,kw OR promethazine:ti,ab,kw OR alimemazine:ti,ab,kw OR trimeprazine:ti,ab,kw OR cyproheptadine:ti,ab,kw OR antazoline:ti,ab,kw OR quifenadine:ti,ab,kw OR dimethindene:ti,ab,kw OR dimetindene:ti,ab,kw OR oxomemazine:ti,ab,kw OR pyrilamine:ti,ab,kw OR methapyrilene:ti,ab,kw OR tripelennamine:ti,ab,kw))

Table S2 (continued). WHO ICTRP search strategy.

WHO International Clinical Trials Registry Platform via the Trial Search Portal; searched 3 July 2026; review coverage cutoff 30 June 2026; records identified before deduplication, n = 36.

Condition: "allergic rhinitis" OR "seasonal allergic rhinitis" OR "perennial allergic rhinitis" OR rhinoconjunctivitis OR "allergic rhinoconjunctivitis" OR pollinosis OR "hay fever" OR hayfever OR "pollen allergy" OR ragweed OR "grass pollen" OR "Japanese cedar pollinosis"

Intervention: antihistamine OR antihistamines OR "oral antihistamine" OR "oral antihistamines" OR "H1 antihistamine" OR "H1 antihistamines" OR "H1 antagonist" OR "H1 antagonists" OR "H1 receptor antagonist" OR "H1 receptor antagonists" OR "histamine H1 antagonist" OR "histamine H1 receptor antagonist"

Table S2 (continued). ClinicalTrials.gov search strategy.

ClinicalTrials.gov via its public web interface, U.S. National Library of Medicine; searched 3 July 2026; review coverage cutoff 30 June 2026; records identified before deduplication, n = 31.

Condition: "allergic rhinitis" OR "seasonal allergic rhinitis" OR "perennial allergic rhinitis" OR rhinoconjunctivitis OR "allergic rhinoconjunctivitis" OR pollinosis OR "hay fever" OR hayfever OR "pollen allergy" OR ragweed OR "grass pollen" OR "Japanese cedar pollinosis"

Intervention: antihistamine OR antihistamines OR "oral antihistamine" OR "oral antihistamines" OR "H1 antihistamine" OR "H1 antihistamines" OR "H1 antagonist" OR "H1 antagonists" OR "H1 receptor antagonist" OR "H1 receptor antagonists" OR "histamine H1 antagonist" OR "histamine H1 receptor antagonist" Other: cetirizine OR levocetirizine OR loratadine OR desloratadine OR fexofenadine OR bilastine OR rupatadine OR ebastine OR acrivastine OR bepotastine OR ketotifen OR olopatadine OR epinastine

Table S2 (continued). EU Clinical Trials Register search strategy.

European Union Clinical Trials Register via its public web interface, European Medicines Agency; searched 3 July 2026; review coverage cutoff 30 June 2026; records identified before deduplication, n = 176.

("allergic rhinitis" OR "seasonal allergic rhinitis" OR "perennial allergic rhinitis" OR rhinoconjunctivitis OR "allergic rhinoconjunctivitis" OR pollinosis OR "hay fever" OR hayfever OR "pollen allergy" OR ragweed OR "grass pollen" OR "Japanese cedar pollinosis") AND (antihistamine OR antihistamines OR "oral antihistamine" OR "oral antihistamines" OR "H1 antihistamine" OR "H1 antihistamines" OR "H1 antagonist" OR "H1 antagonists" OR "H1 receptor antagonist" OR "H1 receptor antagonists" OR "histamine H1 antagonist" OR "histamine H1 receptor antagonist")

S3. Eligibility criteria

Table S3. Eligibility criteria.

Domain	Criterion
Population	Participants with clinically diagnosed SAR, PAR, or PER under natural allergen exposure. Mixed-age populations were eligible when otherwise compatible; pediatric-only populations were excluded.
Review-corpus retention	Randomized evidence relevant to an eligible second-generation oral H1-antihistamine regimen, trial family, evidence map, or explicitly labeled contextual/sensitivity layer could be retained even when no contrast met all principal-synthesis requirements. Retention did not imply contribution to a principal NMA.
Principal interventions	Eligible oral second-generation H1-antihistamine monotherapy at a documented molecule, dose, frequency, route, and node-defining formulation.
Principal comparators	Placebo or another eligible oral second-generation H1-antihistamine regimen.
Design and study unit	Randomized parallel-group trials. The independent randomization, not the publication, was the study unit.
Partial-arm retention	Eligible antihistamine and placebo/comparator arms from mixed-class or partially eligible trials were retained only when randomization among retained arms remained valid; ineligible arms were omitted and the full trial context informed transitivity.
Primary continuous outcomes	Canonical four-item TNSS, three-item TOSS, and total RQLQ, with compatible components, direction, respondent, aggregation, timing, and summary statistic.
Primary binary outcomes	Participants with at least one all-causality adverse event and participants withdrawing because of an adverse event during randomized treatment.
Principal-synthesis exclusions	Pediatric-only; non-randomized or uncontrolled; crossover without an eligible parallel comparison; allergen-chamber or experimental-provocation; topical-only or noneligible regimens; inseparable populations or interventions; or incompatible constructs, scales, periods, risk windows, analysis populations, or uncertainty/event data.
Language and report status	No language restriction. Full articles, abstracts, and trial-register records could inform eligibility; quantitative synthesis required sufficient source-verified model-ready data.

S4. Regimen-level treatment-node dictionary

Table S4. Regimen-level treatment-node dictionary.

Node	Molecule	Dose	Frequency	Formulation/route	Structures represented
Bepotastine 10 mg BID	Bepotastine	10 mg	Twice daily	Immediate-release oral	Tolerability only
Bepotastine CR 20 mg OD	Bepotastine	20 mg	Once daily	Controlled-release oral	Tolerability only
Bilastine 20 mg OD	Bilastine	20 mg	Once daily	Oral	Efficacy, quality of life, tolerability
Cetirizine 10 mg OD	Cetirizine	10 mg	Once daily	Oral	Efficacy, quality of life, tolerability
Desloratadine 5 mg OD	Desloratadine	5 mg	Once daily	Oral	Efficacy, quality of life, tolerability
Ebastine 10 mg OD	Ebastine	10 mg	Once daily	Oral	Tolerability only
Ebastine 20 mg OD	Ebastine	20 mg	Once daily	Oral	Tolerability only
Fexofenadine 120 mg OD	Fexofenadine	120 mg	Once daily	Oral	Tolerability only
Fexofenadine 180 mg OD	Fexofenadine	180 mg	Once daily	Oral	Efficacy and tolerability
Levocetirizine 5 mg OD	Levocetirizine	5 mg	Once daily	Oral	Quality of life and tolerability
Loratadine 10 mg OD	Loratadine	10 mg	Once daily	Oral	Efficacy, quality of life, tolerability
Mizolastine 10 mg OD	Mizolastine	10 mg	Once daily	Oral	Tolerability only
Rupatadine 10 mg OD	Rupatadine	10 mg	Once daily	Oral	Efficacy and tolerability; non-estimable in WDAE-SAR
Rupatadine 20 mg OD	Rupatadine	20 mg	Once daily	Oral	Efficacy and tolerability; non-estimable in WDAE-SAR

Note: Nodes were not merged solely because they shared a molecule. Eligibility was operational at the protocol reference date and does not imply contemporaneous authorization in every jurisdiction. OD = once daily; BID = twice daily; CR = controlled-release; legacy machine-readable node identifiers may retain SR for version continuity.

S5. Source hierarchy, extraction, and numerical reconstruction

Table S5. Documented hierarchy of quantitative sources.

Priority	Source type	Rule
1	Valid adjusted randomized between-group contrast	Preferred when it directly matched the target estimand.
2	Compatible arm-level change scores	Used when between-group contrast and uncertainty could be derived without unsupported assumptions.
3	Derivable change scores	Accepted only with sufficient source information.
4	Compatible final-value estimates	Used when change-score information was unavailable and the target estimand remained compatible.
5	Independently verified numerical reconstruction	Permitted only under deterministic, source-auditable rules.

Table S5 (continued). Data preparation and reconstruction rules.

Issue	Operational rule
Missing continuous data	Not imputed. Safety-population denominators were not substituted into efficacy variances.
Missing binary data	Participants with missing or unreported outcomes were not assumed event-free.
Percentages to counts	Converted only when the percentage, denominator, and rounding convention yielded one mathematically possible integer.
Conflicting values	Preserved in separate primary and sensitivity layers rather than silently overwritten.
Multi-arm covariance	Shared-arm sampling covariance derived from the relevant arm variance; one-half correlation used only for the multi-arm random-effects component.
All-zero/all-one studies	Retained in descriptive totals but excluded from the relative-effect likelihood because they contained no relative-odds information.

S6. Outcomes, estimands, treatment periods, and deterministic result selection

Table S6. Target outcomes, estimands, periods, and deterministic result-selection rules.

Structure	Construct	Estimand/direction	Primary period	Selection rule
TNSS-SAR	Four-item TNSS, 0-12	MD; negative favors active	Days 1-14	Treatment-period average preferred; otherwise compatible adjusted endpoint nearest day 14
TNSS-PAR	Compatible TNSS	MD; negative favors active	Days 1-28	Average preferred; otherwise nearest compatible endpoint
TOSS-SAR	Three-item ocular score, 0-9	MD; negative favors active	Days 1-14	Source-verified ocular-only construct: tearing, ocular redness, and ocular itching, each 0-3; exclude broader non-nasal constructs containing ear/palate symptoms
RQLQ-SAR	Total RQLQ, 0-6	MD; negative favors active	Days 1-14	Compatible total score and treatment period
RQLQ-PER	Total RQLQ, 0-6	MD; negative favors active	Days 1-28	Compatible total score and treatment period
Any AE-SAR	At least one all-causality AE	OR; <1 favors active	14-day randomized treatment	All-causality preferred; no post-treatment follow-up
Any AE-PAR	At least one all-causality AE	OR; <1 favors active	28-day randomized treatment	All-causality preferred; no post-treatment follow-up
WDAE-SAR	Withdrawal because of AE	OR; <1 favors active	14-day randomized treatment	Randomized-treatment withdrawals only
WDAE-PAR	Withdrawal because of AE	OR; <1 favors active	28-day randomized treatment	Randomized-treatment withdrawals only

Longer exposures/follow-up-inclusive periods were retained only in labelled sensitivity/exploratory layers. Okubo et al. used four equally weighted nasal items scored 0-4 (T4NSS 0-16). Source-scale contrasts −1.084 (SE 0.208) and −1.509 (SE 0.215) were transformed exactly ×0.75 to −0.813 (SE 0.156) and −1.132 (SE 0.161) on 0-12 before synthesis. Range normalization does not prove semantic equivalence of the 0-4 and 0-3 anchors and is acknowledged as a limitation.

S7. Somnolence, sedation, and other unsynthesized harms

Table S7. Evidence-readiness assessment for somnolence-related and other functionally important harms.

**Audit domain**	**Finding**
Terminology	Somnolence, sedation, drowsiness, sleepiness, fatigue, and related central-nervous-system terms were not used consistently.
Construct definition	Reports often did not distinguish spontaneous adverse-event reporting from prospectively elicited symptoms or psychomotor outcomes.
Denominators	Outcome-specific participant denominators were frequently absent or not separable from broad safety populations.
Risk period	Randomized-treatment and post-treatment periods were not consistently distinguishable.
Count type	Some reports used event counts rather than numbers of participants with at least one event.
Connectivity	A sufficiently connected, regimen-specific, source-verified evidence structure could not be assembled.
Final disposition	No quantitative synthesis. The absence of a model-ready dataset was treated as an evidence gap, not evidence of no difference.
Serious adverse events	Not a target synthesis; any available SAE reporting remained descriptive and was not inferred from Any AE or WDAE.
Psychomotor/driving safety	Not quantitatively synthesized because outcome constructs and ascertainment were incompatible or incomplete.

S8. Network Geometry, Structural Estimability, and Active-Active Audit

Table S8A. Composition and structural status of the nine evidence structures.

Structure	Reports/ units	Arms	Information count and type	Represented N	Events	Active nodes	Structural status		
TNSS-SAR	11/12	26	14 model-ready contrasts	6,852*	-	7	Connected; global incoherence estimable (p=0.065)		
TNSS-PAR	2/2	6	4 model-ready contrasts	1,004*	-	3	Connected; global incoherence estimable (p=0.317)		
TOSS-SAR	2/3	7	4 model-ready contrasts	1,761*	-	3	Connected; global incoherence non-estimable (0 additional df)		
RQLQ-SAR	6/6	13	7 model-ready contrasts	3,700*	-	4	Connected; global incoherence estimable (p=0.279); heterogeneity not informatively estimated		
RQLQ-PER	2/2	4	2 model-ready contrasts	1,093*	-	2	Consistency model saturated; heterogeneity and global incoherence non-estimable		
Any AE-SAR	9/9	23	14 likelihood dimensions	5,648	1,351	9	Connected; global incoherence estimable (p=0.984)		
Any AE-PAR	8/8	20	12 likelihood dimensions	3,239	1,080	10	Connected; local separation not robust; global test not estimated; incoherence: Major concerns		
WDAE-SAR, structural	15/17	38	21 structural contrasts	9,149	83	9	Connected; CINeMA incoherence domain: Major concerns for seven estimable effe		cts; two non-estimable effects: Not evaluable
WDAE-SAR, likelihood	-/16	35	19 likelihood dimensions	8,249	83	7	Connected likelihood subset; CINeMA incoherence domain: Major	concerns for seven estimable effects; rupatadine 10/20 mg non-estimable/not evaluable	
WDAE-PAR	8/8	22	14 likelihood dimensions	3,251	55	10	Connected; local separation not robust; global test not estimated; incoherence: Major concerns		

Note: Across structures, 67 randomization-structure contributions arose from 35 modeled randomizations; counts and denominators must not be summed across structures. Continuous represented N describes arm-level evidence geometry and may differ from exact analyzed samples. TOSS-SAR retained one residual degree of freedom for descriptive heterogeneity but zero additional degrees of freedom for the global design-by-treatment test. For Any AE-PAR and the estimable effects in both WDAE structures, neither robust local separation nor a final source-verified global test was available; the CINeMA incoherence domain was therefore conservatively rated Major concerns. The two non-estimable WDAE-SAR effects remain Not evaluable. The WDAE-SAR all-zero three-arm unit (n = 900) remained in structural totals but contributed no relative-effect information.

Table S8 (continued). Active-active comparison audit.

Continuous structure	Active-active comparisons	Directly informed	Indirect-only through placebo
TNSS-SAR	21	2	19
TNSS-PAR	3	3	0
TOSS-SAR	3	1	2
RQLQ-SAR	6	1	5
RQLQ-PER	1	0	1
Total	34	7	27

A comparison was directly informed when at least one randomized design contained both regimens. None had independently estimable direct and indirect components. Complete estimates and appraisal judgments are reported in Tables S26-S27. After accounting for cycle dimensions induced within multi-arm randomizations (one per three-arm and three per four-arm design), no additional cycle dimension spanning distinct randomized designs remained across the nine structures.

Table S8B. Expanded network-geometry diagnostics.

Structure	Randomized units	Arms	Independent contrasts / likelihood dimensions	Unique direct edges	Two-arm designs	Three-arm designs	Four-arm designs	Connected components	Within-multiarm cycle dimensions	Residual df under consistency model
TNSS-SAR	12	26	14	9	10	2	0	1	2	7.0
TNSS-PAR	2	6	4	6	1	0	1	1	3	1.0
TOSS-SAR	3	7	4	4	2	1	0	1	1	1.0
RQLQ-SAR	6	13	7	5	5	1	0	1	1	3.0
RQLQ-PER	2	4	2	2	2	0	0	1	0	0.0
Any AE-SAR	9	23	14	14	4	5	0	1	5	NA
Any AE-PAR	8	20	12	14	4	4	0	1	4	NA
WDAE-SAR, structural	17	38	21	13	13	4	0	1	4	NA
WDAE-PAR	8	22	14	17	3	4	1	1	7	NA

Cycle dimensions are graph-theoretic dimensions induced within multi-arm randomizations (one per three-arm and three per four-arm design); no additional cycle dimension spanned distinct randomized designs. Residual df are reported for continuous consistency models; binary rows use likelihood dimensions and are marked NA.

S9. Transitivity, indirectness, and incoherence support

Table S9. Reporting and assessment of candidate effect modifiers.

Candidate modifier	Recoverability	Interpretation
Rhinitis category	Generally recoverable	SAR, PAR, and PER analyzed separately; categories may overlap clinically.
Treatment period / assessment timing	Generally recoverable	Primary 14- and 28-day windows applied.
Outcome definition and components	Generally recoverable after source review	Incompatible constructs excluded or moved to sensitivity layers.
Allergen exposure	Incomplete	Natural exposure required, but intensity and timing were unevenly reported.
Baseline symptom burden	Incomplete	Could not be compared quantitatively across all contributing units.
Placebo response	Incomplete	Potentially important in placebo-anchored structures.
Cointerventions	Incomplete	Trial context considered, especially partial-arm trials.
Age distribution	Incomplete in some reports	Pediatric-only trials excluded; mixed-age assumptions classified as exploratory where needed.
Result-specific risk of bias	Available through RoB 2	Applied to the evidence contributing to each estimate.
Overall transitivity judgment	Qualitative only	A fully quantitative assessment was not possible; exchangeability was not inferred from class membership or failure to detect incoherence.

Table S9 (continued). Final CINeMA indirectness distribution.

CINeMA indirectness judgment	Number	Location/interpretation
Some concerns	81	81 of the 89 evaluable estimates; incomplete effect-modifier reporting and placebo-anchored indirectness
Major concerns	8	TNSS-PAR 5/6; TNSS-SAR 2/28; Any AE-SAR 1/9
Not evaluable	2	Non-estimable WDAE-SAR effects

Table S9 (continued). Global and local incoherence assessment.

Structure	Assessment	Global status	Additional df	p value	Local status	CINeMA incoherence	Qualification
TNSS-SAR	Design-by-treatment interaction	Estimable	1	0.065	No independently estimable local direct-indirect separation	Some concerns	Least reassuring; sparse and weakly replicated.
TNSS-PAR	Design-by-treatment interaction	Estimable	1	0.317	No independently estimable local direct-indirect separation	No concerns	Limited power.
TOSS-SAR	Design-by-treatment interaction	Not estimable; 0 additional df	0		Local separation unavailable	Major concerns	One residual df remained for descriptive heterogeneity, but none for the global test.
RQLQ-SAR	Design-by-treatment interaction	Estimable	1	0.279	No independently estimable local direct-indirect separation	No concerns	Global test used only as fallback.

Structure	Assessment	Global status	Additional df	p value	Local status	CINeMA incoherence	Qualification
RQLQ-PER	Consistency model saturated	Not estimable; 0 additional df	0		Local separation unavailable	Major concerns	Heterogeneity and global incoherence not identifiable.
Any AE-SAR	Design-by-treatment interaction	Estimable	4	0.984	Independent indirect component not estimable	No concerns	Limited power.
Any AE-PAR	Final source-verified network	Not estimated after final freeze			Local separation not robustly identifiable	Major concerns	Major concerns was assigned conservatively because neither robust local separation nor a final source-verified global test was available; absence of a test is not evidence of consistency or incoherence.
WDAE-SAR	Final source-verified likelihood	Not estimated after final freeze			Local separation not robustly identifiable; two active effects non-estimable	Major concerns	For seven estimable effects, Major concerns was assigned conservatively because neither robust local separation nor a final source-verified global test was available; two non-estimable effects remain not evaluable.
WDAE-PAR	Final source-verified network	Not estimated after final freeze			Local separation not robustly identifiable	Major concerns	Major concerns was assigned conservatively because neither robust local separation nor a final source-verified global test was available; absence of a test is not evidence of consistency or incoherence.

Failure to detect, estimate, or reestimate incoherence was not interpreted as evidence of consistency. For the 27 estimable placebo-referenced comparisons in Any AE–PAR, WDAE–SAR, and WDAE–PAR, the absence of both robust local direct–indirect separation and a final source-verified global test supports a conservative Major concerns rating. The two non-estimable WDAE–SAR effects remain Not evaluable.

S10. Sensitivity and exploratory analysis map

Table S10. Sensitivity and exploratory analysis map.

Domain	Classification	Affected evidence	Main finding
Source completeness / scale compatibility	Pre-final-model robustness	Continuous structures	Stricter source/scale restrictions reduced several structures. After source verification, the Kuna three-item ocular 0-9 construct met the primary TOSS rule; no separate construct-exclusion TOSS-SAR sensitivity remained.
Alternative treatment periods	Pre-final-model robustness / extended period	Continuous and binary structures	Assessed dependence on treatment timing; longer exposure retained only in labelled analyses.
Alternative event definitions/counts/denominators	Pre-final-model source/event/denominator sensitivity	Binary structures	Evaluated source-supported alternatives for Bronsky, Philip, Ratner, and Meltzer.
Partial-arm exclusion	Pre-final-model robustness	Affected structures	Assessed dependence on eligible antihistamine/placebo arms from trials with ineligible arms.
Change-from-baseline vs final value	Pre-final-model estimand sensitivity	Continuous structures	Compared compatible inputs; incompatible constructs/scales/periods were excluded.
Fixed vs random effects	Pre-final-model model-form sensitivity	Continuous structures	Compared assumptions where structure/model remained identifiable.
Heterogeneity prior scale 1.0	Pre-final-model prior sensitivity	Binary structures	Cetirizine WDAE-SAR PrI widened to include 1; rupatadine 20 mg Any AE-SAR CrI widened to include 1.
Higher-risk-of-bias exclusion	Pre-final-model robustness	Selected structures	Performed only where exclusion left structure/model identifiable.
Influential trial/family removal	Pre-final-model leave-one-out	Selected structures	Performed only where removal left structure/model identifiable.
Mixed-age eligibility	Exploratory evidence expansion	TNSS-SAR	Added mixed-age Ratner phase 3 result.
Alternative scale/rater compatibility	Exploratory evidence expansion	TNSS-PAR	Added mizolastine from Bachert under broader compatibility.
Per-protocol population	Exploratory evidence expansion	TNSS-PAR	Added controlled-release bepotastine from Mo under per-protocol estimand.
Kowalski expansion	Exploratory evidence expansion	TNSS-PAR	Added four-arm trial; unreported covariance approximated as zero.
Combined Bachert-Kowalski-Mo assumptions	Combined stress test	TNSS-PAR	Jointly applied three assumptions; not counted as a fifth individual expansion.
ROB-MEN contribution indicators	Policy sensitivity	91 estimates x 4 indicators	85 some concerns, 4 low risk, 2 not rateable at 10/15/20/25% indicators.
ROB-MEN conservative small-study-effect policy	Policy sensitivity	91 estimates	Four low-risk judgments shifted to some concerns: 89 some concerns, 0 low risk, 2 not rateable.

Note: Pre-final-model denotes a decision documented before final model inspection and does not imply prospective public registration. Four individual evidence-expansion analyses and one combined stress test were conducted. The 455 ROB-MEN sensitivity rows comprised 364 contribution-indicator rows plus 91 conservative-policy rows. Policy sensitivities changed appraisal classifications only and did not refit treatment-effect models.

S11. Computation, diagnostics, calibration, and implementation-independent replication

Table S11. Computational specifications, diagnostic gates, and replication tolerances.

Component	Specification
Software	R 4.6.1; CmdStan 2.39.0; CmdStanR 0.9.0
Bayesian sampling	Four chains; non-centered parameterizations where appropriate; scenario-specific schedules reported below
Relative-effect prior	Normal(0, 1.5^2) on the log-odds scale
Study-baseline prior	Normal(0, 2.5^2) on the log-odds scale
Heterogeneity prior	Half-normal scale 0.5 in primary binary analyses; scale 1.0 sensitivity
Frozen sampling gates	Rank-normalized split R-hat ≤ 1.01; bulk and tail ESS ≥ 1,000; E-BFMI ≥ 0.30 in every chain; no divergences or maximum-treedepth hits; successful return codes
Additional checks	Trace plots, prior-predictive checks, posterior predictive checks, and energy diagnostics
Simulation-based calibration	Thirty deterministic datasets; predefined rank-uniformity and coverage criteria; at least 27 diagnostically valid replicates
Direct binary contrast	Unadjusted OR with two-sided 95% conditional exact CI by inversion of Fisher noncentral hypergeometric distribution
Binary numerical tolerances	Absolute differences ≤ 0.10 in median log-OR, ≤ 0.20 at either 95% interval limit, and ≤ 0.15 in median τ
Continuous numerical tolerance	Effect, interval, prediction-limit, and heterogeneity differences ≤ 1×10^{-4}
Boundary rule	Near-null label discrepancy classified boundary-indeterminate post hoc; no input or fit changed

Table S11 (continued). Scenario-specific execution schedules used in implementation-independent replication.

Component	Scope	Chains	Warmup/chain	Post-warmup/chain	adapt_delta	Max tree depth	Qualification
Binary source-primary and sensitivity fits	12 scenarios	4	3,000	6,000	0.995	16	Real-data Bayesian binomial-logit models
Simulation-based calibration	30 replicates	4	2,500	1,500	0.999	18	Deterministic simulations; aggregate calibration gates
Continuous GLS/REML	10 scenarios	N/A	N/A	N/A	N/A	N/A	Deterministic reconstruction; tolerance 1 × 10-4
Direct continuous components	3 components	N/A	N/A	N/A	N/A	N/A	Deterministic shared-control covariance reconstruction

Table S11 (continued). Implementation-independent replication results.

Validation component	Result
Real-data HMC gates	12/12 passed
Heterogeneity comparisons	12/12 passed
Primary binary magnitude comparisons	4/4 passed
Exact null-label classifications	3/4; one boundary-indeterminate
Continuous GLS/REML scenarios	10 plus three direct components reproduced
Simulation-based calibration	30/30 passed
Frozen inputs preserved	Yes
Refit for boundary adjudication	No

S12. Full-corpus crosswalk, eligibility disposition, and denominator reconciliation

Table S12A. Full-corpus report-randomization-entry crosswalk and cohort-level denominator reconciliation (66 retained entries; 64 non-overlapping randomized cohorts).

Entry	Report ID	Randomization/ cohort ID	Principal / linked report(s)	Category	Year and report/study label	Link role	Distinct cohort?	Source randomized N	Incremental randomized N	Analytical disposition	Counting / reconciliation qualification
1	E15	RU_E15	E15	PAR	1998; Mizolastine therapy also has an effect on nasal blockade in perennial allergic rhinoconjunctivitis. RIPERAN Study Group.	Primary report	Yes	257	257	Model-ready	Counted unique cohort
2	E16	RU_E16	E16	PAR	1999; A 12-week, placebo-controlled study of the efficacy and safety of ebastine, 10 and 20 mg once daily, in the treatment of perennial allergic rhinitis	Primary report	Yes	290	290	Evidence-map-only	Counted unique cohort
3	E17	RU_E17	E17	PAR	1998; Efficacy and tolerability comparison of ebastine 10 and 20 mg with loratadine 10 mg in perennial allergic rhinitis.	Primary report	Yes	317	317	Model-ready	Counted unique cohort; four-week randomized duration from the source; the historical 12-week value does not correspond to this trial.
4	E18	RU_E18	E18	PAR	2002; Mizolastine provides effective symptom relief in perennial allergic rhinitis: a double-blind, placebo-controlled study versus loratadine.	Primary report	Yes	428	428	Model-ready	Counted unique cohort
5	E19	RU_E19	E19	PER	2009; Desloratadine relieves nasal congestion and improves quality of life in persistent allergic rhinitis.	Primary report	Yes	584	584	Model-ready	Counted unique cohort
6	E20	RU_E20	E20	PAR	2009; Rupatadine 10 and 20 mg are effective and safe in perennial allergic rhinitis after 4 weeks of treatment.	Primary report	Yes	283	283	Model-ready	Counted unique cohort
7	E21	RU_E21	E21	PAR	2011; Morning and evening efficacy evaluation of rupatadine (10 and 20 mg), compared with cetirizine 10 mg in perennial allergic rhinitis: a randomized, double-blind, placebo-controlled trial	Primary report	Yes	282	282	Model-ready	Counted unique cohort; partial denominators: 282 randomized, 273 treated, and 269 in the ITT set. Randomized allocation by arm could not be recovered with sufficient certainty.
8	E22	RU_E22	E22	PAR	2021; Double-Blind Placebo-Controlled Trial of Controlled-Release Bepotastine Salicylate in Patients With Allergic Rhinitis	Primary report	Yes	272	272	Model-ready	Controlled-release (CR) bepotastine salicylate 20 mg once daily; source population classified as PAR.
9	E23	RU_E23	E23	PAR	2010; Rupatadine 10 mg in adolescent and adult symptom relief of perennial allergic rhinitis	Primary report	Yes	223	223	Evidence-map-only	Counted unique cohort; The combined efficacy/safety set comprised 219 participants; the randomized denominator was retained.
10	E24	RU_E24	E24	PAR	1998; Comparison of efficacy and safety of cetirizine and ebastine in perennial allergic rhinitis.	Primary report	Yes	215	215	Evidence-map-only	Counted unique cohort; Partial denominators; 215 randomized; arm-level ITT: cetirizine 106 and ebastine 108.
11	E25	RU_E25	E25	PAR	2007; Efficacy of montelukast for treating perennial allergic rhinitis	Primary report	Yes	1365	1365	Model-ready	Counted unique cohort
12	E26	RU_E26	E26	PAR	2003; Efficacy and safety of desloratadine in the treatment of perennial allergic rhinitis.	Primary report	Yes	676	676	Model-ready	Counted unique cohort
13	E27	RU_E27	E27	PAR	2024; Efficacy and safety of rupatadine fumarate in the treatment of perennial allergic rhinitis: A multicenter, double-blinded, randomized, placebo-controlled, bridging study in Koreans	Primary report	Yes	330	330	Model-ready	Immediate-release bepotastine 10 mg twice daily was retained separately from the controlled-release (CR) bepotastine salicylate 20 mg once-daily formulation in E22.

Entry	Report ID	Randomization/ cohort ID	Principal / linked report(s)	Category	Year and report/study label	Link role	Distinct cohort?	Source randomized N	Incremental randomized N	Analytical disposition	Counting / reconciliation qualification
14	E67	RU_E67	E67	PER	2004; Levocetirizine improves quality of life and reduces costs in long-term management of persistent allergic rhinitis (XPERT)	Primary report	Yes	551	551	Model-ready	Counted unique cohort
15	E68	RU_E68	E68	PER	2010; Efficacy of Desloratadine in Persistent Allergic Rhinitis - A GA2LEN Study	Primary report	Yes	716	716	Evidence-map-only	Counted unique cohort
16	E69	RU_E69	E69	PER	2008; A 12-week placebo-controlled study of rupatadine 10 mg once daily compared with cetirizine 10 mg once daily in persistent allergic rhinitis.	Primary report	Yes	543	543	Evidence-map-only	Counted unique cohort
17	E70	RU_E70	E70	PER	2012; The Loss of Smell in Persistent Allergic Rhinitis Is Improved by Levocetirizine due to Reduction of Nasal Inflammation but Not Nasal Congestion (the CIRANO Study)	Primary report	Yes	27	27	Evidence-map-only	Counted unique cohort
18	E71	RU_E71	E71	PAR	2014; Effect of cetirizine on symptom severity and quality of life in perennial allergic rhinitis	Primary report	Yes	321	321	Model-ready	Counted unique cohort
19	E80	RU_E80	E80	PAR	2006; Desloratadine therapy for symptoms associated with perennial allergic rhinitis.	Primary report	Yes	1179	1179	Evidence-map-only	Counted unique cohort
20	E81	RU_E81	E81	PAR	2012; Efficacy and safety of bilastine 20 mg compared with cetirizine 10 mg and placebo in perennial allergic rhinitis.	Primary report	Yes	651	651	Model-ready	Counted unique cohort; 651 randomized; 650 safety population; 641 ITT.
21	E127	RU_E127	E127	PAR	2003; Levocetirizine is effective for symptom relief including nasal congestion in adolescents and adults with perennial allergic rhinitis sensitized to house dust mites	Primary report	Yes	294	294	Evidence-map-only	Counted unique cohort; The T4SS includes ocular itching and excludes nasal congestion; it is not the canonical TNSS.
22	E28	RU_E28	E28	SAR	1997; The Effect of Fluticasone Propionate Aqueous Nasal Spray on Eosinophils and Cytokines in Nasal Secretions of Patients with Ragweed Allergic Rhinitis	Primary report	Yes	45	45	Evidence-map-only	Counted unique cohort; The inactive control was intranasal placebo; it was not a matched oral placebo.
23	E29	RU_E29_FFU109045	E29	SAR	2009; Fluticasone furoate nasal spray is more effective than fexofenadine for nighttime symptoms of seasonal allergy	Independent randomization within multi-trial report	Yes	936	936	Model-ready	Counted unique cohort
24	E29	RU_E29_FFU109047	E29	SAR	2009; Fluticasone furoate nasal spray is more effective than fexofenadine for nighttime symptoms of seasonal allergy	Independent randomization within multi-trial report	Yes	680	680	Model-ready	Counted unique cohort
25	E31	RU_E31	E31	SAR	2009; Comparison of the efficacy and safety of bilastine 20 mg versus desloratadine 5 mg in seasonal allergic rhinitis patients.	Primary report	Yes	721	721	Model-ready	Counted unique cohort; 721 randomized; 720 received study medication.
26	E32	RU_E32	E32	SAR	2002; Safety and efficacy of desloratadine 5 mg in asthma patients with seasonal allergic rhinitis and nasal congestion.	Primary report	Yes	331	331	Evidence-map-only	Counted unique cohort; Included seasonal rhinitis with concomitant mild asthma; recorded as a potential effect modifier.

Entry	Report ID	Randomization/ cohort ID	Principal / linked report(s)	Category	Year and report/study label	Link role	Distinct cohort?	Source randomized N	Incremental randomized N	Analytical disposition	Counting / reconciliation qualification
27	E33	RU_E33	E33	SAR	1995; Comparative efficacy and safety of a once-daily loratadine-pseudoephedrine combination versus its components alone and placebo in seasonal allergic rhinitis.	Primary report	Yes	874	874	Model-ready	Counted unique cohort
28	E34	RU_E34	E34	SAR	1996; Fluticasone propionate aqueous nasal spray compared with terfenadine tablets in the treatment of seasonal allergic rhinitis	Primary report	Yes	348	348	Evidence-map-only	Counted unique cohort; Double-dummy design; the inactive arm matched both formulations.
29	E49	RU_E49_FALL1999	E49; linked: E49; E35	SAR	2002; Efficacy and tolerability of montelukast alone or in combination with loratadine in seasonal allergic rhinitis.	Primary report	Yes	907	907	Model-ready	Counted unique cohort
30	E35	RU_E49_FALL1999	E49; linked: E49; E35	SAR	2002; Efficacy and tolerability of montelukast alone or in combination with loratadine in seasonal allergic rhinitis.	Pooled companion link	No	907	0	Model-ready	Linked, non-incremental; E35 Study 1 corresponds to the same randomization as E49 and was not duplicated.
31	E35	RU_E35_FALL2000	E35	SAR	2004; Montelukast for treating fall allergic rhinitis: effect of pollen exposure in three studies	Unique randomization in pooled report	Yes	830	830	Model-ready	Counted unique cohort; loratadine n = 171 was verified from the source-reported Study 2 disposition and arm totals.
32	E63	RU_E63_FALL2001	E63; linked: E63; E35	SAR	2003; Montelukast improves symptoms of seasonal allergic rhinitis over a 4-week treatment period	Primary report	Yes	1079	1079	Model-ready	Counted unique cohort; The trial randomized treatment over 4 weeks; the prespecified primary horizon was the mean of the first 14 days.
33	E35	RU_E63_FALL2001	E63; linked: E63; E35	SAR	2003; Montelukast improves symptoms of seasonal allergic rhinitis over a 4-week treatment period	Pooled companion link	No	1079	0	Model-ready	Linked, non-incremental; The trial randomized treatment over 4 weeks; the prespecified primary horizon was the mean of the first 14 days. E35 Study 3 corresponds to the same randomization as E63 and was not duplicated.
34	E37	RU_E37	E37	SAR	2009; Desloratadine for the treatment of cypress pollen-induced allergic rhinitis.	Primary report	Yes	233	233	Evidence-map-only	Counted unique cohort
35	E38	RU_E38	E38	SAR	2015; A comparison of fluticasone propionate nasal spray and cetirizine in ragweed fall seasonal allergic rhinitis	Primary report	Yes	682	682	Model-ready	Counted unique cohort; Four parallel arms without a double-dummy design; the oral contrast uses cetirizine versus capsule placebo.
36	E39	RU_E39	E39	SAR	1985; Comparative Study of the Efficacy, Tolerance and Side-Effects of Dexchlorpheniramine Maleate 6 mg B.I.D. with Terfenadine 60 mg B.I.D.	Primary report	Yes	174	174	Evidence-map-only	Counted unique cohort
37	E40	RU_E40	E40	SAR	2004; A randomized, double-blind, placebo-controlled study comparing ebastine 20 mg and 10mg with loratadine 10 mg once daily in seasonal allergic rhinitis.	Primary report	Yes	749	749	Evidence-map-only	Counted unique cohort
38	E42	RU_E42	E42	SAR	2009; Efficacy and safety of bilastine 20 mg compared with cetirizine 10 mg and placebo for seasonal allergic rhinitis.	Primary report	Yes	683	683	Model-ready	Counted unique cohort; 683 randomized, 681 treated, and 678 in the ITT set. Randomized arm allocation was not reported with sufficient certainty; efficacy denominators were 226/227/225.

Entry	Report ID	Randomization/ cohort ID	Principal / linked report(s)	Category	Year and report/study label	Link role	Distinct cohort?	Source randomized N	Incremental randomized N	Analytical disposition	Counting / reconciliation qualification
39	E44	RU_E44_PROTOCOL077	E44	SAR	2009; Clinical Studies of Combination Montelukast and Loratadine in Patients with Seasonal Allergic Rhinitis	Independent randomization within multi-trial report	Yes	632	632	Evidence-map-only	Counted unique cohort
40	E44	RU_E44_PROTOCOL102	E44	SAR	2009; Clinical Studies of Combination Montelukast and Loratadine in Patients with Seasonal Allergic Rhinitis	Independent randomization within multi-trial report	Yes	530	530	Evidence-map-only	Counted unique cohort
41	E45	RU_E45	E45	SAR	2013; A direct comparison of efficacy between desloratadine and rupatadine in seasonal allergic rhinoconjunctivitis.	Primary report	Yes	379	379	Evidence-map-only	Counted unique cohort; 379 randomized. Twenty participants randomized at one center were later excluded from all analysis populations; the analysis-valid denominator was 359.
42	E46	RU_E46	E46	SAR	2011; Olopatadine hydrochloride and rupatadine fumarate in seasonal allergic rhinitis: A comparative study of efficacy and safety	Primary report	Yes	70	70	Evidence-map-only	Counted unique cohort
43	E47	RU_E47	E47	SAR	2010; Rupatadine and Levocetirizine for Seasonal Allergic Rhinitis: A Comparative Study of Efficacy and Safety	Primary report	Yes	60	60	Evidence-map-only	Counted unique cohort
44	E48	RU_E48	E48	SAR	2000; Concomitant montelukast and loratadine as treatment for seasonal allergic rhinitis: a randomized placebo-controlled trial.	Primary report	Yes	460	460	Evidence-map-only	Counted unique cohort; partial denominators: 460 randomized and 458 reported as analyzable across the five arms.
45	E50	RU_E50	E50	SAR	2003; The health-related quality of life effects of once-daily cetirizine HCl in patients with seasonal allergic rhinitis: a randomized double-blind, placebo-controlled trial	Primary report	Yes	403	403	Model-ready	Counted unique cohort
46	E51	RU_E51	E51	SAR	2019; Efficacy and safety of rupatadine in Japanese patients with seasonal allergic rhinitis: a double-blind, randomized, multicenter, placebo-controlled clinical trial	Primary report	Yes	900	900	Model-ready	Source T4NSS used four equally weighted nasal items scored 0–4 (range 0–16). Arm mean-change contrasts and standard errors were transformed exactly ×0.75 to the canonical 0–12 TNSS scale before primary synthesis; no untransformed 0–16 effect entered the model.
47	E52	RU_E52	E52	SAR	2002; Montelukast for treating seasonal allergic rhinitis: a randomized, double-blind, placebo-controlled trial performed in the spring	Primary report	Yes	1302	1302	Model-ready	
48	E53	RU_E53	E53	SAR	2007; Desloratadine improves quality of life and symptom severity in patients with allergic rhinitis.	Primary report	Yes	534	534	Model-ready	Counted unique cohort
49	E54	RU_E54	E54	SAR	2009; Efficacy and safety of fixed-dose loratadine/montelukast in seasonal allergic rhinitis: Effects on nasal congestion	Primary report	Yes	1095	1095	Contextual/sensitivity only	Counted contextual cohort; No oral H1-antihistamine monotherapy arm.

Entry	Report ID	Randomization/ cohort ID	Principal / linked report(s)	Category	Year and report/study label	Link role	Distinct cohort?	Source randomized N	Incremental randomized N	Analytical disposition	Counting / reconciliation qualification
50	E55	RU_E55_NCT01241214	E55	SAR	2017; Efficacy and safety of setipiprant in seasonal allergic rhinitis: results from phase 2 and phase 3 randomized, double-blind, placebo- and active-referenced studies	Independent randomization within multi-trial report	Yes	579	579	Model-ready	Counted unique cohort
51	E55	RU_E55_NCT01484119	E55	SAR	2017; Efficacy and safety of setipiprant in seasonal allergic rhinitis: results from phase 2 and phase 3 randomized, double-blind, placebo- and active-referenced studies	Independent randomization within multi-trial report	Yes	630	630	Model-ready	Counted unique cohort
52	E57	RU_E57	E57	SAR	2004; Efficacy and safety of ebastine 20 mg compared with loratadine 10 mg once daily in seasonal allergic rhinitis.	Primary report	Yes	703	703	Evidence-map-only	Counted unique cohort
53	E58	RU_E58	E58	SAR	1998; A Comparison of the Efficacy of Fluticasone Propionate Aqueous Nasal Spray and Loratadine, Alone and in Combination, for the Treatment of Seasonal Allergic Rhinitis	Primary report	Yes	600	600	Evidence-map-only	Counted unique cohort; Double-dummy design.
54	E59	RU_E59	E59	SAR	2002; 24-hour efficacy of once-daily desloratadine therapy in seasonal allergic rhinitis [ISRCTN32042139].	Primary report	Yes	1036	1036	Evidence-map-only	Counted unique cohort
55	E60	RU_E60	E60	SAR	1988; Double-blind placebo-controlled study of loratadine, mequitazine, and placebo in the symptomatic treatment of seasonal allergic rhinitis	Primary report	Yes	69	69	Evidence-map-only	Counted unique cohort
56	E62	RU_E62	E62	SAR	2003; Randomized controlled trial evaluating the clinical benefit of montelukast for spring seasonal allergic rhinitis.	Primary report	Yes	1214	1214	Model-ready	Counted unique cohort
57	E64	RU_E64	E64	SAR	1994; Intranasal Fluticasone Propionate Is More Effective Than Terfenadine Tablets for Seasonal Allergic Rhinitis	Primary report	Yes	232	232	Evidence-map-only	Counted unique cohort; Double-dummy design.
58	E66	RU_E66	E66	SAR	2010; Efficacy and safety of levocetirizine in improving symptoms and health-related quality of life in US adults with seasonal allergic rhinitis: a randomized, placebo-controlled study	Primary report	Yes	580	580	Model-ready	Counted unique cohort
59	E74	RU_E74	E74	SAR	1999; Safety and efficacy of once-daily fexofenadine HCl in the treatment of autumn seasonal allergic rhinitis	Primary report	Yes	864	864	Model-ready	
60	E75	RU_E75_STAGE1	E75	SAR	1996; Model for outcomes assessment of antihistamine use for seasonal allergic rhinitis.	Primary report	Yes	131	131	Evidence-map-only	Counted unique cohort; The cetirizine dose varied between 5 and 10 mg; it was retained as a variable-dose node.
61	E76	RU_E76	E76	SAR	2010; Study of levocetirizine in seasonal allergic rhinitis	Primary report	Yes	596	596	Model-ready	Counted unique cohort
62	E77	RU_E77	E77	SAR	2005; Fexofenadine improves quality of life and work productivity in Japanese patients with seasonal allergic rhinitis.	Primary report	Yes	210	210	Evidence-map-only	Counted unique cohort; 210 randomized; 206 included in the reported quality-of-life analysis.

Entry	Report ID	Randomization/ cohort ID	Principal / linked report(s)	Category	Year and report/study label	Link role	Distinct cohort?	Source randomized N	Incremental randomized N	Analytical disposition	Counting / reconciliation qualification
63	E79	RU_E79	E79	SAR	2000; Comparison of the efficacy, safety and quality of life provided by fexofenadine hydrochloride 120 mg, loratadine 10 mg and placebo administered once daily for the treatment of seasonal allergic rhinitis	Primary report	Yes	688	688	Model-ready	Counted unique cohort; partial denominators: 688 randomized, 685 treated, and 639 in the ITT set. Food-condition information was not required for eligibility, node definition, estimand specification, or the model input and remains NR where not reported.
64	E84	RU_E84	E84	SAR	2006; Effect of desloratadine therapy on symptom scores and measures of nasal patency in seasonal allergic rhinitis: results of a single-center, placebo-controlled trial	Primary report	Yes	220	220	Model-ready	Counted unique cohort; 220 randomized; 219 safety population; 218 ITT.
65	E86	RU_E86	E86	SAR	1998; Effectiveness and Safety of Fexofenadine, a New Nonsedating H1-Receptor Antagonist, in the Treatment of Fall Allergies	Primary report	Yes	589	589	Evidence-map-only	
66	E140	RU_E140	E140	SAR	NR; Efficacy and Safety Study of Desloratadine (MK-4117) in Japanese Participants With Seasonal Allergic Rhinitis (MK-4117-204)	Primary report	Yes	449	449	Model-ready	Counted unique cohort; 449 randomized; one participant in the active arm did not receive treatment. Safety/analysis: 223 active and 225 placebo.

Note: Each row represents one retained report-randomization entry. Multiple reports arising from the same independent randomization are linked within the same randomization/cohort identifier, whereas a report describing multiple independent randomizations appears in more than one row. Source randomized N is the complete randomized denominator attached to the represented cohort. Incremental randomized N is the contribution to the deduplicated whole-corpus denominator; the two pooled E35 companion links contribute zero incremental participants. Incremental values sum to 34,831. A model-ready independent randomization contributed at least one eligible, estimand-compatible, quantitatively identifiable result to a principal evidence structure. Analytical disposition was determined irrespective of effect direction, magnitude, or statistical significance. NR, not reported.

Table S12B. Report-level exclusion decisions after eligibility assessment (n = 179): 176 uniquely excluded reports and three duplicate bibliographic records.

No.	Rayyan ID	Report citation	Primary exclusion reason	DOI / URL	Qualification
1	RAYYAN-625704167	Fexofenadine is efficacious and safe in children (aged 6-11 years) with seasonal allergic rhinitis Journal of Allergy and Clinical Immunology, 2003.	Wrong population	10.1067/mai.2003.1384	Exclusively pediatric population: children aged 6–11 years.
2	RAYYAN-603236555	Comparison of mizolastine with loratadine in the treatment of perennial allergic rhinitis. Rhinology, 1996.	Wrong outcome	PMID 8876072	
3	RAYYAN-603236568	Brompheniramine, terfenadine, and placebo in allergic rhinitis. Annals of allergy, asthma & immunology : official publication of the American College of Allergy, Asthma, & Immunology, 1996.	Wrong population	10.1016/S1081-1206(10)63334-0	
4	RAYYAN-603236575	Treatment of seasonal allergic rhinitis--a double blind, group comparative study of terfenadine and dexchlorpheniramine. Rhinology, 1987.	Wrong study duration	PMID 2883715	No dedicated duration code exists in the supplied controlled vocabulary.
5	RAYYAN-603236577	Comparison of ebastine to cetirizine in seasonal allergic rhinitis in adults. Annals of allergy, asthma & immunology : official publication of the American College of Allergy, Asthma, & Immunology, 1996.	Wrong outcome	10.1016/S1081-1206(10)63269-3	
6	RAYYAN-603236578	Rupatadine 10 mg and ebastine 10 mg in seasonal allergic rhinitis: a comparison study. Allergy, 2004.	Wrong outcome	10.1111/j.1398-9995.2004.00576.x	
7	RAYYAN-603236579	Brompheniramine maleate: a double-blind, placebo-controlled comparison with terfenadine for symptoms of allergic rhinitis. American journal of rhinology, 1998.	Wrong population	10.2500/105065898781389976	

No.	Rayyan ID	Report citation	Primary exclusion reason	DOI / URL	Qualification
8	RAYYAN-603236584	Efficacy and safety of mizolastine in seasonal allergic rhinitis. The Rhinase Study Group. Annals of allergy, asthma & immunology : official publication of the American College of Allergy, Asthma, & Immunology, 1996.	Wrong drug	10.1016/s1081-1206(10)63417-5	
9	RAYYAN-603236599	Fexofenadine reduces nasal congestion in perennial allergic rhinitis. Allergy, 2001.	Wrong outcome	10.1034/j.1398-9995.2001.00191.x	
10	RAYYAN-603236607	Double-blind comparison of two antihistamines: mequitazine and dexchlorpheniramine. Current medical research and opinion, 1978.	Wrong population	10.1185/03007997809111898	
11	RAYYAN-603236618	Efficacy and safety of levocetirizine in seasonal allergic rhinitis. Acta oto-rhino-laryngologica Belgica, 2001.	Wrong outcome	PMID 11859651	
12	RAYYAN-603236621	Efficacy of acrivastine with pseudoephedrine in treatment of allergic rhinitis due to ragweed. Annals of allergy, asthma & immunology : official publication of the American College of Allergy, Asthma, & Immunology, 1996.	Wrong drug	10.1016/S1081-1206(10)63423-0	
13	RAYYAN-603236634	[Clinical examination of mizolastine in the seasonal allergic rhinitis]. Lin chuang er bi yan hou ke za zhi = Journal of clinical otorhinolaryngology, 2004.	Wrong study design	PMID 15571316	
14	RAYYAN-603236654	Efficacy of acrivastine plus pseudoephedrine for symptomatic relief of seasonal allergic rhinitis due to mountain cedar. Annals of allergy, asthma & immunology : official publication of the American College of Allergy, Asthma, & Immunology, 1996.	Wrong drug	10.1016/S1081-1206(10)63460-6	
15	RAYYAN-603236671	SCH 434: a new antihistamine/decongestant for seasonal allergic rhinitis. The Journal of allergy and clinical immunology, 1989.	Wrong outcome	10.1016/0091-6749(89)90450-8	Final adjudication: Wrong outcome. The original RIS export did not retain the exclusion-reason field.
16	RAYYAN-603236678	Rupatadine 10 mg and cetirizine 10 mg in seasonal allergic rhinitis: a randomised, double-blind parallel study. Journal of investigational allergology & clinical immunology, 2005.	Wrong outcome	PMID 15864879	
17	RAYYAN-603236680	Comparison of once-daily ebastine 20 mg, ebastine 10 mg, loratadine 10 mg, and placebo in the treatment of seasonal allergic rhinitis. The Ebastine Study Group. The Journal of allergy and clinical immunology, 2000.	Wrong outcome	10.1067/mai.2000.105525	
18	RAYYAN-603236682	Efficacy and safety of cetirizine therapy in perennial allergic rhinitis. Annals of allergy, 1992.	Wrong outcome	PMID 1348405	
19	RAYYAN-603236683	Comparison of the efficacy, safety, and onset of action of mizolastine, cetirizine, and placebo in the management of seasonal allergic rhinoconjunctivitis. MIZOCET Study Group. Annals of allergy, asthma & immunology : official publication of the American College of Allergy, Asthma, & Immunology, 1999.	Wrong study design	10.1016/s1081-1206(10)62672-5	
20	RAYYAN-603236689	Double-blind study of cetirizine and loratadine versus placebo in patients with allergic rhinitis. Journal of investigational allergology & clinical immunology, 2000.	Wrong outcome	PMID 10780795	
21	RAYYAN-603236694	Comparative study of the efficacy and tolerance of terfenadine and clemastine in patients with seasonal allergic rhinitis. Arzneimittel-Forschung, 1982.	Wrong study design	PMID 6129872	
22	RAYYAN-603236718	Controlled trial of terfenadine and chlorpheniramine maleate in perennial rhinitis. Postgraduate medical journal, 1982.	Wrong study duration	10.1136/pgmj.58.681.422	No dedicated duration code exists in the supplied controlled vocabulary.
23	RAYYAN-603236719	Rapid and sustained efficacy of mizolastine 10 mg once daily in seasonal allergic rhinitis. The Journal of international medical research, 1998.	Wrong outcome	10.1177/030006059802600603	
24	RAYYAN-603236725	A double-blind, placebo-controlled study of the efficacy and safety of ebastine 20 mg once daily given with and without food in the treatment of seasonal allergic rhinitis. Journal of clinical pharmacology, 2002.	Wrong outcome	10.1177/009127002401382696	
25	RAYYAN-603236729	Comparison of efficacy, safety, and skin test inhibition of cetirizine and astemizole. Annals of allergy, asthma & immunology : official publication of the American College of Allergy, Asthma, & Immunology, 1996.	Wrong drug	10.1016/S1081-1206(10)60039-7	

No.	Rayyan ID	Report citation	Primary exclusion reason	DOI / URL	Qualification
26	RAYYAN-603236742	Desloratadine and levocetirizine improve nasal symptoms, airflow, and allergic inflammation in patients with perennial allergic rhinitis: a pilot study. International immunopharmacology, 2005.	Wrong outcome	10.1016/j.intimp.2005.05.008	
27	RAYYAN-603236743	Randomized controlled trial of desloratadine for persistent allergic rhinitis: correlations between symptom improvement and quality of life. Allergy and asthma proceedings, 2013.	Wrong outcome	10.2500/aap.2013.34.3668	
28	RAYYAN-603236755	A double-blind comparative trial of astemizole and terfenadine for the treatment of hay fever. Allergie et immunologie, 1989.	Wrong drug	PMID 2576206	
29	RAYYAN-603236758	Cetirizine therapy for seasonal allergic rhinitis: alternative dosage schedules. Clinical therapeutics, 1991.	Wrong outcome	PMID 1686422	
30	RAYYAN-603236766	[Comparative trial of 2 antihistaminics: mequitazine and dexchlorpheniramine]. Annales d'oto-laryngologie et de chirurgie cervico faciale : bulletin de la Societe d'oto-laryngologie des hopitaux de Paris, 1978.	Wrong outcome	PMID 33589	
31	RAYYAN-603236767	Treating intermittent allergic rhinitis: a prospective, randomized, placebo and antihistamine-controlled study of Butterbur extract Ze 339. Phytotherapy research : PTR, 2005.	Wrong drug	10.1002/ptr.1705	
32	RAYYAN-603236773	Efficacy of ebastine in the control of nasal congestion associated with allergic rhinitis. Methods and findings in experimental and clinical pharmacology, 2003.	Wrong study design	10.1358/mf.2003.25.2.723684	
33	RAYYAN-603236790	A multicentric study of loratadine, terfenadine and placebo in patients with seasonal allergic rhinitis. Arzneimittel-Forschung, 1988.	Background article	PMID 2896508	
34	RAYYAN-603236804	Clinical studies of the efficacy and tolerability of ebastine 10 or 20 mg once daily in the treatment of seasonal allergic rhinitis in the US. Drugs, 1996.	Wrong outcome	10.2165/00003495-199600521-00006	
35	RAYYAN-603236811	Levocetirizine in persistent allergic rhinitis and asthma: effects on symptoms, quality of life and inflammatory parameters. Clinical and experimental allergy : journal of the British Society for Allergy and Clinical Immunology, 2006.	Wrong outcome	10.1111/j.1365-2222.2006.02548.x	
36	RAYYAN-603236812	Efficacy of cetirizine in the treatment of seasonal allergic rhinoconjunctivitis. Annals of allergy, 1990.	Background article	PMID 1974401	
37	RAYYAN-603236817	[Efficacy and safety of fexofenadine and cetirizine in the treatment of allergic rhinitis]. Revista alergia Mexico (Tecamachalco, Puebla, Mexico : 1993), 2001.	Wrong population	PMID 11802289	
38	RAYYAN-603236818	Astemizole in perennial allergic rhinitis with seasonal exacerbations: a placebo-controlled double-blind study. Annals of allergy, 1989.	Wrong drug	PMID 2574549	
39	RAYYAN-603236819	Evaluation of the efficacy and safety of loratadine in perennial allergic rhinitis. The Journal of allergy and clinical immunology, 1989.	Wrong outcome	10.1016/0091-6749(89)90127-9	
40	RAYYAN-603236822	Oxatomide in the management of hay fever--a placebo-controlled double-blind study in general practice. Clinical allergy, 1981.	Wrong drug	10.1111/j.1365-2222.1981.tb01624.x	
41	RAYYAN-603236828	Comparative effects of loratadine and azatadine in the treatment of seasonal allergic rhinitis. Asian Pacific journal of allergy and immunology, 1990.	Wrong outcome	PMID 1982614	
42	RAYYAN-603236834	Astemizole: a new long-acting antihistamine in the treatment of seasonal allergic rhinitis. Clinical allergy, 1983.	Wrong drug	10.1111/j.1365-2222.1983.tb02580.x	
43	RAYYAN-603236835	A randomized, double-blind, parallel-group study, comparing the efficacy and safety of rupatadine (20 and 10 mg), a new PAF and H1 receptor-specific histamine antagonist, to loratadine 10 mg in the treatment of seasonal allergic rhinitis. Journal of investigational allergology & clinical immunology, 2004.	Wrong outcome	PMID 15160440	
44	RAYYAN-603236857	Efficacy of desloratadine, 5 mg, compared with fexofenadine, 180 mg, in patients with symptomatic seasonal allergic rhinitis. Allergy and asthma proceedings, 2006.	Wrong outcome	10.2500/aap.2006.27.2851	

No.	Rayyan ID	Report citation	Primary exclusion reason	DOI / URL	Qualification
45	RAYYAN-603236859	Reduction of soluble ICAM-1 levels in nasal secretion by H1-blockers in seasonal allergic rhinitis. Allergy, 1997.	Wrong outcome	10.1111/j.1398-9995.1997.tb02425.x	
46	RAYYAN-603236863	Astemizole--a new, non-sedating antihistamine for hayfever. The Journal of otolaryngology, 1985.	Wrong drug	PMID 2866257	
47	RAYYAN-603236872	[Comparison of astemizole and terfenadine in maintenance therapy of hay fever]. Zeitschrift fur Hautkrankheiten, 1985.	Wrong drug	PMID 2859712	
48	RAYYAN-603236876	Comparative trial of two non-sedative H1 antihistamines, terfenadine and astemizole, for hay fever. Thorax, 1984.	Wrong drug	10.1136/thx.39.9.668	
49	RAYYAN-603236893	French multicentre double-blind study to evaluate the efficacy and safety of acrivastine as compared with terfenadine in seasonal allergic rhinitis. The Journal of international medical research, 1989.	Wrong outcome	PMID 2570002	
50	RAYYAN-603236908	Levocetirizine improves nasal obstruction and modulates cytokine pattern in patients with seasonal allergic rhinitis: a pilot study. Clinical and experimental allergy : journal of the British Society for Allergy and Clinical Immunology, 2004.	Wrong outcome	10.1111/j.1365-2222.2004.01960.x	
51	RAYYAN-603236925	Comprehensive evaluation of cetirizine in the management of seasonal allergic rhinitis: impact on symptoms, quality of life, productivity, and activity impairment. Allergy and asthma proceedings, 2002.	Background article	PMID 12528605	
52	RAYYAN-603236941	Comparison of the efficacy and safety of loratadine, terfenadine, and placebo in the treatment of seasonal allergic rhinitis. The Journal of allergy and clinical immunology, 1988.	Wrong outcome	10.1016/0091-6749(88)90948-7	
53	RAYYAN-603236951	[The effect of desloratadine on quality of life of patients with seasonal allergic rhinitis]. Lin chuang er bi yan hou tou jing wai ke za zhi = Journal of clinical otorhinolaryngology head and neck surgery, 2008.	Wrong outcome	PMID 18720881	
54	RAYYAN-603236952	Double-blind, placebo-controlled study comparing the efficacy and safety of fexofenadine hydrochloride (120 and 180 mg once daily) and cetirizine in seasonal allergic rhinitis. The Journal of allergy and clinical immunology, 1999.	Wrong outcome	10.1016/s0091-6749(99)70070-9	
55	RAYYAN-603236964	Loratadine (SCH29851) 40 mg once daily versus terfenadine 60 mg twice daily in the treatment of seasonal allergic rhinitis. The Journal of international medical research, 1987.	Wrong outcome	10.1177/030006058701500201	
56	RAYYAN-603236965	A placebo-controlled comparison of the efficacy and tolerability of picumast dihydrochloride and terfenadine in patients with seasonal allergic rhinitis. Arzneimittel-Forschung, 1989.	Wrong outcome	PMID 2576363	
57	RAYYAN-603236966	Clinical evaluation of the efficacy and safety of noberastine, a new H1 antagonist, in seasonal allergic rhinitis: a placebo-controlled, dose-response study. The Journal of allergy and clinical immunology, 1991.	Wrong drug	10.1016/0091-6749(91)90250-r	
58	RAYYAN-603236986	Double-blind comparison of loratadine (SCH 29851), astemizole, and placebo in hay fever with special regard to onset of action. Annals of allergy, 1988.	Wrong outcome	PMID 2904776	
59	RAYYAN-603236987	Efficacy and safety of loratadine (10 mg once daily), terfenadine (60 mg twice daily), and placebo in the treatment of seasonal allergic rhinitis. The Journal of allergy and clinical immunology, 1989.	Wrong outcome	10.1016/0091-6749(89)90303-5	
60	RAYYAN-603236994	Double-blind multicenter controlled clinical study comparing the efficacy of picumast dihydrochloride versus astemizole and placebo in patients with seasonal allergic rhinitis. Arzneimittel-Forschung, 1989.	Wrong outcome	PMID 2576364	
61	RAYYAN-603237009	Comparison of the efficacy of ebastine 10mg and 20mg once daily with that of cetirizine 10mg once daily in adults with seasonal allergic rhinitis. A multicentre double-blind study. Drugs, 1996.	Wrong outcome	10.2165/00003495-199600521-00007	
62	RAYYAN-603237081	Pharmacologic prophylaxis of allergic rhinitis: relative efficacy of hydroxyzine and chlorpheniramine. The Journal of allergy and clinical immunology, 1981.	Wrong drug	10.1016/0091-6749(81)90065-8	
63	RAYYAN-603237098	The efficacy and sedative profile of astemizole and cetirizine in the treatment of grass pollen hayfever in general practice. The British journal of clinical practice, 1993.	Background article	PMID 8347437	

No.	Rayyan ID	Report citation	Primary exclusion reason	DOI / URL	Qualification
64	RAYYAN-603237099	Efficacy of loratadine versus placebo in the prophylactic treatment of seasonal allergic rhinitis. Annals of allergy, 1994.	Wrong outcome	PMID 8092558	
65	RAYYAN-603237112	Comparative study of cetirizine and terfenadine versus placebo in the symptomatic management of seasonal allergic rhinitis. Annals of allergy, asthma & immunology : official publication of the American College of Allergy, Asthma, & Immunology, 1996.	Wrong outcome	10.1016/S1081-1206(10)63462-X	
66	RAYYAN-603237137	Efficacy and safety of fexofenadine hydrochloride for treatment of seasonal allergic rhinitis. Annals of allergy, asthma & immunology : official publication of the American College of Allergy, Asthma, & Immunology, 1997.	Wrong outcome	10.1016/S1081-1206(10)63041-4	
67	RAYYAN-603237162	Double-blind assessment of azelastine in the treatment of perennial allergic rhinitis. Annals of allergy, 1994.	Wrong drug	PMID 8067597	
68	RAYYAN-603237174	Prophylactic treatment of seasonal allergic rhinitis. Clinical therapeutics, 1991.	Background article	PMID 1827615	
69	RAYYAN-603237177	[Multicenter hospital study on the efficacy of Zyrtec (R) (cetirizine) compared to a placebo, in the treatment of perennial allergic rhinitis in 254 adolescents aged 12-15]. Allergie et immunologie, 1995.	Wrong outcome	PMID 8851038	
70	RAYYAN-603237195	Once daily loratadine versus astemizole once daily. Annals of allergy, 1994.	Wrong study design	PMID 8067592	
71	RAYYAN-603237218	Treatment of hay fever with loratadine--a new non-sedating antihistamine. Allergy, 1990.	Background article	10.1111/j.1398-9995.1990.tb00463.x	
72	RAYYAN-603237221	Suppression of seasonal allergic rhinitis symptoms with daily hydroxyzine. The Journal of allergy and clinical immunology, 1979.	Wrong drug	10.1016/0091-6749(79)90203-3	
73	RAYYAN-603237224	A comparative study of clemastine ('Tavegil') and chlorpheniramine maleate in the treatment of hayfever. Current medical research and opinion, 1976.	Wrong population	10.1185/03007997609109312	
74	RAYYAN-603237246	Efficacy and safety of loratadine compared with astemizole in Malaysian patients with allergic rhinitis. Singapore medical journal, 1994.	Wrong population	PMID 7761882	
75	RAYYAN-603237253	Comparative study of the efficacy, tolerance and side-effects of dexchlorpheniramine maleate 6 mg b.i.d. with terfenadine 60 mg b.i.d. The Journal of international medical research, 1985.	Duplicate bibliographic record	10.1177/030006058501300507	Duplicate representation of retained report E39 (RAYYAN-625704157); no corpus or analytical effect.
76	RAYYAN-603237268	Multicenter, double-blind, multiple-dose, parallel-groups efficacy and safety trial of azelastine, chlorpheniramine, and placebo in the treatment of spring allergic rhinitis. The Journal of allergy and clinical immunology, 1988.	Wrong drug	10.1016/0091-6749(88)90082-6	
77	RAYYAN-603237318	Comparison of loratadine and terfenadine in allergic seasonal rhinoconjunctivitis with emphasis on nasal stuffiness and peak flow. Arzneimittel-Forschung, 1992.	Wrong outcome	PMID 1472143	
78	RAYYAN-603237336	Fexofenadine hydrochloride, 180 mg, exhibits equivalent efficacy to cetirizine, 10 mg, with less drowsiness in patients with moderate-to-severe seasonal allergic rhinitis. Annals of allergy, asthma & immunology : official publication of the American College of Allergy, Asthma, & Immunology, 2003.	Wrong outcome	10.1016/S1081-1206(10)61682-1	
79	RAYYAN-603237366	Clinical investigation of ketotifen in perennial allergic rhinitis: a double-blind comparative study of ketotifen and clemastine fumarate. Rhinology, 1984.	Wrong drug	PMID 6390659	
80	RAYYAN-603237383	Effectiveness and safety of fexofenadine, a new nonsedating H1-receptor antagonist, in the treatment of fall allergies. Allergy and asthma proceedings, 1998.	Duplicate bibliographic record	10.2500/108854198778604112	Duplicate representation of retained report E86 (RAYYAN-625704166); no corpus or analytical effect.
81	RAYYAN-603237403	[Multicenter double-blind study comparing astemizole and terfenadine for nonseasonal rhinitis in the adult]. Allergie et immunologie, 1988.	Background article	PMID 3144979	

No.	Rayyan ID	Report citation	Primary exclusion reason	DOI / URL	Qualification
82	RAYYAN-603237424	Comparative study of terfenadine and cetirizine in hay fever: assessment of efficacy and central nervous system effects. Journal of investigational allergology & clinical immunology, 1995.	Background article	PMID 7551204	
83	RAYYAN-603237473	Oxatomide in seasonal rhinoconjunctivitis. Allergy, 1980.	Wrong population	10.1111/j.1398-9995.1980.tb01773.x	
84	RAYYAN-603237554	A Phase III, Comparative Study for the Efficacy and Safety of Bilastine 20 mg Versus Cetirizine 10 mg and Placebo in the Treatment of Perennial Allergic Rhinitis During 4 Weeks, Followed by a Long-term Safety Extension With Bilastine 20 mg , 2010.	Background article	NCT01127620	
85	RAYYAN-603237578	Placebo Controlled Pilot Study on the Efficacy of Levocetirizine 5 mg in Reducing Symptoms, Airway Resistance, and Sleep Impairment in Patients With Persistent Allergic Rhinitis , 2006.	Wrong outcome	NCT00355771	
86	RAYYAN-603237636	[Cetirizine for treating allergic seasonal rhinitis]. Pneumonologia i alergologia polska, 1992.	Background article	PMID 1303775	
87	RAYYAN-603237657	Evaluation of cetirizine in patients with allergic rhinitis and perennial asthma. Annals of allergy, asthma & immunology : official publication of the American College of Allergy, Asthma, & Immunology, 1996.	Wrong outcome	10.1016/S1081-1206(10)63461-8	
88	RAYYAN-603237658	Two doses of triprolidine for treatment of allergic rhinitis. Annals of allergy, 1979.	Wrong population	PMID 378040	
89	RAYYAN-603237726	Estudio de la eficacia y seguridad del antihistamínico V0114CP 2.5 mg en el tratamiento de la rinitis alérgica estacional. Estudio aleatorizado, doble ciego, de tres ramas y grupos paralelos, que incluye una rama de placebo y otra de control activo (desloratadina 5 mg). EFFICACY AND SAFETY STUDY... , 2008.	Wrong outcome	EudraCT 2008-000133-22	
90	RAYYAN-603237842	Clinical investigation of terfenadine, a non-sedating antihistamine. Annals of allergy, 1980.	Wrong study duration	PMID 6102442	No dedicated duration code exists in the supplied controlled vocabulary.
91	RAYYAN-603237988	[Comparative study of the efficacy and tolerance of cetirizine verses astemizole in patients with allergic rhinitis]. Acta otorrinolaringologica espanola, 1992.	Wrong population	PMID 1355345	
92	RAYYAN-603238029	Comparison of antileukotrienes and antihistamines in the treatment of allergic rhinitis. American journal of rhinology, 2007.	Wrong outcome	10.2500/ajr.2007.21.3044	
93	RAYYAN-603238297	Effectiveness of bilastine and fexofenadine among allergic Rhinitis patients in Ranchi, Jharkhand, India. Bioinformation, 2022.	Wrong study design	10.6026/97320630018596	
94	RAYYAN-603238307	Efficacy and safety of setipiprant in seasonal allergic rhinitis: results from Phase 2 and Phase 3 randomized, double-blind, placebo- and active-referenced studies. Allergy, asthma, and clinical immunology, 2017.	Duplicate bibliographic record	10.1186/s13223-017-0183-z	Duplicate representation of retained report E55 (RAYYAN-625704161); the retained article contains two independent randomizations.
95	RAYYAN-603238517	Efficacy and Safety of Levocetirizine in Moderate-to-Severe Seasonal Allergic Rhinitis: A Randomized, Double-Blind, Placebo-Controlled Trial Int. J. Life Sci. Biotechnol. Pharma Res., 2026.	Wrong publication type	10.69605/ijlbpr_15.3.2026.214	
96	RAYYAN-603238801	National, Multicenter, Randomized, Double-blind, Phase III Clinical Trial to Evaluate the Efficacy and Safety of MAZ-101 in the Treatment of Persistent Allergic Rhinitis (PER) clinicaltrials.gov, 2022.	Wrong drug	Source URL in screening ledger	
97	RAYYAN-603238964	To evaluate efficacy and safety of rupatadine, bilastine, and levocetrizine in Allergic rhinitis at teritary care hospital, Telangana Asian J. Pharm. Clin. Res., 2021.	Wrong study design	10.22159/ajpcr.2021v14i12.43427	
98	RAYYAN-603239016	The effectiveness comparison of desloratadine and loratadine in reducing total nasal symptom score and the level of interleukin 4 in the nasal secretions of allergic rhinitis patients Indian J. Forensic Med. Toxicol., 2020.	Wrong population	Embase record L2004654237	
99	RAYYAN-603239092	H1 antihistamines on minimal persistent inflammation in patients with allergic rhinitis Eur. J. Clin. Pharmacol., 2019.	Background article	10.1007/s00228-019-02685-2	

No.	Rayyan ID	Report citation	Primary exclusion reason	DOI / URL	Qualification
100	RAYYAN-603239132	New antihistamine drug in treatment of patients with seasonal allergic rhinitis: Clinical study results Syst. Rev. Pharm., 2019.	Wrong study design	10.5530/srp.2019.2.14	
101	RAYYAN-603239235	How could we influence systemic inflammation in allergic rhinitis? The role of H1 antihistamines Oxidative Med. Cell. Longevity, 2018.	Background article	10.1155/2018/3718437	
102	RAYYAN-603239313	Posthoc analysis demonstrates oral cetirizine 10mg effectively relieves subjects' worst seasonal allergy symptom(S) J. Allergy Clin. Immunol., 2017.	Wrong study design	Embase record L615336351	
103	RAYYAN-603239314	Oral cetirizine 10mg significantly improves highest perennial allergic rhinitis symptom severity score(S) in posthoc analysis J. Allergy Clin. Immunol., 2017.	Wrong study design	Embase record L615336394	
104	RAYYAN-603239419	Randomized controlled study of desloratadine citrate and loratadine in the treatment of allergic rhinitis Int. J. Clin. Exp. Med., 2016.	Wrong population	Embase record L609237840	
105	RAYYAN-603239534	Cetirizine improves both ocular and nasal allergy symptoms in subjects with perennial allergic rhinitis J. Allergy Clin. Immunol., 2015.	Wrong publication type	Embase record L71789666	
106	RAYYAN-603239642	Cetirizine significantly relieves ocular allergy symptoms in subjects with seasonal allergic rhinitis J. Allergy Clin. Immunol., 2014.	Wrong publication type	10.1016/j.jaci.2013.12.987	
107	RAYYAN-603239643	Cetirizine effectively relieves both ocular allergy symptoms and nasal allergy symptoms in subjects with seasonal allergic rhinitis J. Allergy Clin. Immunol., 2014.	Wrong publication type	10.1016/j.jaci.2013.12.988	
108	RAYYAN-603239709	A comparative study of efficacy & Safety of rupatadine and levocetirizine in seasonal allergic rhinitis Indian J. Pharmacol., 2013.	Wrong publication type	Embase record L71394939	
109	RAYYAN-603239922	Rupatadine and desloratadine showed similar profile for seasonal allergic rhinoconjunctivitis Allergy Eur. J. Allergy Clin. Immunol., 2012.	Wrong outcome	10.1111/all.12033	
110	RAYYAN-603240416	Randomized, Double-blind, Placebo/Active-controlled, Multi-center Clinical Trial to Investigate the Safety/Tolerability and Efficacy of Bilastine 20mg After 14-day Oral Administration in Patients With Seasonal Allergic Rhinitis clinicaltrials.gov, 2011.	Background article	Source URL in screening ledger	
111	RAYYAN-603240972	Improvement in sleep quality and daily functioning correlate with reduced congestion in subjects with persistent allergic rhinitis treated with desloratadine 5 mg daily: Results from the aerius control: Clinical and evaluative profile of treatment-2 (ACCEPT2) trial in collaboration with global al... Allergy Eur. J. Allergy Clin. Immunol., 2009.	Wrong publication type	10.1111/j.1398-9995.2009.02078.x	
112	RAYYAN-603241026	A Multi-Centre, Double-Blind, Randomised Comparison Of The Effects Of Desloratadine (Aerius®) And Placebo In The Relief Of Nasal Symptom Scores In Subjects With Seasonal Allergic Rhinitis (Sar) To Cypress Pollen clinicaltrials.gov, 2009.	Background article	Source URL in screening ledger	
113	RAYYAN-603241040	Randomized, placebo-controlled, multicenter trial evaluating the effects of levocetirizine on symptoms, work productivity, and daytime sleepiness in US adults with seasonal allergic rhinitis Allergy Asthma Proc., 2009.	Background article	Embase record L70148905	
114	RAYYAN-603241053	In subjects with persistent allergic rhinitis (PER) treated with desloratadine, improvements in Total 5-Symptom Scores (T5SS) and Rhinoconjunctivitis Quality of Life Questionnaire-Standardized (RQLQ-S) correlate with disease burden as measured using a simple Visual Analog Scale (VAS) J. Allergy Clin. Immunol., 2009.	Wrong study design	Embase record L70105619	
115	RAYYAN-603241056	Decrease in burden of disease, as measured on a simple visual analog scale, correlates significantly with improvements in total symptom score and quality of life in desloratadine-treated subjects with intermittent allergic rhinitis: Results of the ACCEPT1 study J. Allergy Clin. Immunol., 2009.	Wrong study design	Embase record L70105626	
116	RAYYAN-603241184	A Placebo Controlled Study of the Efficacy and Safety of Desloratadine vs. Fexofenadine 180 mg. in the Treatment of Subjects With Symptomatic Seasonal Allergic Rhinitis (SAR) clinicaltrials.gov, 2008.	Background article	Source URL in screening ledger	

No.	Rayyan ID	Report citation	Primary exclusion reason	DOI / URL	Qualification
117	RAYYAN-603241651	A Multi-Center Randomized, Double-Blind, Placebo-Controlled, Parallel Study to Evaluate the Efficacy, Safety and Pharmacokinetics of Once or Twice Daily Bilastine (10 or 20 mg) Compared With Placebo Given Orally in The Treatment of the Symptoms of Seasonal Allergic Rhino-ConjunctivitisWith Aller... clinicaltrials.gov, 2007.	Wrong publication type	Source URL in screening ledger	
118	RAYYAN-603241872	Fexofenadine is effective and well tolerated in seasonal allergic rhinitis: Results from a placebo-controlled study in japan JOURNAL OF ALLERGY AND CLINICAL IMMUNOLOGY, 2001.	Wrong publication type		
119	RAYYAN-603241901	Ebastine 10 and 20mg are able to control all symptoms associated to allergic rhinitis. An efficacy placebo-controlled comparative clinical trial ALLERGY, 2002.	Wrong publication type		
120	RAYYAN-603241974	An evaluation of the ocular drying effects of 2 systemic antihistamines: loratadine and cetirizine hydrochloride ANNALS OF ALLERGY ASTHMA & IMMUNOLOGY, 2004.	Wrong publication type	10.1016/S1081-1206(10)61413-5	
121	RAYYAN-603242457	A DOUBLE-BLIND RANDOMIZED EVALUATION OF ASTEMIZOLE IN COMPARISON WITH PLACEBO IN THE TREATMENT OF SEASONAL ALLERGIC RHINITIS JOURNAL OF ALLERGY AND CLINICAL IMMUNOLOGY, 1985.	Wrong publication type	10.1016/0091-6749(85)90382-3	
122	RAYYAN-603243655	CETIRIZINE (C) VERSUS ASTEMIZOLE (A) IN THE TREATMENT OF SEASONAL ALLERGIC RHINITIS (SAR) - A DOUBLE-BLIND, PARALLEL-GROUP, MULTICENTER STUDY JOURNAL OF ALLERGY AND CLINICAL IMMUNOLOGY, 1994.	Wrong publication type		
123	RAYYAN-603243837	A double-blind, placebo-controlled study on the efficacy of Rinasek in symptomatic treatment of seasonal and non-seasonal rhinitis Dvostruko slepa placebom kontrolisana studija za ocenu efikasnosti primene leka Rinasek u simptomatskom lecenju sezonskog i nesezonskog rinitisa., 1995.	Wrong drug	Embase record L126185152	
124	RAYYAN-603243885	Effects of emedastine on Japanese cedar pollinosis. A multicentered, double-blind study PRACT. OTOL., 1995.	Wrong study design	Embase record L25193115	
125	RAYYAN-603243993	Double blind randomized clinical trial of cetirizine in the treatment of perennial allergic rhinitis INDIAN J. PHARMACOL., 1994.	Wrong outcome	Embase record L24212437	
126	RAYYAN-603244056	Efficacy and tolerability of once-daily 5mg desloratadine, an H₁-receptor antagonist, in patients with seasonal allergic rhinitis - Assessment during the spring and fall allergy seasons CLINICAL DRUG INVESTIGATION, 2001.	Wrong publication type	10.2165/00044011-200121010-00004	
127	RAYYAN-603244083	A phase 3 study comparing the efficacy and safety of once daily bilastine with cetirizine and placebo for the treatment of seasonal allergic rhinitis ALLERGY, 2007.	Wrong publication type		
128	RAYYAN-603244154	A comparative study of clinical efficacy and tolerability of second generation (cetirizine) and third generation (levocetirizine) antihistaminics in seasonal allergic rhinitis INDIAN JOURNAL OF PHARMACOLOGY, 2008.	Wrong publication type		
129	RAYYAN-603244218	Double blind comparison between terfenadine (120 mg/day) and loratadine (10 mg/day) in patients with seasonal allergic rhinitis DOPPELBLINDER GRUPPENVERGLEICH ZWISCHEN TERFENADIN (120 MG/TAG) UND LORATADIN (10 MG/TAG) BEI PATIENTEN MIT SAISONALER ALLERGISCHER RHINITIS, 1992.	Wrong outcome	Embase record L22338175	
130	RAYYAN-603244399	Double-blind parallel-group comparison of once a day terfenadine and loratadine in seasonal allergic rhinitis. A multicentre study ACTA THER., 1990.	Wrong outcome	Embase record L20320679	
131	RAYYAN-603244539	Terfenadine and astemizole in perennial allergic rhinitis, with emphasis to the first day of therapy FOLHA MED., 1988.	Wrong outcome	Embase record L19029292	
132	RAYYAN-603244541	Astemizole vs terfenadine in the treatment of seasonal allergic rhinitis FOLIA ALLERGOL. IMMUNOL. CLIN., 1988.	Wrong outcome	Embase record L19221720	

No.	Rayyan ID	Report citation	Primary exclusion reason	DOI / URL	Qualification
133	RAYYAN-603244547	Placebo-controlled, randomized evaluation of acrivastine in seasonal allergic rhinitis ACTA THER., 1988.	Wrong outcome	Embase record L18234052	
134	RAYYAN-603244552	Astemizole versus terfenadine in perennial allergic rhinitis FOLHA MED., 1988.	Wrong outcome	Embase record L18171594	
135	RAYYAN-603244660	A comparison of six antihistamine drugs in the treatment of perennial allergic rhinitis IMMUNOL. ALLERGY PRACT., 1986.	Wrong outcome	Embase record L16092132	
136	RAYYAN-603244679	DOUBLE-BLIND PLACEBO-CONTROLLED COMPARISON OF ASTEMIZOLE AND TERFENADINE IN SEASONAL ALLERGIC RHINITIS JOURNAL OF ALLERGY AND CLINICAL IMMUNOLOGY, 1988.	Wrong publication type	10.1016/0091-6749(88)90473-3	
137	RAYYAN-603244745	DOUBLE-BLIND COMPARISON OF THE EFFICACY AND SAFETY OF ASTEMIZOLE AND LORATADINE IN THE TREATMENT OF SEASONAL ALLERGIC RHINITIS DRUG INVESTIGATION, 1992.	Wrong publication type		
138	RAYYAN-603244747	Comparative study between mequitazine and terfenadine in allergic rhinitis ETUDE COMPARATIVE DE LA MEQUITAZINE ET DE LA TERFENADINE DANS LES RHINITES ALLERGIQUES, 1984.	Wrong outcome	Embase record L14059035	
139	RAYYAN-603244865	EFFICACY AND TOLERABILITY OF CETIRIZINE, LORATADINE AND PLACEBO IN THE TREATMENT OF SEASONAL ALLERGIC RHINITIS (SAR) - A CANADIAN MULTICENTER STUDY JOURNAL OF ALLERGY AND CLINICAL IMMUNOLOGY, 1994.	Wrong publication type		
140	RAYYAN-603244926	Mequitazine and placebo, double blind trial in outpatients ESSAI CLINIQUE EN DOUBLE AVEUGLE CONTRE PLACEBO DE LA MEQUITAZINE CHEZ DES PATIENTS NON HOSPITALISES, 1980.	Wrong population	Embase record L10062699	
141	RAYYAN-603245011	Comparative trial of two antihistamines, mequitazine and brompheniramine CURR. MED. RES. OPIN., 1978.	Wrong population	Embase record L8342409	
142	RAYYAN-603245187	Desloratacline is effective at relieving nasal congestion, as demonstrated in three placebo-controlled trials in patients with seasonal allergic rhinitis NEW TRENDS IN ALLERGY V, 2002.	Wrong publication type		
143	RAYYAN-603245232	The comparative efficacy and safety of ebastine 20 mg and 10 mg once daily (OD) and loratadine 10mg OD vs placebo during 4 week treatment of seasonal allergic rhinitis (SAR). JOURNAL OF ALLERGY AND CLINICAL IMMUNOLOGY, 1999.	Wrong publication type		
144	RAYYAN-603245351	THE EFFICACY OF ASTEMIZOLE IN THE TREATMENT OF SEASONAL ALLERGIC RHINITIS (SAR) - A DOUBLE-BLIND-STUDY ANNALS OF ALLERGY, 1987.	Wrong publication type		
145	RAYYAN-603245626	Evaluation of the efficacy and safety of levocetirizine during 8 weeks preceeding and following the anticipated onset of the grass pollen season in subjects suffering from seasonal allergic rhinitis associated with pollen-induced asthma Clinicaltrials.gov [accessed 31 jul 2008], year NR.	Wrong outcome	10.1002/central/CN-00652485/full	
146	RAYYAN-603245669	Trial to Compare the Efficacy and Safety of Levocetirizine 5 mg od and Fexofenadine 120 mg od in Reducing Symptoms of Seasonal Allergic Rhinitis in Grass Pollen Sensitized Adults Clinicaltrials.gov [www.clinicaltrials.gov], year NR.	Wrong outcome	10.1002/central/CN-01039553/full	
147	RAYYAN-603245691	A multicentre, double-blind, parallel, randomized, placebo-controlled study : evaluation of the efficacy and safety of Levocetirizine 5 mg and Desloratadine 5 mg administered orally as capsules once daily, in the morning, over 2 weeks in patients suffering from Seasonal Allergic Rhinitis (SAR) du... EUCTR [www.clinicaltrialsregister.eu], year NR.	Background article	10.1002/central/CN-01013203/full	
148	RAYYAN-603245692	A monocenter, double-blind, randomized trial, with two parallel groups comparing the clinical efficacy of levocetirizine 5 mg capsules and desloratadine 5 mg capsules taken once a day over 3 weeks of treatment in adult subjects suffering from seasonal allergic rhinitis (SAR) due to grass pollen Clinicaltrials.gov [accessed 31 jul 2008], year NR.	Wrong publication type	10.1002/central/CN-00652493/full	

No.	Rayyan ID	Report citation	Primary exclusion reason	DOI / URL	Qualification
149	RAYYAN-603246275	A double blind comparative study versus placebo of cetirizine and astemizole in hay fever treatment Revue des maladies respiratoires, 1990.	Wrong publication type	10.1002/central/CN-00335560/full	
150	RAYYAN-603246512	The efficacy and safety of loratadine versus astemizole in the treatment of perennial allergic rhinitis Siriraj Hospital gazette, 1993.	Wrong outcome	10.1002/central/CN-00614779/full	
151	RAYYAN-603246516	Comparative clinical study between cetirizine and placebo in the treatment of patients with perennial allergic rhinitis Revista brasileira medicina - otorrinolaringologia, 1997.	Wrong outcome	10.1002/central/CN-00452494/full	
152	RAYYAN-603246519	Safety and Effectiveness of Bilastine and Fexofenadine in Perennial Allergic Rhinitis patients https://trialsearch.who.int/Trial2.aspx?TrialID=CTRI/2024/07/071414, 2024.	Wrong outcome	10.1002/central/CN-02740228/full	
153	RAYYAN-603246523	An evaluation of the efficacy and safety of loratadine syrup vs. astemizole syrup in perennial allergic rhinitis Immunology and allergy practice, 1991.	Wrong outcome	10.1002/central/CN-00441086/full	
154	RAYYAN-603246600	Effect of once daily desloratadine in patients with perennial allergic rhinitis (PAR) a double blind placebo controlled trial Journal of allergy and clinical immunology, 2003.	Wrong outcome	10.1002/central/CN-00525119/full	
155	RAYYAN-603246670	A study on the efficacy and safety of a new second generation antihistamine -mizolastine, compared to cetrizine in patients of allergic rhinoconjunctivitis Indian journal of allergy asthma immunology, 2003.	Wrong outcome	10.1002/central/CN-00663335/full	
156	RAYYAN-603246672	A Clinical Trial to evaluate Efficacy and Safety of Bilastine Tablet 20 mg Vs. Levocetirizine Tablet 5mg for the Treatment of Allergic Rhinoconjunctivitishttps://trialsearch.who.int/Trial2.aspx?TrialID=CTRI/2017/06/008889, 2017.	Wrong outcome	10.1002/central/CN-01885873/full	
157	RAYYAN-603246698	Comparative investigation on two antihistaminics in seasonal allergic rhinitis Die Medizinische Welt, 1985.	Wrong outcome	10.1002/central/CN-00308963/full	
158	RAYYAN-603246853	Efficacy and Safety of Desloratadine vs. Fexofenadine 180 mg. vs. Placebo for Treating Seasonal Allergic Rhinitis (SAR) (Study P04054) https://clinicaltrials.gov/ct2/show/NCT00783146, year NR.	Wrong outcome	10.1002/central/CN-01012833/full	
159	RAYYAN-603246885	A comparison of cetirizine and loratadine in the treatment of seasonal allergic rhinitis American association of asthma, allergy & immunology (AAAAI) 56th annual meeting, march 3-8, 2000 . Journal of allergy and clinical immunology, 2000.	Wrong outcome	10.1002/central/CN-00526444/full	
160	RAYYAN-603246982	Desloratadine reduces symptoms of seasonal allergic rhinitis, including nasal congestion: results of a multicenter, placebo-controlled study XXII congress of the european academy of allergology and clinical immunology (EAACI) . Paris, france, 7-11 june, 2003, year NR.	Wrong outcome	10.1002/central/CN-00526503/full	
161	RAYYAN-603246986	Effect of fexofenadine hydrochloride sustained capsule for seasonal allergic rhinitis: a double-blind,randomized,comparative multicenter trial Journal of shandong university(health sciences), 2006.	Wrong outcome	10.1002/central/CN-00715112/full	
162	RAYYAN-603247156	Comparison of the efficacy and safety of cetirizine 10 mg. O.D. (oral tablets) and fexofenadine 120 mg O.D. (oral tablets) in reducing symptoms of seasonal allergic rhinitis European respiratory society annual congress . Madrid, spain, 9-13 october, 1999, year NR.	Wrong outcome	10.1002/central/CN-00452649/full	
163	RAYYAN-603247158	Randomised, double-blind, placebo-controlled dose-effect study of V0114 (25, 5, 75 and 10 mg) versus mequitazine 10 mg and placebo in the treatment of seasonal allergic rhinitis - EUCTR [www.clinicaltrialsregister.eu], year NR.	Wrong outcome	10.1002/central/CN-01017599/full	
164	RAYYAN-603247197	A multi-center, randomized, double-blind, placebo-controlled, parallel-A multi-center, randomized, double-blind, placebo-controlled, parallel-group study evaluating the efficacy and impact on health-related quality of life of levocetirizine 5 mg once daily given for 2 weeks in subjects 18 yr of a... Clinicaltrials.gov [accessed 31 jul 2008], year NR.	Wrong outcome	10.1002/central/CN-00652495/full	
165	RAYYAN-603247213	COMPARISON OF drug ORAL BILASTINE WITH ORAL CETIRIZINE IN ALLERGIC RHINITIS https://trialsearch.who.int/Trial2.aspx?TrialID=CTRI/2025/09/095235, 2025.	Wrong outcome	10.1002/central/CN-02921819/full	

No.	Rayyan ID	Report citation	Primary exclusion reason	DOI / URL	Qualification
166	RAYYAN-603247656	Cetirizine and astemizole in allergic rhinitis : a comparative study Indian journal of otolaryngology and head and neck surgery, 1999.	Wrong outcome	10.1002/central/CN-00452667/full	
167	RAYYAN-603247670	Comparison effect of cetirizine, loratadine and fexofenadine on allergic rhinitis https://trialsearch.who.int/Trial2.aspx?TrialID=IRCT201510136252N9, 2015.	Wrong outcome	10.1002/central/CN-01851297/full	
168	RAYYAN-603247765	Efficacy of oral bilastine in comparison with levocetirizine in allergic rhinitis- a randomised clinical trial Journal of clinical and diagnostic research, 2021.	Wrong outcome	10.7860/JCDR/2021/48303.15183	
169	RAYYAN-603247786	A clinical trial of two non-sedative antihistamines, loratadine and astemizole, for allergic rhinitis Majalah demografi Indonesia, 1991.	Wrong outcome	10.1002/central/CN-00614721/full	
170	RAYYAN-603247789	Comparative trial of astemizole and dextrochlorpheniramine in the treatment of allergic rhinitis Folha m? D, 1985.	Wrong outcome	10.1002/central/CN-00452514/full	
171	RAYYAN-603247821	Double-blind study of the efficacy and safety of desloratadine for treating allergic rhinitis Acta academiae medicinae militaris tertiae, 2003.	Wrong outcome	10.1002/central/CN-00508414/full	
172	RAYYAN-603247869	Double-Blind, Randomized, Placebo-Controlled, Parallel-Group, Multicenter/ Multinational, Efficacy and Safety Study of Desloratadine 5 mg in the Treatment of Subjects With Allergic Rhinitis Who Meet the Criteria for Persistent Allergic Rhinitis (PER) EUCTR [www.clinicaltrialsregister.eu], year NR.	Wrong outcome	10.1002/central/CN-01013355/full	
173	RAYYAN-603247902	A multicenter, randomized, comparative study on the safety and efficacy of Mizolastine versus Loratadine for the treatment of allergic rhinitis Chinese journal of microbiology and immunology, 2001.	Wrong outcome	10.1002/central/CN-00366968/full	
174	RAYYAN-603248023	Desloratadine 5mg and total nasal symptoms in patients intermittent allergic rhinitis to cypress pollen, a double-blind, placebo-controlled study XXIII congress of the european academy of allergology and clinical immunology (EAACI) . Amsterdam, the netherlands, 12-16 june, 2004, year NR.	Wrong outcome	10.1002/central/CN-00526395/full	
175	RAYYAN-603248060	A double-blind placebo controlled study to assess the safety and efficacy of cetirizine in patients with allergic rhinitis and perennial bronchial asthma Annals of allergy, asthma and immunology, 1995.	Wrong outcome	10.1002/central/CN-00278282/full	
176	RAYYAN-603248106	Double-blind, randomised, placebo-controlled, phase III study comparing the efficacy and safety of bilastine 20 mg once daily and cetirizine 10 mg for the treatment of allergic allergic rhinitis EUCTR [www.clinicaltrialsregister.eu], year NR.	Wrong outcome	10.1002/central/CN-01013270/full	
177	RAYYAN-603248108	A double blind, randomised, parallel, placebo-controlled, pilot study to evaluate the anti-inflammatory effects and the efficacy of Rupatadine 10 mg on the symptoms of persistent allergic rhinitis EUCTR [www.clinicaltrialsregister.eu], year NR.	Wrong outcome	10.1002/central/CN-01013262/full	
178	RAYYAN-603248254	The placebo (PLC) controlled effect of cetirizine 10 mg (CTZ) on rhinoconjunctivitis as objectivated by conjunctival digital imaging (CDI) and patient symptom scores Annals of allergy, asthma and immunology, 1997.	Wrong outcome	10.1002/central/CN-00281313/full	
179	RAYYAN-603248371	Efficacy, Safety and PK of Once or Twice Daily Bilastine (10 or 20 mg) Compared With Placebo in the Symptomatic Treatment of SAR Clinicaltrials.gov [www.clinicaltrials.gov], year NR.	Wrong outcome	10.1002/central/CN-00756621/full	

Note: Rows represent report-level exclusion decisions. The 179 decisions comprise 176 unique substantive exclusions and three duplicate bibliographic records corresponding to reports already retained. Abstract-only status is a source format and did not automatically replace the substantive reason. Final classifications agree with Figure 1: wrong outcome, 86; wrong publication type, 23; wrong drug, 20; background article, 17; wrong population, 14; wrong study design, 13; wrong study duration, 3; duplicate bibliographic record, 3.

Table S12C. Whole-corpus denominator and analytical-disposition reconciliation.

Level/entity	Count	Arithmetic/relationship	Interpretation/qualification
Report-level records assessed for eligibility	240	240 = 237 + 3	Historical report-level record count

Level/entity	Count	Arithmetic/relationship	Interpretation/qualification
Unique reports assessed	237	237 = 61 + 176	Bibliographically unique reports
Duplicate bibliographic records	3	240 - 237	Copies of retained E39, E55, E86; no analytical impact
Report-level exclusion decisions	179	179 = 176 + 3	176 substantive exclusions plus 3 duplicate records
Unique reports excluded	176	237 - 61	Duplicate records classified separately
Retained reports	61		Documentary reports retained
Retained report-randomization entries	66		Two are non-incremental E35 companion links
Unique non-overlapping randomized cohorts	64	66 - 2	Participant-counting layer after E35 overlap control
Randomized participants in retained cohorts	34,831	22,668 + 12,163	Deduplicated whole-corpus denominator
Analysis-valid participants	34,811	34,831 - 20	Sensitivity denominator after E45 center exclusion
Reports describing model-ready evidence	33		Linked to at least one model-ready randomization
Model-ready independent randomizations	35		One non-overlapping cohort each
Complete randomized participants in modeled trials	22,668	18,022 + 4,646	All randomized arms in 35 modeled trials
Participants in review-eligible modeled arms	18,022		Primary eligible-arm denominator
Frozen arm-level denominator	17,887	18,022 - 135	Source-adjudicated arm-level analysis-population denominator used in frozen inputs
Participants in ineligible arms in modeled trials	4,646	22,668 - 18,022	Retained for complete-trial accounting
Other retained entries	31	66 - 35	28 evidence-map cohorts + 1 contextual + 2 companion links
Unique evidence-map-only cohorts	28		Retained but not model-ready
Contextual/sensitivity-only cohort	1		E54/Prenner; N=1,095
Non-incremental E35 companion links	2	Fall 1999 -> E49; Fall 2001 -> E63	Pollen seasons represented in pooled E35 report; incremental N=0 for both
Unique non-modeled/context cohorts	29	28 + 1	Evidence-map plus contextual cohort
Participants in non-modeled/context cohorts	12,163	34,831 - 22,668	Includes E54
Participants excluding E54	11,068	12,163 - 1,095	Evidence-map-only cohorts excluding contextual cohort

Companion-link detail: the pooled E35 report represents Fall 1999, Fall 2000, and Fall 2001 pollen-season randomizations. Fall 1999 is already counted through E49/TF_E49; Fall 2001 is already counted through E63/TF_E63; only Fall 2000 is incrementally counted through TF_E35_FALL2000_MONT_LOR_PBO. The companion entries therefore add zero participants and do not create extra cohorts. The 135-person difference between 18,022 and 17,887 reflects source-adjudicated arm-level analysis-population differences documented in S13; it is not a loss from the randomized corpus.

S13. Characteristics of Model-Ready Evidence

Table S13. Characteristics and denominator provenance of the 35 model-ready randomizations.

No.	Study/randomization	Report ID	Canonical trial ID	Cat.	Eligible regimens/comparators	Full N	Eligible N	Frozen N	Primary structure(s)	Source qualification
1	Mansfield et al. (2010)	E76	TF_A00430_NCT00621959	SAR	Levocetirizine 5 mg + placebo	596	596	596	See S29 / model-input workbook	PDF p. 3: 596 randomized (301/295). Canonical reporting ID TF_A00430_NCT00621959; registry alias NCT00621959 retained in Identifier_Alias_Crosswalk_v1.1.csv.
2	Segall et al. (2010)	E66	TF_A00431_NCT00653224	SAR	Levocetirizine 5 mg + placebo	580	580	578	See S29 / model-input workbook	PDF p. 3: 580 randomized (287/293). Canonical reporting ID TF_A00431_NCT00653224; registry alias NCT00653224 retained in Identifier_Alias_Crosswalk_v1.1.csv.
3	Bachert et al. (1998)	E15	TF_E15	PAR	Mizolastine 10 mg + placebo	257	257	253	See S29 / model-input workbook	PDF p. 3: 257 randomized (133/124). Model uses safety denominators.
4	Davies et al. (1998)	E17	TF_E17	PAR	Ebastine 10 mg + ebastine 20 mg + loratadine 10 mg	317	317	317	See S29 / model-input workbook	Abstract section of full report: trial conducted in 317 randomized patients.
5	Frèche et al. (2002)	E18	TF_E18	PAR	Mizolastine 10 mg + loratadine 10 mg + placebo	428	428	425	See S29 / model-input workbook	Abstract section of full report: 428 randomized (146/141/141). Model uses safety denominators 145/140/140.
6	Holmberg et al. (2009)	E19	TF_E19	PER	Desloratadine 5 mg + placebo	584	584	584	See S29 / model-input workbook	PDF p. 3: 584 randomized (293/291).
7	Kowalski et al. (2009)	E20	TF_E20	PAR	Loratadine 10 mg + rupatadine 10/20 mg + placebo	283	283	283	See S29 / model-input workbook	PDF p. 3: 283 randomized (69/70/73/71).
8	Marmouz et al. (2011)	E21	TF_E21	PAR	Cetirizine 10 mg + rupatadine 10/20 mg + placebo	282	282	269	See S29 / model-input workbook	PDF p. 3: 282 randomized. Model uses ITT arm denominators totaling 269.
9	Mo et al. (2021)	E22	TF_E22	PAR	Bepotastine CR 20 mg OD + placebo	272	272	271	See S29 / model-input workbook	Full-report abstract/flow, p.1: 272 randomized (134/138). Controlled-release 20 mg OD; one randomized participant received no placebo.
10	Philip et al. (2007)	E25	TF_E25	PAR	Cetirizine 10 mg + placebo	1365	735	735	See S29 / model-input workbook	PDF p. 3: full trial n=1365; eligible arms n=735. Source-report and modeled-unit attribution reconciled. Fantin et al. (2008) is not the TNSS-PAR model-ready unit.
11	Simons et al. (2003)	E26	TF_E26	PAR	Desloratadine 5 mg + placebo	676	676	676	See S29 / model-input workbook	Abstract section of full report: 676 randomized.
12	Won et al. (2024)	E27	TF_E27	PAR	Bepotastine 10 mg BID + rupatadine 10 mg + placebo	330	330	327	See S29 / model-input workbook	PDF p. 4: 330 randomized. Three did not receive allocated intervention.
13	Andrews et al. (2009), FFU109045	E29	TF_E29_FFU109045	SAR	Fexofenadine 180 mg + placebo	936	624	624	See S29 / model-input workbook	Trial FFU109045: full n=936; eligible arms n=624. Canonical reporting ID TF_E29_FFU109045; source identifier FFU109045 retained as alias.

No.	Study/randomization	Report ID	Canonical trial ID	Cat.	Eligible regimens/comparators	Full N	Eligible N	Frozen N	Primary structure(s)	Source qualification
14	Andrews et al. (2009), FFU109047	E29	TF_E29_FFU109047	SAR	Fexofenadine 180 mg + placebo	680	456	456	See S29 / model-input workbook	Trial FFU109047: full n=680; eligible arms n=456. Canonical reporting ID TF_E29_FFU109047; source identifier FFU109047 retained as alias.
15	Bachert et al. (2009)	E31	TF_E31	SAR	Bilastine 20 mg + desloratadine 5 mg + placebo	721	721	720	See S29 / model-input workbook	PDF p. 4: 721 randomized. One participant did not receive study medication.
16	Bronsky et al. (1995)	E33	TF_E33	SAR	Loratadine 10 mg + placebo	874	433	433	See S29 / model-input workbook	PDF p. 3: full trial 221/217/220/216 (total 874); eligible 217+216. Combination/decongestant arms excluded.
17	Ford et al. (2015)	E38	TF_E38	SAR	Cetirizine 10 mg + matched placebo	682	341	341	See S29 / model-input workbook	Abstract section of full report: full trial n=682; eligible oral arms 170+171. Intranasal-steroid arms excluded.
18	Kuna et al. (2009)	E42	TF_E42	SAR	Bilastine 20 mg + cetirizine 10 mg + placebo	683	683	681	See S29 / model-input workbook	Abstract section/flow of full report: 683 randomized. 681 received study drug.
19	Nayak et al. (2002), Fall 1999 trial	E49	TF_E49	SAR	Loratadine 10 mg + placebo	907	450	450	See S29 / model-input workbook	Full-report abstract/table, p.1: full n=907; eligible loratadine/placebo arms n=450. Montelukast and combination arms excluded.
20	Noonan et al. (2003)	E50	TF_E50	SAR	Cetirizine 10 mg + placebo	403	403	398	See S29 / model-input workbook	Abstract section of full report: 403 randomized. Model uses evaluable/safety denominators.
21	Okubo et al. (2019)	E51	TF_E51	SAR	Rupatadine 10 mg + rupatadine 20 mg + placebo	900	900	900	See S29 / model-input workbook	Table 1: T4NSS 0-16 arm mean changes and sampling SEs; primary contrasts and SEs transformed exactly x0.75 to canonical 0-12 TNSS. No untransformed 0-16 effect entered the primary model.
22	Philip et al. (2002)	E52	TF_E52	SAR	Loratadine 10 mg + placebo	1302	954	954	See S29 / model-input workbook	Abstract section of full report: full n=1302; eligible 602+352. Montelukast arm excluded.
23	Pradalier et al. (2007)	E53	TF_E53	SAR	Desloratadine 5 mg + placebo	534	534	483	See S29 / model-input workbook	PDF p. 2: 534 randomized (264/270). ITT population n=483 after post-randomization eligibility exclusions.
24	Ratner et al. (2017), NCT01241214	E55	TF_E55_NCT01241214	SAR	Cetirizine 10 mg + placebo	579	192	192	See S29 / model-input workbook	Phase 2: full n=579; eligible arms 96+96. Canonical reporting ID TF_E55_NCT01241214; registry alias NCT01241214 retained.
25	Ratner et al. (2017), NCT01484119	E55	TF_E55_NCT01484119	SAR	Cetirizine 10 mg + placebo	630	420	420	See S29 / model-input workbook	Phase 3: full n=630; eligible arms 210+210. Canonical reporting ID TF_E55_NCT01484119; registry alias NCT01484119 retained.

No.	Study/randomization	Report ID	Canonical trial ID	Cat.	Eligible regimens/comparators	Full N	Eligible N	Frozen N	Primary structure(s)	Source qualification
26	Van Adelsberg et al. (2003a)	E62	TF_E62	SAR	Loratadine 10 mg + placebo	1214	692	692	See S29 / model-input workbook	Abstract section/table of full report: full n=1214; eligible 171+521. Montelukast arm excluded.
27	Van Adelsberg et al. (2003b)	E63	TF_E63	SAR	Loratadine 10 mg + placebo	1079	631	631	See S29 / model-input workbook	PDF p. 3 flow: full n=1079; placebo 451, montelukast 448, loratadine 180. Source flow: 1,079 randomized (451 placebo, 448 montelukast, 180 loratadine); eligible/frozen loratadine-placebo n=631. Prior 692 value was copied from E62-like Table 2 metadata. Comparative effects and standard errors unchanged.
28	Skoner et al. (2014)	E71	TF_E71	PAR	Cetirizine 10 mg + placebo	321	321	320	See S29 / model-input workbook	Abstract/Results section of full report: 158+163=321 randomized. Model placebo denominator is 162.
29	Casale et al. (1999)	E74	TF_E74	SAR	Fexofenadine 120/180 mg + placebo	864	864	863	See S29 / model-input workbook	PDF p. 3: 864 randomized. One had no post-baseline assessment.
30	Van Cauwenberge et al. (2000)	E79	TF_E79	SAR	Fexofenadine 120 mg + loratadine 10 mg + placebo	688	688	685	See S29 / model-input workbook	Abstract section of full report: 688 randomized. Model uses safety/evaluable denominator n=685.
31	Sastre et al. (2012)	E81	TF_E81	PAR	Bilastine 20 mg + cetirizine 10 mg + placebo	651	651	650	See S29 / model-input workbook	PDF pp. 4-5: 651 randomized. One did not receive medication.
32	Meltzer et al. (2006)	E84	TF_E84	SAR	Desloratadine 5 mg + placebo	220	220	219	See S29 / model-input workbook	PDF p. 3: 220 randomized (109/111). 219 received at least one dose.
33	Chervinsky et al. (2004), Study 2 (Fall 2000)	E35	TF_E35_FALL2000_MONT_LOR_PBO	SAR	Loratadine 10 mg + placebo	830	504	504	See S29 / model-input workbook	Table 1: Fall 2000 montelukast 326, placebo 333; source text gives loratadine 171. Canonical reporting ID TF_E35_FALL2000_MONT_LOR_PBO; legacy aliases retained in Identifier_Alias_Crosswalk_v1.1.csv. Fall 1999 and Fall non-incremental companion links to E49 and E63, respectively.
34	Organon and Co. (2014), NCT02320396	E140	TF_NCT02320396	SAR	Desloratadine 5 mg + placebo	449	449	448	See S29 / model-input workbook	Registry: 449 randomized (224/225). Canonical reporting ID TF_NCT02320396; registry alias NCT02320396 retained.
35	Bachert et al. (2004), XPERT	E67	TF_XPERT	PER	Levocetirizine 5 mg + placebo	551	551	509	See S29 / model-input workbook	Abstract section/table of full report: 551 randomized (278/273). Week-4 RQLQ analysis population 257+252=509.

Note: Identifier policy: canonical IDs are displayed in table cells. Legacy aliases and source trial identifiers are preserved only in Identifier_Alias_Crosswalk_v1.1.csv. Fall 1999 and Fall 2001 denote pollen seasons, not publication years.

S14. Standardized absolute-risk scenarios for binary outcomes

Table S14. Posterior absolute-risk transformations for the four binary evidence structures under standardized placebo baseline risks.

Structure	Regimen versus placebo	Standardized placebo baseline risk	Treated risk per 1000 (95% CrI)	Risk difference per 1000 (95% CrI)	Risk ratio (95% CrI)
Any AE–SAR	Bilastine 20 mg OD	1%	8.9 [5.5, 13.6]	−1.1 [−4.5, 3.6]	0.89 [0.55, 1.36]
Any AE–SAR	Bilastine 20 mg OD	5%	44.5 [27.8, 66.9]	−5.5 [−22.2, 16.9]	0.89 [0.56, 1.34]
Any AE–SAR	Bilastine 20 mg OD	10%	89.6 [56.9, 131.4]	−10.4 [−43.1, 31.4]	0.90 [0.57, 1.31]
Any AE–SAR	Bilastine 20 mg OD	20%	181.2 [119.6, 254.0]	−18.8 [−80.4, 54.0]	0.91 [0.60, 1.27]
Any AE–SAR	Cetirizine 10 mg OD	1%	11.5 [6.0, 18.0]	1.5 [−4.0, 8.0]	1.15 [0.60, 1.80]
Any AE–SAR	Cetirizine 10 mg OD	5%	57.1 [30.6, 87.2]	7.1 [−19.4, 37.2]	1.14 [0.61, 1.74]
Any AE–SAR	Cetirizine 10 mg OD	10%	113.4 [62.5, 167.8]	13.4 [−37.5, 67.8]	1.13 [0.63, 1.68]
Any AE–SAR	Cetirizine 10 mg OD	20%	223.5 [130.5, 312.2]	23.5 [−69.5, 112.2]	1.12 [0.65, 1.56]
Any AE–SAR	Desloratadine 5 mg OD	1%	12.4 [6.6, 21.9]	2.4 [−3.4, 11.9]	1.24 [0.66, 2.19]
Any AE–SAR	Desloratadine 5 mg OD	5%	61.2 [33.5, 104.4]	11.2 [−16.5, 54.4]	1.22 [0.67, 2.09]
Any AE–SAR	Desloratadine 5 mg OD	10%	120.9 [68.3, 197.5]	20.9 [−31.7, 97.5]	1.21 [0.68, 1.97]
Any AE–SAR	Desloratadine 5 mg OD	20%	236.4 [141.6, 356.3]	36.4 [−58.4, 156.3]	1.18 [0.71, 1.78]
Any AE–SAR	Fexofenadine 120 mg OD	1%	10.4 [6.7, 16.5]	0.4 [−3.3, 6.5]	1.04 [0.67, 1.65]
Any AE–SAR	Fexofenadine 120 mg OD	5%	51.9 [33.7, 80.5]	1.9 [−16.3, 30.5]	1.04 [0.67, 1.61]
Any AE–SAR	Fexofenadine 120 mg OD	10%	103.6 [68.6, 156.1]	3.6 [−31.4, 56.1]	1.04 [0.69, 1.56]
Any AE–SAR	Fexofenadine 120 mg OD	20%	206.3 [142.2, 293.8]	6.3 [−57.8, 93.8]	1.03 [0.71, 1.47]
Any AE–SAR	Fexofenadine 180 mg OD	1%	10.4 [5.8, 18.6]	0.4 [−4.2, 8.6]	1.04 [0.58, 1.86]
Any AE–SAR	Fexofenadine 180 mg OD	5%	51.8 [29.5, 89.7]	1.8 [−20.5, 39.7]	1.04 [0.59, 1.79]
Any AE–SAR	Fexofenadine 180 mg OD	10%	103.4 [60.2, 172.2]	3.4 [−39.8, 72.2]	1.03 [0.60, 1.72]
Any AE–SAR	Fexofenadine 180 mg OD	20%	206.0 [126.0, 318.8]	6.0 [−74.0, 118.8]	1.03 [0.63, 1.59]
Any AE–SAR	Levocetirizine 5 mg OD	1%	8.6 [5.3, 13.7]	−1.4 [−4.7, 3.7]	0.86 [0.53, 1.37]
Any AE–SAR	Levocetirizine 5 mg OD	5%	43.5 [27.1, 67.5]	−6.5 [−22.9, 17.5]	0.87 [0.54, 1.35]
Any AE–SAR	Levocetirizine 5 mg OD	10%	87.6 [55.5, 132.5]	−12.4 [−44.5, 32.5]	0.88 [0.55, 1.33]
Any AE–SAR	Levocetirizine 5 mg OD	20%	177.6 [116.7, 255.8]	−22.4 [−83.3, 55.8]	0.89 [0.58, 1.28]
Any AE–SAR	Loratadine 10 mg OD	1%	10.8 [6.8, 17.1]	0.8 [−3.2, 7.1]	1.08 [0.68, 1.71]
Any AE–SAR	Loratadine 10 mg OD	5%	54.0 [34.7, 83.3]	4.0 [−15.3, 33.3]	1.08 [0.69, 1.67]
Any AE–SAR	Loratadine 10 mg OD	10%	107.6 [70.5, 160.9]	7.6 [−29.5, 60.9]	1.08 [0.70, 1.61]
Any AE–SAR	Loratadine 10 mg OD	20%	213.4 [145.8, 301.5]	13.4 [−54.2, 101.5]	1.07 [0.73, 1.51]
Any AE–SAR	Rupatadine 10 mg OD	1%	21.0 [9.8, 41.7]	11.0 [−0.2, 31.7]	2.10 [0.98, 4.17]
Any AE–SAR	Rupatadine 10 mg OD	5%	100.6 [48.9, 184.8]	50.6 [−1.1, 134.8]	2.01 [0.98, 3.70]

Structure	Regimen versus placebo	Standardized placebo baseline risk	Treated risk per 1000 (95% CrI)	Risk difference per 1000 (95% CrI)	Risk ratio (95% CrI)
Any AE–SAR	Rupatadine 10 mg OD	10%	191.1 [97.9, 323.7]	91.1 [−2.1, 223.7]	1.91 [0.98, 3.24]
Any AE–SAR	Rupatadine 10 mg OD	20%	347.1 [196.3, 518.6]	147.1 [−3.7, 318.6]	1.74 [0.98, 2.59]
Any AE–SAR	Rupatadine 20 mg OD	1%	22.6 [10.7, 44.6]	12.6 [0.7, 34.6]	2.26 [1.07, 4.46]
Any AE–SAR	Rupatadine 20 mg OD	5%	107.6 [53.6, 195.7]	57.6 [3.6, 145.7]	2.15 [1.07, 3.91]
Any AE–SAR	Rupatadine 20 mg OD	10%	202.9 [106.7, 339.3]	102.9 [6.7, 239.3]	2.03 [1.07, 3.39]
Any AE–SAR	Rupatadine 20 mg OD	20%	364.2 [211.9, 536.1]	164.2 [11.9, 336.1]	1.82 [1.06, 2.68]
Any AE–PAR	Bepotastine 10 mg BID	1%	15.8 [7.0, 34.2]	5.8 [−3.0, 24.2]	1.58 [0.70, 3.42]
Any AE–PAR	Bepotastine 10 mg BID	5%	77.2 [35.7, 155.9]	27.2 [−14.3, 105.9]	1.54 [0.71, 3.12]
Any AE–PAR	Bepotastine 10 mg BID	10%	150.1 [72.4, 280.6]	50.1 [−27.6, 180.6]	1.50 [0.72, 2.81]
Any AE–PAR	Bepotastine 10 mg BID	20%	284.4 [149.4, 467.4]	84.4 [−50.6, 267.4]	1.42 [0.75, 2.34]
Any AE–PAR	Bepotastine CR 20 mg OD	1%	16.4 [7.1, 37.6]	6.4 [−2.9, 27.6]	1.64 [0.71, 3.76]
Any AE–PAR	Bepotastine CR 20 mg OD	5%	80.1 [35.9, 169.1]	30.1 [−14.1, 119.1]	1.60 [0.72, 3.38]
Any AE–PAR	Bepotastine CR 20 mg OD	10%	155.3 [72.9, 300.5]	55.3 [−27.1, 200.5]	1.55 [0.73, 3.01]
Any AE–PAR	Bepotastine CR 20 mg OD	20%	292.6 [150.4, 491.5]	92.6 [−49.6, 291.5]	1.46 [0.75, 2.46]
Any AE–PAR	Bilastine 20 mg OD	1%	10.2 [5.5, 19.4]	0.2 [−4.5, 9.4]	1.02 [0.55, 1.94]
Any AE–PAR	Bilastine 20 mg OD	5%	51.2 [28.0, 93.3]	1.2 [−22.0, 43.3]	1.02 [0.56, 1.87]
Any AE–PAR	Bilastine 20 mg OD	10%	102.3 [57.4, 178.5]	2.3 [−42.6, 78.5]	1.02 [0.57, 1.79]
Any AE–PAR	Bilastine 20 mg OD	20%	204.0 [120.4, 328.4]	4.0 [−79.6, 128.4]	1.02 [0.60, 1.64]
Any AE–PAR	Cetirizine 10 mg OD	1%	8.0 [4.9, 13.6]	−2.0 [−5.1, 3.6]	0.80 [0.49, 1.36]
Any AE–PAR	Cetirizine 10 mg OD	5%	40.2 [25.0, 67.0]	−9.8 [−25.0, 17.0]	0.80 [0.50, 1.34]
Any AE–PAR	Cetirizine 10 mg OD	10%	81.2 [51.4, 131.7]	−18.8 [−48.6, 31.7]	0.81 [0.51, 1.32]
Any AE–PAR	Cetirizine 10 mg OD	20%	165.9 [108.7, 254.5]	−34.1 [−91.3, 54.5]	0.83 [0.54, 1.27]
Any AE–PAR	Desloratadine 5 mg OD	1%	7.6 [4.0, 15.0]	−2.4 [−6.0, 5.0]	0.76 [0.40, 1.50]
Any AE–PAR	Desloratadine 5 mg OD	5%	38.4 [20.4, 73.3]	−11.6 [−29.6, 23.3]	0.77 [0.41, 1.47]
Any AE–PAR	Desloratadine 5 mg OD	10%	77.7 [42.0, 143.2]	−22.3 [−58.0, 43.2]	0.78 [0.42, 1.43]
Any AE–PAR	Desloratadine 5 mg OD	20%	159.4 [89.8, 273.2]	−40.6 [−110.2, 73.2]	0.80 [0.45, 1.37]
Any AE–PAR	Ebastine 10 mg OD	1%	6.9 [2.8, 18.4]	−3.1 [−7.2, 8.4]	0.69 [0.28, 1.84]
Any AE–PAR	Ebastine 10 mg OD	5%	34.7 [14.2, 89.0]	−15.3 [−35.8, 39.0]	0.69 [0.28, 1.78]
Any AE–PAR	Ebastine 10 mg OD	10%	70.6 [29.6, 171.0]	−29.4 [−70.4, 71.0]	0.71 [0.30, 1.71]
Any AE–PAR	Ebastine 10 mg OD	20%	145.9 [64.1, 317.0]	−54.1 [−135.9, 117.0]	0.73 [0.32, 1.59]
Any AE–PAR	Ebastine 20 mg OD	1%	5.4 [2.2, 15.0]	−4.6 [−7.8, 5.0]	0.54 [0.22, 1.50]
Any AE–PAR	Ebastine 20 mg OD	5%	27.6 [11.3, 73.3]	−22.4 [−38.7, 23.3]	0.55 [0.23, 1.47]
Any AE–PAR	Ebastine 20 mg OD	10%	56.6 [23.5, 143.1]	−43.4 [−76.5, 43.1]	0.57 [0.23, 1.43]

Structure	Regimen versus placebo	Standardized placebo baseline risk	Treated risk per 1000 (95% CrI)	Risk difference per 1000 (95% CrI)	Risk ratio (95% CrI)
Any AE–PAR	Ebastine 20 mg OD	20%	118.9 [51.3, 273.2]	−81.1 [−148.7, 73.2]	0.59 [0.26, 1.37]
Any AE–PAR	Loratadine 10 mg OD	1%	7.1 [3.8, 13.9]	−2.9 [−6.2, 3.9]	0.71 [0.38, 1.39]
Any AE–PAR	Loratadine 10 mg OD	5%	36.1 [19.5, 68.5]	−13.9 [−30.5, 18.5]	0.72 [0.39, 1.37]
Any AE–PAR	Loratadine 10 mg OD	10%	73.2 [40.3, 134.4]	−26.8 [−59.7, 34.4]	0.73 [0.40, 1.34]
Any AE–PAR	Loratadine 10 mg OD	20%	150.8 [86.4, 258.9]	−49.2 [−113.6, 58.9]	0.75 [0.43, 1.29]
Any AE–PAR	Mizolastine 10 mg OD	1%	8.6 [5.1, 14.6]	−1.4 [−4.9, 4.6]	0.86 [0.51, 1.46]
Any AE–PAR	Mizolastine 10 mg OD	5%	43.4 [26.1, 71.8]	−6.6 [−23.9, 21.8]	0.87 [0.52, 1.44]
Any AE–PAR	Mizolastine 10 mg OD	10%	87.3 [53.5, 140.4]	−12.7 [−46.5, 40.4]	0.87 [0.54, 1.40]
Any AE–PAR	Mizolastine 10 mg OD	20%	177.1 [112.9, 268.7]	−22.9 [−87.1, 68.7]	0.89 [0.56, 1.34]
Any AE–PAR	Rupatadine 10 mg OD	1%	14.0 [6.3, 30.7]	4.0 [−3.7, 20.7]	1.40 [0.63, 3.07]
Any AE–PAR	Rupatadine 10 mg OD	5%	68.8 [32.1, 141.8]	18.8 [−17.9, 91.8]	1.38 [0.64, 2.84]
Any AE–PAR	Rupatadine 10 mg OD	10%	134.9 [65.4, 258.5]	34.9 [−34.6, 158.5]	1.35 [0.65, 2.59]
Any AE–PAR	Rupatadine 10 mg OD	20%	259.7 [136.0, 439.6]	59.7 [−64.0, 239.6]	1.30 [0.68, 2.20]
WDAE–SAR	Bilastine 20 mg OD	1%	3.4 [0.8, 12.7]	−6.6 [−9.2, 2.7]	0.34 [0.08, 1.27]
WDAE–SAR	Bilastine 20 mg OD	5%	17.6 [4.0, 62.8]	−32.4 [−46.0, 12.8]	0.35 [0.08, 1.26]
WDAE–SAR	Bilastine 20 mg OD	10%	36.5 [8.3, 123.9]	−63.5 [−91.7, 23.9]	0.37 [0.08, 1.24]
WDAE–SAR	Bilastine 20 mg OD	20%	78.6 [18.5, 241.3]	−121.4 [−181.5, 41.3]	0.39 [0.09, 1.21]
WDAE–SAR	Cetirizine 10 mg OD	1%	2.5 [0.8, 6.6]	−7.5 [−9.2, −3.4]	0.25 [0.08, 0.66]
WDAE–SAR	Cetirizine 10 mg OD	5%	12.8 [4.3, 33.4]	−37.2 [−45.7, −16.6]	0.26 [0.09, 0.67]
WDAE–SAR	Cetirizine 10 mg OD	10%	26.7 [8.9, 68.1]	−73.3 [−91.1, −31.9]	0.27 [0.09, 0.68]
WDAE–SAR	Cetirizine 10 mg OD	20%	58.2 [19.9, 141.1]	−141.8 [−180.1, −58.9]	0.29 [0.10, 0.71]
WDAE–SAR	Desloratadine 5 mg OD	1%	7.4 [2.6, 20.9]	−2.6 [−7.4, 10.9]	0.74 [0.26, 2.09]
WDAE–SAR	Desloratadine 5 mg OD	5%	37.5 [13.3, 100.2]	−12.5 [−36.7, 50.2]	0.75 [0.27, 2.00]
WDAE–SAR	Desloratadine 5 mg OD	10%	76.0 [27.6, 190.4]	−24.0 [−72.4, 90.4]	0.76 [0.28, 1.90]
WDAE–SAR	Desloratadine 5 mg OD	20%	156.2 [60.1, 346.1]	−43.8 [−139.9, 146.1]	0.78 [0.30, 1.73]
WDAE–SAR	Fexofenadine 120 mg OD	1%	5.3 [1.3, 18.9]	−4.7 [−8.7, 8.9]	0.53 [0.13, 1.89]
WDAE–SAR	Fexofenadine 120 mg OD	5%	26.9 [6.9, 91.3]	−23.1 [−43.1, 41.3]	0.54 [0.14, 1.83]
WDAE–SAR	Fexofenadine 120 mg OD	10%	55.1 [14.4, 174.9]	−44.9 [−85.6, 74.9]	0.55 [0.14, 1.75]
WDAE–SAR	Fexofenadine 120 mg OD	20%	115.9 [31.8, 323.0]	−84.1 [−168.2, 123.0]	0.58 [0.16, 1.61]
WDAE–SAR	Fexofenadine 180 mg OD	1%	16.8 [5.0, 57.4]	6.8 [−5.0, 47.4]	1.68 [0.50, 5.74]
WDAE–SAR	Fexofenadine 180 mg OD	5%	81.9 [25.4, 240.8]	31.9 [−24.6, 190.8]	1.64 [0.51, 4.82]
WDAE–SAR	Fexofenadine 180 mg OD	10%	158.4 [52.2, 401.0]	58.4 [−47.8, 301.0]	1.58 [0.52, 4.01]
WDAE–SAR	Fexofenadine 180 mg OD	20%	297.6 [110.3, 601.0]	97.6 [−89.7, 401.0]	1.49 [0.55, 3.01]

Structure	Regimen versus placebo	Standardized placebo baseline risk	Treated risk per 1000 (95% CrI)	Risk difference per 1000 (95% CrI)	Risk ratio (95% CrI)
WDAE–SAR	Levocetirizine 5 mg OD	1%	4.6 [1.2, 15.6]	−5.4 [−8.8, 5.6]	0.46 [0.12, 1.56]
WDAE–SAR	Levocetirizine 5 mg OD	5%	23.6 [6.0, 76.3]	−26.4 [−44.0, 26.3]	0.47 [0.12, 1.53]
WDAE–SAR	Levocetirizine 5 mg OD	10%	48.5 [12.5, 148.5]	−51.5 [−87.5, 48.5]	0.49 [0.13, 1.48]
WDAE–SAR	Levocetirizine 5 mg OD	20%	103.0 [27.8, 281.8]	−97.0 [−172.2, 81.8]	0.51 [0.14, 1.41]
WDAE–SAR	Loratadine 10 mg OD	1%	10.7 [4.7, 23.8]	0.7 [−5.3, 13.8]	1.07 [0.47, 2.38]
WDAE–SAR	Loratadine 10 mg OD	5%	53.3 [24.2, 112.6]	3.3 [−25.8, 62.6]	1.07 [0.48, 2.25]
WDAE–SAR	Loratadine 10 mg OD	10%	106.1 [49.7, 211.2]	6.1 [−50.3, 111.2]	1.06 [0.50, 2.11]
WDAE–SAR	Loratadine 10 mg OD	20%	210.8 [105.4, 376.0]	10.8 [−94.6, 176.0]	1.05 [0.53, 1.88]
WDAE–PAR	Bepotastine 10 mg BID	1%	6.7 [0.8, 43.5]	−3.3 [−9.2, 33.5]	0.67 [0.08, 4.35]
WDAE–PAR	Bepotastine 10 mg BID	5%	33.8 [4.3, 191.7]	−16.2 [−45.7, 141.7]	0.68 [0.09, 3.83]
WDAE–PAR	Bepotastine 10 mg BID	10%	68.7 [9.0, 333.7]	−31.3 [−91.0, 233.7]	0.69 [0.09, 3.34]
WDAE–PAR	Bepotastine 10 mg BID	20%	142.4 [20.1, 529.8]	−57.6 [−179.9, 329.8]	0.71 [0.10, 2.65]
WDAE–PAR	Bilastine 20 mg OD	1%	11.9 [2.1, 58.4]	1.9 [−7.9, 48.4]	1.19 [0.21, 5.84]
WDAE–PAR	Bilastine 20 mg OD	5%	59.3 [10.9, 244.4]	9.3 [−39.1, 194.4]	1.19 [0.22, 4.89]
WDAE–PAR	Bilastine 20 mg OD	10%	117.4 [22.7, 405.7]	17.4 [−77.3, 305.7]	1.17 [0.23, 4.06]
WDAE–PAR	Bilastine 20 mg OD	20%	230.3 [49.7, 605.7]	30.3 [−150.3, 405.7]	1.15 [0.25, 3.03]
WDAE–PAR	Cetirizine 10 mg OD	1%	7.8 [2.1, 27.1]	−2.2 [−7.9, 17.1]	0.78 [0.21, 2.71]
WDAE–PAR	Cetirizine 10 mg OD	5%	39.2 [11.0, 126.7]	−10.8 [−39.0, 76.7]	0.78 [0.22, 2.53]
WDAE–PAR	Cetirizine 10 mg OD	10%	79.2 [23.0, 234.5]	−20.8 [−77.0, 134.5]	0.79 [0.23, 2.35]
WDAE–PAR	Cetirizine 10 mg OD	20%	162.2 [50.3, 408.0]	−37.8 [−149.7, 208.0]	0.81 [0.25, 2.04]
WDAE–PAR	Desloratadine 5 mg OD	1%	14.5 [4.7, 44.1]	4.5 [−5.3, 34.1]	1.45 [0.47, 4.41]
WDAE–PAR	Desloratadine 5 mg OD	5%	71.2 [23.8, 193.9]	21.2 [−26.2, 143.9]	1.42 [0.48, 3.88]
WDAE–PAR	Desloratadine 5 mg OD	10%	139.3 [48.9, 336.7]	39.3 [−51.1, 236.7]	1.39 [0.49, 3.37]
WDAE–PAR	Desloratadine 5 mg OD	20%	266.9 [103.7, 533.2]	66.9 [−96.3, 333.2]	1.33 [0.52, 2.67]
WDAE–PAR	Ebastine 10 mg OD	1%	15.0 [2.1, 95.5]	5.0 [−7.9, 85.5]	1.50 [0.21, 9.55]
WDAE–PAR	Ebastine 10 mg OD	5%	73.4 [11.1, 354.8]	23.4 [−38.9, 304.8]	1.47 [0.22, 7.10]
WDAE–PAR	Ebastine 10 mg OD	10%	143.3 [23.1, 537.2]	43.3 [−76.9, 437.2]	1.43 [0.23, 5.37]
WDAE–PAR	Ebastine 10 mg OD	20%	273.5 [50.5, 723.1]	73.5 [−149.5, 523.1]	1.37 [0.25, 3.62]
WDAE–PAR	Ebastine 20 mg OD	1%	3.5 [0.3, 29.3]	−6.5 [−9.7, 19.3]	0.35 [0.03, 2.93]
WDAE–PAR	Ebastine 20 mg OD	5%	18.0 [1.6, 136.0]	−32.0 [−48.4, 86.0]	0.36 [0.03, 2.72]
WDAE–PAR	Ebastine 20 mg OD	10%	37.2 [3.5, 249.5]	−62.8 [−96.5, 149.5]	0.37 [0.03, 2.49]
WDAE–PAR	Ebastine 20 mg OD	20%	79.9 [7.8, 427.9]	−120.1 [−192.2, 227.9]	0.40 [0.04, 2.14]
WDAE–PAR	Loratadine 10 mg OD	1%	16.8 [5.6, 48.6]	6.8 [−4.4, 38.6]	1.68 [0.56, 4.86]

Structure	Regimen versus placebo	Standardized placebo baseline risk	Treated risk per 1000 (95% CrI)	Risk difference per 1000 (95% CrI)	Risk ratio (95% CrI)
WDAE–PAR	Loratadine 10 mg OD	5%	81.6 [28.3, 210.3]	31.6 [−21.7, 160.3]	1.63 [0.57, 4.21]
WDAE–PAR	Loratadine 10 mg OD	10%	158.0 [58.0, 359.9]	58.0 [−42.0, 259.9]	1.58 [0.58, 3.60]
WDAE–PAR	Loratadine 10 mg OD	20%	296.8 [121.6, 558.5]	96.8 [−78.4, 358.5]	1.48 [0.61, 2.79]
WDAE–PAR	Mizolastine 10 mg OD	1%	7.4 [2.3, 21.9]	−2.6 [−7.7, 11.9]	0.74 [0.23, 2.19]
WDAE–PAR	Mizolastine 10 mg OD	5%	37.2 [12.0, 104.6]	−12.8 [−38.0, 54.6]	0.74 [0.24, 2.09]
WDAE–PAR	Mizolastine 10 mg OD	10%	75.3 [25.0, 197.8]	−24.7 [−75.0, 97.8]	0.75 [0.25, 1.98]
WDAE–PAR	Mizolastine 10 mg OD	20%	154.9 [54.5, 356.8]	−45.1 [−145.5, 156.8]	0.77 [0.27, 1.78]
WDAE–PAR	Rupatadine 10 mg OD	1%	4.4 [0.7, 21.6]	−5.6 [−9.3, 11.6]	0.44 [0.07, 2.16]
WDAE–PAR	Rupatadine 10 mg OD	5%	22.3 [3.4, 103.1]	−27.7 [−46.6, 53.1]	0.45 [0.07, 2.06]
WDAE–PAR	Rupatadine 10 mg OD	10%	46.0 [7.2, 195.3]	−54.0 [−92.8, 95.3]	0.46 [0.07, 1.95]
WDAE–PAR	Rupatadine 10 mg OD	20%	97.8 [16.1, 353.2]	−102.2 [−183.9, 153.2]	0.49 [0.08, 1.77]
WDAE–PAR	Rupatadine 20 mg OD	1%	8.4 [1.1, 51.4]	−1.6 [−8.9, 41.4]	0.84 [0.11, 5.14]
WDAE–PAR	Rupatadine 20 mg OD	5%	42.1 [5.6, 220.1]	−7.9 [−44.4, 170.1]	0.84 [0.11, 4.40]
WDAE–PAR	Rupatadine 20 mg OD	10%	85.0 [11.7, 373.3]	−15.0 [−88.3, 273.3]	0.85 [0.12, 3.73]
WDAE–PAR	Rupatadine 20 mg OD	20%	172.8 [25.9, 572.7]	−27.2 [−174.1, 372.7]	0.86 [0.13, 2.86]

Note: The placebo baseline risks (1%, 5%, 10%, and 20%) are fixed standardized scenarios rather than observed or pooled event risks. For each posterior draw of the placebo-referenced odds ratio (OR), treated risk was calculated as $p_1 = OR \cdot p_0/(1 - p_0 + OR \cdot p_0)$; risk difference was $1000 \cdot (p_1 - p_0)$, and risk ratio was p_1/p_0. Values use the primary half-normal heterogeneity prior for τ with scale 0.5 and summarize uncertainty in the average relative effect; they are not prediction intervals for a future setting. Positive risk differences indicate more participants experiencing the adverse outcome with the active regimen, and negative values indicate fewer. The 144 rows represent 36 estimable regimen–structure effects evaluated at four baseline risks. Rupatadine 10 mg and 20 mg in WDAE–SAR are omitted because their effects were non-estimable and therefore received no absolute-risk transformation. Values are rounded for presentation; results/binary_absolute_risks.csv is controlling.

S15. Primary continuous comparative effects

Table S15. Primary continuous placebo-referenced effects.

Structure	Regimen vs placebo	MD	95% CI	95% prediction interval
RQLQ-PER	Desloratadine 5 mg OD	-0.30	-0.49 to -0.11	—a
RQLQ-PER	Levocetirizine 5 mg OD	-0.48	-0.67 to -0.29	—a
RQLQ-SAR	Bilastine 20 mg OD	-0.34	-0.55 to -0.14	—b
RQLQ-SAR	Desloratadine 5 mg OD	-0.38	-0.55 to -0.22	—b
RQLQ-SAR	Levocetirizine 5 mg OD	-0.17	-0.32 to -0.03	—b
RQLQ-SAR	Loratadine 10 mg OD	-0.31	-0.44 to -0.18	—b
TNSS-PAR	Cetirizine 10 mg OD	-0.50	-0.87 to -0.12	-0.93 to -0.07
TNSS-PAR	Rupatadine 10 mg OD	-0.80	-1.53 to -0.07	-1.56 to -0.04
TNSS-PAR	Rupatadine 20 mg OD	-1.06	-1.83 to -0.30	-1.85 to -0.27
TNSS-SAR	Bilastine 20 mg OD	-0.93	-1.43 to -0.43	-1.52 to -0.34
TNSS-SAR	Cetirizine 10 mg OD	-1.17	-1.50 to -0.83	-1.63 to -0.71
TNSS-SAR	Desloratadine 5 mg OD	-0.76	-1.10 to -0.41	-1.23 to -0.29
TNSS-SAR	Fexofenadine 180 mg OD	-0.07	-0.43 to 0.29	-0.55 to 0.41
TNSS-SAR	Loratadine 10 mg OD	-0.91	-1.15 to -0.67	-1.31 to -0.51
TNSS-SAR	Rupatadine 10 mg OD	-0.81	-1.25 to -0.37	-1.36 to -0.27
TNSS-SAR	Rupatadine 20 mg OD	-1.13	-1.58 to -0.68	-1.68 to -0.58
TOSS-SAR	Bilastine 20 mg OD	-0.74	-1.18 to -0.30	-1.25 to -0.23
TOSS-SAR	Cetirizine 10 mg OD	-0.85	-1.30 to -0.40	-1.37 to -0.33
TOSS-SAR	Fexofenadine 180 mg OD	-0.07	-0.36 to 0.23	-0.46 to 0.32

Notes: [a] RQLQ–PER was saturated under the consistency model; heterogeneity and prediction intervals were not estimable. [b] For RQLQ–SAR, the REML estimate of τ was at the boundary of zero and was not considered informatively estimated; a plug-in prediction interval was therefore not reported. MD, mean difference; CI, confidence interval.

S16. Primary binary effects and prediction intervals

Table S16. Primary binary placebo-referenced effects and predictive uncertainty.

Structure	Regimen vs placebo	OR	95% CrI	95% prediction interval	Null classification
ANY-AE-PAR	Bepotastine 10 mg BID	1.59	0.70 to 3.51	0.57 to 4.35	includes 1
ANY-AE-PAR	Bepotastine CR 20 mg OD	1.65	0.71 to 3.87	0.57 to 4.64	includes 1
ANY-AE-PAR	Bilastine 20 mg OD	1.03	0.55 to 1.96	0.42 to 2.58	includes 1
ANY-AE-PAR	Cetirizine 10 mg OD	0.80	0.49 to 1.37	0.36 to 1.82	includes 1
ANY-AE-PAR	Desloratadine 5 mg OD	0.76	0.39 to 1.50	0.31 to 1.92	includes 1
ANY-AE-PAR	Ebastine 10 mg OD	0.68	0.27 to 1.86	0.23 to 2.24	includes 1
ANY-AE-PAR	Ebastine 20 mg OD	0.54	0.22 to 1.50	0.18 to 1.83	includes 1
ANY-AE-PAR	Loratadine 10 mg OD	0.71	0.38 to 1.40	0.30 to 1.83	includes 1
ANY-AE-PAR	Mizolastine 10 mg OD	0.86	0.51 to 1.47	0.38 to 1.97	includes 1
ANY-AE-PAR	Rupatadine 10 mg OD	1.40	0.63 to 3.14	0.50 to 3.76	includes 1
ANY-AE-SAR	Bilastine 20 mg OD	0.89	0.54 to 1.36	0.40 to 1.79	includes 1
ANY-AE-SAR	Cetirizine 10 mg OD	1.15	0.60 to 1.82	0.47 to 2.24	includes 1
ANY-AE-SAR	Desloratadine 5 mg OD	1.24	0.66 to 2.21	0.52 to 2.77	includes 1
ANY-AE-SAR	Fexofenadine 120 mg OD	1.04	0.66 to 1.66	0.51 to 2.27	includes 1
ANY-AE-SAR	Fexofenadine 180 mg OD	1.04	0.58 to 1.87	0.45 to 2.38	includes 1
ANY-AE-SAR	Levocetirizine 5 mg OD	0.86	0.53 to 1.38	0.41 to 1.81	includes 1
ANY-AE-SAR	Loratadine 10 mg OD	1.09	0.68 to 1.73	0.53 to 2.26	includes 1
ANY-AE-SAR	Rupatadine 10 mg OD	2.13	0.98 to 4.31	0.80 to 5.14	includes 1
ANY-AE-SAR	Rupatadine 20 mg OD	2.29	1.08 to 4.62	0.88 to 5.52	OR>1
WDAE-PAR	Bepotastine 10 mg BID	0.66	0.08 to 4.51	0.07 to 5.33	includes 1
WDAE-PAR	Bilastine 20 mg OD	1.20	0.21 to 6.14	0.18 to 7.37	includes 1
WDAE-PAR	Cetirizine 10 mg OD	0.77	0.21 to 2.76	0.17 to 3.51	includes 1
WDAE-PAR	Desloratadine 5 mg OD	1.46	0.46 to 4.57	0.35 to 5.88	includes 1
WDAE-PAR	Ebastine 10 mg OD	1.51	0.21 to 10.45	0.18 to 12.56	includes 1
WDAE-PAR	Ebastine 20 mg OD	0.35	0.03 to 2.99	0.03 to 3.51	includes 1
WDAE-PAR	Loratadine 10 mg OD	1.69	0.55 to 5.06	0.43 to 6.46	includes 1
WDAE-PAR	Mizolastine 10 mg OD	0.73	0.23 to 2.22	0.18 to 3.03	includes 1
WDAE-PAR	Rupatadine 10 mg OD	0.43	0.07 to 2.18	0.05 to 2.68	includes 1
WDAE-PAR	Rupatadine 20 mg OD	0.84	0.11 to 5.36	0.09 to 6.34	includes 1
WDAE-SAR	Bilastine 20 mg OD	0.34	0.08 to 1.27	0.06 to 1.60	includes 1
WDAE-SAR	Cetirizine 10 mg OD	0.25	0.08 to 0.66	0.06 to 0.89	OR<1

Structure	Regimen vs placebo	OR	95% CrI	95% prediction interval	Null classification
WDAE-SAR	Desloratadine 5 mg OD	0.74	0.26 to 2.12	0.20 to 2.84	includes 1
WDAE-SAR	Fexofenadine 120 mg OD	0.52	0.13 to 1.91	0.11 to 2.45	includes 1
WDAE-SAR	Fexofenadine 180 mg OD	1.69	0.50 to 6.03	0.38 to 7.39	includes 1
WDAE-SAR	Levocetirizine 5 mg OD	0.46	0.11 to 1.57	0.09 to 2.00	includes 1
WDAE-SAR	Loratadine 10 mg OD	1.07	0.47 to 2.41	0.34 to 3.41	includes 1

Note: Rupatadine 10 mg and 20 mg are absent from the WDAE-SAR likelihood table because their only contributing unit had zero withdrawals in every arm; they were structurally present but non-estimable.

S17. Binary heterogeneity and diagnostic status

Table S17. Binary heterogeneity estimates and sampling status.

Structure	Model units	Model arms	Median tau	95% CrI	Sampling status
Any AE-PAR	8	20	0.202	0.012 to 0.656	PASS
Any AE-SAR	9	23	0.182	0.010 to 0.606	PASS
WDAE-PAR	8	22	0.272	0.012 to 0.903	PASS
WDAE-SAR	16	35	0.271	0.013 to 0.862	PASS

S18. Continuous sensitivity and exploratory effect estimates

Table S18. Continuous sensitivity and exploratory estimates.

Structure	Scenario	Regimen vs placebo	MD	95% CI
TNSS-PAR	Combined Bachert et al. (1998), Kowalski et al. (2009), and Mo et al. (2021) expansion	Bepotastine CR 20 mg OD	-0.76	-1.30 to -0.21
TNSS-PAR	Combined Bachert et al. (1998), Kowalski et al. (2009), and Mo et al. (2021) expansion	Cetirizine 10 mg OD	-0.49	-0.81 to -0.16
TNSS-PAR	Combined Bachert et al. (1998), Kowalski et al. (2009), and Mo et al. (2021) expansion	Loratadine 10 mg OD	-0.98	-1.56 to -0.40
TNSS-PAR	Combined Bachert et al. (1998), Kowalski et al. (2009), and Mo et al. (2021) expansion	Mizolastine 10 mg OD	-1.17	-1.73 to -0.61
TNSS-PAR	Combined Bachert et al. (1998), Kowalski et al. (2009), and Mo et al. (2021) expansion	Rupatadine 10 mg OD	-0.91	-1.35 to -0.47
TNSS-PAR	Combined Bachert et al. (1998), Kowalski et al. (2009), and Mo et al. (2021) expansion	Rupatadine 20 mg OD	-1.03	-1.48 to -0.58
TNSS-PAR	Bachert et al. (1998) scale/rater compatibility	Cetirizine 10 mg OD	-0.50	-0.87 to -0.12
TNSS-PAR	Bachert et al. (1998) scale/rater compatibility	Mizolastine 10 mg OD	-1.17	-1.77 to -0.57
TNSS-PAR	Bachert et al. (1998) scale/rater compatibility	Rupatadine 10 mg OD	-0.80	-1.53 to -0.07
TNSS-PAR	Bachert et al. (1998) scale/rater compatibility	Rupatadine 20 mg OD	-1.06	-1.83 to -0.30
TNSS-PAR	Kowalski et al. (2009), covariance approximated as zero	Cetirizine 10 mg OD	-0.49	-0.81 to -0.16
TNSS-PAR	Kowalski et al. (2009), covariance approximated as zero	Loratadine 10 mg OD	-0.98	-1.56 to -0.40
TNSS-PAR	Kowalski et al. (2009), covariance approximated as zero	Rupatadine 10 mg OD	-0.91	-1.35 to -0.47
TNSS-PAR	Kowalski et al. (2009), covariance approximated as zero	Rupatadine 20 mg OD	-1.03	-1.48 to -0.58
TNSS-PAR	Mo et al. (2021) per-protocol estimand	Bepotastine CR 20 mg OD	-0.76	-1.34 to -0.17
TNSS-PAR	Mo et al. (2021) per-protocol estimand	Cetirizine 10 mg OD	-0.50	-0.87 to -0.12
TNSS-PAR	Mo et al. (2021) per-protocol estimand	Rupatadine 10 mg OD	-0.80	-1.53 to -0.07
TNSS-PAR	Mo et al. (2021) per-protocol estimand	Rupatadine 20 mg OD	-1.06	-1.83 to -0.30
TNSS-SAR	Ratner et al. (2017) mixed-age phase 3 result	Bilastine 20 mg OD	-0.89	-1.37 to -0.41
TNSS-SAR	Ratner et al. (2017) mixed-age phase 3 result	Cetirizine 10 mg OD	-1.09	-1.36 to -0.82
TNSS-SAR	Ratner et al. (2017) mixed-age phase 3 result	Desloratadine 5 mg OD	-0.75	-1.09 to -0.42
TNSS-SAR	Ratner et al. (2017) mixed-age phase 3 result	Fexofenadine 180 mg OD	-0.07	-0.43 to 0.28
TNSS-SAR	Ratner et al. (2017) mixed-age phase 3 result	Loratadine 10 mg OD	-0.91	-1.14 to -0.68
TNSS-SAR	Ratner et al. (2017) mixed-age phase 3 result	Rupatadine 10 mg OD	-0.81	-1.24 to -0.38
TNSS-SAR	Ratner et al. (2017) mixed-age phase 3 result	Rupatadine 20 mg OD	-1.13	-1.57 to -0.69

Note: Four individual evidence-expansion analyses evaluated mixed-age eligibility using Ratner et al. (2017), broader scale/rater compatibility using Bachert et al. (1998), a per-protocol population using Mo et al. (2021), and inclusion of Kowalski et al. (2009), for which unreported covariance among adjusted contrasts was approximated as zero. The combined TNSS–PAR scenario was a separate stress test that jointly applied the Bachert, Kowalski, and Mo expansion assumptions; it was not counted as a fifth individual evidence-expansion analysis. All scenarios were exploratory and were not interpreted as equivalent robustness analyses of the

S19. WDAE-SAR source and prior sensitivities

Table S19. WDAE-SAR source and heterogeneity-prior sensitivity.

Scenario	Regimen(s)	Effect summary	Interval/classification	Interpretation
Primary heterogeneity prior, scale 0.5	Cetirizine 10 mg	0.25	0.08 to 0.66	0.06 to 0.89; both intervals below 1
Wider heterogeneity prior, scale 1.0	Cetirizine 10 mg	0.25	0.08 to 0.69	0.05 to 1.10; prediction interval includes 1
Bronsky et al. (1995) alternative placebo value	Seven estimable regimens	Max ∣delta median log-OR∣ 0.139	No class changes	No primary conclusion changed
Philip et al. (2002) alternative event definition	Seven estimable regimens	Max ∣delta median log-OR∣ 0.048	No class changes	No primary conclusion changed
Ratner et al. (2017) run-out-inclusive event definition	Seven estimable regimens	Max ∣delta median log-OR∣ 0.167	No class changes	No primary conclusion changed
Meltzer et al. (2006) alternative denominator	Seven estimable regimens	Max ∣delta median log-OR∣ 0.023	No class changes	No primary conclusion changed

S20. Rupatadine Any AE-SAR Null-Boundary Audit

Table S20. Rupatadine Any AE-SAR null-boundary audit.

Series	Dose	OR	95% interval	P(OR>1)	Boundary-indeterminate
Primary implementation	10 mg	2.13	0.98 to 4.31	0.973	Yes
Implementation-independent replication	10 mg	2.14	1.01 to 4.30	0.977	Yes
Primary implementation	20 mg	2.29	1.08 to 4.62	0.982	No
Implementation-independent replication	20 mg	2.30	1.10 to 4.64	0.984	No

Under the wider half-normal(0, 1.0) heterogeneity prior, the rupatadine 20-mg posterior median remained similar (OR 2.26) but the 95% CrI included 1 (0.99-4.70). The direct 20-mg versus 10-mg randomized comparison was unadjusted OR 1.08 (two-sided 95% conditional exact CI 0.67-1.74) and did not establish a dose-related difference.

S21. RoB 2, CINeMA, and ROB-MEN Assessments

Table S21. Summary of RoB 2, CINeMA, and ROB-MEN assessments.

Framework	Unit/count	Final distribution	Status
RoB 2	73 result-specific trial-result assessments	49 some concerns; 24 high risk; 0 low risk	Finalized
CINeMA	91 comparison-level assessments	89 very low; 0 low; 2 not evaluable	Finalized
ROB-MEN	91 network-estimate judgments	85 some concerns; 4 low risk; 2 not rateable	Finalized
ROB-MEN pairwise evidence universe	253 pairwise comparisons	Retained in machine-readable workbook	Finalized
ROB-MEN contribution-indicator sensitivity	364 rows: 91 estimates x 4 indicators	Unchanged at 10%, 15%, 20%, 25%	Finalized
ROB-MEN conservative small-study-effect policy	91 rows	89 some concerns; 0 low risk; 2 not rateable	Finalized

Note: Across the 91 current CINeMA assessments, the incoherence domain comprised 25 No concerns, 28 Some concerns, 36 Major concerns, and 2 Not evaluable judgments; the latter corresponded to the two non-estimable WDAE-SAR effects. Overall confidence comprised 89 Very low and 2 Not evaluable assessments. The 147 secondary binary active-active contrasts were not formally appraised with CINeMA.

Table S21 (continued). Final CINeMA domain distributions across 91 assessments.

CINeMA domain	Distribution
Within-study bias	No concerns 0; Some concerns 75; Major concerns 14; Not evaluable 2
Reporting/across-studies bias	No concerns 4; Some concerns 85; Major concerns 0; Not evaluable 2
Indirectness	No concerns 0; Some concerns 81; Major concerns 8; Not evaluable 2
Imprecision	No concerns 38; Some concerns 17; Major concerns 34; Not evaluable 2
Heterogeneity	No concerns 68; Some concerns 17; Major concerns 4; Not evaluable 2
Incoherence	No concerns 25; Some concerns 28; Major concerns 36; Not evaluable 2

Table S21A. Result-specific RoB 2 distribution by principal and exploratory evidence structure

Evidence structure	Assessments	Some concerns	High risk	Low risk
Any AE-SAR	9	7	2	0
Any AE-PAR	8	8	0	0
WDAE-SAR	17	9	8	0
WDAE-PAR	8	8	0	0
TNSS-SAR	13	4	9	0
TNSS-PAR	6	5	1	0
TOSS-SAR	3	1	2	0
RQLQ-SAR	6	4	2	0
RQLQ-PER	2	2	0	0
TOSS-PAR (appraisal-only exploratory)	1	1	0	0
Total	73	49	24	0

Counts are result-specific RoB 2 assessments, not unique primary-model randomizations. TNSS-SAR includes one additional mixed-age exploratory result; TNSS-PAR includes four additional exploratory/sensitivity records from E15, E17, E20, and E22; the E15 TOSS-PAR record was appraisal-only. Full records are in Table S30 and RoB2_Result_Level_Assessments_v1.1.csv.

S22. Contribution-matrix arithmetic and graphical quality assurance

Table S22A. Binary contribution-matrix reconstruction and quality-assurance results.

QA item	Result / interpretation
Release	v56.0; final frozen binary contribution-matrix release
Scope	Any AE–SAR, Any AE–PAR, WDAE–SAR, and WDAE–PAR
Overall status	PASS
Placebo-referenced target estimates	38
Estimable target estimates	36
Non-estimable target estimates	2: rupatadine 10 mg versus placebo and rupatadine 20 mg versus placebo in WDAE–SAR
Likelihood-informative evidence	Any AE–SAR: 9 independent randomizations/23 arms; Any AE–PAR: 8/20; WDAE–SAR: 16/35 after exclusion of the all-zero TF_E51 design from the likelihood; WDAE–PAR: 8/22
Independent-randomization matrix rows	1,390 (legacy QA field: study_matrix_rows)
Direct-evidence matrix rows	1,012 (legacy QA field: direct_matrix_rows)
Maximum absolute contribution-sum error	$2.220446049250313 \times 10^{-16}$
Model-linked validation	PASS for all four binary evidence structures
Controlling QA files	results/contribution_matrix_qa.csv; results/contribution_matrix_qa.json
Qualification	Same-team deterministic reconstruction from frozen source-primary arm data, posterior medians, and τ medians. Implementation-independent model replication remained a separate procedure.

Table S22B. All-structure contribution-matrix arithmetic, provenance, and quality-assurance results.

QA item	Result / interpretation
Release	v56.0; final frozen all-structure contribution-matrix release
Overall status	PASS
Evidence structures	9
Comparison-level appraisal universe	91 targets: 53 pairwise comparisons in the five continuous structures and 38 placebo-referenced targets in the four binary structures
Estimable target columns	89
Non-estimable targets	2: rupatadine 10 mg versus placebo and rupatadine 20 mg versus placebo in WDAE–SAR
Estimable targets by structure	TNSS–SAR: 28; TNSS–PAR: 6; TOSS–SAR: 6; RQLQ–SAR: 10; RQLQ–PER: 3; Any AE–SAR: 9; Any AE–PAR: 10; WDAE–SAR: 7; WDAE–PAR: 10
Long-format decomposition rows	963
Unique target–independent-randomization cells after aggregation	517
Row-level QA	963/963 PASS
Target-column contribution sums	All 89 estimable target columns summed to 100% within numerical tolerance
Maximum absolute target-column sum error	5.68×10^{-14} percentage points
Projection check	PASS

QA item	Result / interpretation
Conservation check	PASS
Aggregation check	PASS
Column-sum check	PASS
Controlling long-format file	data/contribution_matrix_long.csv
Controlling graphical matrix file	code/figures/data/Study_Contribution_Matrix_All_Networks_v56_0.csv (legacy filename; rows represent independent randomizations)
Controlling QA files	results/contribution_matrix_qa.csv; results/contribution_matrix_qa.json
Graphical outputs	Figures S20–S28
Qualification	Arithmetic closure verifies evidence-flow consistency only. It does not alter effect estimates, resolve limitations of the evidence base, or increase comparison-specific confidence.

Note: Contribution matrices were reconstructed at the independent-randomization level by random-walk evidence-flow decomposition and aggregated by target comparison. Shortest-path reconstruction was used solely as an independent arithmetic check. The two non-estimable WDAE–SAR rupatadine effects received no contribution column and were excluded from matrix arithmetic rather than assigned zero contributions. The binary-specific reconstruction underwent additional model-linked checks; the complete matrix underwent projection, conservation, aggregation, row-level, and column-sum checks. Terminology in the table and figure captions should therefore use “independent-randomization-level” and “target–independent-randomization cells,” not “study-level” or “target–study cells.”

S23. Implementation-independent computational replication

Table S23. Implementation-independent R/CmdStanR validation results.

Replication component	Result
Terminal status	Complete with one explicit boundary qualification
Real-data HMC gates	12/12 PASS
Tau comparisons	12/12 PASS
Source-primary magnitude tolerances	4/4 PASS
Exact null-label agreement	3/4
Boundary-indeterminate item	Any AE-SAR, rupatadine 10 mg
Continuous cross-check	PASS: ten GLS/REML scenarios and three direct components
Strict SBC	30/30 PASS
Frozen inputs preserved	Yes
Refit for boundary adjudication	No

The replicator had developed neither source implementation and recoded the models from frozen specifications. Because the replicator had participated in quantitative extraction and evidence appraisal, the exercise was implementation-independent but not fully outcome-blinded; reference outputs remained inaccessible until independent fits and continuous reconstructions had been archived.

S24. Supplementary Figure Inventory and Generation Provenance

Table S24. Supplementary figure inventory and generation provenance.

Figure	Display/scientific purpose	Principal interpretation or qualification	Generator	Controlling scientific input(s)
Figure S1	PRISMA 2020 flow diagram	Study-selection and analytical-disposition flow; reproduces Figure 1 of the main manuscript	–	Main-manuscript Figure 1
Figure S2	TNSS-PAR placebo-referenced forest plot	MDs versus placebo; 95% CIs and plug-in 95% PrIs.	F	Continuous_Effect_Summary_Primary_v56_0.csv
Figure S3	TOSS-SAR placebo-referenced forest plot	MDs versus placebo; 95% CIs and plug-in 95% PrIs.	F	Continuous_Effect_Summary_Primary_v56_0.csv
Figure S4	RQLQ-SAR placebo-referenced forest plot	tau at REML boundary; no plug-in PrI.	F	Continuous_Effect_Summary_Primary_v56_0.csv
Figure S5	RQLQ-PER placebo-referenced forest plot	Saturated; heterogeneity/PrI/incoherence non-estimable.	F	Continuous_Effect_Summary_Primary_v56_0.csv
Figure S6	Any AE-PAR placebo-referenced forest plot	Posterior ORs and 95% CrIs/PrIs.	F	Binary_Effect_Summary_Source_Primary_v56_0.csv; Binary_Prediction_Summary_Source_Primary_v56_0.csv
Figure S7	WDAE-PAR placebo-referenced forest plot	Posterior ORs and 95% CrIs/PrIs.	F	Binary_Effect_Summary_Source_Primary_v56_0.csv; Binary_Prediction_Summary_Source_Primary_v56_0.csv
Figure S8	TNSS-SAR direct-comparison geometry	Node area = accumulated arm N; edges = direct randomized comparisons.	N	Continuous_Primary_Contrast_Data_v56_0.csv
Figure S9	TNSS-PAR direct-comparison geometry	Geometry only.	N	Continuous_Primary_Contrast_Data_v56_0.csv
Figure S10	TOSS-SAR direct-comparison geometry	Geometry only.	N	Continuous_Primary_Contrast_Data_v56_0.csv
Figure S11	RQLQ-SAR direct-comparison geometry	Geometry only.	N	Continuous_Primary_Contrast_Data_v56_0.csv
Figure S12	RQLQ-PER direct-comparison geometry	Geometry only.	N	Continuous_Primary_Contrast_Data_v56_0.csv
Figure S13	Any AE-SAR direct-comparison geometry	Geometry only.	N	Binary_Arm_Data_v1.1.csv
Figure S14	Any AE-PAR direct-comparison geometry	Geometry only.	N	Binary_Arm_Data_v1.1.csv
Figure S15	WDAE-SAR direct-comparison geometry	Includes all-zero three-arm randomization; rupatadine nodes non-estimable.	N	Binary_Arm_Data_v1.1.csv
Figure S16	WDAE-PAR direct-comparison geometry	Geometry only.	N	Binary_Arm_Data_v1.1.csv
Figure S17	Final RoB 2 overall-judgment distribution	73 result-specific assessments: 49 some concerns, 24 high, 0 low.	A	RoB2_Final_Panel_v56_0.csv
Figure S18	Final CINeMA confidence distribution	91 estimates: 89 very low; 2 not evaluable.	A	CINeMA_Comparison_Appraisals_v1.1.csv
Figure S19	Final ROB-MEN distribution	91 estimates: 85 some concerns; 4 low risk; 2 not rateable.	A	ROB_MEN_Comparison_Appraisals_v1.1.csv
Figure S20	TNSS-SAR contribution matrix	Independent-randomization rows; columns sum to 100%.	C	Study_Contribution_Matrix_All_Networks_v56_0.csv
Figure S21	TNSS-PAR contribution matrix	Independent-randomization rows; columns sum to 100%.	C	Study_Contribution_Matrix_All_Networks_v56_0.csv
Figure S22	TOSS-SAR contribution matrix	Independent-randomization rows; columns sum to 100%.	C	Study_Contribution_Matrix_All_Networks_v56_0.csv
Figure S23	RQLQ-SAR contribution matrix	Independent-randomization rows; columns sum to 100%.	C	Study_Contribution_Matrix_All_Networks_v56_0.csv
Figure S24	RQLQ-PER contribution matrix	Independent-randomization rows; columns sum to 100%.	C	Study_Contribution_Matrix_All_Networks_v56_0.csv
Figure S25	Any AE-SAR contribution matrix	Independent-randomization rows; columns sum to 100%.	C	Study_Contribution_Matrix_All_Networks_v56_0.csv
Figure S26	Any AE-PAR contribution matrix	Independent-randomization rows; columns sum to 100%.	C	Study_Contribution_Matrix_All_Networks_v56_0.csv
Figure S27	WDAE-SAR contribution matrix	Seven estimable columns; non-estimable rupatadine effects have no column.	C	Study_Contribution_Matrix_All_Networks_v56_0.csv
Figure S28	WDAE-PAR contribution matrix	Independent-randomization rows; columns sum to 100%.	C	Study_Contribution_Matrix_All_Networks_v56_0.csv
Figure S29	CINeMA domain-judgment distribution	Comparison-specific and estimability-dependent.	A	CINeMA_Comparison_Appraisals_v1.1.csv
Figure S30	ROB-MEN pairwise evidence-availability groups	Evidence availability; not a treatment ranking.	A	ROB_MEN_Pairwise_Comparisons_Table_v56_0.csv
Figure S31	ROB-MEN policy-sensitivity display	Project-specific indicator sensitivities; not official thresholds.	A	ROB_MEN_Threshold_Sensitivity_v56_0.csv
Figure S32	Rupatadine Any AE-SAR null-boundary audit	Primary, replication, direct dose contrast and prior sensitivity.	B	Rupatadine_Boundary_Audit_v56_0.csv; Rupatadine_Direct_Dose_Contrast_v56_0.csv

Generator codes: F = forest figures; N = network figures; A = evidence-appraisal figures; C = contribution figures; B = boundary figure.

S25. Reproducibility Package, Availability, and Interpretive Scope

Material	File/location	Status/qualification
Protocol and audit trail	OSF doi:10.17605/OSF.IO/DPT5N	Public retrospective registration
Previous public repository release	Zenodo v1.0.0; doi:10.5281/zenodo.21780525	Corresponds to submission version 1.0 and analytical release v56.0
Current public repository release	Zenodo v1.1; doi:10.5281/zenodo.22086455	Current reproducibility package supporting submission version 1.1
Current submission	Version 1.1	Complete version 1.1 editorial file set; replaces submitted version 1.0
Eligibility decisions	Screening ledger	179 report-level decisions
Corpus and denominators	Whole-corpus denominator file	61 reports / 66 entries / 64 cohorts; frozen arm-level N=17,887
Study characteristics and RoB 2	Study-characteristics and RoB 2 files	35 study rows; 73 result-specific RoB 2 records
Current appraisals	CINeMA_Comparison_Appraisals_v1.1.csv; ROB_MEN_Comparison_Appraisals_v1.1.csv	91 current comparison-level records
Binary active-active results	Supplementary_Data_S1_Binary_Active_Active_Effects_v1.1.csv	147 secondary exact within-draw contrasts; no refit or new MCMC
Placebo concordance	Binary_Placebo_Referenced_Concordance_Check_v1.1.csv	36/36 effects within prespecified numerical tolerances
Binary arm data	Binary_Arm_Data_v1.1.csv	103 arms across four binary structures
Derivation code	derive_binary_active_active_from_retained_draws_v1.1.py	Reads native chains and treatment maps; aborts unless 36/45/21/45=147
Human validation	validate_binary_active_active_LRR_v1.1.R	Executed by L.R.R.; 147/147 PASS; max difference $5.0000049 \times 10^{-10}$; completed record included.
Retained draw subset	Binary_Active_Active_Source_Draws_v1.1.zip	Stan model, four inputs/maps/metadata sets, and 16 native CmdStan chain CSVs
Source manifest	SOURCE_FREEZE_SHA256_v1.1.csv	SHA-256 for source inputs and retained chains
Version crosswalk	Submission_Version_Crosswalk_v1.1.csv	Explicit relationship between version 1.0/v56.0 and current version 1.1
Reporting checklists	PRISMA 2020; PRISMA Abstracts; PRISMA-NMA; PRISMA-S	Completed reporting checklists included in the Supplementary Materials
Repository package	Zenodo version 1.1; doi:10.5281/zenodo.22086455	Publicly deposited reproducibility package corresponding to the current submission

Material	Availability statement	Interpretation
Original database exports and platform timestamps	Not retained; exact strategies, dates, and record counts reported in S2	Limitation reported transparently.
Duplicate-cluster log	Not retained; operational rules and final duplicate classifications reported	Selection arithmetic and decisions remain auditable.
Reviewer-specific screening timestamps	Not retained; independent dual review and adjudication reported	No effect on the final eligibility ledger.
Joint posterior draws	Not retained by the source-primary implementation; retained by the implementation-independent CmdStanR replication	Used only for secondary exact within-draw active-active contrasts.
CINeMA for the 147 secondary contrasts	Not formally assessed	No rating was inferred; contrasts are non-confirmatory.
Multiplicity	No family-wise adjustment for the 147 secondary contrasts	Intervals excluding 1 do not establish confirmatory superiority.

Public repository: Zenodo v1.1 (doi:10.5281/zenodo.22086455) is the current public reproducibility release; Zenodo v1.0.0 (doi:10.5281/zenodo.21780525) remains the archived release corresponding to submission version 1.0.

S26. Complete Active-Active Comparative Effects

Table S26. Complete network estimates for the 34 continuous active-active comparisons. No comparison had independently estimable direct and indirect components; unavailable components are reported explicitly rather than imputed.

Structure	Active regimen comparison	NMA estimate (95% CI)	Evidence route	Independent direct component	Independent indirect component	CINeMA	ROB-MEN
RQLQ-PER	Levocetirizine 5 mg OD vs Desloratadine 5 mg OD	-0.18 (-0.45 to 0.09)	Indirect-only through placebo	No direct evidence	Not independently estimable	Very low	Some concerns
RQLQ-SAR	Desloratadine 5 mg OD vs Bilastine 20 mg OD	-0.04 (-0.24 to 0.17)	Directly informed	Not independently estimable	Not independently estimable	Very low	Some concerns
RQLQ-SAR	Levocetirizine 5 mg OD vs Bilastine 20 mg OD	0.17 (-0.08 to 0.43)	Indirect-only through placebo	No direct evidence	Not independently estimable	Very low	Some concerns
RQLQ-SAR	Levocetirizine 5 mg OD vs Desloratadine 5 mg OD	0.21 (-0.01 to 0.43)	Indirect-only through placebo	No direct evidence	Not independently estimable	Very low	Some concerns
RQLQ-SAR	Loratadine 10 mg OD vs Bilastine 20 mg OD	0.04 (-0.21 to 0.28)	Indirect-only through placebo	No direct evidence	Not independently estimable	Very low	Some concerns
RQLQ-SAR	Loratadine 10 mg OD vs Desloratadine 5 mg OD	0.07 (-0.13 to 0.28)	Indirect-only through placebo	No direct evidence	Not independently estimable	Very low	Some concerns
RQLQ-SAR	Loratadine 10 mg OD vs Levocetirizine 5 mg OD	-0.14 (-0.33 to 0.05)	Indirect-only through placebo	No direct evidence	Not independently estimable	Very low	Some concerns
TNSS-PAR	Rupatadine 10 mg OD vs Cetirizine 10 mg OD	-0.31 (-1.05 to 0.44)	Directly informed	Not independently estimable	Not independently estimable	Very low	Some concerns
TNSS-PAR	Rupatadine 20 mg OD vs Cetirizine 10 mg OD	-0.57 (-1.34 to 0.21)	Directly informed	Not independently estimable	Not independently estimable	Very low	Some concerns
TNSS-PAR	Rupatadine 20 mg OD vs Rupatadine 10 mg OD	-0.26 (-1.12 to 0.60)	Directly informed	Not independently estimable	Not independently estimable	Very low	Some concerns
TNSS-SAR	Cetirizine 10 mg OD vs Bilastine 20 mg OD	-0.24 (-0.74 to 0.25)	Directly informed	Not independently estimable	Not independently estimable	Very low	Some concerns
TNSS-SAR	Desloratadine 5 mg OD vs Bilastine 20 mg OD	0.17 (-0.43 to 0.78)	Indirect-only through placebo	No direct evidence	Not independently estimable	Very low	Some concerns
TNSS-SAR	Desloratadine 5 mg OD vs Cetirizine 10 mg OD	0.41 (-0.07 to 0.89)	Indirect-only through placebo	No direct evidence	Not independently estimable	Very low	Some concerns
TNSS-SAR	Fexofenadine 180 mg OD vs Bilastine 20 mg OD	0.86 (0.24 to 1.47)	Indirect-only through placebo	No direct evidence	Not independently estimable	Very low	Some concerns
TNSS-SAR	Fexofenadine 180 mg OD vs Cetirizine 10 mg OD	1.10 (0.60 to 1.59)	Indirect-only through placebo	No direct evidence	Not independently estimable	Very low	Some concerns
TNSS-SAR	Fexofenadine 180 mg OD vs Desloratadine 5 mg OD	0.68 (0.18 to 1.19)	Indirect-only through placebo	No direct evidence	Not independently estimable	Very low	Some concerns
TNSS-SAR	Loratadine 10 mg OD vs Bilastine 20 mg OD	0.02 (-0.53 to 0.57)	Indirect-only through placebo	No direct evidence	Not independently estimable	Very low	Some concerns
TNSS-SAR	Loratadine 10 mg OD vs Cetirizine 10 mg OD	0.26 (-0.15 to 0.67)	Indirect-only through placebo	No direct evidence	Not independently estimable	Very low	Some concerns
TNSS-SAR	Loratadine 10 mg OD vs Desloratadine 5 mg OD	-0.15 (-0.57 to 0.27)	Indirect-only through placebo	No direct evidence	Not independently estimable	Very low	Some concerns
TNSS-SAR	Loratadine 10 mg OD vs Fexofenadine 180 mg OD	-0.84 (-1.27 to -0.40)	Indirect-only through placebo	No direct evidence	Not independently estimable	Very low	Some concerns
TNSS-SAR	Rupatadine 10 mg OD vs Bilastine 20 mg OD	0.12 (-0.55 to 0.78)	Indirect-only through placebo	No direct evidence	Not independently estimable	Very low	Some concerns
TNSS-SAR	Rupatadine 10 mg OD vs Cetirizine 10 mg OD	0.36 (-0.20 to 0.91)	Indirect-only through placebo	No direct evidence	Not independently estimable	Very low	Some concerns
TNSS-SAR	Rupatadine 10 mg OD vs Desloratadine 5 mg OD	-0.06 (-0.62 to 0.51)	Indirect-only through placebo	No direct evidence	Not independently estimable	Very low	Some concerns
TNSS-SAR	Rupatadine 10 mg OD vs Fexofenadine 180 mg OD	-0.74 (-1.31 to -0.17)	Indirect-only through placebo	No direct evidence	Not independently estimable	Very low	Some concerns
TNSS-SAR	Rupatadine 10 mg OD vs Loratadine 10 mg OD	0.10 (-0.41 to 0.60)	Indirect-only through placebo	No direct evidence	Not independently estimable	Very low	Some concerns
TNSS-SAR	Rupatadine 20 mg OD vs Bilastine 20 mg OD	-0.20 (-0.87 to 0.47)	Indirect-only through placebo	No direct evidence	Not independently estimable	Very low	Some concerns
TNSS-SAR	Rupatadine 20 mg OD vs Cetirizine 10 mg OD	0.04 (-0.52 to 0.60)	Indirect-only through placebo	No direct evidence	Not independently estimable	Very low	Some concerns
TNSS-SAR	Rupatadine 20 mg OD vs Desloratadine 5 mg OD	-0.37 (-0.94 to 0.19)	Indirect-only through placebo	No direct evidence	Not independently estimable	Very low	Some concerns
TNSS-SAR	Rupatadine 20 mg OD vs Fexofenadine 180 mg OD	-1.06 (-1.64 to -0.48)	Indirect-only through placebo	No direct evidence	Not independently estimable	Very low	Some concerns
TNSS-SAR	Rupatadine 20 mg OD vs Loratadine 10 mg OD	-0.22 (-0.73 to 0.28)	Indirect-only through placebo	No direct evidence	Not independently estimable	Very low	Some concerns
TNSS-SAR	Rupatadine 20 mg OD vs Rupatadine 10 mg OD	-0.32 (-0.77 to 0.14)	Directly informed	Not independently estimable	Not independently estimable	Very low	Some concerns

Structure	Active regimen comparison	NMA estimate (95% CI)	Evidence route	Independent direct component	Independent indirect component	CINeMA	ROB-MEN
TOSS-SAR	Cetirizine 10 mg OD vs Bilastine 20 mg OD	-0.11 (-0.55 to 0.33)	Directly informed	Not independently estimable	Not independently estimable	Very low	Some concerns
TOSS-SAR	Fexofenadine 180 mg OD vs Bilastine 20 mg OD	0.67 (0.15 to 1.20)	Indirect-only through placebo	No direct evidence	Not independently estimable	Very low	Some concerns
TOSS-SAR	Fexofenadine 180 mg OD vs Cetirizine 10 mg OD	0.78 (0.25 to 1.32)	Indirect-only through placebo	No direct evidence	Not independently estimable	Very low	Some concerns

S27. Comparison-Level CINeMA and ROB-MEN Appraisals

Table S27. Final matrix for all 91 target estimates. Binary rows use source-primary OR medians and 95% CrIs; continuous rows use MDs and 95% CIs.

Structure	Comparison	Effect (95% CI/CrI)	Route	WSB	ASB	Ind	Imp	Het	Inc	CINeMA	ROB-MEN
TNSS-SAR	Bilastine 20 mg OD vs Placebo	MD -0.93 (-1.43 to -0.43)	Direct; independent indirect not estimable	Some concerns	Some concerns	Some concerns	No concerns	No concerns	Some concerns	Very low	Some concerns
TNSS-SAR	Cetirizine 10 mg OD vs Bilastine 20 mg OD	MD -0.24 (-0.74 to 0.25)	Direct; independent indirect not estimable	Some concerns	Some concerns	Some concerns	Some concerns	No concerns	Some concerns	Very low	Some concerns
TNSS-SAR	Cetirizine 10 mg OD vs Placebo	MD -1.17 (-1.50 to -0.83)	Direct; independent indirect not estimable	Major concerns	Some concerns	Some concerns	No concerns	No concerns	Some concerns	Very low	Some concerns
TNSS-SAR	Desloratadine 5 mg OD vs Bilastine 20 mg OD	MD 0.17 (-0.43 to 0.78)	Indirect-only through placebo	Some concerns	Some concerns	Some concerns	Some concerns	No concerns	Some concerns	Very low	Some concerns
TNSS-SAR	Desloratadine 5 mg OD vs Cetirizine 10 mg OD	MD 0.41 (-0.07 to 0.89)	Indirect-only through placebo	Some concerns	Some concerns	Some concerns	Some concerns	No concerns	Some concerns	Very low	Some concerns
TNSS-SAR	Desloratadine 5 mg OD vs Placebo	MD -0.76 (-1.10 to -0.41)	Direct; independent indirect not estimable	Some concerns	Some concerns	Major concerns	No concerns	No concerns	Some concerns	Very low	Some concerns
TNSS-SAR	Fexofenadine 180 mg OD vs Bilastine 20 mg OD	MD 0.86 (0.24 to 1.47)	Indirect-only through placebo	Major concerns	Some concerns	Some concerns	No concerns	No concerns	Some concerns	Very low	Some concerns
TNSS-SAR	Fexofenadine 180 mg OD vs Cetirizine 10 mg OD	MD 1.10 (0.60 to 1.59)	Indirect-only through placebo	Major concerns	Some concerns	Some concerns	No concerns	No concerns	Some concerns	Very low	Some concerns
TNSS-SAR	Fexofenadine 180 mg OD vs Desloratadine 5 mg OD	MD 0.68 (0.18 to 1.19)	Indirect-only through placebo	Some concerns	Some concerns	Some concerns	No concerns	No concerns	Some concerns	Very low	Some concerns
TNSS-SAR	Fexofenadine 180 mg OD vs Placebo	MD -0.07 (-0.43 to 0.29)	Direct; independent indirect not estimable	Major concerns	Some concerns	Some concerns	No concerns	Some concerns	Some concerns	Very low	Some concerns
TNSS-SAR	Loratadine 10 mg OD vs Bilastine 20 mg OD	MD 0.02 (-0.53 to 0.57)	Indirect-only through placebo	Major concerns	Some concerns	Some concerns	Some concerns	Major concerns	Some concerns	Very low	Some concerns
TNSS-SAR	Loratadine 10 mg OD vs Cetirizine 10 mg OD	MD 0.26 (-0.15 to 0.67)	Indirect-only through placebo	Major concerns	Some concerns	Some concerns	Some concerns	No concerns	Some concerns	Very low	Some concerns
TNSS-SAR	Loratadine 10 mg OD vs Desloratadine 5 mg OD	MD -0.15 (-0.57 to 0.27)	Indirect-only through placebo	Some concerns	Some concerns	Major concerns	Some concerns	No concerns	Some concerns	Very low	Some concerns
TNSS-SAR	Loratadine 10 mg OD vs Fexofenadine 180 mg OD	MD -0.84 (-1.27 to -0.40)	Indirect-only through placebo	Major concerns	Some concerns	Some concerns	No concerns	No concerns	Some concerns	Very low	Some concerns
TNSS-SAR	Loratadine 10 mg OD vs Placebo	MD -0.91 (-1.15 to -0.67)	Direct; independent indirect not estimable	Major concerns	Some concerns	Some concerns	No concerns	No concerns	Some concerns	Very low	Some concerns

Structure	Comparison	Effect (95% CI/CrI)	Route	WSB	ASB	Ind	Imp	Het	Inc	CINeMA	ROB-MEN
TNSS-SAR	Rupatadine 10 mg OD vs Bilastine 20 mg OD	MD 0.12 (-0.55 to 0.78)	Indirect-only through placebo	Some concerns	Some concerns	Some concerns	Some concerns	Major concerns	Some concerns	Very low	Some concerns
TNSS-SAR	Rupatadine 10 mg OD vs Cetirizine 10 mg OD	MD 0.36 (-0.20 to 0.91)	Indirect-only through placebo	Some concerns	Some concerns	Some concerns	Some concerns	No concerns	Some concerns	Very low	Some concerns
TNSS-SAR	Rupatadine 10 mg OD vs Desloratadine 5 mg OD	MD -0.06 (-0.62 to 0.51)	Indirect-only through placebo	Some concerns	Some concerns	Some concerns	Some concerns	Major concerns	Some concerns	Very low	Some concerns
TNSS-SAR	Rupatadine 10 mg OD vs Fexofenadine 180 mg OD	MD -0.74 (-1.31 to -0.17)	Indirect-only through placebo	Some concerns	Some concerns	Some concerns	No concerns	No concerns	Some concerns	Very low	Some concerns
TNSS-SAR	Rupatadine 10 mg OD vs Loratadine 10 mg OD	MD 0.10 (-0.41 to 0.60)	Indirect-only through placebo	Some concerns	Some concerns	Some concerns	Some concerns	No concerns	Some concerns	Very low	Some concerns
TNSS-SAR	Rupatadine 10 mg OD vs Placebo	MD -0.81 (-1.25 to -0.37)	Direct; independent indirect not estimable	Some concerns	Some concerns	Some concerns	No concerns	No concerns	Some concerns	Very low	Some concerns
TNSS-SAR	Rupatadine 20 mg OD vs Bilastine 20 mg OD	MD -0.20 (-0.87 to 0.47)	Indirect-only through placebo	Some concerns	Some concerns	Some concerns	Some concerns	No concerns	Some concerns	Very low	Some concerns
TNSS-SAR	Rupatadine 20 mg OD vs Cetirizine 10 mg OD	MD 0.04 (-0.52 to 0.60)	Indirect-only through placebo	Some concerns	Some concerns	Some concerns	Some concerns	Major concerns	Some concerns	Very low	Some concerns
TNSS-SAR	Rupatadine 20 mg OD vs Desloratadine 5 mg OD	MD -0.37 (-0.94 to 0.19)	Indirect-only through placebo	Some concerns	Some concerns	Some concerns	Some concerns	No concerns	Some concerns	Very low	Some concerns
TNSS-SAR	Rupatadine 20 mg OD vs Fexofenadine 180 mg OD	MD -1.06 (-1.64 to -0.48)	Indirect-only through placebo	Some concerns	Some concerns	Some concerns	No concerns	No concerns	Some concerns	Very low	Some concerns
TNSS-SAR	Rupatadine 20 mg OD vs Loratadine 10 mg OD	MD -0.22 (-0.73 to 0.28)	Indirect-only through placebo	Some concerns	Some concerns	Some concerns	Some concerns	No concerns	Some concerns	Very low	Some concerns
TNSS-SAR	Rupatadine 20 mg OD vs Placebo	MD -1.13 (-1.58 to -0.68)	Direct; independent indirect not estimable	Some concerns	Some concerns	Some concerns	No concerns	No concerns	Some concerns	Very low	Some concerns
TNSS-SAR	Rupatadine 20 mg OD vs Rupatadine 10 mg OD	MD -0.32 (-0.77 to 0.14)	Direct; independent indirect not estimable	Some concerns	Some concerns	Some concerns	Some concerns	No concerns	Some concerns	Very low	Some concerns
TNSS-PAR	Cetirizine 10 mg OD vs Placebo	MD -0.50 (-0.87 to -0.12)	Direct; independent indirect not estimable	Major concerns	Some concerns	Some concerns	No concerns	No concerns	No concerns	Very low	Some concerns
TNSS-PAR	Rupatadine 10 mg OD vs Cetirizine 10 mg OD	MD -0.31 (-1.05 to 0.44)	Direct; independent indirect not estimable	Some concerns	Some concerns	Major concerns	Some concerns	No concerns	No concerns	Very low	Some concerns
TNSS-PAR	Rupatadine 10 mg OD vs Placebo	MD -0.80 (-1.53 to -0.07)	Direct; independent indirect not estimable	Some concerns	Some concerns	Major concerns	No concerns	No concerns	No concerns	Very low	Some concerns

Structure	Comparison	Effect (95% CI/CrI)	Route	WSB	ASB	Ind	Imp	Het	Inc	CINeMA	ROB-MEN
TNSS-PAR	Rupatadine 20 mg OD vs Cetirizine 10 mg OD	MD -0.57 (-1.34 to 0.21)	Direct; independent indirect not estimable	Some concerns	Some concerns	Major concerns	Some concerns	No concerns	No concerns	Very low	Some concerns
TNSS-PAR	Rupatadine 20 mg OD vs Placebo	MD -1.06 (-1.83 to -0.30)	Direct; independent indirect not estimable	Some concerns	Some concerns	Major concerns	No concerns	No concerns	No concerns	Very low	Some concerns
TNSS-PAR	Rupatadine 20 mg OD vs Rupatadine 10 mg OD	MD -0.26 (-1.12 to 0.60)	Direct; independent indirect not estimable	Some concerns	Some concerns	Major concerns	Major concerns	No concerns	No concerns	Very low	Some concerns
TOSS-SAR	Bilastine 20 mg OD vs Placebo	MD -0.74 (-1.18 to -0.30)	Direct; independent indirect not estimable	Some concerns	Some concerns	Some concerns	No concerns	No concerns	Major concerns	Very low	Some concerns
TOSS-SAR	Cetirizine 10 mg OD vs Bilastine 20 mg OD	MD -0.11 (-0.55 to 0.33)	Direct; independent indirect not estimable	Some concerns	Some concerns	Some concerns	No concerns	No concerns	Major concerns	Very low	Some concerns
TOSS-SAR	Cetirizine 10 mg OD vs Placebo	MD -0.85 (-1.30 to -0.40)	Direct; independent indirect not estimable	Some concerns	Some concerns	Some concerns	No concerns	No concerns	Major concerns	Very low	Some concerns
TOSS-SAR	Fexofenadine 180 mg OD vs Bilastine 20 mg OD	MD 0.67 (0.15 to 1.20)	Indirect-only through placebo	Some concerns	Some concerns	Some concerns	No concerns	No concerns	Major concerns	Very low	Some concerns
TOSS-SAR	Fexofenadine 180 mg OD vs Cetirizine 10 mg OD	MD 0.78 (0.25 to 1.32)	Indirect-only through placebo	Some concerns	Some concerns	Some concerns	No concerns	No concerns	Major concerns	Very low	Some concerns
TOSS-SAR	Fexofenadine 180 mg OD vs Placebo	MD -0.07 (-0.36 to 0.23)	Direct; independent indirect not estimable	Major concerns	Some concerns	Some concerns	No concerns	No concerns	Major concerns	Very low	Some concerns
RQLQ-SAR	Bilastine 20 mg OD vs Placebo	MD -0.34 (-0.55 to -0.14)	Direct; independent indirect not estimable	Some concerns	Some concerns	Some concerns	No concerns	Some concerns	No concerns	Very low	Some concerns
RQLQ-SAR	Desloratadine 5 mg OD vs Bilastine 20 mg OD	MD -0.04 (-0.24 to 0.17)	Direct; independent indirect not estimable	Some concerns	Some concerns	Some concerns	No concerns	Some concerns	No concerns	Very low	Some concerns
RQLQ-SAR	Desloratadine 5 mg OD vs Placebo	MD -0.38 (-0.55 to -0.22)	Direct; independent indirect not estimable	Some concerns	Some concerns	Some concerns	No concerns	Some concerns	No concerns	Very low	Some concerns
RQLQ-SAR	Levocetirizine 5 mg OD vs Bilastine 20 mg OD	MD 0.17 (-0.08 to 0.43)	Indirect-only through placebo	Some concerns	Some concerns	Some concerns	No concerns	Some concerns	No concerns	Very low	Some concerns
RQLQ-SAR	Levocetirizine 5 mg OD vs Desloratadine 5 mg OD	MD 0.21 (-0.01 to 0.43)	Indirect-only through placebo	Some concerns	Some concerns	Some concerns	No concerns	Some concerns	No concerns	Very low	Some concerns

Structure	Comparison	Effect (95% CI/CrI)	Route	WSB	ASB	Ind	Imp	Het	Inc	CINeMA	ROB-MEN
RQLQ-SAR	Levocetirizine 5 mg OD vs Placebo	MD -0.17 (-0.32 to -0.03)	Direct; independent indirect not estimable	Some concerns	No concerns	Some concerns	No concerns	Some concerns	No concerns	Very low	Low risk
RQLQ-SAR	Loratadine 10 mg OD vs Bilastine 20 mg OD	MD 0.04 (-0.21 to 0.28)	Indirect-only through placebo	Some concerns	Some concerns	Some concerns	No concerns	Some concerns	No concerns	Very low	Some concerns
RQLQ-SAR	Loratadine 10 mg OD vs Desloratadine 5 mg OD	MD 0.07 (-0.13 to 0.28)	Indirect-only through placebo	Some concerns	Some concerns	Some concerns	No concerns	Some concerns	No concerns	Very low	Some concerns
RQLQ-SAR	Loratadine 10 mg OD vs Levocetirizine 5 mg OD	MD -0.14 (-0.33 to 0.05)	Indirect-only through placebo	Some concerns	Some concerns	Some concerns	No concerns	Some concerns	No concerns	Very low	Some concerns
RQLQ-SAR	Loratadine 10 mg OD vs Placebo	MD -0.31 (-0.44 to -0.18)	Direct; independent indirect not estimable	Major concerns	Some concerns	Some concerns	No concerns	Some concerns	No concerns	Very low	Some concerns
RQLQ-PER	Desloratadine 5 mg OD vs Placebo	MD -0.30 (-0.49 to -0.11)	Direct; independent indirect not estimable	Some concerns	Some concerns	Some concerns	No concerns	Some concerns	Major concerns	Very low	Some concerns
RQLQ-PER	Levocetirizine 5 mg OD vs Desloratadine 5 mg OD	MD -0.18 (-0.45 to 0.09)	Indirect-only through placebo	Some concerns	Some concerns	Some concerns	No concerns	Some concerns	Major concerns	Very low	Some concerns
RQLQ-PER	Levocetirizine 5 mg OD vs Placebo	MD -0.48 (-0.67 to -0.29)	Direct; independent indirect not estimable	Some concerns	Some concerns	Some concerns	No concerns	Some concerns	Major concerns	Very low	Some concerns
Any AE-SAR	Bilastine 20 mg OD vs Placebo	OR 0.89 (0.54 to 1.36)	Direct; independent indirect not estimable	Some concerns	Some concerns	Some concerns	Major concerns	No concerns	No concerns	Very low	Some concerns
Any AE-SAR	Cetirizine 10 mg OD vs Placebo	OR 1.15 (0.60 to 1.82)	Direct; independent indirect not estimable	Some concerns	Some concerns	Some concerns	Major concerns	No concerns	No concerns	Very low	Some concerns
Any AE-SAR	Desloratadine 5 mg OD vs Placebo	OR 1.24 (0.66 to 2.21)	Direct; independent indirect not estimable	Some concerns	Some concerns	Some concerns	Major concerns	No concerns	No concerns	Very low	Some concerns
Any AE-SAR	Fexofenadine 120 mg OD vs Placebo	OR 1.04 (0.66 to 1.66)	Direct; independent indirect not estimable	Some concerns	Some concerns	Some concerns	Major concerns	No concerns	No concerns	Very low	Some concerns
Any AE-SAR	Fexofenadine 180 mg OD vs Placebo	OR 1.04 (0.58 to 1.87)	Direct; independent indirect not estimable	Some concerns	Some concerns	Some concerns	Major concerns	No concerns	No concerns	Very low	Some concerns
Any AE-SAR	Levocetirizine 5 mg OD vs Placebo	OR 0.86 (0.53 to 1.38)	Direct; independent indirect not estimable	Some concerns	No concerns	Some concerns	Major concerns	No concerns	No concerns	Very low	Low risk

Structure	Comparison	Effect (95% CI/CrI)	Route	WSB	ASB	Ind	Imp	Het	Inc	CINeMA	ROB-MEN
Any AE-SAR	Loratadine 10 mg OD vs Placebo	OR 1.09 (0.68 to 1.73)	Direct; independent indirect not estimable	Major concerns	Some concerns	Major concerns	Major concerns	No concerns	No concerns	Very low	Some concerns
Any AE-SAR	Rupatadine 10 mg OD vs Placebo	OR 2.13 (0.98 to 4.31)	Direct; independent indirect not estimable	Some concerns	Some concerns	Some concerns	No concerns	Some concerns	No concerns	Very low	Some concerns
Any AE-SAR	Rupatadine 20 mg OD vs Placebo	OR 2.29 (1.08 to 4.62)	Direct; independent indirect not estimable	Some concerns	Some concerns	Some concerns	No concerns	Some concerns	No concerns	Very low	Some concerns
Any AE-PAR	Bepotastine 10 mg BID vs Placebo	OR 1.59 (0.70 to 3.51)	Local direct-indirect separation not robust	Some concerns	Some concerns	Some concerns	Major concerns	No concerns	Major concerns	Very low	Some concerns
Any AE-PAR	Bepotastine CR 20 mg OD vs Placebo	OR 1.65 (0.71 to 3.87)	Local direct-indirect separation not robust	Some concerns	Some concerns	Some concerns	Major concerns	No concerns	Major concerns	Very low	Some concerns
Any AE-PAR	Bilastine 20 mg OD vs Placebo	OR 1.03 (0.55 to 1.96)	Local direct-indirect separation not robust	Some concerns	Some concerns	Some concerns	Major concerns	No concerns	Major concerns	Very low	Some concerns
Any AE-PAR	Cetirizine 10 mg OD vs Placebo	OR 0.80 (0.49 to 1.37)	Local direct-indirect separation not robust	Some concerns	Some concerns	Some concerns	Major concerns	No concerns	Major concerns	Very low	Some concerns
Any AE-PAR	Desloratadine 5 mg OD vs Placebo	OR 0.76 (0.39 to 1.50)	Local direct-indirect separation not robust	Some concerns	Some concerns	Some concerns	Major concerns	No concerns	Major concerns	Very low	Some concerns
Any AE-PAR	Ebastine 10 mg OD vs Placebo	OR 0.68 (0.27 to 1.86)	Local direct-indirect separation not robust	Some concerns	Some concerns	Some concerns	Major concerns	No concerns	Major concerns	Very low	Some concerns
Any AE-PAR	Ebastine 20 mg OD vs Placebo	OR 0.54 (0.22 to 1.50)	Local direct-indirect separation not robust	Some concerns	Some concerns	Some concerns	Major concerns	No concerns	Major concerns	Very low	Some concerns
Any AE-PAR	Loratadine 10 mg OD vs Placebo	OR 0.71 (0.38 to 1.40)	Local direct-indirect separation not robust	Some concerns	Some concerns	Some concerns	Major concerns	No concerns	Major concerns	Very low	Some concerns
Any AE-PAR	Mizolastine 10 mg OD vs Placebo	OR 0.86 (0.51 to 1.47)	Local direct-indirect separation not robust	Some concerns	Some concerns	Some concerns	Major concerns	No concerns	Major concerns	Very low	Some concerns

Structure	Comparison	Effect (95% CI/CrI)	Route	WSB	ASB	Ind	Imp	Het	Inc	CINeMA	ROB-MEN
Any AE-PAR	Rupatadine 10 mg OD vs Placebo	OR 1.40 (0.63 to 3.14)	Local direct-indirect separation not robust	Some concerns	Some concerns	Some concerns	Major concerns	No concerns	Major concerns	Very low	Some concerns
WDAE-SAR	Bilastine 20 mg OD vs Placebo	OR 0.34 (0.08 to 1.27)	Local direct-indirect separation not robust	Some concerns	Some concerns	Some concerns	Major concerns	No concerns	Major concerns	Very low	Some concerns
WDAE-SAR	Cetirizine 10 mg OD vs Placebo	OR 0.25 (0.08 to 0.66)	Local direct-indirect separation not robust	Some concerns	No concerns	Some concerns	No concerns	Some concerns	Major concerns	Very low	Low risk
WDAE-SAR	Desloratadine 5 mg OD vs Placebo	OR 0.74 (0.26 to 2.12)	Local direct-indirect separation not robust	Some concerns	Some concerns	Some concerns	Major concerns	No concerns	Major concerns	Very low	Some concerns
WDAE-SAR	Fexofenadine 120 mg OD vs Placebo	OR 0.52 (0.13 to 1.91)	Local direct-indirect separation not robust	Some concerns	Some concerns	Some concerns	Major concerns	No concerns	Major concerns	Very low	Some concerns
WDAE-SAR	Fexofenadine 180 mg OD vs Placebo	OR 1.69 (0.50 to 6.03)	Local direct-indirect separation not robust	Major concerns	Some concerns	Some concerns	Major concerns	No concerns	Major concerns	Very low	Some concerns
WDAE-SAR	Levocetirizine 5 mg OD vs Placebo	OR 0.46 (0.11 to 1.57)	Local direct-indirect separation not robust	Some concerns	No concerns	Some concerns	Major concerns	No concerns	Major concerns	Very low	Low risk
WDAE-SAR	Loratadine 10 mg OD vs Placebo	OR 1.07 (0.47 to 2.41)	Local direct-indirect separation not robust	Major concerns	Some concerns	Some concerns	Major concerns	No concerns	Major concerns	Very low	Some concerns
WDAE-SAR	Rupatadine 10 mg OD vs Placebo	Not estimable	Non-estimable network effect	Not evaluable	Not evaluable	Not evaluable	Not evaluable	Not evaluable	Not evaluable	Not evaluable	Not rateable
WDAE-SAR	Rupatadine 20 mg OD vs Placebo	Not estimable	Non-estimable network effect	Not evaluable	Not evaluable	Not evaluable	Not evaluable	Not evaluable	Not evaluable	Not evaluable	Not rateable
WDAE-PAR	Bepotastine 10 mg BID vs Placebo	OR 0.66 (0.08 to 4.51)	Local direct-indirect separation not robust	Some concerns	Some concerns	Some concerns	Major concerns	No concerns	Major concerns	Very low	Some concerns
WDAE-PAR	Bilastine 20 mg OD vs Placebo	OR 1.20 (0.21 to 6.14)	Local direct-indirect separation not robust	Some concerns	Some concerns	Some concerns	Major concerns	No concerns	Major concerns	Very low	Some concerns
WDAE-PAR	Cetirizine 10 mg OD vs Placebo	OR 0.77 (0.21 to 2.76)	Local direct-indirect separation not robust	Some concerns	Some concerns	Some concerns	Major concerns	No concerns	Major concerns	Very low	Some concerns

Structure	Comparison	Effect (95% CI/CrI)	Route	WSB	ASB	Ind	Imp	Het	Inc	CINeMA	ROB-MEN
WDAE-PAR	Desloratadine 5 mg OD vs Placebo	OR 1.46 (0.46 to 4.57)	Local direct-indirect separation not robust	Some concerns	Some concerns	Some concerns	Major concerns	No concerns	Major concerns	Very low	Some concerns
WDAE-PAR	Ebastine 10 mg OD vs Placebo	OR 1.51 (0.21 to 10.45)	Local direct-indirect separation not robust	Some concerns	Some concerns	Some concerns	Major concerns	No concerns	Major concerns	Very low	Some concerns
WDAE-PAR	Ebastine 20 mg OD vs Placebo	OR 0.35 (0.03 to 2.99)	Local direct-indirect separation not robust	Some concerns	Some concerns	Some concerns	Major concerns	No concerns	Major concerns	Very low	Some concerns
WDAE-PAR	Loratadine 10 mg OD vs Placebo	OR 1.69 (0.55 to 5.06)	Local direct-indirect separation not robust	Some concerns	Some concerns	Some concerns	Major concerns	No concerns	Major concerns	Very low	Some concerns
WDAE-PAR	Mizolastine 10 mg OD vs Placebo	OR 0.73 (0.23 to 2.22)	Local direct-indirect separation not robust	Some concerns	Some concerns	Some concerns	Major concerns	No concerns	Major concerns	Very low	Some concerns
WDAE-PAR	Rupatadine 10 mg OD vs Placebo	OR 0.43 (0.07 to 2.18)	Local direct-indirect separation not robust	Some concerns	Some concerns	Some concerns	Major concerns	No concerns	Major concerns	Very low	Some concerns
WDAE-PAR	Rupatadine 20 mg OD vs Placebo	OR 0.84 (0.11 to 5.36)	Local direct-indirect separation not robust	Some concerns	Some concerns	Some concerns	Major concerns	No concerns	Major concerns	Very low	Some concerns

S28. Model Inputs and Individual-Study/Arm Reporting

Table S28A. Continuous model-ready contrasts. Okubo source-scale values are shown alongside transformed values.

ID	Structure	Trial unit	Treatment	Control	Source effect	Source SE	Model effect	Model SE	Time	Covariance/source
CONT-P-0001	TNSS_SAR	TF_E42	bilastine_20_od	placebo	Same as model scale	Same as model SE	-1.140	0.225	day 14	E42_TNSS; Table 4, change from baseline in NSS
CONT-P-0002	TNSS_SAR	TF_E42	cetirizine_10_od	placebo	Same as model scale	Same as model SE	-1.590	0.233	day 14	E42_TNSS; Table 4, change from baseline in NSS
CONT-P-0003	TNSS_SAR	TF_E51	rupatadine_10_od	placebo	-1.084 (0-16)	0.208	-0.813	0.156	week 2	E51_TNSS; Table 1: T4NSS 0-16 mean change and SE transformed exactly x0.75 to canonical 0-12 TNSS scale
CONT-P-0004	TNSS_SAR	TF_E51	rupatadine_20_od	placebo	-1.509 (0-16)	0.215	-1.132	0.161	week 2	E51_TNSS; Table 1: T4NSS 0-16 mean change and SE transformed exactly x0.75 to canonical 0-12 TNSS scale
CONT-P-0005	TNSS_SAR	TF_E53	desloratadine_5_od	placebo	Same as model scale	Same as model SE	-1.180	0.319	day 14	Table 2, Total Nasal Symptoms change at day 14
CONT-P-0006	TNSS_SAR	TF_NCT02320396	desloratadine_5_od	placebo	Same as model scale	Same as model SE	-0.622	0.121	day 15	ClinicalTrials.gov outcome table: adjusted mean difference in TNSS
CONT-P-0007	TNSS_SAR	TF_E38	cetirizine_10_od	placebo	Same as model scale	Same as model SE	-1.000	0.230	2-week average	Efficacy results, reflective TNSS, matched oral placebo
CONT-P-0008	TNSS_SAR	TF_E49	loratadine_10_od	placebo	Same as model scale	Same as model SE	-1.040	0.214	2-week average	Table 2, daytime nasal symptoms
CONT-P-0009	TNSS_SAR	TF_E52	loratadine_10_od	placebo	Same as model scale	Same as model SE	-0.960	0.143	2-week average	Table 3, daytime nasal symptoms
CONT-P-0010	TNSS_SAR	TF_E62	loratadine_10_od	placebo	Same as model scale	Same as model SE	-0.760	0.184	2-week average	Table 4, daytime nasal symptoms
CONT-P-0011	TNSS_SAR	TF_E63	loratadine_10_od	placebo	Same as model scale	Same as model SE	-0.880	0.184	weeks 1-2 average	Table 4, daytime nasal symptoms, 2 weeks
CONT-P-0012	TNSS_SAR	TF_E29_FFU109045	fexofenadine_180_od	placebo	Same as model scale	Same as model SE	-0.300	0.190	2-week average	Table 2, Study 1 reflective TNSS, FEX versus placebo
CONT-P-0013	TNSS_SAR	TF_E29_FFU109047	fexofenadine_180_od	placebo	Same as model scale	Same as model SE	0.200	0.220	2-week average	Table 2, Study 2 reflective TNSS, FEX versus placebo
CONT-P-0014	TNSS_SAR	TF_E55_NCT01241214	cetirizine_10_od	placebo	Same as model scale	Same as model SE	-0.840	0.286	2-week average	Primary efficacy text, Phase 2 rDNSS cetirizine vs placebo
CONT-P-0015	TNSS_PAR	TF_E21	rupatadine_10_od	placebo	Same as model scale	Same as model SE	-0.960	0.389	week 4	E21_TNSS; Table 2, morning reflective 4NTSS change at 4 weeks
CONT-P-0016	TNSS_PAR	TF_E21	rupatadine_20_od	placebo	Same as model scale	Same as model SE	-1.220	0.405	week 4	E21_TNSS; Table 2, morning reflective 4NTSS change at 4 weeks
CONT-P-0017	TNSS_PAR	TF_E21	cetirizine_10_od	placebo	Same as model scale	Same as model SE	-0.870	0.405	week 4	E21_TNSS; Table 2, morning reflective 4NTSS change at 4 weeks
CONT-P-0018	TNSS_PAR	TF_E25	cetirizine_10_od	placebo	Same as model scale	Same as model SE	-0.400	0.184	weeks 1-4 average	Abstract/results, DNSS over initial 4 weeks
CONT-P-0019	TOSS_SAR	TF_E42	bilastine_20_od	placebo	Same as model scale	Same as model SE	-0.740	0.178	day 14	E42_TOSS; Table 4: ocular NNSS = tearing, redness, itching, each 0-3; total 0-9; construct-equivalent to TOSS
CONT-P-0020	TOSS_SAR	TF_E42	cetirizine_10_od	placebo	Same as model scale	Same as model SE	-0.850	0.185	day 14	E42_TOSS; Table 4: ocular NNSS = tearing, redness, itching, each 0-3; total 0-9; construct-equivalent to TOSS

ID	Structure	Trial unit	Treatment	Control	Source effect	Source SE	Model effect	Model SE	Time	Covariance/source
CONT-P-0021	TOSS_SAR	TF_E29_FFU109045	fexofenadine_180_od	placebo	Same as model scale	Same as model SE	-0.200	0.150	2-week average	Table 2, Study 1 reflective TOSS, FEX versus placebo
CONT-P-0022	TOSS_SAR	TF_E29_FFU109047	fexofenadine_180_od	placebo	Same as model scale	Same as model SE	0.100	0.180	2-week average	Table 2, Study 2 reflective TOSS, FEX versus placebo
CONT-P-0023	RQLQ_SAR	TF_E31	bilastine_20_od	placebo	Same as model scale	Same as model SE	-0.300	0.114	day 14	E31_RQLQ; Table 3, total RQLQ change at day 14
CONT-P-0024	RQLQ_SAR	TF_E31	desloratadine_5_od	placebo	Same as model scale	Same as model SE	-0.300	0.113	day 14	E31_RQLQ; Table 3, total RQLQ change at day 14
CONT-P-0025	RQLQ_SAR	TF_E53	desloratadine_5_od	placebo	Same as model scale	Same as model SE	-0.480	0.122	day 14	Table 3, total RQLQ change at day 14
CONT-P-0026	RQLQ_SAR	TF_A00430_NCT00621959	levocetirizine_5_od	placebo	Same as model scale	Same as model SE	-0.080	0.099	week 2	Table 1b, adjusted RQLQ difference
CONT-P-0027	RQLQ_SAR	TF_A00431_NCT00653224	levocetirizine_5_od	placebo	Same as model scale	Same as model SE	-0.280	0.107	week 2	Results, final RQLQ means and SDs
CONT-P-0028	RQLQ_SAR	TF_E62	loratadine_10_od	placebo	Same as model scale	Same as model SE	-0.320	0.097	week 2	Table 4, RQLQ mean
CONT-P-0029	RQLQ_SAR	TF_E63	loratadine_10_od	placebo	Same as model scale	Same as model SE	-0.300	0.087	week 2	Table 4, RQLQ, 2 weeks
CONT-P-0030	RQLQ_PER	TF_E19	desloratadine_5_od	placebo	Same as model scale	Same as model SE	-0.300	0.095	day 28	Table 3, total RQLQ score at day 28
CONT-P-0031	RQLQ_PER	TF_XPERT	levocetirizine_5_od	placebo	Same as model scale	Same as model SE	-0.480	0.097	week 4	Table III, adjusted RQLQ difference at 4 weeks

Table S28B. Binary model-ready arm-level data.

ID	Structure	Canonical trial unit	Treatment	Events	N	Period	Source/qualification
BIN-0001	ANY_AE_PAR	TF_E15	Mizolastine 10 mg once daily	56	131	28 days	Safety results, pp.5-6; Exact reported integer count and denominator; VERIFIED_COMPLETE
BIN-0002	ANY_AE_PAR	TF_E15	Placebo oral	58	122	28 days	Safety results, pp.5-6; Exact reported integer count and denominator; VERIFIED_COMPLETE
BIN-0003	ANY_AE_PAR	TF_E17	Ebastine 10 mg once daily	41	103	28 days	Adverse-events table/section; Integer count uniquely reconstructed from the reported percentage and randomized arm denominator; four-week randomized double-blind treatment period.; VERIFIED_COMPLETE
BIN-0004	ANY_AE_PAR	TF_E17	Ebastine 20 mg once daily	38	111	28 days	Adverse-events table/section; Integer count uniquely reconstructed from the reported percentage and randomized arm denominator; four-week randomized double-blind treatment period.; VERIFIED_COMPLETE
BIN-0005	ANY_AE_PAR	TF_E17	Loratadine 10 mg once daily	43	103	28 days	Adverse-events table/section; Integer count uniquely reconstructed from the reported percentage and randomized arm denominator; four-week randomized double-blind treatment period.; VERIFIED_COMPLETE
BIN-0006	ANY_AE_PAR	TF_E18	Mizolastine 10 mg once daily	56	140	28 days	Safety results; Exact reported integer count and denominator; VERIFIED_COMPLETE
BIN-0007	ANY_AE_PAR	TF_E18	Loratadine 10 mg once daily	48	140	28 days	Safety results; Exact reported integer count and denominator; VERIFIED_COMPLETE

ID	Structure	Canonical trial unit	Treatment	Events	N	Period	Source/qualification
BIN-0008	ANY_AE_PAR	TF_E18	Placebo oral	63	145	28 days	Safety results; Exact reported integer count and denominator; VERIFIED_COMPLETE
BIN-0009	ANY_AE_PAR	TF_E22	Bepotastine salicylate controlled-release 20 mg once daily	26	133	28 days	Safety results; Exact reported integer count and denominator; regimen node verified as controlled-release 20 mg once daily; VERIFIED_COMPLETE
BIN-0010	ANY_AE_PAR	TF_E22	Placebo oral	17	138	28 days	Safety results; Exact reported integer count and denominator; VERIFIED_COMPLETE
BIN-0011	ANY_AE_PAR	TF_E26	Desloratadine 5 mg once daily	87	337	28 days	Safety results; exact integer reconstruction from percentages; Exact integer uniquely reconstructed from reported percentage and denominator; verified by back-calculation; VERIFIED_COMPLETE
BIN-0012	ANY_AE_PAR	TF_E26	Placebo oral	107	339	28 days	Safety results; exact integer reconstruction from percentages; Exact integer uniquely reconstructed from reported percentage and denominator; verified by back-calculation; VERIFIED_COMPLETE
BIN-0013	ANY_AE_PAR	TF_E27	Rupatadine 10 mg once daily	28	108	28 days	Safety results; Exact reported integer count and denominator; VERIFIED_COMPLETE
BIN-0014	ANY_AE_PAR	TF_E27	Bepotastine besilate 10 mg twice daily	31	109	28 days	Safety results; Exact reported integer count and denominator; regimen node verified as 10 mg twice daily (20 mg/day); VERIFIED_COMPLETE
BIN-0015	ANY_AE_PAR	TF_E27	Placebo oral	21	110	28 days	Safety results; Exact reported integer count and denominator; VERIFIED_COMPLETE
BIN-0016	ANY_AE_PAR	TF_E71	Cetirizine 10 mg once daily	51	158	28 days	Safety results; Exact reported integer count and denominator; VERIFIED_COMPLETE
BIN-0017	ANY_AE_PAR	TF_E71	Placebo oral	50	162	28 days	Safety results; Exact reported integer count and denominator; VERIFIED_COMPLETE
BIN-0018	ANY_AE_PAR	TF_E81	Bilastine 20 mg once daily	90	214	28 days	Safety results; Exact reported integer count and denominator; VERIFIED_COMPLETE
BIN-0019	ANY_AE_PAR	TF_E81	Cetirizine 10 mg once daily	72	217	28 days	Safety results; Exact reported integer count and denominator; VERIFIED_COMPLETE
BIN-0020	ANY_AE_PAR	TF_E81	Placebo oral	97	219	28 days	Safety results; Exact reported integer count and denominator; VERIFIED_COMPLETE
BIN-0021	WDAE_PAR	TF_E15	Mizolastine 10 mg once daily	4	131	28 days	Withdrawals due to adverse events; Exact reported integer count and denominator; VERIFIED_COMPLETE
BIN-0022	WDAE_PAR	TF_E15	Placebo oral	5	122	28 days	Withdrawals due to adverse events; Exact reported integer count and denominator; VERIFIED_COMPLETE
BIN-0023	WDAE_PAR	TF_E17	Ebastine 10 mg once daily	1	103	28 days	Withdrawals due to adverse events; Explicit integer withdrawal count during the four-week randomized double-blind treatment period.; VERIFIED_COMPLETE
BIN-0024	WDAE_PAR	TF_E17	Ebastine 20 mg once daily	0	111	28 days	Withdrawals due to adverse events; Explicit integer withdrawal count during the four-week randomized double-blind treatment period.; VERIFIED_COMPLETE
BIN-0025	WDAE_PAR	TF_E17	Loratadine 10 mg once daily	2	103	28 days	Withdrawals due to adverse events; Explicit integer withdrawal count during the four-week randomized double-blind treatment period.; VERIFIED_COMPLETE
BIN-0026	WDAE_PAR	TF_E18	Mizolastine 10 mg once daily	2	140	28 days	Withdrawals due to adverse events; Exact reported integer count and denominator; VERIFIED_COMPLETE
BIN-0027	WDAE_PAR	TF_E18	Loratadine 10 mg once daily	5	140	28 days	Withdrawals due to adverse events; Exact reported integer count and denominator; VERIFIED_COMPLETE

ID	Structure	Canonical trial unit	Treatment	Events	N	Period	Source/qualification
BIN-0028	WDAE_PAR	TF_E18	Placebo oral	1	145	28 days	Withdrawals due to adverse events; Exact reported integer count and denominator; VERIFIED_COMPLETE
BIN-0029	WDAE_PAR	TF_E20	Rupatadine 10 mg once daily	1	73	28 days	Safety narrative/Table 3 denominators; Exact reported integer count and denominator; VERIFIED_COMPLETE
BIN-0030	WDAE_PAR	TF_E20	Rupatadine 20 mg once daily	1	71	28 days	Safety narrative/Table 3 denominators; Exact reported integer count and denominator; VERIFIED_COMPLETE
BIN-0031	WDAE_PAR	TF_E20	Loratadine 10 mg once daily	1	70	28 days	Safety narrative/Table 3 denominators; Exact reported integer count and denominator; VERIFIED_COMPLETE
BIN-0032	WDAE_PAR	TF_E20	Placebo oral	1	69	28 days	Safety narrative/Table 3 denominators; Exact reported integer count and denominator; VERIFIED_COMPLETE
BIN-0033	WDAE_PAR	TF_E27	Rupatadine 10 mg once daily	0	108	28 days	Withdrawals due to adverse events; Exact reported integer count and denominator; VERIFIED_COMPLETE
BIN-0034	WDAE_PAR	TF_E27	Bepotastine besilate 10 mg twice daily	1	109	28 days	Withdrawals due to adverse events; Exact reported integer count and denominator; regimen node verified as 10 mg twice daily (20 mg/day); VERIFIED_COMPLETE
BIN-0035	WDAE_PAR	TF_E27	Placebo oral	2	110	28 days	Withdrawals due to adverse events; Exact reported integer count and denominator; VERIFIED_COMPLETE
BIN-0036	WDAE_PAR	TF_E71	Cetirizine 10 mg once daily	2	158	28 days	Withdrawals due to adverse events; Exact reported integer count and denominator; VERIFIED_COMPLETE
BIN-0037	WDAE_PAR	TF_E71	Placebo oral	4	162	28 days	Withdrawals due to adverse events; Exact reported integer count and denominator; VERIFIED_COMPLETE
BIN-0038	WDAE_PAR	TF_E81	Bilastine 20 mg once daily	2	214	28 days	Withdrawals due to adverse events; Exact reported integer count and denominator; VERIFIED_COMPLETE
BIN-0039	WDAE_PAR	TF_E81	Cetirizine 10 mg once daily	2	217	28 days	Withdrawals due to adverse events; Exact reported integer count and denominator; VERIFIED_COMPLETE
BIN-0040	WDAE_PAR	TF_E81	Placebo oral	0	219	28 days	Withdrawals due to adverse events; Exact reported integer count and denominator; VERIFIED_COMPLETE
BIN-0041	ANY_AE_SAR	TF_E31	Bilastine 20 mg once daily	66	233	14 days	Adverse-events results; Exact reported integer count and denominator; VERIFIED_COMPLETE
BIN-0042	ANY_AE_SAR	TF_E31	Desloratadine 5 mg once daily	79	242	14 days	Adverse-events results; Exact reported integer count and denominator; VERIFIED_COMPLETE
BIN-0043	ANY_AE_SAR	TF_E31	Placebo oral	62	245	14 days	Adverse-events results; Exact reported integer count and denominator; VERIFIED_COMPLETE
BIN-0044	ANY_AE_SAR	TF_E42	Bilastine 20 mg once daily	56	227	14 days	Adverse-events results; Exact reported integer count and denominator; VERIFIED_COMPLETE
BIN-0045	ANY_AE_SAR	TF_E42	Cetirizine 10 mg once daily	82	228	14 days	Adverse-events results; Exact reported integer count and denominator; VERIFIED_COMPLETE
BIN-0046	ANY_AE_SAR	TF_E42	Placebo oral	67	226	14 days	Adverse-events results; Exact reported integer count and denominator; VERIFIED_COMPLETE
BIN-0047	ANY_AE_SAR	TF_E51	Rupatadine 10 mg once daily	42	298	14 days	Adverse-events results; Exact reported integer count and denominator; VERIFIED_COMPLETE
BIN-0048	ANY_AE_SAR	TF_E51	Rupatadine 20 mg once daily	45	300	14 days	Adverse-events results; Exact reported integer count and denominator; VERIFIED_COMPLETE

ID	Structure	Canonical trial unit	Treatment	Events	N	Period	Source/qualification
BIN-0049	ANY_AE_SAR	TF_E51	Placebo oral	20	302	14 days	Adverse-events results; Exact reported integer count and denominator; VERIFIED_COMPLETE
BIN-0050	ANY_AE_SAR	TF_E55_NCT01241214	Cetirizine 10 mg once daily	6	96	14 days	Phase 2 safety results; Exact reported integer count and denominator; VERIFIED_COMPLETE
BIN-0051	ANY_AE_SAR	TF_E55_NCT01241214	Placebo oral	13	96	14 days	Phase 2 safety results; Exact reported integer count and denominator; VERIFIED_COMPLETE
BIN-0052	ANY_AE_SAR	TF_E74	Fexofenadine 120 mg once daily	86	287	14 days	Table II; Exact reported integer count and denominator; VERIFIED_COMPLETE
BIN-0053	ANY_AE_SAR	TF_E74	Fexofenadine 180 mg once daily	86	283	14 days	Table II; Exact reported integer count and denominator; VERIFIED_COMPLETE
BIN-0054	ANY_AE_SAR	TF_E74	Placebo oral	88	293	14 days	Table II; Exact reported integer count and denominator; VERIFIED_COMPLETE
BIN-0055	ANY_AE_SAR	TF_A00430_NCT00621959	Levocetirizine 5 mg once daily	72	301	14 days	Safety results; Exact integer uniquely reconstructed from reported percentage and denominator; verified by back-calculation; VERIFIED_COMPLETE
BIN-0056	ANY_AE_SAR	TF_A00430_NCT00621959	Placebo oral	72	295	14 days	Safety results; Exact integer uniquely reconstructed from reported percentage and denominator; verified by back-calculation; VERIFIED_COMPLETE
BIN-0057	ANY_AE_SAR	TF_E79	Fexofenadine 120 mg once daily	39	232	14 days	Table 4 / safety results; Exact reported integer count and denominator; VERIFIED_COMPLETE
BIN-0058	ANY_AE_SAR	TF_E79	Loratadine 10 mg once daily	40	228	14 days	Table 4 / safety results; Exact reported integer count and denominator; VERIFIED_COMPLETE
BIN-0059	ANY_AE_SAR	TF_E79	Placebo oral	33	225	14 days	Table 4 / safety results; Exact reported integer count and denominator; VERIFIED_COMPLETE
BIN-0060	ANY_AE_SAR	TF_E33	Loratadine 10 mg once daily	102	217	14 days	Table IV; Exact reported integer count and denominator; VERIFIED_COMPLETE
BIN-0061	ANY_AE_SAR	TF_E33	Placebo oral	100	216	14 days	Table IV; Exact reported integer count and denominator; VERIFIED_COMPLETE
BIN-0062	ANY_AE_SAR	TF_A00431_NCT00653224	Levocetirizine 5 mg once daily	41	285	14 days	Safety results; Exact reported integer count and denominator; VERIFIED_COMPLETE
BIN-0063	ANY_AE_SAR	TF_A00431_NCT00653224	Placebo oral	54	293	14 days	Safety results; Exact reported integer count and denominator; VERIFIED_COMPLETE
BIN-0064	WDAE_SAR	TF_E31	Bilastine 20 mg once daily	1	233	14 days	Withdrawals due to adverse events; Exact reported integer count and denominator; VERIFIED_COMPLETE
BIN-0065	WDAE_SAR	TF_E31	Desloratadine 5 mg once daily	2	242	14 days	Withdrawals due to adverse events; Exact reported integer count and denominator; VERIFIED_COMPLETE
BIN-0066	WDAE_SAR	TF_E31	Placebo oral	5	245	14 days	Withdrawals due to adverse events; Exact reported integer count and denominator; VERIFIED_COMPLETE
BIN-0067	WDAE_SAR	TF_E42	Bilastine 20 mg once daily	1	227	14 days	Withdrawals due to adverse events; Exact reported integer count and denominator; VERIFIED_COMPLETE
BIN-0068	WDAE_SAR	TF_E42	Cetirizine 10 mg once daily	0	228	14 days	Withdrawals due to adverse events; Exact reported integer count and denominator; VERIFIED_COMPLETE
BIN-0069	WDAE_SAR	TF_E42	Placebo oral	3	226	14 days	Withdrawals due to adverse events; Exact reported integer count and denominator; VERIFIED_COMPLETE

ID	Structure	Canonical trial unit	Treatment	Events	N	Period	Source/qualification
BIN-0070	WDAE_SAR	TF_E51	Rupatadine 10 mg once daily	0	298	14 days	No adverse events resulted in premature discontinuation; Exact reported integer count and denominator; VERIFIED_COMPLETE
BIN-0071	WDAE_SAR	TF_E51	Rupatadine 20 mg once daily	0	300	14 days	No adverse events resulted in premature discontinuation; Exact reported integer count and denominator; VERIFIED_COMPLETE
BIN-0072	WDAE_SAR	TF_E51	Placebo oral	0	302	14 days	No adverse events resulted in premature discontinuation; Exact reported integer count and denominator; VERIFIED_COMPLETE
BIN-0073	WDAE_SAR	TF_A00430_NCT00621959	Levocetirizine 5 mg once daily	2	301	14 days	Withdrawals due to adverse events; Exact reported integer count and denominator; VERIFIED_COMPLETE
BIN-0074	WDAE_SAR	TF_A00430_NCT00621959	Placebo oral	4	295	14 days	Withdrawals due to adverse events; Exact reported integer count and denominator; VERIFIED_COMPLETE
BIN-0075	WDAE_SAR	TF_E84	Desloratadine 5 mg once daily	2	109	14 days	Safety results; Exact reported event count and verified safety-population denominator (2/109).; VERIFIED_COMPLETE
BIN-0076	WDAE_SAR	TF_E84	Placebo oral	0	110	14 days	Safety results; Exact reported integer count and denominator; VERIFIED_COMPLETE
BIN-0077	WDAE_SAR	TF_E38	Cetirizine 10 mg once daily	0	170	14 days	Matched oral placebo safety analysis; Exact reported integer count and denominator; VERIFIED_COMPLETE
BIN-0078	WDAE_SAR	TF_E38	Placebo oral	1	171	14 days	Matched oral placebo safety analysis; Exact reported integer count and denominator; VERIFIED_COMPLETE
BIN-0079	WDAE_SAR	TF_E52	Loratadine 10 mg once daily	8	602	14 days	Safety table/narrative; Primary treatment-emergent WDAE: 8/602; excludes one discontinuation attributed to an event that began before randomization. Assignment-estimand sensitivity uses 9/602.; VERIFIED_COMPLETE
BIN-0080	WDAE_SAR	TF_E52	Placebo oral	1	352	14 days	Safety table/narrative; Exact reported integer count and denominator; VERIFIED_COMPLETE
BIN-0081	WDAE_SAR	TF_E29_FFU109045	Fexofenadine 180 mg once daily	6	311	14 days	Study FFU109045 Table 1; Exact reported integer count and denominator; VERIFIED_COMPLETE
BIN-0082	WDAE_SAR	TF_E29_FFU109045	Placebo oral	1	313	14 days	Study FFU109045 Table 1; Exact reported integer count and denominator; VERIFIED_COMPLETE
BIN-0083	WDAE_SAR	TF_E29_FFU109047	Fexofenadine 180 mg once daily	1	227	14 days	Study FFU109047 Table 1; Exact reported integer count and denominator; VERIFIED_COMPLETE
BIN-0084	WDAE_SAR	TF_E29_FFU109047	Placebo oral	1	229	14 days	Study FFU109047 Table 1; Exact reported integer count and denominator; VERIFIED_COMPLETE
BIN-0085	WDAE_SAR	TF_E55_NCT01241214	Cetirizine 10 mg once daily	2	96	14 days	Phase 2 safety results; Exact reported integer count and denominator; VERIFIED_COMPLETE
BIN-0086	WDAE_SAR	TF_E55_NCT01241214	Placebo oral	0	96	14 days	Phase 2 safety results; Exact reported integer count and denominator; VERIFIED_COMPLETE
BIN-0087	WDAE_SAR	TF_E55_NCT01484119	Cetirizine 10 mg once daily	1	210	14 days	Phase III safety narrative: randomized treatment and subsequent placebo run-out; One discontinuation (bronchitis) occurred during randomized double-blind cetirizine treatment. A later pregnancy discontinuation during single-blind placebo run-out is outside the primary estimand.; VERIFIED_COMPLETE
BIN-0088	WDAE_SAR	TF_E55_NCT01484119	Placebo oral	5	210	14 days	Phase 3 safety results; Exact reported integer count and denominator; VERIFIED_COMPLETE
BIN-0089	WDAE_SAR	TF_A00431_NCT00653224	Levocetirizine 5 mg once daily	1	285	14 days	Safety results; Exact reported integer count and denominator; VERIFIED_COMPLETE
BIN-0090	WDAE_SAR	TF_A00431_NCT00653224	Placebo oral	2	293	14 days	Safety results; Exact reported integer count and denominator; VERIFIED_COMPLETE
BIN-0091	WDAE_SAR	TF_E35_FALL2000_MONT_LOR_PBO	Loratadine 10 mg once daily	2	171	14 days	Fall 2000 subgroup safety results; Exact reported integer count and denominator; VERIFIED_COMPLETE

ID	Structure	Canonical trial unit	Treatment	Events	N	Period	Source/qualification
BIN-0092	WDAE_SAR	TF_E35_FALL2000_MONT_LOR_PBO	Placebo oral	4	333	14 days	Fall 2000 subgroup safety results; Exact reported integer count and denominator; VERIFIED_COMPLETE
BIN-0093	WDAE_SAR	TF_NCT02320396	Desloratadine 5 mg once daily	2	223	14 days	ClinicalTrials.gov results, withdrawals due to AE; Exact reported integer count and denominator; VERIFIED_COMPLETE
BIN-0094	WDAE_SAR	TF_NCT02320396	Placebo oral	2	225	14 days	ClinicalTrials.gov results, withdrawals due to AE; Exact reported integer count and denominator; VERIFIED_COMPLETE
BIN-0095	WDAE_SAR	TF_E79	Fexofenadine 120 mg once daily	3	232	14 days	Table 4 / safety results; Exact reported integer count and denominator; VERIFIED_COMPLETE
BIN-0096	WDAE_SAR	TF_E79	Loratadine 10 mg once daily	5	228	14 days	Table 4 / safety results; Exact reported integer count and denominator; VERIFIED_COMPLETE
BIN-0097	WDAE_SAR	TF_E79	Placebo oral	7	225	14 days	Table 4 / safety results; Exact reported integer count and denominator; VERIFIED_COMPLETE
BIN-0098	WDAE_SAR	TF_E33	Loratadine 10 mg once daily	1	217	14 days	Safety results; Exact reported integer count and denominator; VERIFIED_COMPLETE
BIN-0099	WDAE_SAR	TF_E33	Placebo oral	0	216	14 days	Safety results; Source-primary explicit zero: the narrative allocates all 12 adverse-event discontinuations to combination (3), loratadine (1), and pseudoephedrine (8), leaving none in placebo. A legacy secondary extraction of 2/216 is retained only as a source-conflict sensitivity.; SOURCE_PRIMARY_WITH_CONFLICT_SENSITIVITY
BIN-0100	WDAE_SAR	TF_E50	Cetirizine 10 mg once daily	1	201	14 days	Safety results; Exact reported integer count and denominator; VERIFIED_COMPLETE
BIN-0101	WDAE_SAR	TF_E50	Placebo oral	6	197	14 days	Safety results; Exact reported integer count and denominator; VERIFIED_COMPLETE
BIN-0107	WDAE_PAR	TF_E26	Desloratadine 5 mg once daily	11	337	28 days	Safety results: discontinuations due to adverse events reported as 3.3% vs 2.1%; Uniquely reconstructed integer count from the reported one-decimal percentage and randomized denominator; back-calculation is unique at the stated precision.; R1_R4_AUDITED_FINAL_v55_8
BIN-0108	WDAE_PAR	TF_E26	Placebo oral	7	339	28 days	Safety results: discontinuations due to adverse events reported as 3.3% vs 2.1%; Uniquely reconstructed integer count from the reported one-decimal percentage and randomized denominator; back-calculation is unique at the stated precision.; R1_R4_AUDITED_FINAL_v55_8

S29. Characteristics of Model-Ready Studies and Participants

Table S29. Quantitative fields are NR where not reported or not recovered; no values were inferred.

No.	Study/unit	Cat.	Setting	Age eligibility	Arm N	Mean age (SD)	Female %	Baseline/entry	Duration	Sponsor
1	Mansfield et al. (2010); TF_A00430_NCT00621959	SAR	Multicenter	Adults; exact range in source report	301 / 295	NR in audited extraction	NR in audited extraction	NR unless described qualitatively in Baseline burden/entry	14 days; week 2	UCB
2	Segall et al. (2010); TF_A00431_NCT00653224	SAR	United States; multicenter	18-65 years	287 / 293	NR in audited extraction	NR in audited extraction	NR unless described qualitatively in Baseline burden/entry	14 days; week 2	UCB Inc and sanofi-aventis
3	Bachert et al. (1998); TF_E15	PAR	Germany and United Kingdom; 22 centers	Adults; exact eligibility in source report	133 / 124	NR in audited extraction	NR in audited extraction	NR unless described qualitatively in Baseline burden/entry	28 days	Synthélabo/Synthelabo Groupe involvement reported
4	Davies et al. (1998); TF_E17	PAR	European multicenter study	12-68 years	NR	NR in audited extraction	NR in audited extraction	NR unless described qualitatively in Baseline burden/entry	28 days	NR
5	Frèche et al. (2002); TF_E18	PAR	Europe; 41 centers	>=18 years	146 / 141 / 141	NR in audited extraction	NR in audited extraction	NR unless described qualitatively in Baseline burden/entry	28 days	NR
6	Holmberg et al. (2009); TF_E19	PER	Sweden/multicenter	Adults; exact eligibility in source	293 / 291	NR in audited extraction	NR in audited extraction	NR unless described qualitatively in Baseline burden/entry	28 days	Schering-Plough AB involvement reported
7	Kowalski et al. (2009); TF_E20	PAR	Europe and Brazil; multicenter	Adults	69 / 70 / 73 / 71	NR in audited extraction	NR in audited extraction	NR unless described qualitatively in Baseline burden/entry	28 days	Uriach / sponsor reported in source
8	Marmouz et al. (2011); TF_E21	PAR	Multicenter	>=18 years	NR	NR in audited extraction	NR in audited extraction	NR unless described qualitatively in Baseline burden/entry	28 days	NR
9	Mo et al. (2021); TF_E22	PAR	South Korea; multicenter	19-65 years	134 / 138	NR in audited extraction	NR in audited extraction	NR unless described qualitatively in Baseline burden/entry	28 days	Hanlim Pharm Co., Ltd.
10	Philip et al. (2007); TF_E25	PAR	Multicenter; perennial rhinitis trial	Adults; exact eligibility in source	Cetirizine 368; placebo 367 (eligible arms; n=735)	NR in audited extraction	NR in audited extraction	NR unless described qualitatively in Baseline burden/entry	28 days	Merck Research Laboratories
11	Simons et al. (2003); TF_E26	PAR	Multicenter	Adults/adolescents; exact eligibility in source	NR	NR in audited extraction	NR in audited extraction	NR unless described qualitatively in Baseline burden/entry	28 days	Schering-Plough
12	Won et al. (2024); TF_E27	PAR	South Korea; nine tertiary centers	Adults	NR	NR in audited extraction	NR in audited extraction	NR unless described qualitatively in Baseline burden/entry	28 days	NR
13	Andrews et al. (2009), FFU109045; TF_E29_FFU109045	SAR	United States; multicenter	Adults; exact eligibility in source report	See trial-specific rows: FFU109045 eligible n=624; FFU109047 eligible n=456	NR in audited extraction	NR in audited extraction	NR unless described qualitatively in Baseline burden/entry	14 days; 2-week average	GlaxoSmithKline

No.	Study/unit	Cat.	Setting	Age eligibility	Arm N	Mean age (SD)	Female %	Baseline/entry	Duration	Sponsor
14	Andrews et al. (2009), FFU109047; TF_E29_FFU109047	SAR	United States; multicenter	Adults; exact eligibility in source report	See trial-specific rows: FFU109045 eligible n=624; FFU109047 eligible n=456	NR in audited extraction	NR in audited extraction	NR unless described qualitatively in Baseline burden/entry	14 days; 2-week average	GlaxoSmithKline
15	Bachert et al. (2009); TF_E31	SAR	Europe; multicenter	12-70 years	NR	NR in audited extraction	NR in audited extraction	NR unless described qualitatively in Baseline burden/entry	14 days; day 14	FAES Farma
16	Bronsky et al. (1995); TF_E33	SAR	United States; multicenter	Adults/adolescents; exact eligibility in source	Loratadine 217; placebo 216	NR in audited extraction	NR in audited extraction	NR unless described qualitatively in Baseline burden/entry	14 days	NR
17	Ford et al. (2015); TF_E38	SAR	United States; multicenter	>=12 years	Cetirizine 170; placebo 171	NR in audited extraction	NR in audited extraction	NR unless described qualitatively in Baseline burden/entry	14 days; 2-week average	GlaxoSmithKline
18	Kuna et al. (2009); TF_E42	SAR	Europe; 61 centers; multicenter	12-70 years	NR	NR in audited extraction	NR in audited extraction	NR unless described qualitatively in Baseline burden/entry	14 days; day 14	FAES Farma (study sponsor/support reported in source)
19	Nayak et al. (2002), Fall 1999 trial; TF_E49	SAR	United States; multicenter fall pollen study	15-85 years	Loratadine 301; placebo 149	NR in audited extraction	NR in audited extraction	NR unless described qualitatively in Baseline burden/entry	14 days; 2-week average	Merck Research Laboratories
20	Noonan et al. (2003); TF_E50	SAR	United States; multicenter	Adults; exact eligibility in source	NR	NR in audited extraction	NR in audited extraction	NR unless described qualitatively in Baseline burden/entry	14 days	NR
21	Okubo et al. (2019); TF_E51	SAR	Japan; multicenter	12-65 years	NR	NR in audited extraction	NR in audited extraction	NR unless described qualitatively in Baseline burden/entry	14 days; week 2	Teikoku Seiyaku / sponsor support reported in source
22	Philip et al. (2002); TF_E52	SAR	Multicenter	15-81 years	Loratadine 602; placebo 352	NR in audited extraction	NR in audited extraction	NR unless described qualitatively in Baseline burden/entry	14 days; 2-week average	Merck Research Laboratories
23	Pradalier et al. (2007); TF_E53	SAR	Europe; multicenter	>=18 years	264 / 270	NR in audited extraction	NR in audited extraction	NR unless described qualitatively in Baseline burden/entry	14 days; day 14	Schering-Plough
24	Ratner et al. (2017), NCT01241214; TF_E55_NCT01241214	SAR	Texas, USA; seven centers	Phase 2 mean 41.6-43.4 years; Phase 3 mean 37.5-40.7 years	NCT01241214: 96/96; NCT01484119: 210/210	NR in audited extraction	NR in audited extraction	NR unless described qualitatively in Baseline burden/entry	14 days; 2-week average	Actelion Pharmaceuticals / sponsor reported in source
25	Ratner et al. (2017), NCT01484119; TF_E55_NCT01484119	SAR	Texas, USA; seven centers	Phase 2 mean 41.6-43.4 years; Phase 3 mean 37.5-40.7 years	NCT01241214: 96/96; NCT01484119: 210/210	NR in audited extraction	NR in audited extraction	NR unless described qualitatively in Baseline burden/entry	14 days	Actelion Pharmaceuticals / sponsor reported in source
26	Van Adelsberg et al. (2003a); TF_E62	SAR	United States; multicenter spring-pollen study	15-85 years	Loratadine 171; placebo 521	NR in audited extraction	NR in audited extraction	NR unless described qualitatively in Baseline burden/entry	14 days; 2-week average	Merck Research Laboratories

No.	Study/unit	Cat.	Setting	Age eligibility	Arm N	Mean age (SD)	Female %	Baseline/entry	Duration	Sponsor
27	Van Adelsberg et al. (2003b); TF_E63	SAR	North America; multicenter fall-pollen study	15-85 years	Loratadine 180; placebo 451	NR in audited extraction	NR in audited extraction	NR unless described qualitatively in Baseline burden/entry	14 days; weeks 1-2 average	Merck Research Laboratories
28	Skoner et al. (2014); TF_E71	PAR	United States; 15 centers outside pollen season	18-65 years	NR	NR in audited extraction	NR in audited extraction	NR unless described qualitatively in Baseline burden/entry	28 days	Pfizer, Inc.
29	Casale et al. (1999); TF_E74	SAR	United States; multicenter	Adults/adolescents; exact eligibility in source	NR	NR in audited extraction	NR in audited extraction	NR unless described qualitatively in Baseline burden/entry	14 days	Hoechst Marion Roussel
30	Van Cauwenberge et al. (2000); TF_E79	SAR	Nine European countries and South Africa; multinational multicenter	12-75 years	NR	NR in audited extraction	NR in audited extraction	NR unless described qualitatively in Baseline burden/entry	14 days	NR
31	Sastre et al. (2012); TF_E81	PAR	Europe; multicenter	Adults/adolescents; exact eligibility in source	NR	NR in audited extraction	NR in audited extraction	NR unless described qualitatively in Baseline burden/entry	28 days	FAES Farma
32	Meltzer et al. (2006); TF_E84	SAR	United States; multicenter	18-60 years	109 / 111	NR in audited extraction	NR in audited extraction	NR unless described qualitatively in Baseline burden/entry	14 days	Integrated Therapeutics Group/Schering-Plough
33	Chervinsky et al. (2004), Study 2 (Fall 2000); TF_E35_FALL2000_MONT_LOR_PBO	SAR	North America; pooled fall-pollen studies	NR in pooled report	Loratadine 171; placebo 333	NR in audited extraction	NR in audited extraction	NR unless described qualitatively in Baseline burden/entry	14 days; Fall 2000	Merck Research Laboratories
34	Organon and Co. (2014), NCT02320396; TF_NCT02320396	SAR	Japan; multicenter trial registry	Adults/adolescents per registry	224 / 225	NR in audited extraction	NR in audited extraction	NR unless described qualitatively in Baseline burden/entry	14 days; day 15	Organon/Merck Sharp & Dohme
35	Bachert et al. (2004), XPERT; TF_XPERT	PER	Multinational multicenter	Adults	278 / 273	NR in audited extraction	NR in audited extraction	NR unless described qualitatively in Baseline burden/entry	28 days; week 4	UCB

S30. Result-Specific RoB 2 Domain Assessments

Table S30. Seventy-three result-specific assessments with actual windows and adjudication rationales; full domain evidence summaries are in RoB2_Result_Level_Assessments_v1.1.csv.

ID	Structure	Trial unit	Window	D1	D2	D3	D4	D5	Overall	Brief justification
ROB2-APPRAISAL-001	ANY_AE_PAR	TF_E15	28-day randomized treatment period	Some concerns	Low	Low	Low	Some concerns	Some concerns	Overall Some concerns because D1, D5 raised Some concerns and no domain was High.
ROB2-APPRAISAL-002	ANY_AE_PAR	TF_E17	28-day randomized double-blind treatment period	Some concerns	Low	Low	Low	Some concerns	Some concerns	Overall Some concerns because D1, D5 raised Some concerns and no domain was High.
ROB2-APPRAISAL-003	ANY_AE_PAR	TF_E18	28-day randomized treatment period	Some concerns	Low	Low	Low	Some concerns	Some concerns	Overall Some concerns because D1, D5 raised Some concerns and no domain was High.
ROB2-APPRAISAL-004	ANY_AE_PAR	TF_E22	28-day randomized treatment period	Some concerns	Low	Some concerns	Low	Low	Some concerns	Overall Some concerns because D1, D3 raised Some concerns and no domain was High.
ROB2-APPRAISAL-005	ANY_AE_PAR	TF_E26	28-day randomized treatment period	Low	Low	Low	Low	Some concerns	Some concerns	Overall Some concerns because D5 raised Some concerns and no domain was High.
ROB2-APPRAISAL-006	ANY_AE_PAR	TF_E27	28-day randomized treatment period	Some concerns	Low	Low	Low	Some concerns	Some concerns	Overall Some concerns because D1, D5 raised Some concerns and no domain was High.
ROB2-APPRAISAL-007	ANY_AE_PAR	TF_E71	28-day randomized treatment period	Some concerns	Low	Low	Low	Some concerns	Some concerns	Overall Some concerns because D1, D5 raised Some concerns and no domain was High.
ROB2-APPRAISAL-008	ANY_AE_PAR	TF_E81	28-day randomized treatment period	Low	Low	Low	Low	Some concerns	Some concerns	Overall Some concerns because D5 raised Some concerns and no domain was High.
ROB2-APPRAISAL-009	ANY_AE_SAR	TF_E55_NCT01241214	14-day randomized treatment period	Some concerns	Low	Low	Low	High	High	Overall High because D5 was High.
ROB2-APPRAISAL-010	ANY_AE_SAR	TF_A00430_NCT00621959	14-day randomized treatment period	Some concerns	Low	Low	Low	Low	Some concerns	Overall Some concerns because D1 raised Some concerns and no domain was High.
ROB2-APPRAISAL-011	ANY_AE_SAR	TF_A00431_NCT00653224	14-day randomized treatment period	Some concerns	Low	Low	Low	Low	Some concerns	Overall Some concerns because D1 raised Some concerns and no domain was High.
ROB2-APPRAISAL-012	ANY_AE_SAR	TF_E31	14-day randomized treatment period	Low	Low	Low	Low	Some concerns	Some concerns	Overall Some concerns because D5 raised Some concerns and no domain was High.
ROB2-APPRAISAL-013	ANY_AE_SAR	TF_E33	14-day randomized treatment period	Some concerns	Low	Low	Low	High	High	Overall High because D5 was High.
ROB2-APPRAISAL-014	ANY_AE_SAR	TF_E42	14-day randomized treatment period	Some concerns	Low	Low	Low	Some concerns	Some concerns	Overall Some concerns because D1, D5 raised Some concerns and no domain was High.

ID	Structure	Trial unit	Window	D1	D2	D3	D4	D5	Overall	Brief justification
ROB2-APPRAISAL-015	ANY_AE_SAR	TF_E51	14-day randomized treatment period	Some concerns	Low	Some concerns	Low	Some concerns	Some concerns	Overall Some concerns because D1, D3, D5 raised Some concerns and no domain was High.
ROB2-APPRAISAL-016	ANY_AE_SAR	TF_E74	14-day randomized treatment period	Some concerns	Low	Low	Low	Some concerns	Some concerns	Overall Some concerns because D1, D5 raised Some concerns and no domain was High.
ROB2-APPRAISAL-017	ANY_AE_SAR	TF_E79	14-day randomized treatment period	Some concerns	Low	Low	Low	Some concerns	Some concerns	Overall Some concerns because D1, D5 raised Some concerns and no domain was High.
ROB2-APPRAISAL-018	RQLQ_PER	TF_E19	days 1-28 primary treatment-period result	Some concerns	Low	Low	Low	Some concerns	Some concerns	Overall Some concerns because D1, D5 raised Some concerns and no domain was High.
ROB2-APPRAISAL-019	RQLQ_PER	TF_XPERT	days 1-28 primary treatment-period result	Some concerns	Low	Low	Low	Some concerns	Some concerns	Overall Some concerns because D1, D5 raised Some concerns and no domain was High.
ROB2-APPRAISAL-020	RQLQ_SAR	TF_A00430_NCT00621959	days 1-14 primary treatment-period result	Some concerns	Low	Low	Low	Low	Some concerns	Overall Some concerns because D1 raised Some concerns and no domain was High.
ROB2-APPRAISAL-021	RQLQ_SAR	TF_A00431_NCT00653224	days 1-14 primary treatment-period result	Some concerns	Low	Low	Low	Low	Some concerns	Overall Some concerns because D1 raised Some concerns and no domain was High.
ROB2-APPRAISAL-022	RQLQ_SAR	TF_E31	days 1-14 primary treatment-period result	Low	Low	Low	Low	Some concerns	Some concerns	Overall Some concerns because D5 raised Some concerns and no domain was High.
ROB2-APPRAISAL-023	RQLQ_SAR	TF_E53	days 1-14 primary treatment-period result	Some concerns	Low	Low	Low	Some concerns	Some concerns	Overall Some concerns because D1, D5 raised Some concerns and no domain was High.
ROB2-APPRAISAL-024	RQLQ_SAR	TF_E62	days 1-14 primary treatment-period result	Some concerns	Low	Low	Low	High	High	Overall High because D5 was High.
ROB2-APPRAISAL-025	RQLQ_SAR	TF_E63	days 1-14 primary treatment-period result	Some concerns	Low	Low	Some concerns	High	High	Overall High because D5 was High.
ROB2-APPRAISAL-026	TNSS_PAR	TF_E21	days 1-28 primary treatment-period result	Some concerns	Low	Low	Low	Some concerns	Some concerns	Overall Some concerns because D1, D5 raised Some concerns and no domain was High.

ID	Structure	Trial unit	Window	D1	D2	D3	D4	D5	Overall	Brief justification
ROB2-APPRAISAL-027	TNSS_PAR	TF_E25	days 1-28 primary treatment-period result	Some concerns	Low	Low	Low	High	High	Overall High because D5 was High.
ROB2-APPRAISAL-028	TNSS_SAR	TF_E29_FFU109045	days 1-14 primary treatment-period result	Some concerns	Low	Low	Low	High	High	Overall High because D5 was High.
ROB2-APPRAISAL-029	TNSS_SAR	TF_E29_FFU109047	days 1-14 primary treatment-period result	Some concerns	Low	Low	Low	High	High	Overall High because D5 was High.
ROB2-APPRAISAL-030	TNSS_SAR	TF_E55_NCT01241214	days 1-14 primary treatment-period result	Some concerns	Low	Low	Low	High	High	Overall High because D5 was High.
ROB2-APPRAISAL-031	TNSS_SAR	TF_E38	days 1-14 primary treatment-period result	Some concerns	Low	Low	Low	High	High	Overall High because D5 was High.
ROB2-APPRAISAL-032	TNSS_SAR	TF_E42	days 1-14 primary treatment-period result	Some concerns	Low	Low	Low	Some concerns	Some concerns	Overall Some concerns because D1, D5 raised Some concerns and no domain was High.
ROB2-APPRAISAL-033	TNSS_SAR	TF_E49	days 1-14 primary treatment-period result	Some concerns	Low	Low	Low	High	High	Overall High because D5 was High.
ROB2-APPRAISAL-034	TNSS_SAR	TF_E51	days 1-14 primary treatment-period result	Some concerns	Low	Some concerns	Low	Some concerns	Some concerns	Overall Some concerns because D1, D3, D5 raised Some concerns and no domain was High.
ROB2-APPRAISAL-035	TNSS_SAR	TF_E52	days 1-14 primary treatment-period result	Some concerns	High	Some concerns	High	High	High	Overall High because D2, D4, D5 was High.
ROB2-APPRAISAL-036	TNSS_SAR	TF_E53	days 1-14 primary treatment-period result	Some concerns	Low	Low	Low	Some concerns	Some concerns	Overall Some concerns because D1, D5 raised Some concerns and no domain was High.
ROB2-APPRAISAL-037	TNSS_SAR	TF_E62	days 1-14 primary treatment-period result	Some concerns	Low	Low	Low	High	High	Overall High because D5 was High.
ROB2-APPRAISAL-038	TNSS_SAR	TF_E63	days 1-14 primary treatment-period result	Some concerns	Low	Low	Some concerns	High	High	Overall High because D5 was High.

ID	Structure	Trial unit	Window	D1	D2	D3	D4	D5	Overall	Brief justification
ROB2-APPRAISAL-039	TNSS_SAR	TF_NCT02320396	days 1-14 primary treatment-period result	Some concerns	Low	Low	Low	Some concerns	Some concerns	Overall Some concerns because D1, D5 raised Some concerns and no domain was High.
ROB2-APPRAISAL-040	TOSS_SAR	TF_E29_FFU109045	days 1-14 primary treatment-period result	Some concerns	Low	Low	Low	High	High	Overall High because D5 was High.
ROB2-APPRAISAL-041	TOSS_SAR	TF_E29_FFU109047	days 1-14 primary treatment-period result	Some concerns	Low	Low	Low	High	High	Overall High because D5 was High.
ROB2-APPRAISAL-042	TOSS_SAR	TF_E42	days 1-14 primary treatment-period result	Some concerns	Low	Low	Low	Some concerns	Some concerns	Overall Some concerns because D1, D5 raised Some concerns and no domain was High.
ROB2-APPRAISAL-043	WDAE_PAR	TF_E15	28-day randomized treatment period	Some concerns	Low	Low	Low	Some concerns	Some concerns	Overall Some concerns because D1, D5 raised Some concerns and no domain was High.
ROB2-APPRAISAL-044	WDAE_PAR	TF_E17	28-day randomized double-blind treatment period	Some concerns	Low	Low	Low	Some concerns	Some concerns	Overall Some concerns because D1, D5 raised Some concerns and no domain was High.
ROB2-APPRAISAL-045	WDAE_PAR	TF_E18	28-day randomized treatment period	Some concerns	Low	Low	Low	Some concerns	Some concerns	Overall Some concerns because D1, D5 raised Some concerns and no domain was High.
ROB2-APPRAISAL-046	WDAE_PAR	TF_E20	28-day randomized treatment period	Low	Low	Low	Low	Some concerns	Some concerns	Overall Some concerns because D5 raised Some concerns and no domain was High.
ROB2-APPRAISAL-047	WDAE_PAR	TF_E27	28-day randomized treatment period	Some concerns	Low	Low	Low	Some concerns	Some concerns	Overall Some concerns because D1, D5 raised Some concerns and no domain was High.
ROB2-APPRAISAL-048	WDAE_PAR	TF_E71	28-day randomized treatment period	Some concerns	Low	Low	Low	Some concerns	Some concerns	Overall Some concerns because D1, D5 raised Some concerns and no domain was High.
ROB2-APPRAISAL-049	WDAE_PAR	TF_E81	28-day randomized treatment period	Low	Low	Low	Low	Some concerns	Some concerns	Overall Some concerns because D5 raised Some concerns and no domain was High.
ROB2-APPRAISAL-050	WDAE_SAR	TF_E29_FFU109045	14-day randomized treatment period	Some concerns	Low	Low	Low	High	High	Overall High because D5 was High.
ROB2-APPRAISAL-051	WDAE_SAR	TF_E29_FFU109047	14-day randomized treatment period	Some concerns	Low	Low	Low	High	High	Overall High because D5 was High.
ROB2-APPRAISAL-052	WDAE_SAR	TF_E55_NCT01241214	14-day randomized treatment period	Some concerns	Low	Low	Low	High	High	Overall High because D5 was High.

ID	Structure	Trial unit	Window	D1	D2	D3	D4	D5	Overall	Brief justification
ROB2-APPRAISAL-053	WDAE_SAR	TF_E55_NCT01484119	14-day randomized treatment period	Some concerns	Low	Low	Low	High	High	Overall High because D5 was High.
ROB2-APPRAISAL-054	WDAE_SAR	TF_A00430_NCT00621959	14-day randomized treatment period	Some concerns	Low	Low	Low	Low	Some concerns	Overall Some concerns because D1 raised Some concerns and no domain was High.
ROB2-APPRAISAL-055	WDAE_SAR	TF_A00431_NCT00653224	14-day randomized treatment period	Some concerns	Low	Low	Low	Low	Some concerns	Overall Some concerns because D1 raised Some concerns and no domain was High.
ROB2-APPRAISAL-056	WDAE_SAR	TF_E31	14-day randomized treatment period	Low	Low	Low	Low	Some concerns	Some concerns	Overall Some concerns because D5 raised Some concerns and no domain was High.
ROB2-APPRAISAL-057	WDAE_SAR	TF_E33	14-day randomized treatment period	Some concerns	Low	Low	Low	High	High	Overall High because D5 was High.
ROB2-APPRAISAL-058	WDAE_SAR	TF_E38	14-day randomized treatment period	Some concerns	Low	Low	Low	High	High	Overall High because D5 was High.
ROB2-APPRAISAL-059	WDAE_SAR	TF_E42	14-day randomized treatment period	Some concerns	Low	Low	Low	Some concerns	Some concerns	Overall Some concerns because D1, D5 raised Some concerns and no domain was High.
ROB2-APPRAISAL-060	WDAE_SAR	TF_E50	14-day randomized treatment period	Some concerns	Low	Low	Some concerns	Some concerns	Some concerns	Overall Some concerns because D1, D4, D5 raised Some concerns and no domain was High.
ROB2-APPRAISAL-061	WDAE_SAR	TF_E51	14-day randomized treatment period	Some concerns	Low	Some concerns	Low	Some concerns	Some concerns	Overall Some concerns because D1, D3, D5 raised Some concerns and no domain was High.
ROB2-APPRAISAL-062	WDAE_SAR	TF_E52	14-day randomized treatment period	Some concerns	Low	Some concerns	Some concerns	High	High	Overall High because D5 was High.
ROB2-APPRAISAL-063	WDAE_SAR	TF_E79	14-day randomized treatment period	Some concerns	Low	Low	Low	Some concerns	Some concerns	Overall Some concerns because D1, D5 raised Some concerns and no domain was High.
ROB2-APPRAISAL-064	WDAE_SAR	TF_E84	14-day randomized treatment period	Some concerns	Low	Low	Low	Some concerns	Some concerns	Overall Some concerns because D1, D5 raised Some concerns and no domain was High.
ROB2-APPRAISAL-065	WDAE_SAR	TF_E35_FALL2000_MONT_LOR_PBO	14-day randomized treatment period	Some concerns	Low	Some concerns	Low	High	High	Overall High because D5 was High.
ROB2-APPRAISAL-066	WDAE_SAR	TF_NCT02320396	14-day randomized treatment period	Some concerns	Low	Low	Low	Some concerns	Some concerns	Overall Some concerns because D1, D5 raised Some concerns and no domain was High.
ROB2-APPRAISAL-067	WDAE_PAR	TF_E26	28-day randomized treatment period	Low	Low	Low	Low	Some concerns	Some concerns	Overall Some concerns because D5 raised Some concerns and no domain was High.

ID	Structure	Trial unit	Window	D1	D2	D3	D4	D5	Overall	Brief justification
ROB2-APPRAISAL-068	TNSS_PAR	TF_E15	day 28	Some concerns	Low	Low	Low	Some concerns	Some concerns	Overall Some concerns because D1, D5 raised Some concerns and no domain was High.
ROB2-APPRAISAL-069	TOSS_PAR	TF_E15	day 28	Some concerns	Low	Low	Low	Some concerns	Some concerns	Overall Some concerns because D1, D5 raised Some concerns and no domain was High.
ROB2-APPRAISAL-070	TNSS_PAR	TF_E17	week 4 / 28 days	Some concerns	Low	Low	Low	Some concerns	Some concerns	Overall Some concerns because D1, D5 raised Some concerns and no domain was High.
ROB2-APPRAISAL-071	TNSS_PAR	TF_E20	days 1-28 average	Low	Low	Low	Low	Some concerns	Some concerns	Overall Some concerns because D5 raised Some concerns and no domain was High.
ROB2-APPRAISAL-072	TNSS_PAR	TF_E22	week 4	Some concerns	Low	Some concerns	Low	Low	Some concerns	Overall Some concerns because D1, D3 raised Some concerns and no domain was High.
ROB2-APPRAISAL-073	TNSS_SAR	TF_E55_NCT01484119	2-week average	Some concerns	Low	Low	Low	High	High	Overall High because D5 was High.

S31. Binary Active-Active Secondary Contrasts

Table S31. Binary active-active secondary contrasts.

Structure	Estimable active nodes	Active-active pairs	95% CrI includes 1	95% CrI excludes 1	Directly informed	Indirect-only
Any AE-SAR	9	36	32	4	5	31
Any AE-PAR	10	45	45	0	6	39
WDAE-SAR	7	21	19	2	3	18
WDAE-PAR	10	45	45	0	9	36
Total	-	147	141	6	23	124

ORs were derived as exact within-draw differences, d[A] – d[B], using 24,000 retained posterior draws per structure from the implementation-independent CmdStanR replication of the corresponding source-primary scenarios. OR < 1 indicates lower odds of the adverse outcome with the first-listed regimen. No model was refitted and no additional MCMC sampling was performed. Before derivation, all 36 estimable placebo-referenced effects reproduced the source-primary summaries within prespecified numerical tolerances. The complete 147-row results and evidence-route classifications are provided in Supplementary Data S1 (CSV file: Supplementary_Data_S1_Binary_Active_Active_Effects_v1.1.csv). These secondary contrasts were not multiplicity-adjusted or formally appraised with CINeMA and were not used to support ranking, superiority, equivalence, or preferential-tolerability claims.

S32. Binary Effect Cross-Table Consistency Audit

Table S32. Automated source-primary consistency check linking Tables S16–S20 and S27 with Figures 4 and 5.

Network	Treatment	OR median	95% CrI	Null class	S20 status	QA
ANY_AE_SAR	bilastine_20mg_OD	0.885	0.543-1.362	Includes 1	Not applicable	PASS
ANY_AE_SAR	cetirizine_10mg_OD	1.151	0.600-1.815	Includes 1	Not applicable	PASS
ANY_AE_SAR	desloratadine_5mg_OD	1.238	0.660-2.214	Includes 1	Not applicable	PASS
ANY_AE_SAR	fexofenadine_120mg_OD	1.040	0.663-1.664	Includes 1	Not applicable	PASS
ANY_AE_SAR	fexofenadine_180mg_OD	1.038	0.577-1.872	Includes 1	Not applicable	PASS
ANY_AE_SAR	levocetirizine_5mg_OD	0.864	0.529-1.375	Includes 1	Not applicable	PASS
ANY_AE_SAR	loratadine_10mg_OD	1.085	0.683-1.726	Includes 1	Not applicable	PASS
ANY_AE_SAR	rupatadine_10mg_OD	2.126	0.977-4.308	Includes 1	Boundary-indeterminate: primary CrI includes 1; implementation-independent replication excludes 1	PASS
ANY_AE_SAR	rupatadine_20mg_OD	2.291	1.076-4.623	Excludes 1	Not boundary-indeterminate under primary/replication; wider-prior sensitivity includes 1	PASS
ANY_AE_PAR	bepotastine_10mg_BID	1.590	0.703-3.510	Includes 1	Not applicable	PASS
ANY_AE_PAR	bepotastine_CR_20mg_OD	1.655	0.708-3.866	Includes 1	Not applicable	PASS
ANY_AE_PAR	bilastine_20mg_OD	1.025	0.548-1.956	Includes 1	Not applicable	PASS
ANY_AE_PAR	cetirizine_10mg_OD	0.795	0.488-1.365	Includes 1	Not applicable	PASS
ANY_AE_PAR	desloratadine_5mg_OD	0.759	0.395-1.504	Includes 1	Not applicable	PASS
ANY_AE_PAR	ebastine_10mg_OD	0.683	0.274-1.857	Includes 1	Not applicable	PASS
ANY_AE_PAR	ebastine_20mg_OD	0.540	0.216-1.503	Includes 1	Not applicable	PASS
ANY_AE_PAR	loratadine_10mg_OD	0.711	0.378-1.397	Includes 1	Not applicable	PASS
ANY_AE_PAR	mizolastine_10mg_OD	0.861	0.509-1.470	Includes 1	Not applicable	PASS
ANY_AE_PAR	rupatadine_10mg_OD	1.404	0.630-3.138	Includes 1	Not applicable	PASS
WDAE_SAR	bilastine_20mg_OD	0.341	0.076-1.272	Includes 1	Not applicable	PASS
WDAE_SAR	cetirizine_10mg_OD	0.247	0.081-0.657	Excludes 1	Not applicable	PASS
WDAE_SAR	desloratadine_5mg_OD	0.740	0.256-2.117	Includes 1	Not applicable	PASS
WDAE_SAR	fexofenadine_120mg_OD	0.525	0.131-1.908	Includes 1	Not applicable	PASS
WDAE_SAR	fexofenadine_180mg_OD	1.694	0.496-6.025	Includes 1	Not applicable	PASS
WDAE_SAR	levocetirizine_5mg_OD	0.459	0.114-1.569	Includes 1	Not applicable	PASS
WDAE_SAR	loratadine_10mg_OD	1.069	0.471-2.410	Includes 1	Not applicable	PASS
WDAE_SAR	rupatadine_10mg_OD	Not estimable	-	Not estimable	Not applicable	PASS
WDAE_SAR	rupatadine_20mg_OD	Not estimable	-	Not estimable	Not applicable	PASS
WDAE_PAR	bepotastine_10mg_BID	0.664	0.082-4.507	Includes 1	Not applicable	PASS
WDAE_PAR	bilastine_20mg_OD	1.197	0.209-6.145	Includes 1	Not applicable	PASS
WDAE_PAR	cetirizine_10mg_OD	0.774	0.212-2.757	Includes 1	Not applicable	PASS
WDAE_PAR	desloratadine_5mg_OD	1.456	0.463-4.570	Includes 1	Not applicable	PASS
WDAE_PAR	ebastine_10mg_OD	1.506	0.213-10.447	Includes 1	Not applicable	PASS

Network	Treatment	OR median	95% CrI	Null class	S20 status	QA
WDAE_PAR	ebastine_20mg_OD	0.347	0.031-2.991	Includes 1	Not applicable	PASS
WDAE_PAR	loratadine_10mg_OD	1.688	0.554-5.060	Includes 1	Not applicable	PASS
WDAE_PAR	mizolastine_10mg_OD	0.733	0.231-2.219	Includes 1	Not applicable	PASS
WDAE_PAR	rupatadine_10mg_OD	0.434	0.066-2.185	Includes 1	Not applicable	PASS
WDAE_PAR	rupatadine_20mg_OD	0.836	0.106-5.362	Includes 1	Not applicable	PASS

Result: 38/38 rows passed; 36 estimable OR/CrI rows match the source-primary file and two WDAE-SAR rows remain non-estimable.

S33. Continuous Heterogeneity and Generalized Cochran Q Diagnostics

Table S33. Continuous-network heterogeneity and generalized Cochran Q diagnostics.

Structure	k	p	Residual df	Generalized Q	Q p value	REML tau	95% profile CI	I^2 (%)	Interpretation
RQLQ-PER	2	2	0	0.0	Not testable (df=0)	0.0	Not estimable to Not estimable	Not estimable	Saturated; heterogeneity not identifiable
RQLQ-SAR	7	4	3	3.062019355	0.3821615058	0.0	0.0 to 0.2896345263	2.0254396801	Boundary estimate; not informatively estimated
TNSS-PAR	4	3	1	1.1188438956	0.2901675293	0.108314488	0.0 to 3.7120511459	10.6220265473	Identifiable but weakly estimated
TNSS-SAR	14	7	7	11.9260205116	0.103018122	0.1626354558	0.0 to 0.4823481945	41.3048133435	Identifiable; comparison-level CINeMA judgments vary
TOSS-SAR	4	3	1	1.6393442623	0.2004154617	0.1324764203	0.0 to 2.3720876178	39.0	Identifiable but weakly estimated

Q is the generalized fixed-effect Cochran statistic calculated with the within-multiarm sampling covariance matrix; residual df equals contrast rank minus the number of basic treatment parameters. I^2 is descriptive. REML tau profile intervals are shown where heterogeneity was identifiable. RQLQ-PER was saturated (df=0), so Q was not testable and heterogeneity was not identifiable. These values reproduce the frozen continuous source implementation; no model was refitted for v1.1.

S34. Submission Version and Repository Status

Table S34. Submission version and repository status.

Element	Submitted version 1.0	Current version 1.1
Primary analytical source	Frozen analytical release v56.0	Same primary fitted models and placebo-referenced estimates
Binary active-active reporting	Pair scope only	All 147 estimable secondary contrasts reported from retained implementation-independent posterior draws
Model execution	Primary models fitted in v56.0	No model refit and no additional MCMC sampling
Human validation	Implementation-independent replication by L.R.R.	L.R.R. completed and approved validation: 147/147 PASS; max difference $5.0000049 \times 10^{-10}$.
PRISMA materials	Initial submitted set	Complete PRISMA 2020, PRISMA Abstracts, PRISMA-NMA, and PRISMA-S set
Public repository	Zenodo v1.0.0 (doi:10.5281/zenodo.21780525), corresponding to submission version 1.0	Zenodo v1.1 (doi:10.5281/zenodo.22086455), corresponding to the current submission
Editorial status	Replaced by version 1.1	Current complete submission

File-set instruction: replace all documents and supplementary files from submitted version 1.0 with the corresponding version 1.1 files. Version 1.1 is the complete current submission. The frozen primary analytical release remains v56.0; the 147 binary active-active estimates are secondary algebraic derivations from retained posterior draws and do not alter the primary models, existing appraisals, figures, or conclusions. Zenodo v1.1 (doi:10.5281/zenodo.22086455) is the current public reproducibility release corresponding to submission version 1.1; Zenodo v1.0.0 (doi:10.5281/zenodo.21780525) remains the archived public release corresponding to submission version 1.0.

Supplementary abbreviations

Abbreviations used in the supplement.

Abbreviation	Definition
AE	Adverse event
BID	Twice daily
CI	Confidence interval
CINeMA	Confidence in Network Meta-Analysis
CrI	Credible interval
df	Degrees of freedom
GLS	Generalized least squares
GRADE-NMA	GRADE approach for network meta-analysis
H1	Histamine H1 receptor
I2	Percentage of total variation attributable to heterogeneity
MD	Mean difference
NMA	Network meta-analysis
OD	Once daily
OR	Odds ratio
OSF	Open Science Framework
PAR	Perennial allergic rhinitis
PER	Persistent allergic rhinitis
PrI	Prediction interval
PRISMA	Preferred Reporting Items for Systematic Reviews and Meta-Analyses
REML	Restricted maximum likelihood
ROB-MEN	Risk Of Bias due to Missing Evidence in Network meta-analysis
RoB 2	Revised Cochrane risk-of-bias tool for randomized trials
RQLQ	Rhinoconjunctivitis Quality of Life Questionnaire
SAR	Seasonal allergic rhinitis
CR	Controlled-release formulation
TNSS	Total nasal symptom score
TOSS	Total ocular symptom score
WDAE	Withdrawal due to adverse events

Supplementary Figures S1–S32

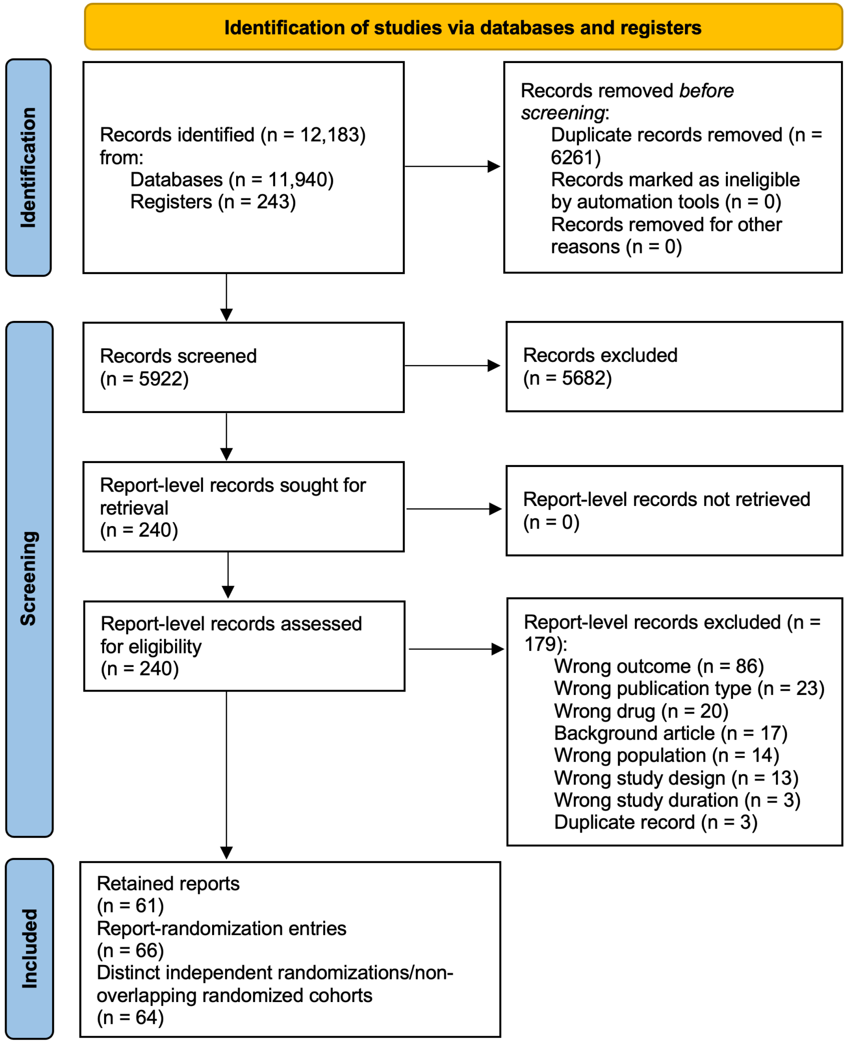

Figure S1. PRISMA 2020 flow diagram. The 240 report-level records assessed for eligibility represented 237 unique reports because three were duplicate bibliographic representations of reports already retained. Thus, the 179 report-level exclusion decisions comprised 176 substantive exclusions and three duplicate-report records, while 61 unique reports were retained (237 = 61 + 176; 240 = 237 + 3). The 61 retained reports generated 66 report–randomization entries; two were non-incremental companion links to randomizations already represented by primary reports, yielding 64 distinct independent randomizations/non-overlapping randomized cohorts comprising 34,831 randomized participants (66 − 2 = 64). Of these 64 cohorts, 35 contributed model-ready data to at least one principal evidence structure, 28 were retained only in the evidence map, and one was contextual/sensitivity-only (64 = 35 + 28 + 1). All 240 report-level records sought for retrieval were retrieved in an evaluable form (not retrieved, $n = 0$); 41 unique reports were available only as abstracts because no more complete report could be identified, and all were excluded after eligibility assessment. No records were identified through other methods ($n = 0$).

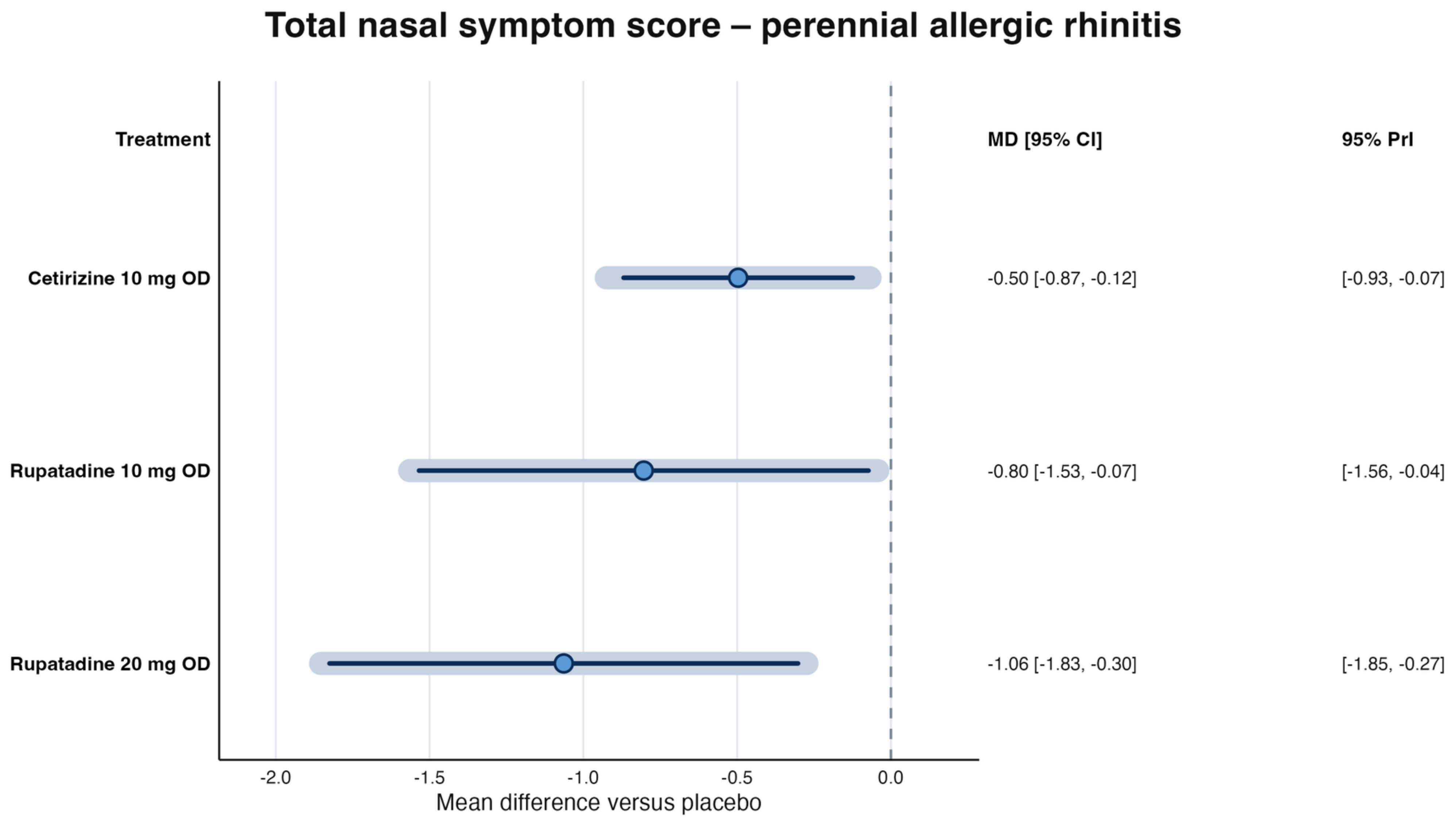

Negative values favour the active treatment. Pale thick lines are 95% prediction intervals when estimable; non-estimable prediction intervals are stated explicitly.

Figure S2. Mean differences versus placebo for TNSS in perennial allergic rhinitis (TNSS–PAR). Negative values favor active treatment. Dark lines show 95% confidence intervals; pale lines show plug-in 95% prediction intervals conditional on the REML estimate of τ.

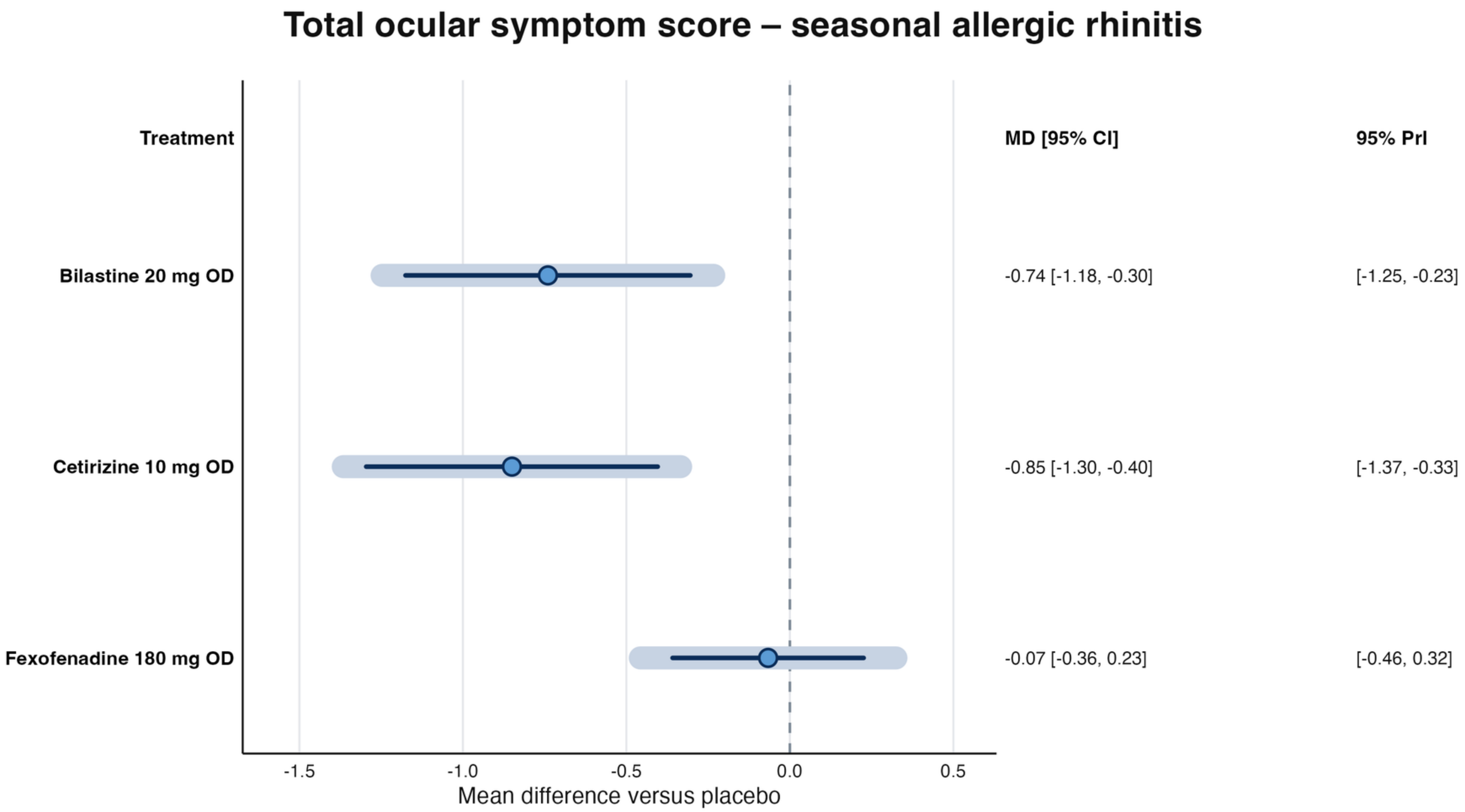

Negative values favour the active treatment. Pale thick lines are 95% prediction intervals when estimable; non-estimable prediction intervals are stated explicitly.

Figure S3. Mean differences versus placebo for TOSS in seasonal allergic rhinitis (TOSS–SAR). Negative values favor active treatment. Dark lines show 95% confidence intervals; pale lines show plug-in 95% prediction intervals conditional on the REML estimate of τ.

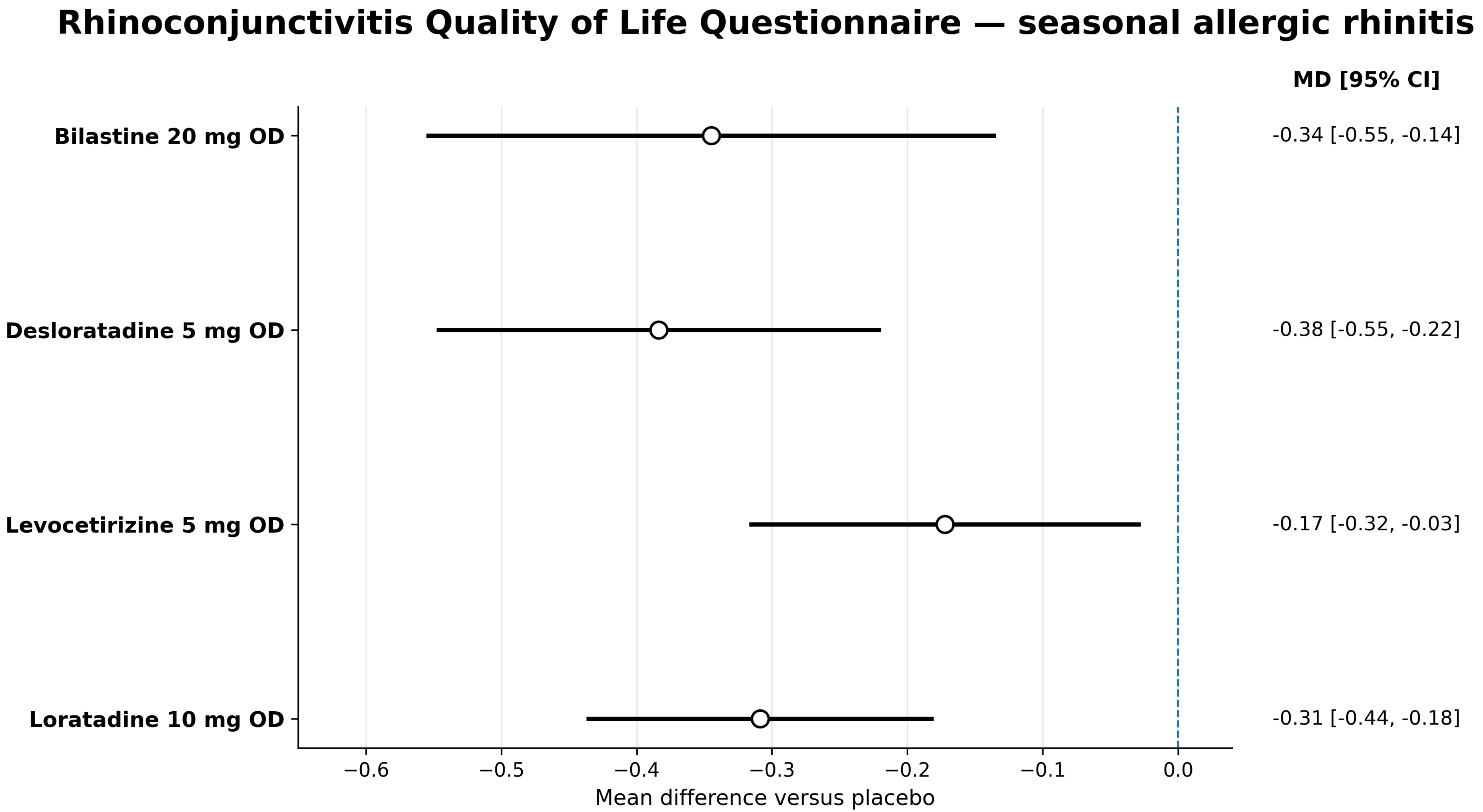

Negative values favor active treatment. No plug-in prediction interval is shown because the REML estimate of τ was at the boundary of zero and was not considered informatively estimated.

Figure S4. Mean differences versus placebo for RQLQ in seasonal allergic rhinitis (RQLQ–SAR). Negative values favor active treatment. Points and lines represent mean differences and 95% confidence intervals. The REML estimate of τ was at the boundary of zero and was not considered informatively estimated; no plug-in prediction interval is shown.

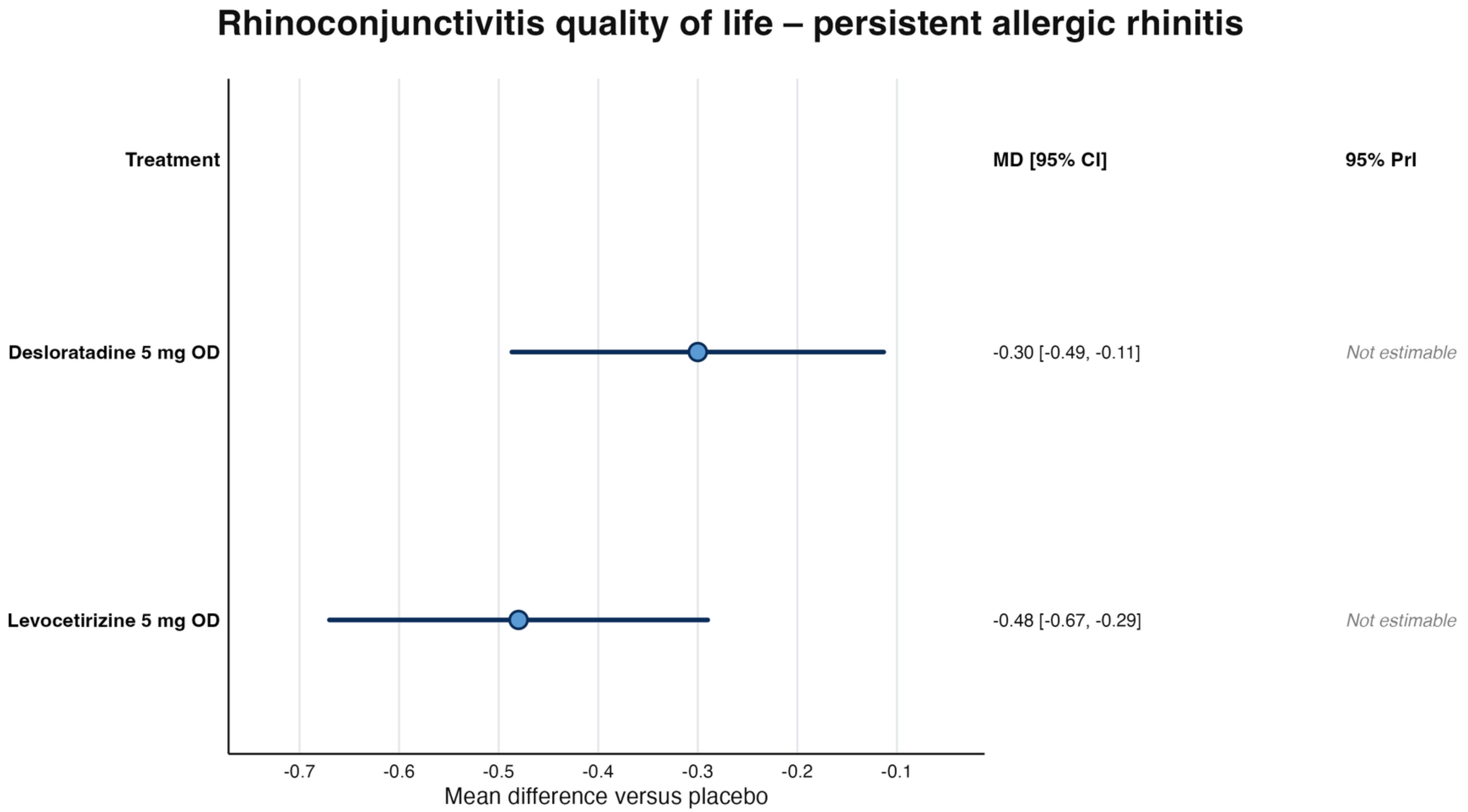

Negative values favour the active treatment. Pale thick lines are 95% prediction intervals when estimable; non-estimable prediction intervals are stated explicitly.

Figure S5. Mean differences versus placebo for RQLQ in persistent allergic rhinitis (RQLQ–PER). Negative values favor active treatment. The consistency model was saturated; heterogeneity, prediction intervals, and incoherence were not estimable.

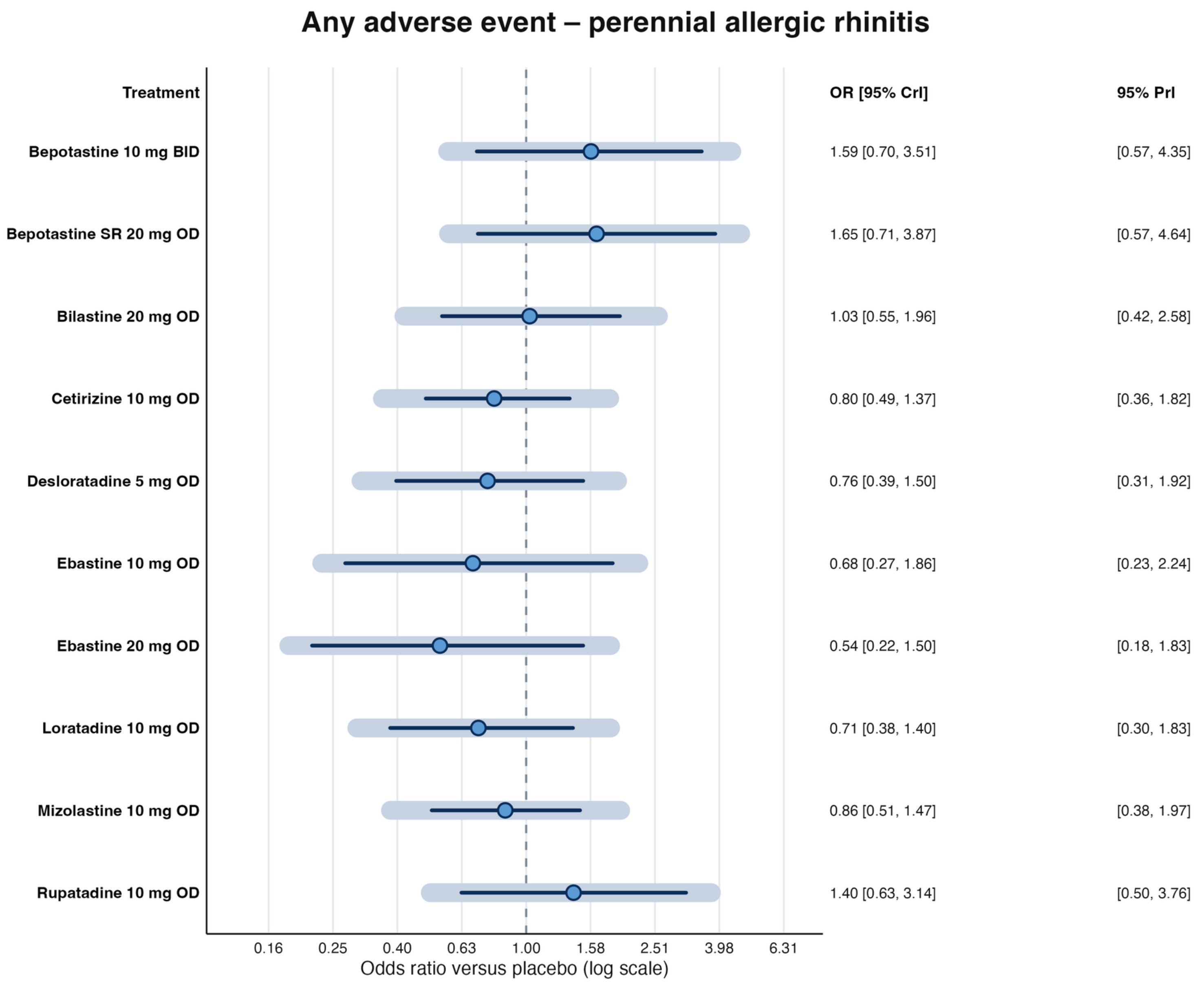

Values <1 favour active treatment. Pale thick lines are 95% prediction intervals when estimable; non-estimable prediction intervals are stated explicitly.

Figure S6. Posterior odds ratios versus placebo for any adverse event in perennial allergic rhinitis (Any AE–PAR) under the primary half-normal heterogeneity prior with scale 0.5. Values below 1 favor active treatment. Dark and pale lines show 95% credible and prediction intervals, respectively.

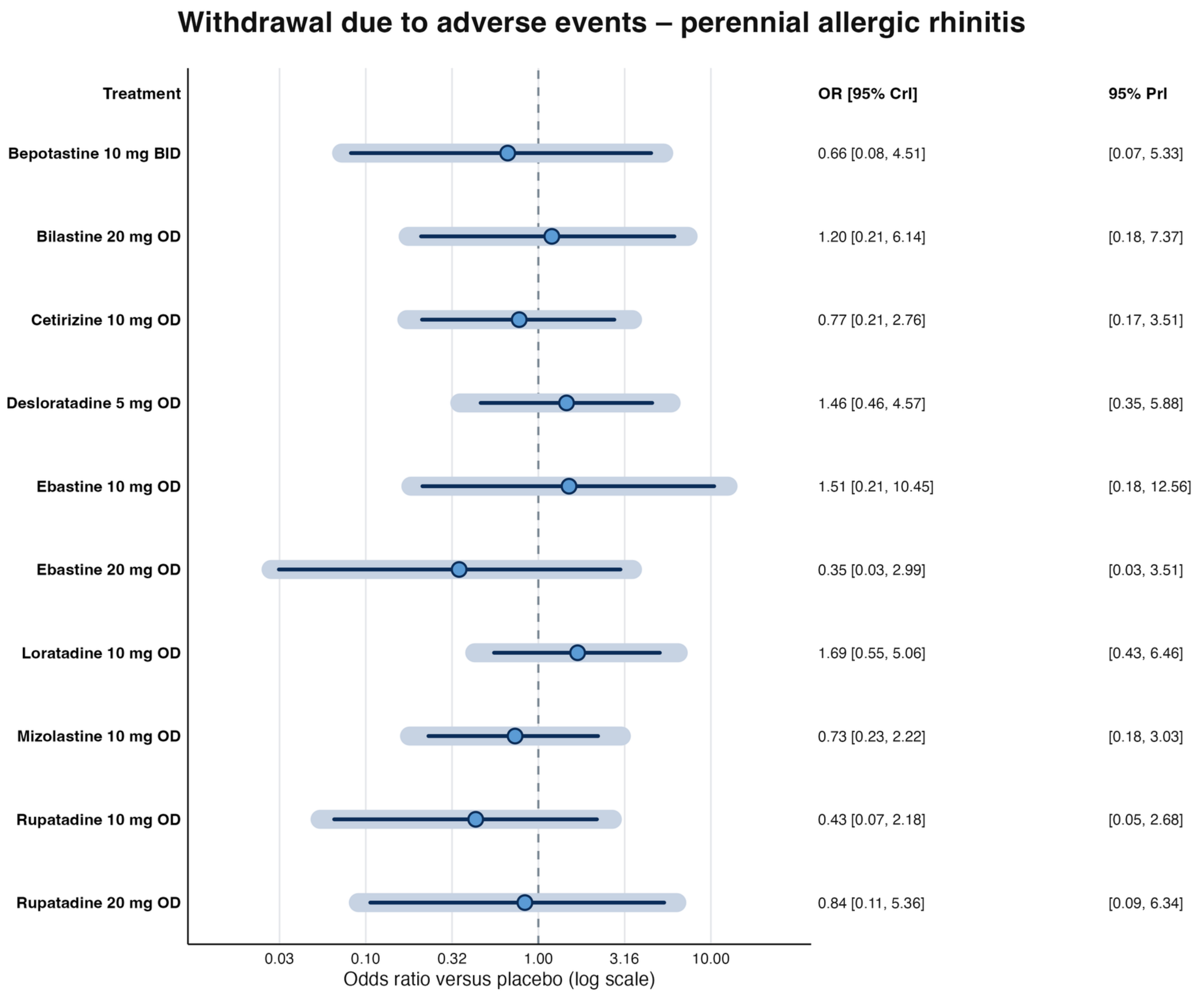

Values <1 favour active treatment. Pale thick lines are 95% prediction intervals when estimable; non-estimable prediction intervals are stated explicitly.

Figure S7. Posterior odds ratios versus placebo for withdrawal due to adverse events in perennial allergic rhinitis (WDAE–PAR) under the primary half-normal heterogeneity prior with scale 0.5. Values below 1 favor active treatment. Dark and pale lines show 95% credible and prediction intervals, respectively.

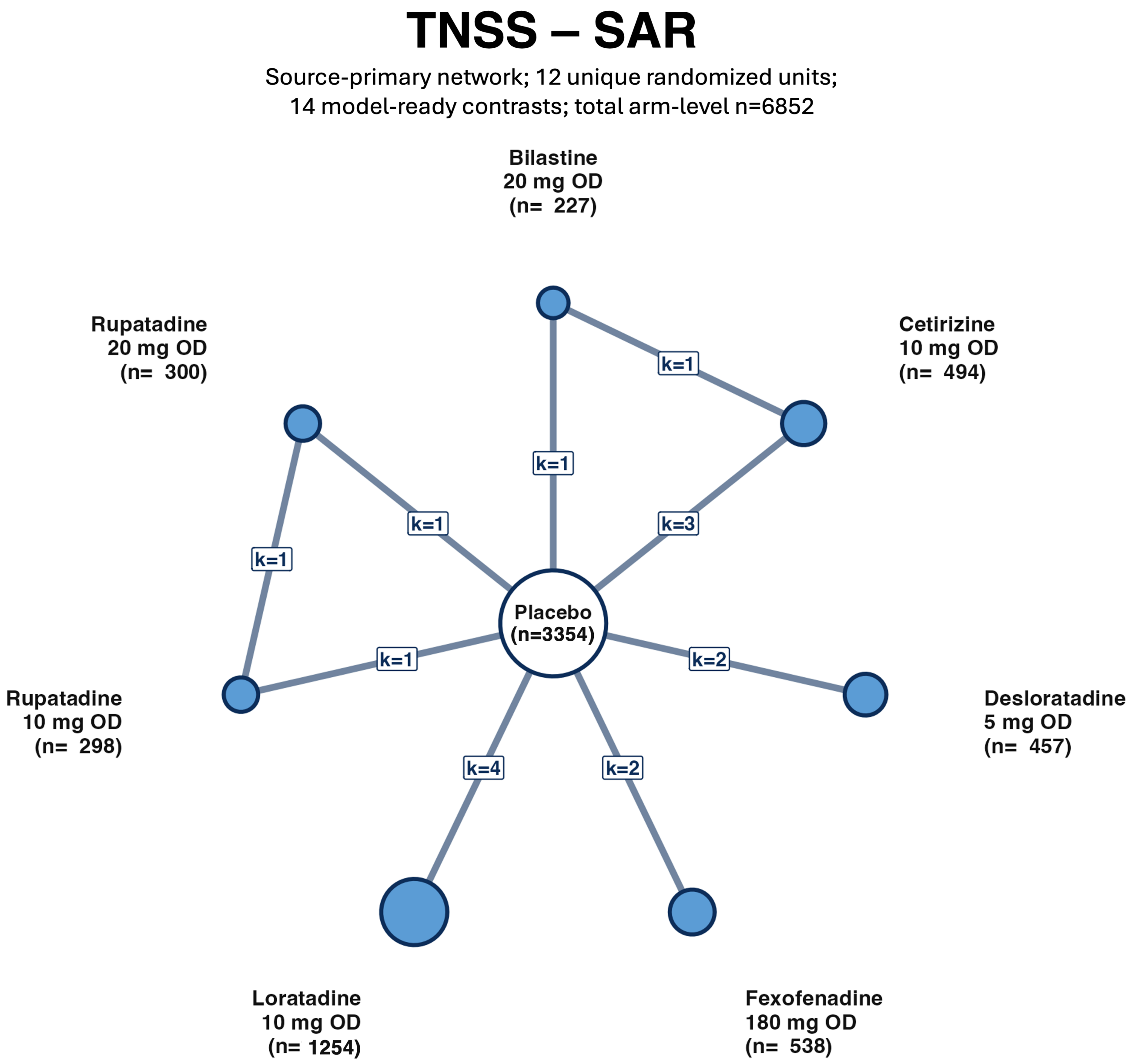

Figure S8. Direct-comparison geometry for TNSS–SAR. Node area is proportional to the accumulated arm-level denominator; edges denote direct randomized comparisons, and edge labels give the number of contributing randomizations. Geometry represents evidence quantity and structure, not effect magnitude, ranking, or confidence.

TNSS – PAR

Source-primary network; 2 unique randomized units;
4 model-ready contrasts; total arm-level n=1004

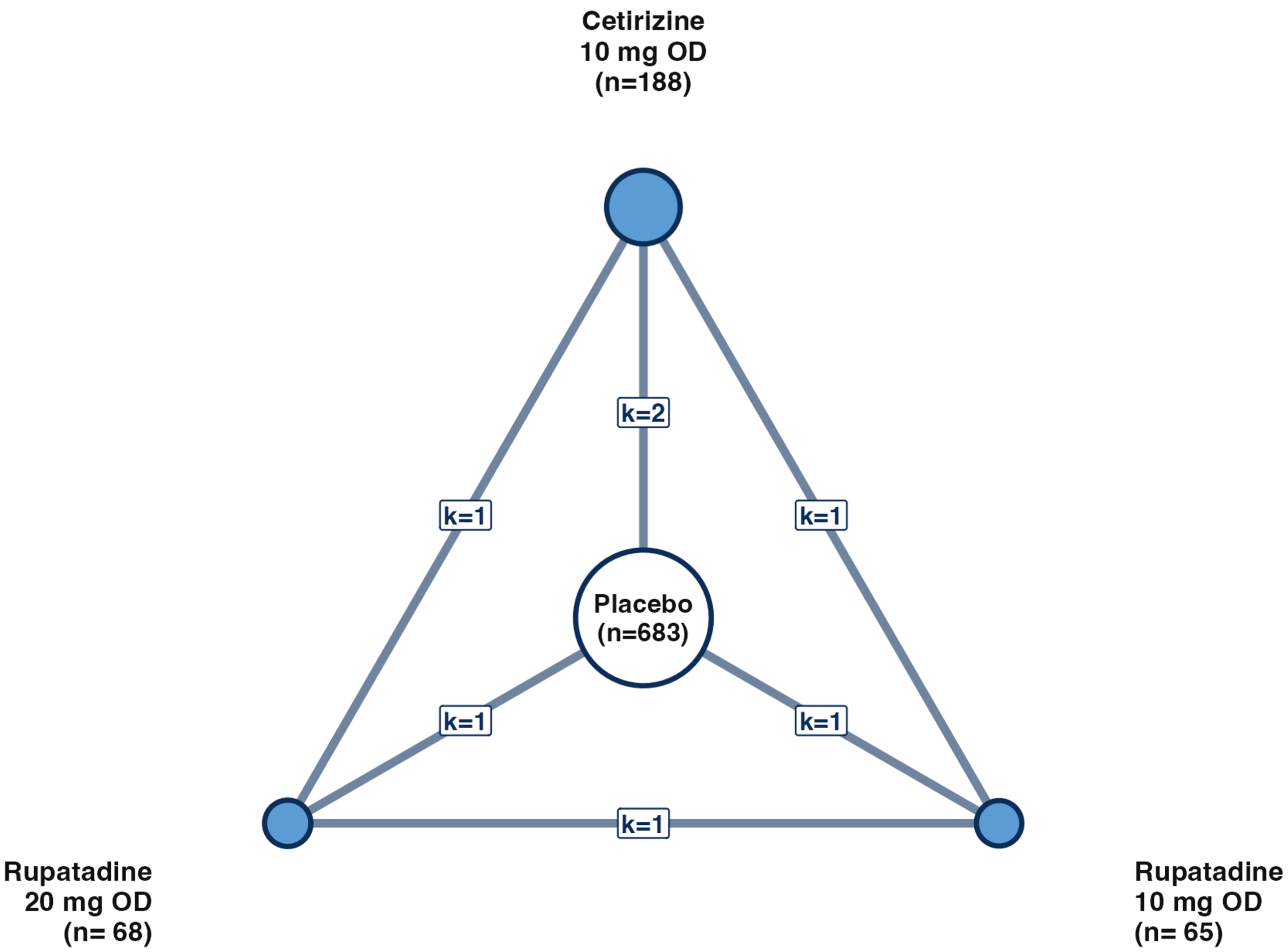

Figure S9. Direct-comparison geometry for TNSS–PAR. Node area is proportional to the accumulated arm-level denominator; edges denote direct randomized comparisons, and edge labels give the number of contributing randomizations. Geometry represents evidence quantity and structure, not effect magnitude, ranking, or confidence.

TOSS – SAR

Source-primary network; 3 unique randomized units;
4 model-ready contrasts; total arm-level n=1761

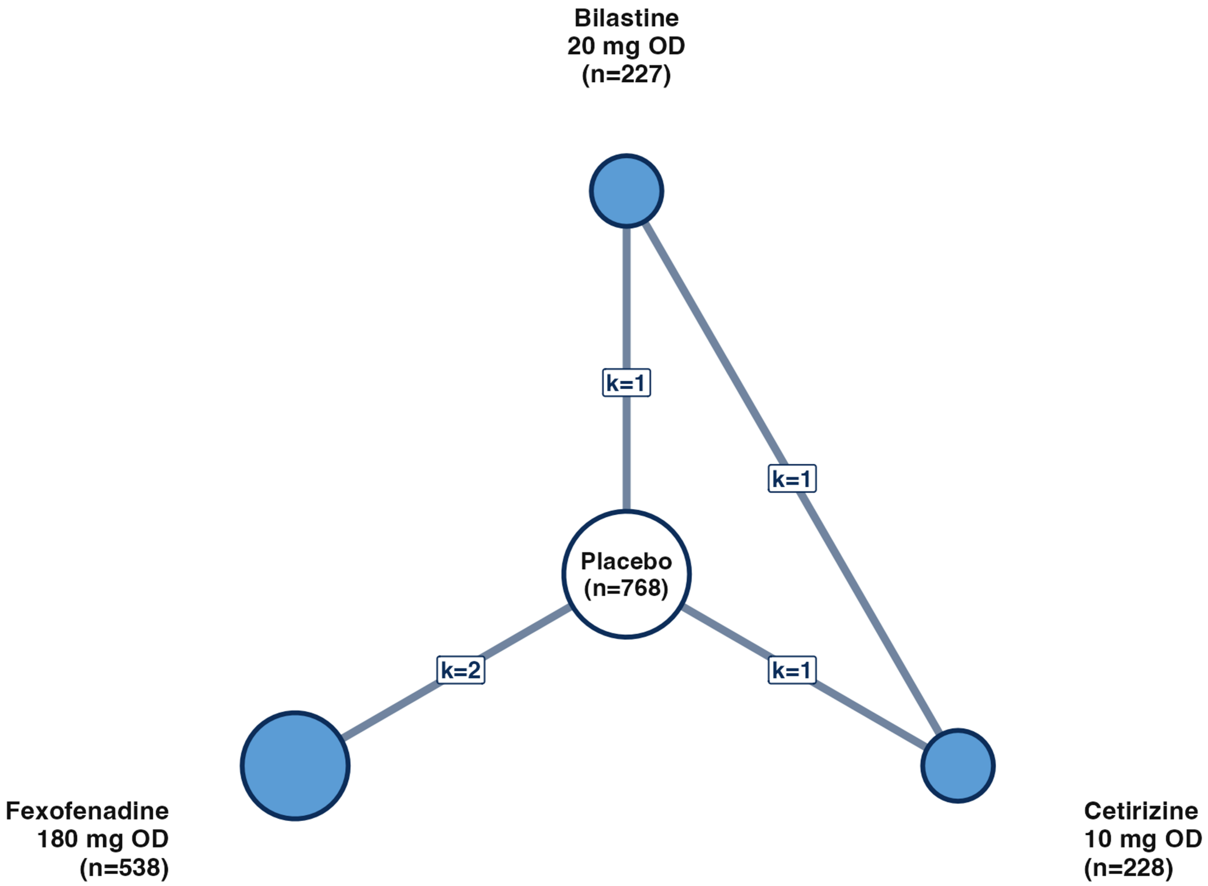

Figure S10. Direct-comparison geometry for TOSS–SAR. Node area is proportional to the accumulated arm-level denominator; edges denote direct randomized comparisons, and edge labels give the number of contributing randomizations. Geometry represents evidence quantity and structure, not effect magnitude, ranking, or confidence.

RQLQ – SAR

Source-primary network; 6 unique randomized units;
7 model-ready contrasts; total arm-level n=3700

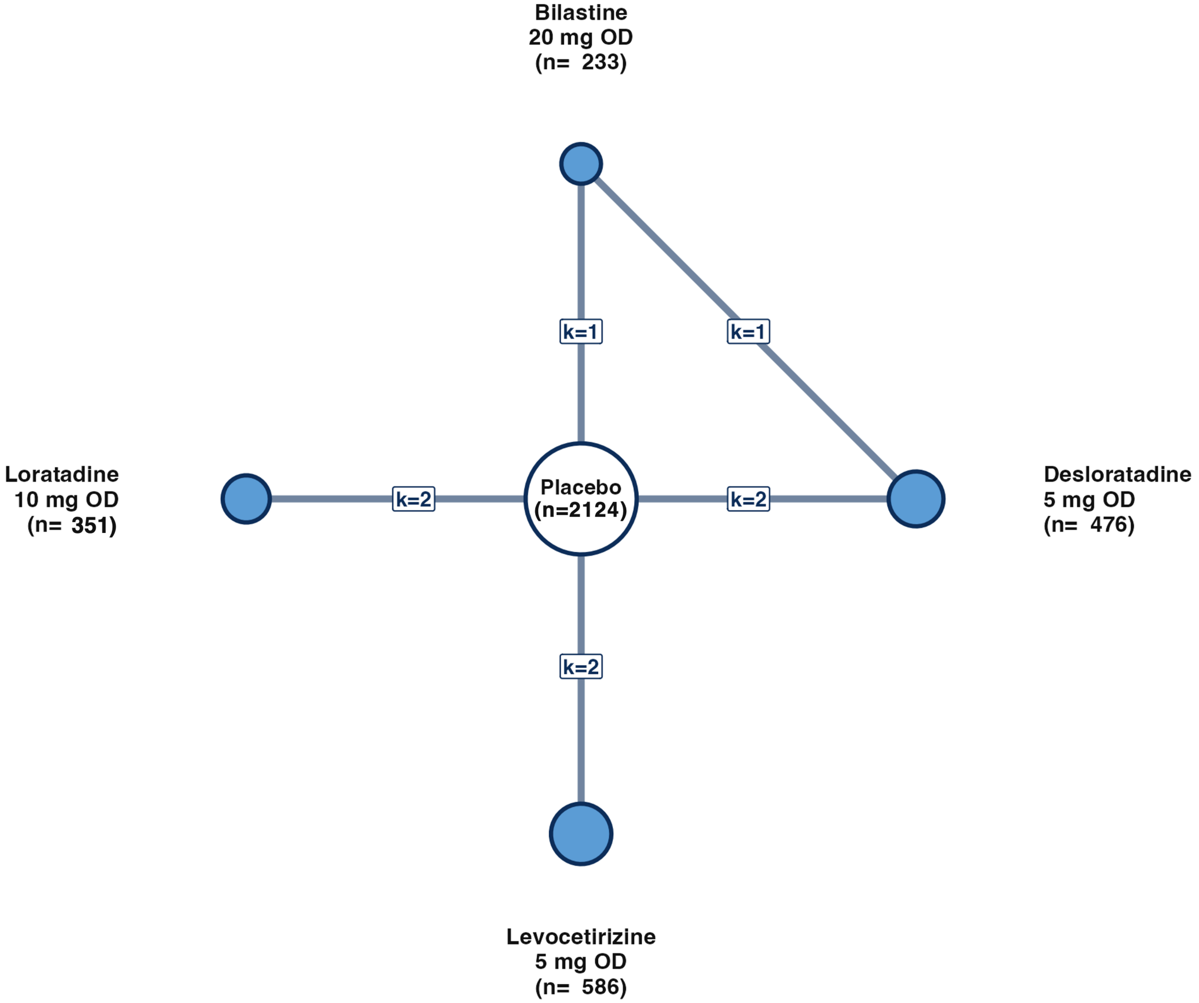

Figure S11. Direct-comparison geometry for RQLQ–SAR. Node area is proportional to the accumulated arm-level denominator; edges denote direct randomized comparisons, and edge labels give the number of contributing randomizations. Geometry represents evidence quantity and structure, not effect magnitude, ranking, or confidence.

RQLQ – PER

Source-primary network; 2 unique randomized units;
2 model-ready contrasts; total arm-level n=1093

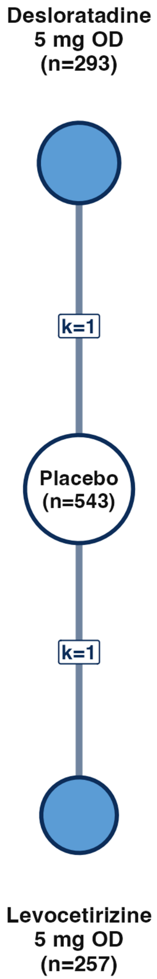

Figure S12. Direct-comparison geometry for RQLQ–PER. Node area is proportional to the accumulated arm-level denominator; edges denote direct randomized comparisons, and edge labels give the number of contributing randomizations. Geometry represents evidence quantity and structure, not effect magnitude, ranking, or confidence.

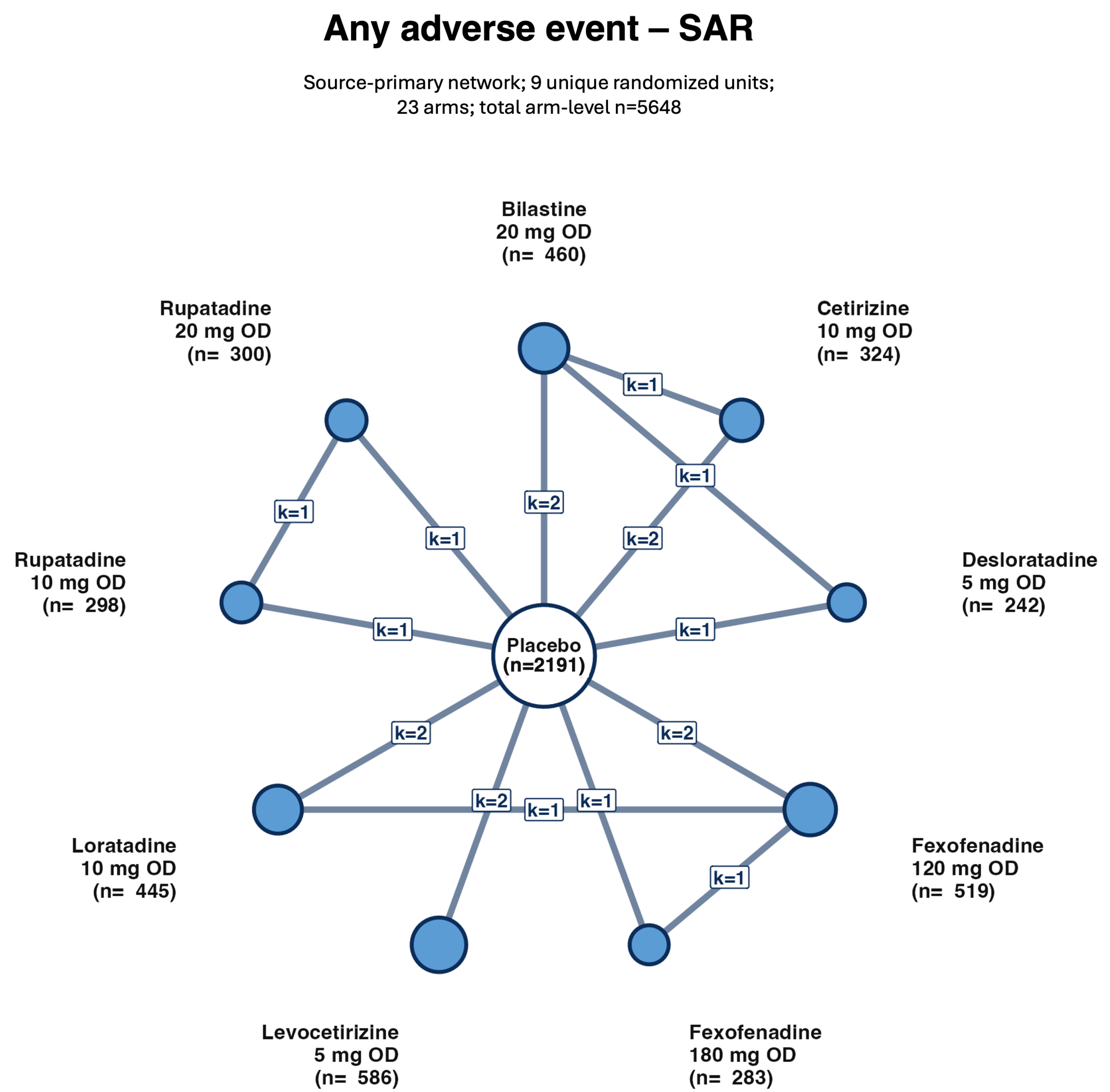

Figure S13. Direct-comparison geometry for Any AE–SAR. Node area is proportional to the accumulated arm-level denominator; edges denote direct randomized comparisons, and edge labels give the number of contributing randomizations. Geometry represents evidence quantity and structure, not effect magnitude, ranking, or confidence.

Any adverse event – PAR

Source-primary network; 8 unique randomized units;
20 arms; total arm-level n=3239

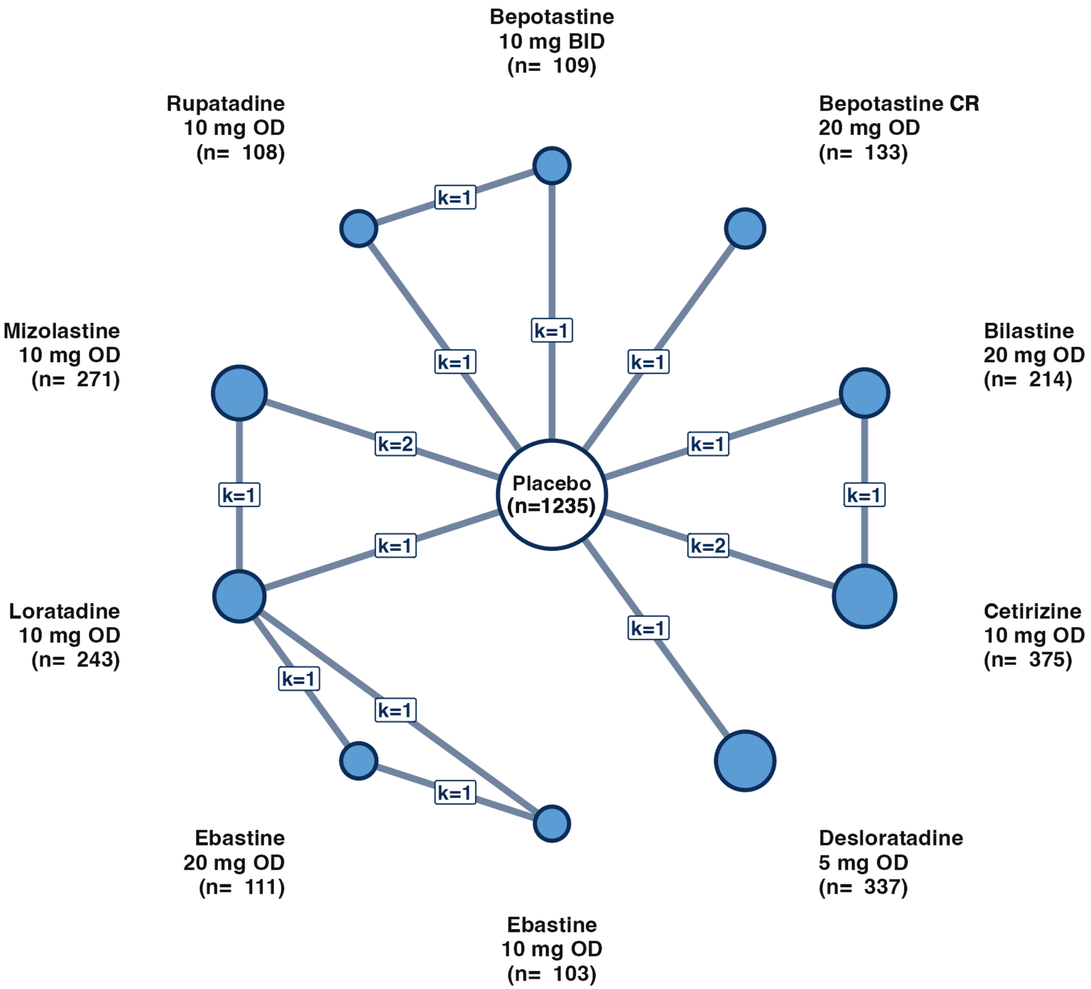

Figure S14. Direct-comparison geometry for Any AE–PAR. Node area is proportional to the accumulated arm-level denominator; edges denote direct randomized comparisons, and edge labels give the number of contributing randomizations. Geometry represents evidence quantity and structure, not effect magnitude, ranking, or confidence.

Withdrawal due to adverse events – SAR

Source-primary network; 17 unique randomized units;
38 arms; total arm-level n=9149

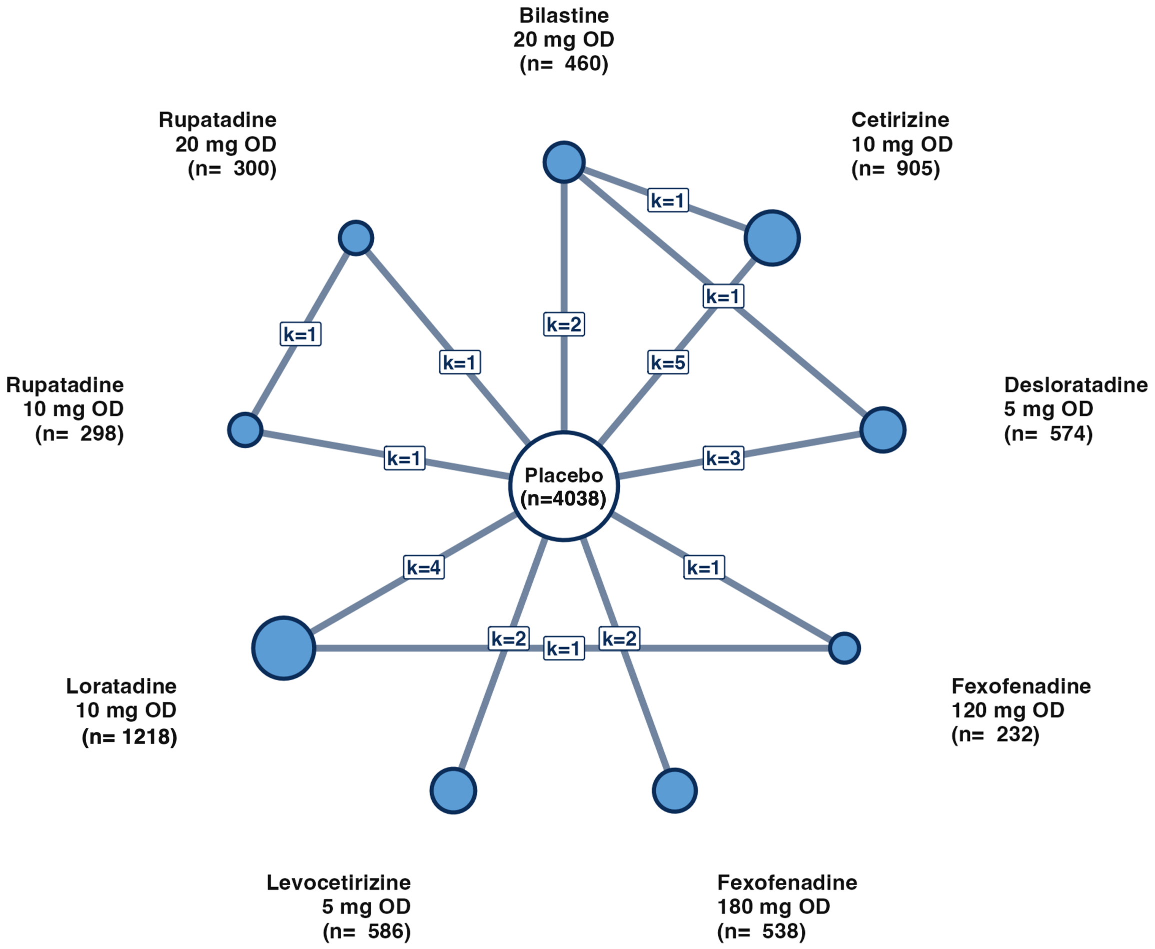

Figure S15. Direct-comparison geometry for WDAE–SAR. The structural geometry includes one three-arm randomization with zero withdrawals in every arm. That unit contributed no relative-effect information; rupatadine 10 mg and 20 mg were therefore structurally present but non-estimable.

Withdrawal due to adverse events – PAR

Source-primary network; 8 unique randomized units;
22 arms; total arm-level n=3251

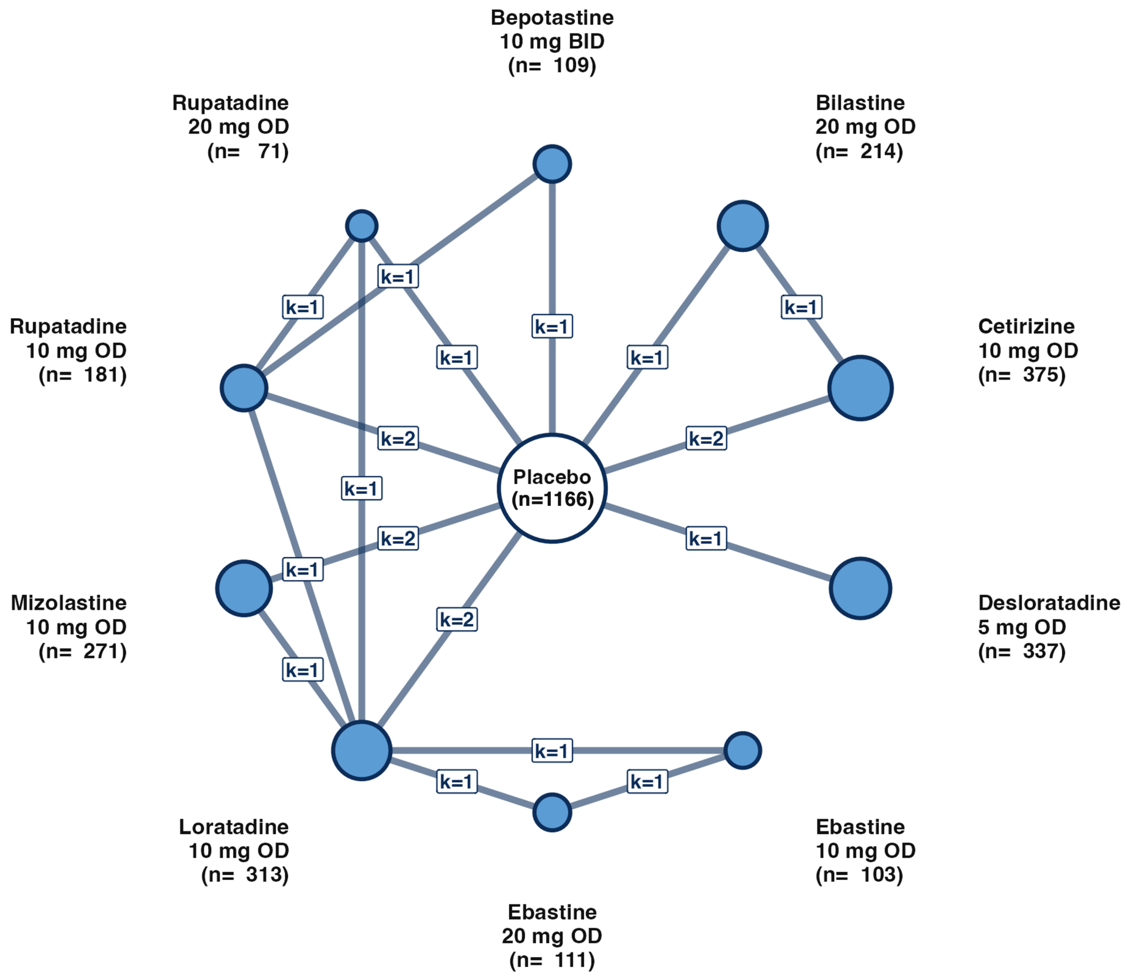

Figure S16. Direct-comparison geometry for WDAE–PAR. Node area is proportional to the accumulated arm-level denominator; edges denote direct randomized comparisons, and edge labels give the number of contributing randomizations. Geometry represents evidence quantity and structure, not effect magnitude, ranking, or confidence.

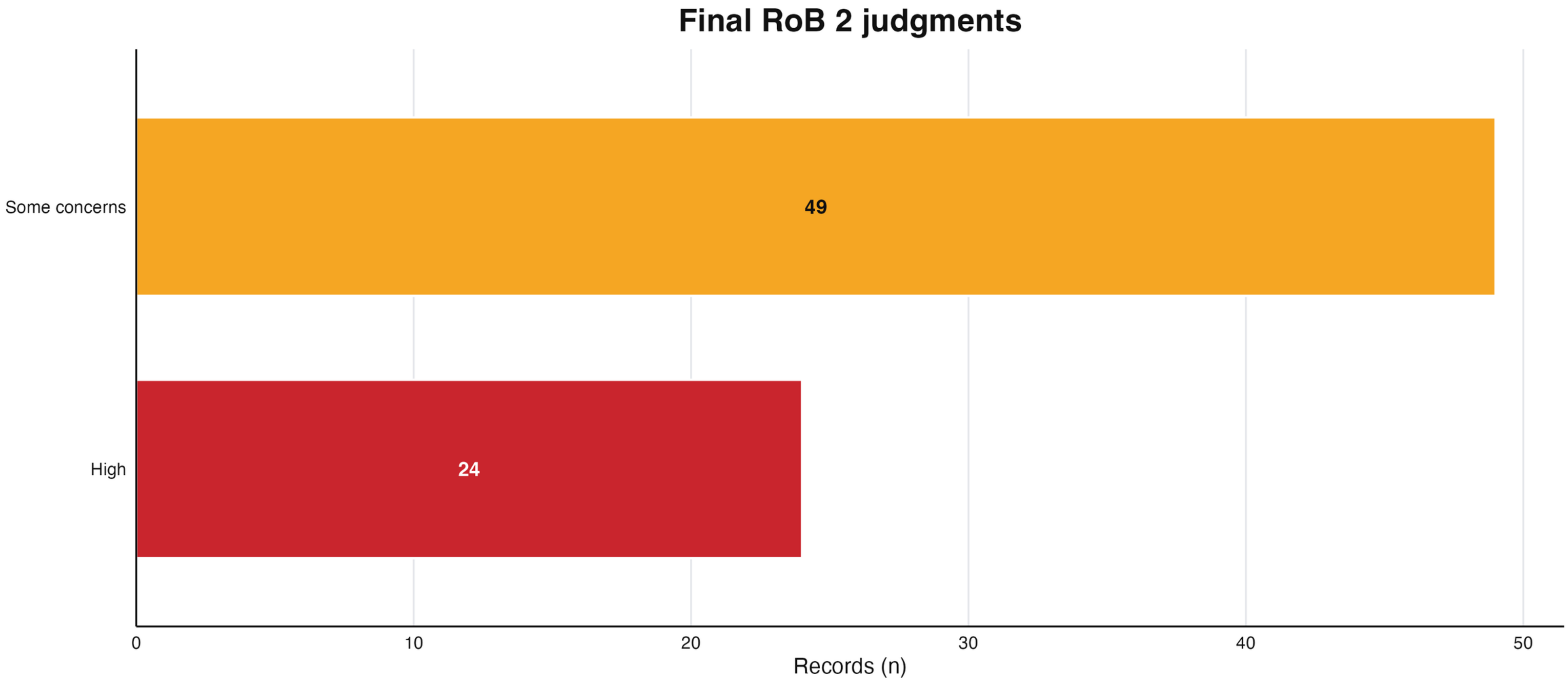

Seventy-three result-specific assessments confirmed in the final version closed panel.

Figure S17. Final RoB 2 overall judgments across 73 result-specific assessments: 49 some concerns and 24 high risk; none low risk.

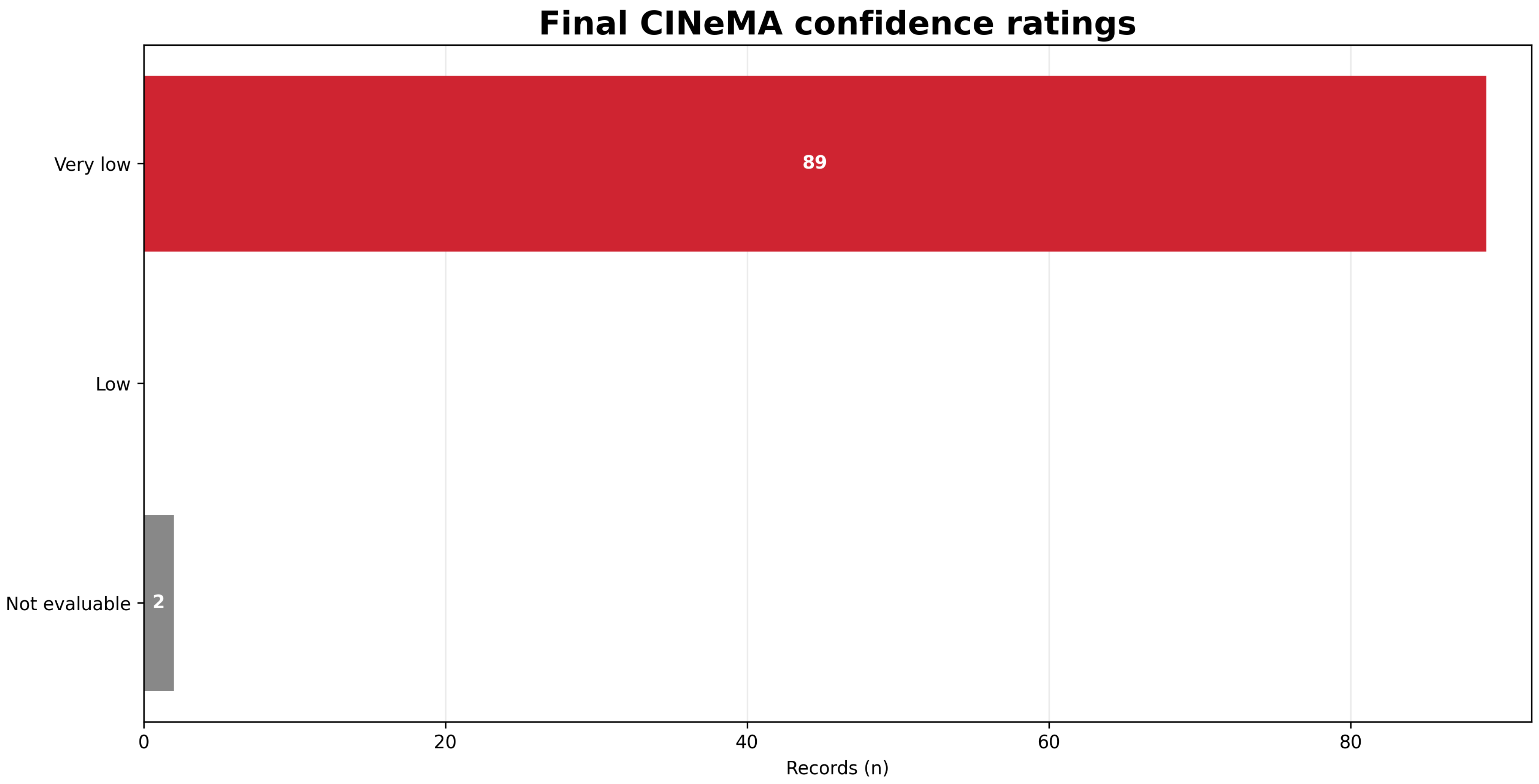

Ninety-one comparisons: 89 estimable effects and two non-estimable effects.

Figure S18. Final CINeMA confidence distribution across 91 assessments: 89 very low confidence, no low-confidence assessments, and two not evaluable because the corresponding effects were non-estimable.

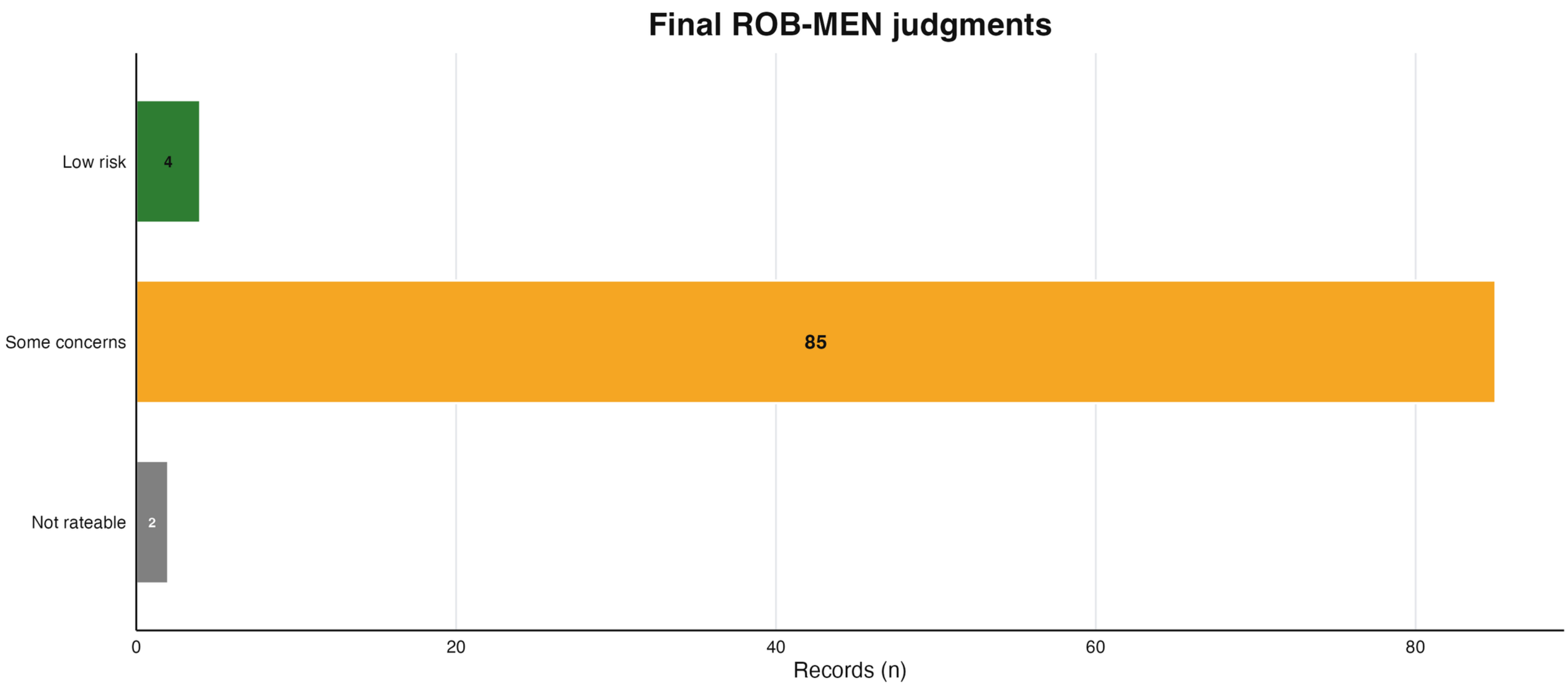

Ninety-one network estimates in the final version evidence appraisal.

Figure S19. Final ROB-MEN distribution across 91 network estimates: 85 some concerns, four low risk, and two not rateable because the corresponding effects were non-estimable.

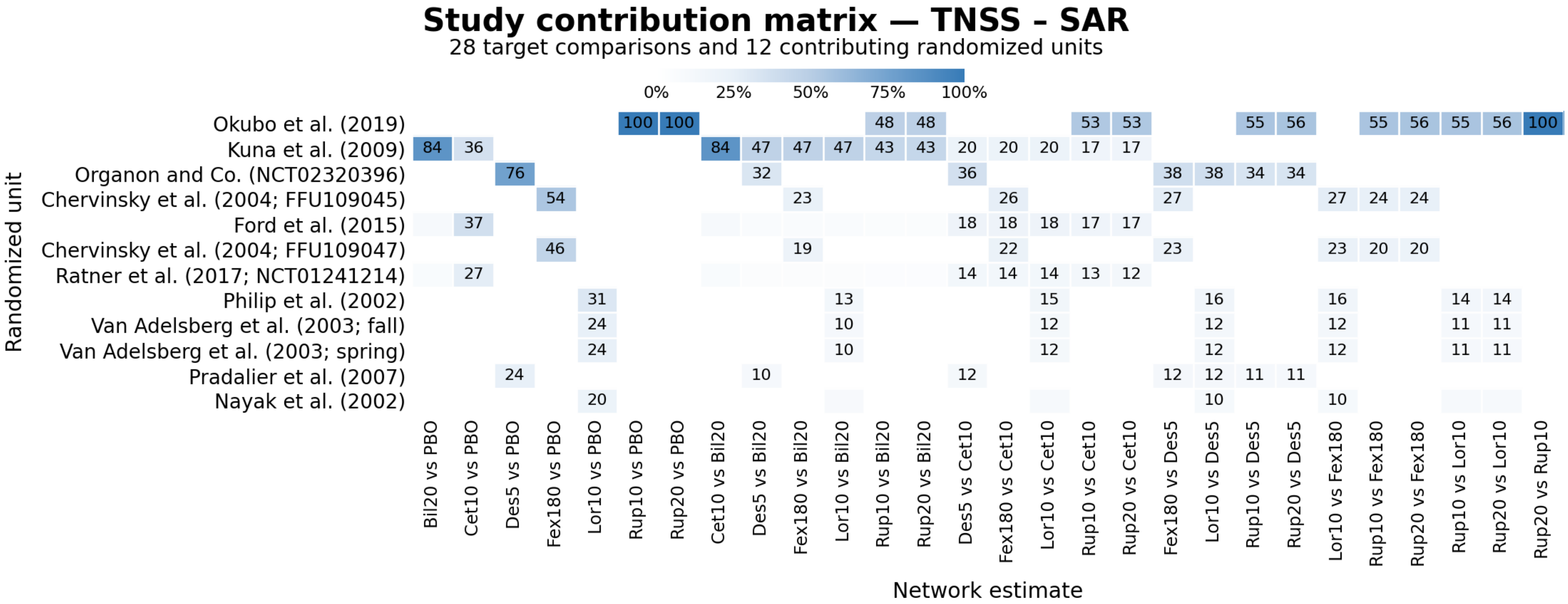

Figure S20. Independent-randomization-level contribution matrix for TNSS–SAR. Columns are target estimates and rows are independent randomizations. Cell values are percentage contributions after path-level aggregation; each target-comparison column sums to 100% within numerical tolerance. Contributions describe evidence flow, not certainty or risk of bias.

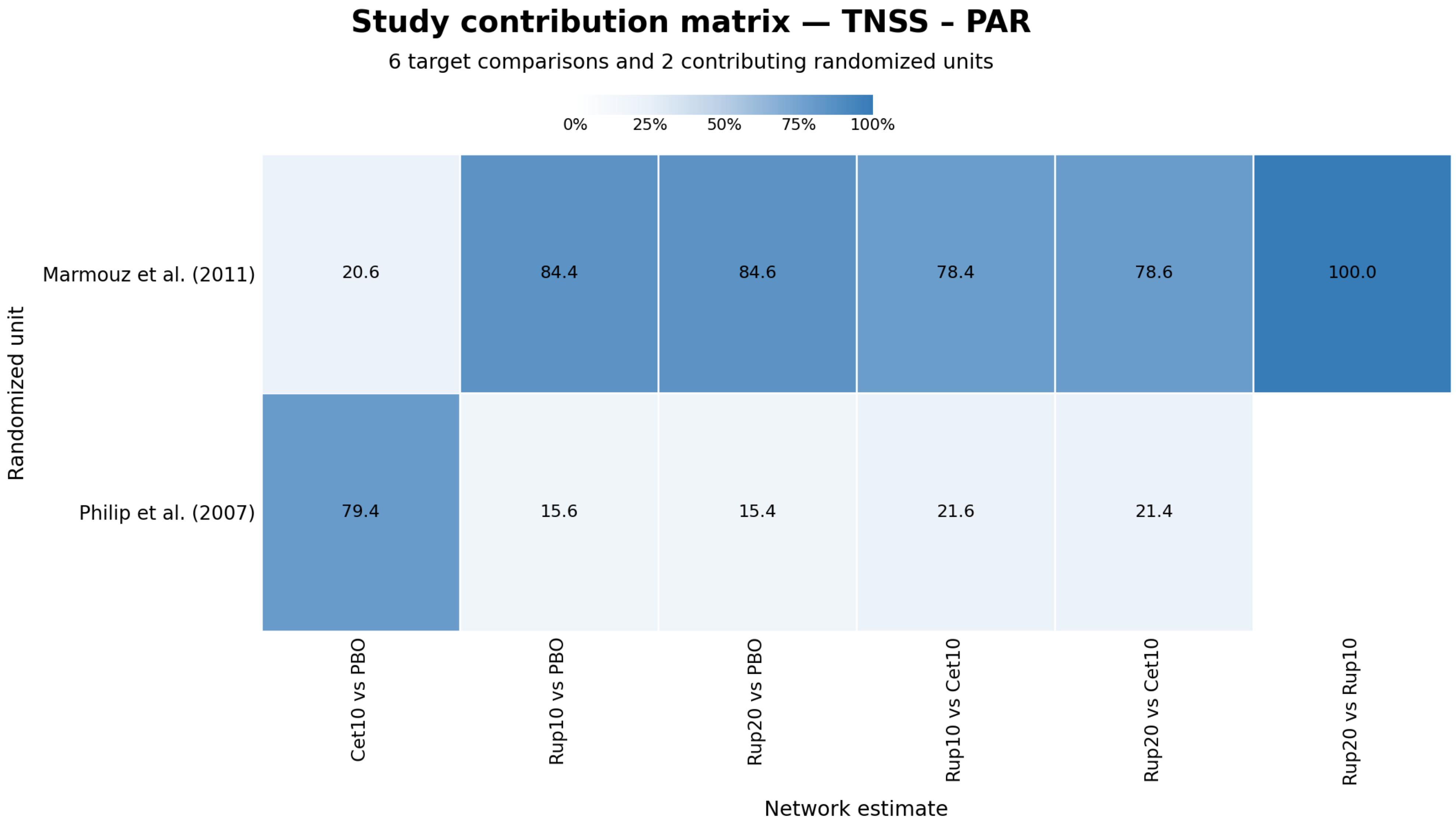

Figure S21. Independent-randomization-level contribution matrix for TNSS–PAR. Columns are target estimates and rows are independent randomizations. Cell values are percentage contributions after path-level aggregation; each target-comparison column sums to 100% within numerical tolerance. Contributions describe evidence flow, not certainty or risk of bias.

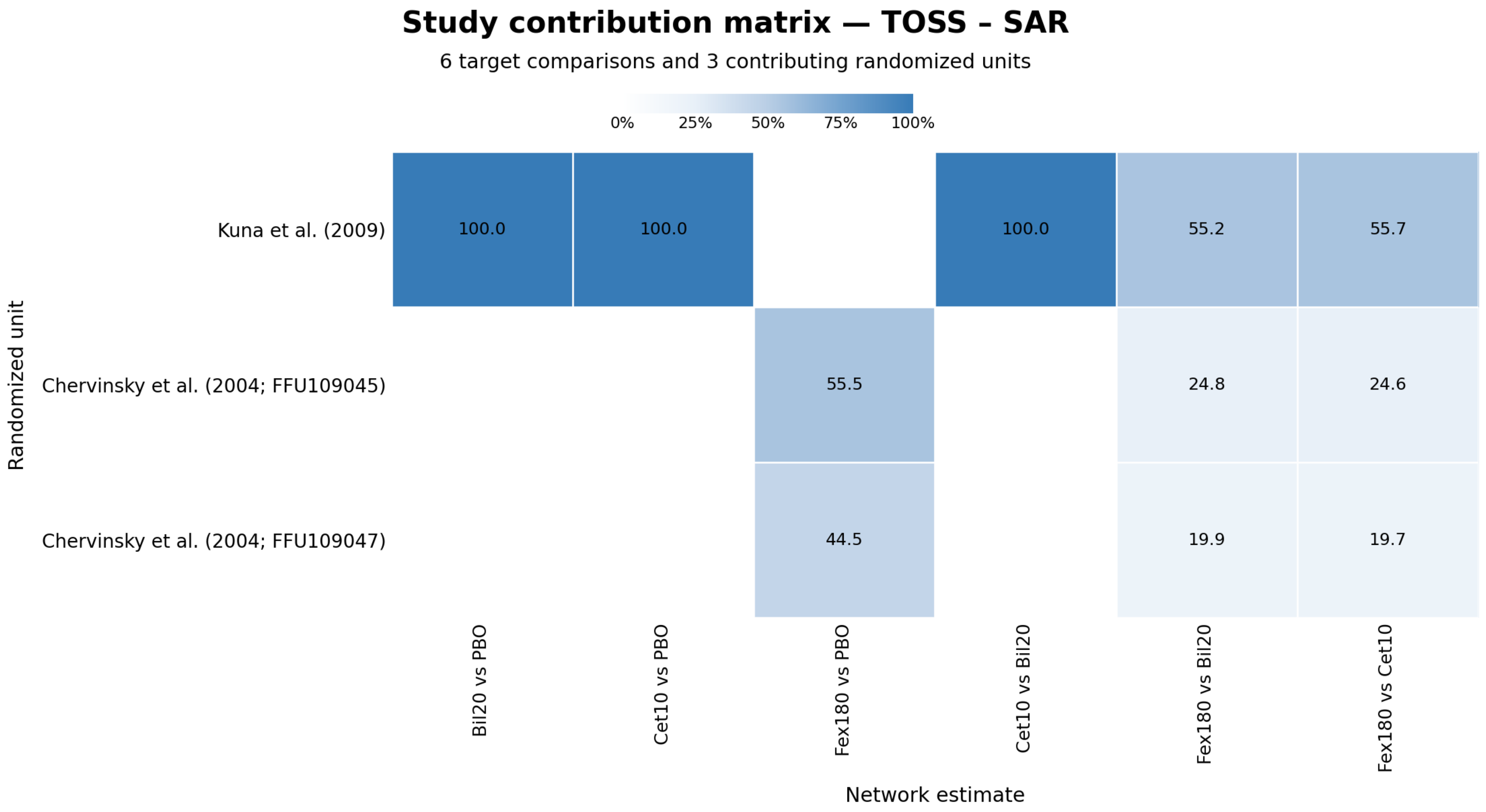

Figure S22. Independent-randomization-level contribution matrix for TOSS–SAR. Columns are target estimates and rows are independent randomizations. Cell values are percentage contributions after path-level aggregation; each target-comparison column sums to 100% within numerical tolerance. Contributions describe evidence flow, not certainty or risk of bias.

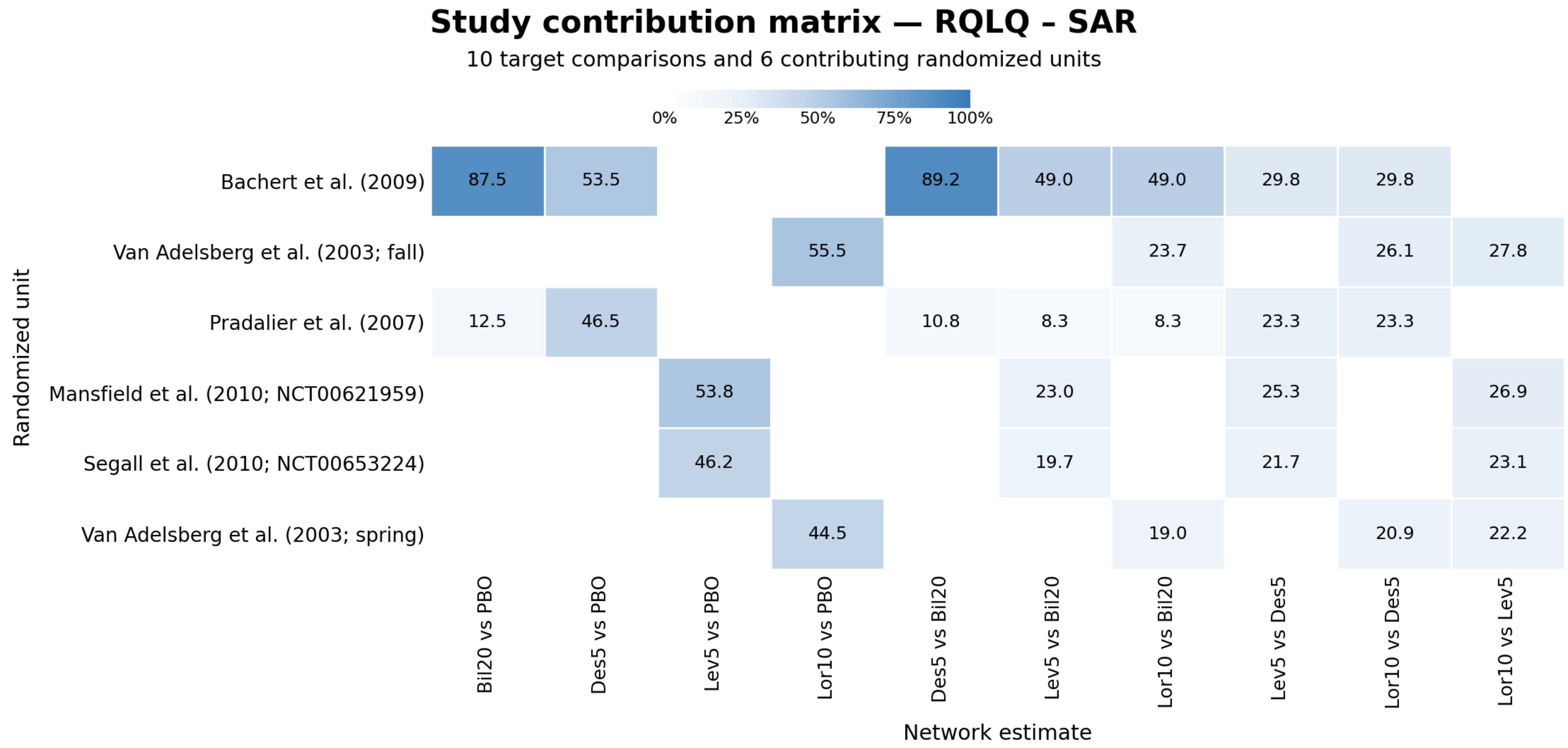

Figure S23. Independent-randomization-level contribution matrix for RQLQ–SAR. Columns are target estimates and rows are independent randomizations. Cell values are percentage contributions after path-level aggregation; each target-comparison column sums to 100% within numerical tolerance. Contributions describe evidence flow, not certainty or risk of bias.

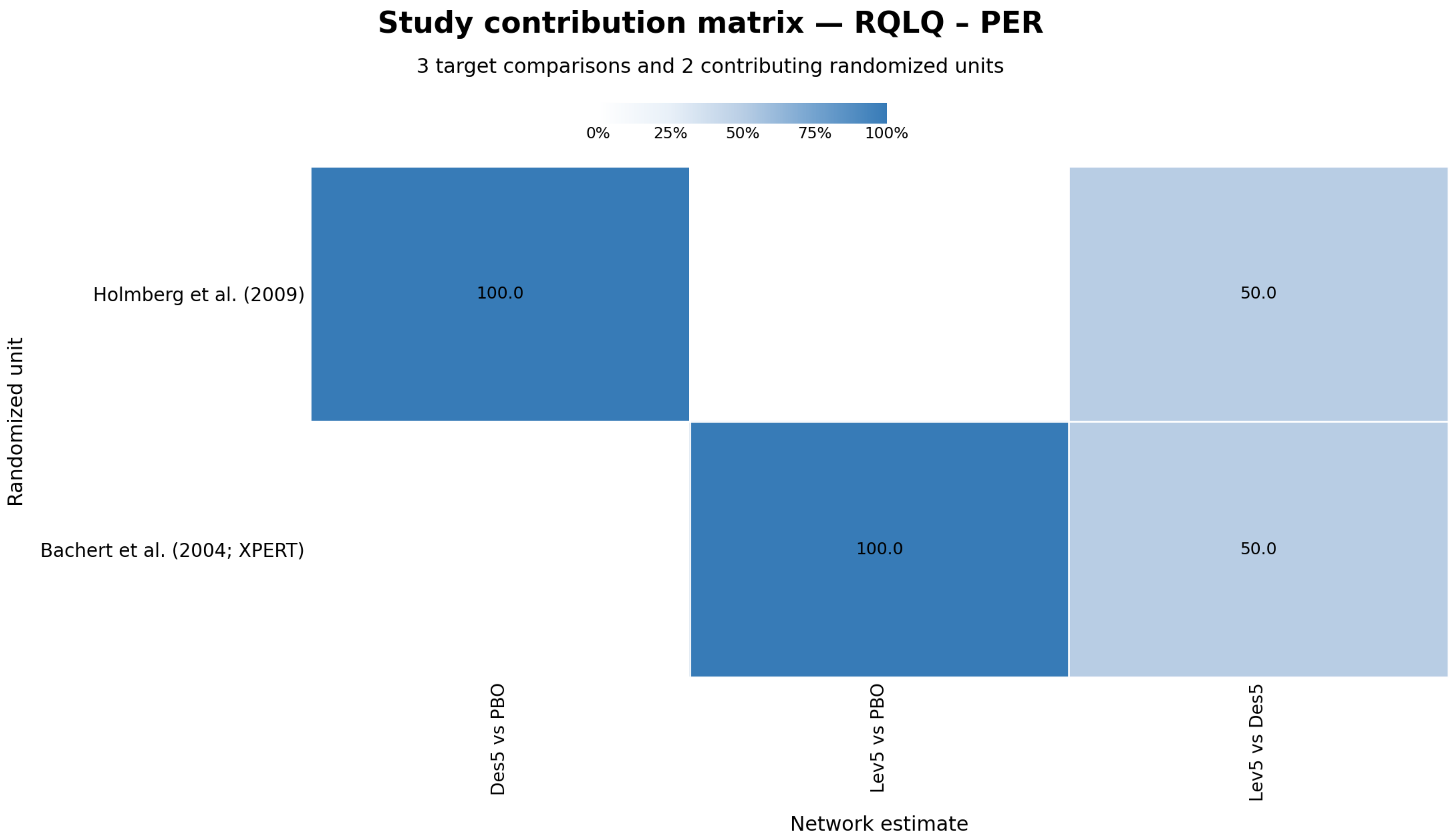

Figure S24. Independent-randomization-level contribution matrix for RQLQ–PER. Columns are target estimates and rows are independent randomizations. Cell values are percentage contributions after path-level aggregation; each target-comparison column sums to 100% within numerical tolerance. Contributions describe evidence flow, not certainty or risk of bias.

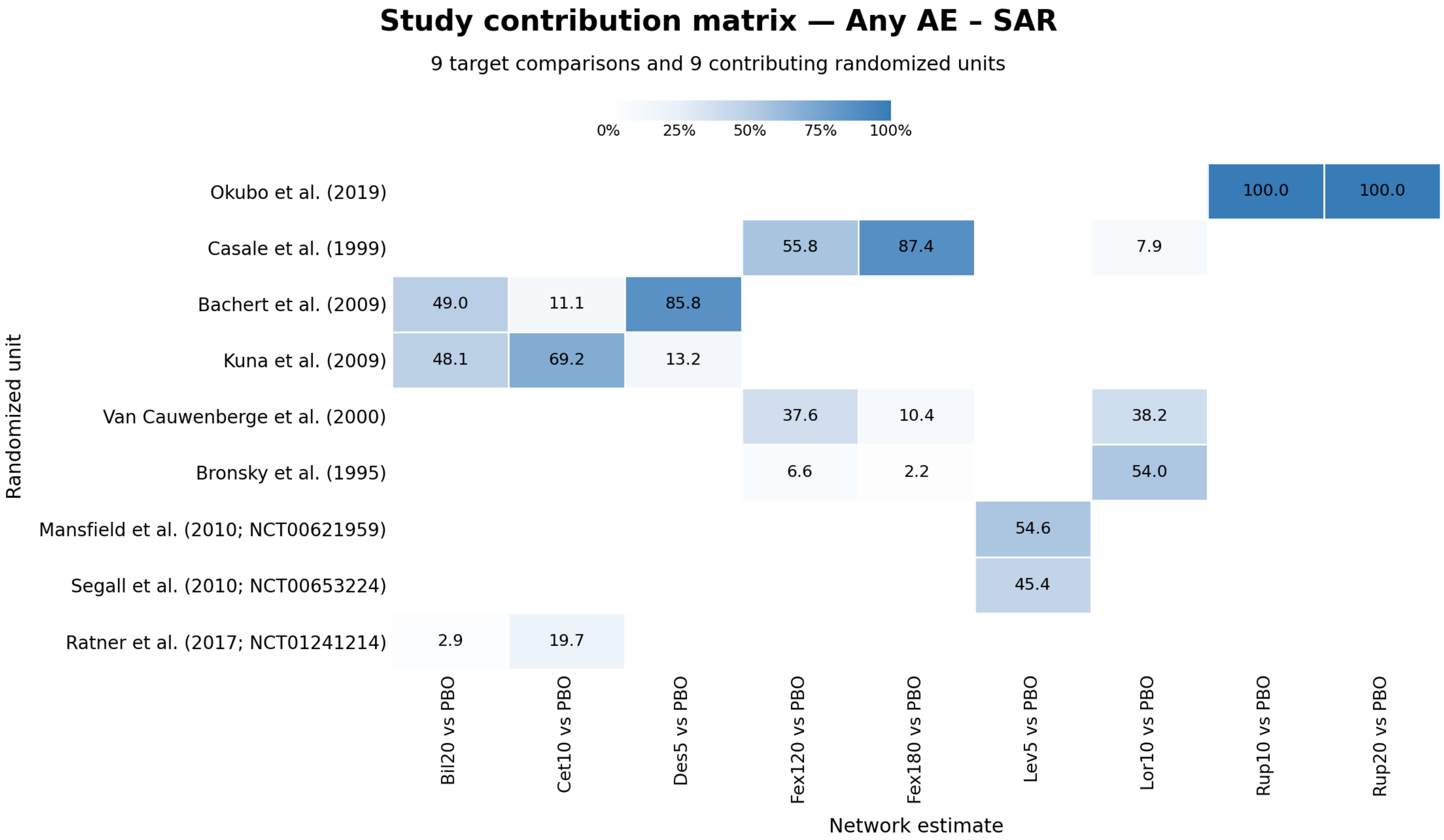

Figure S25. Independent-randomization-level contribution matrix for Any AE–SAR. Columns are target estimates and rows are independent randomizations. Cell values are percentage contributions after path-level aggregation; each target-comparison column sums to 100% within numerical tolerance. Contributions describe evidence flow, not certainty or risk of bias.

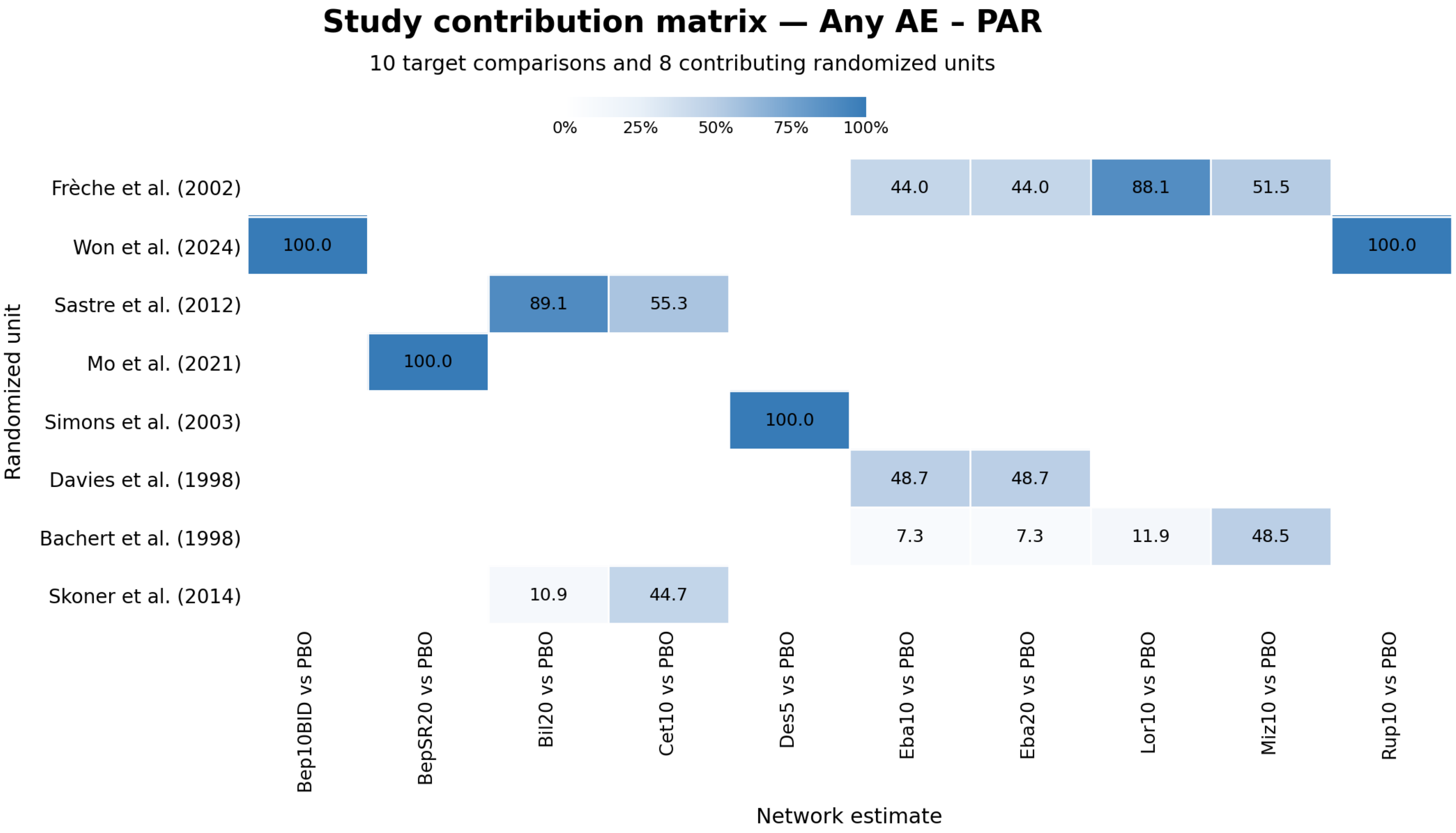

Figure S26. Independent-randomization-level contribution matrix for Any AE–PAR. Columns are target estimates and rows are independent randomizations. Cell values are percentage contributions after path-level aggregation; each target-comparison column sums to 100% within numerical tolerance. Contributions describe evidence flow, not certainty or risk of bias.

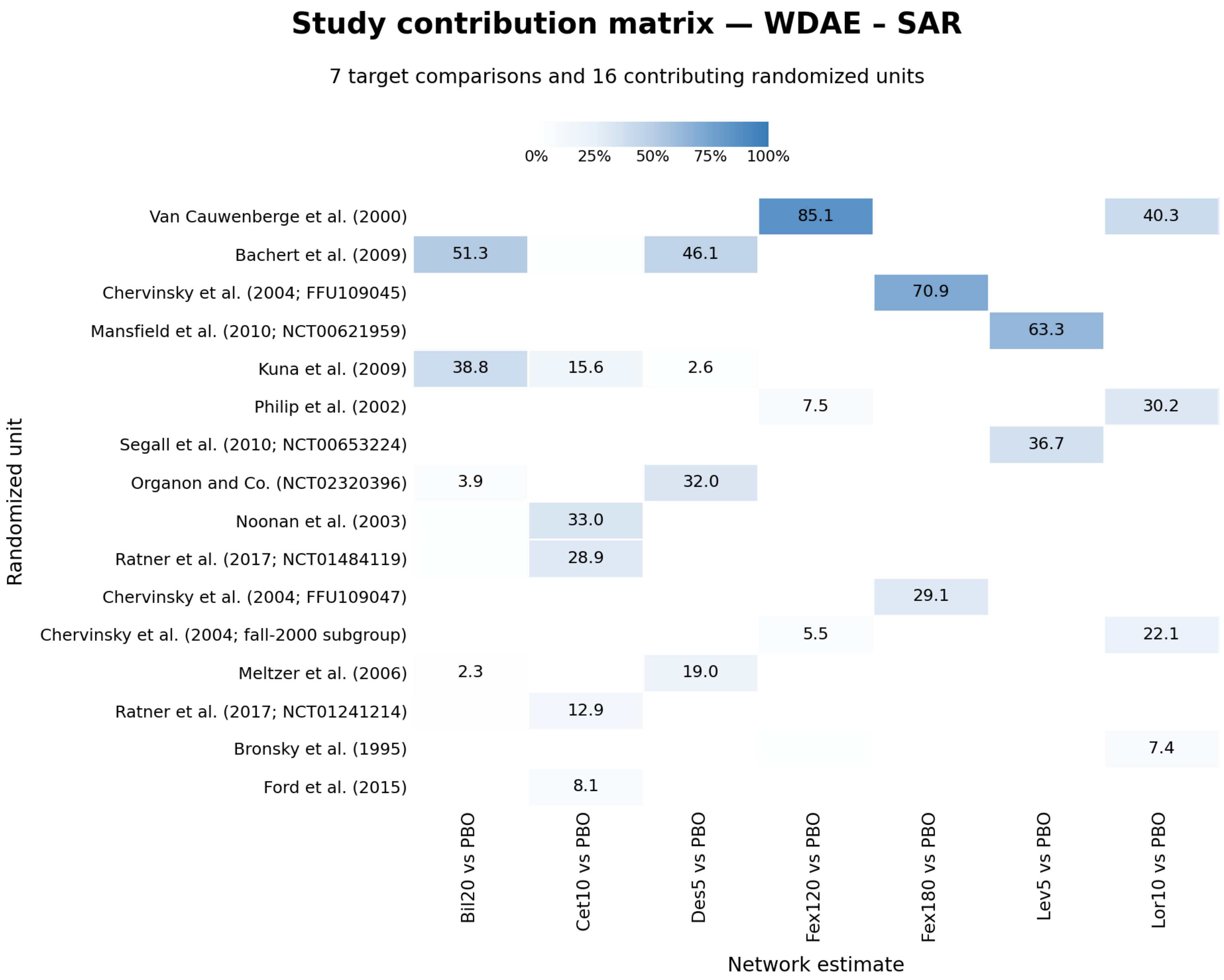

Figure S27. Independent-randomization-level contribution matrix for WDAE–SAR. Columns are target estimates and rows are independent randomizations. Cell values are percentage contributions after path-level aggregation; each target-comparison column sums to 100% within numerical tolerance. Contributions describe evidence flow, not certainty or risk of bias. Seven estimable target columns are shown; the two non-estimable rupatadine effects receive no contribution column.

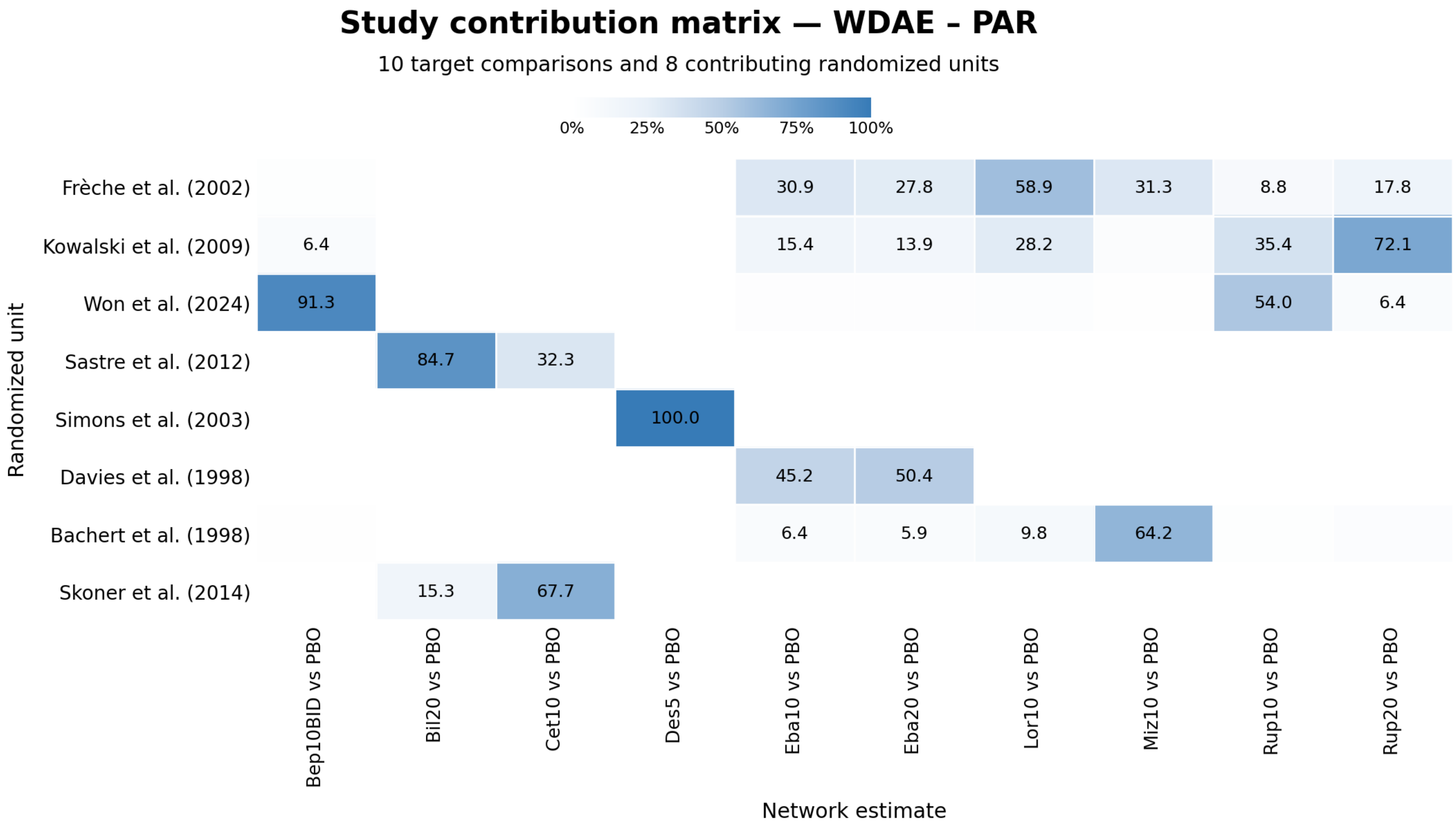

Figure S28. Independent-randomization-level contribution matrix for WDAE–PAR. Columns are target estimates and rows are independent randomizations. Cell values are percentage contributions after path-level aggregation; each target-comparison column sums to 100% within numerical tolerance. Contributions describe evidence flow, not certainty or risk of bias.

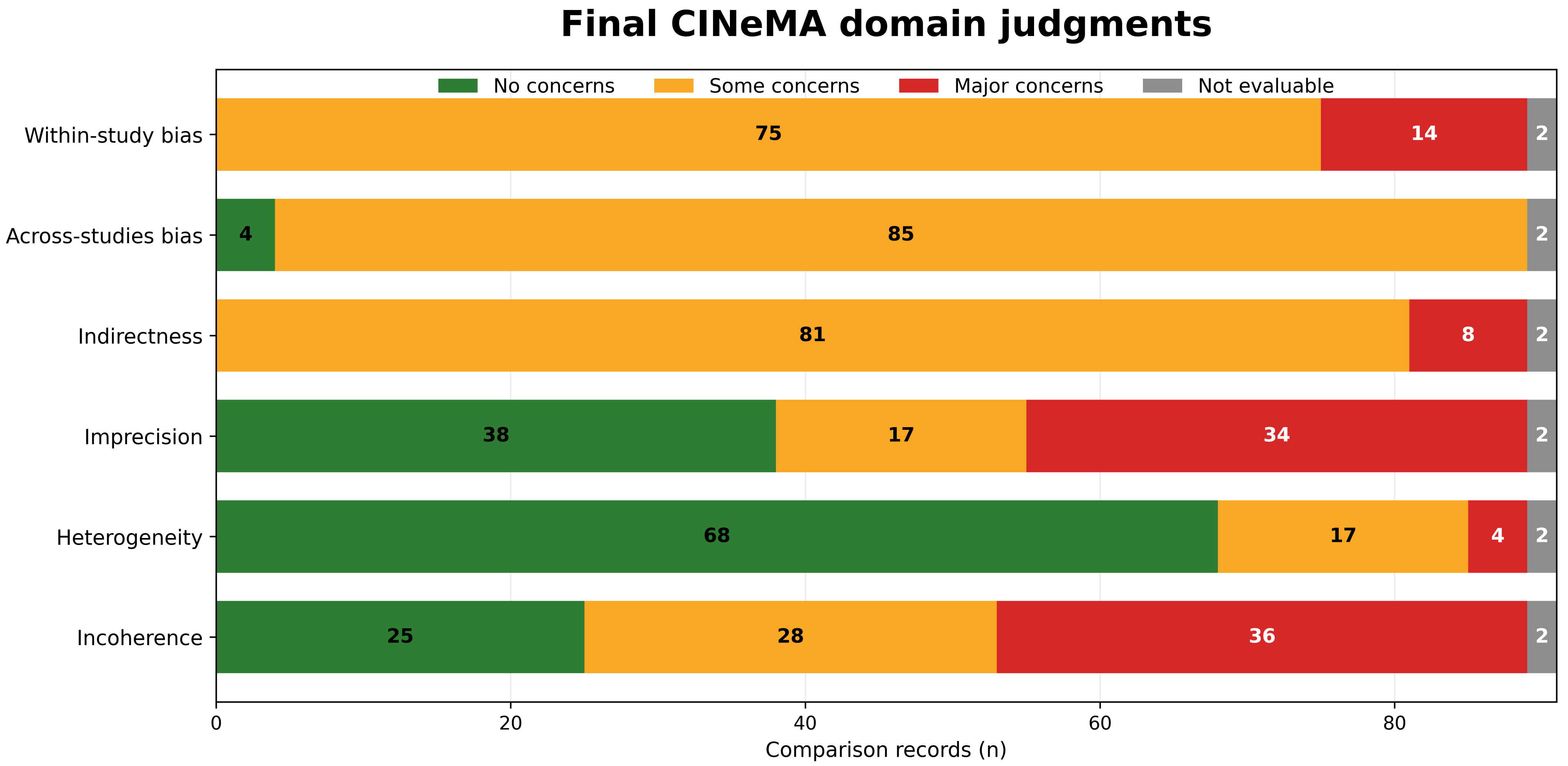

Figure S29. Current CINeMA domain-judgment distribution across 91 assessments. The incoherence domain is rated Major concerns for 36 estimable comparisons and Not evaluable only for the two non-estimable WDAE–SAR effects. Overall CINeMA confidence remains 89 very low and 2 not evaluable.

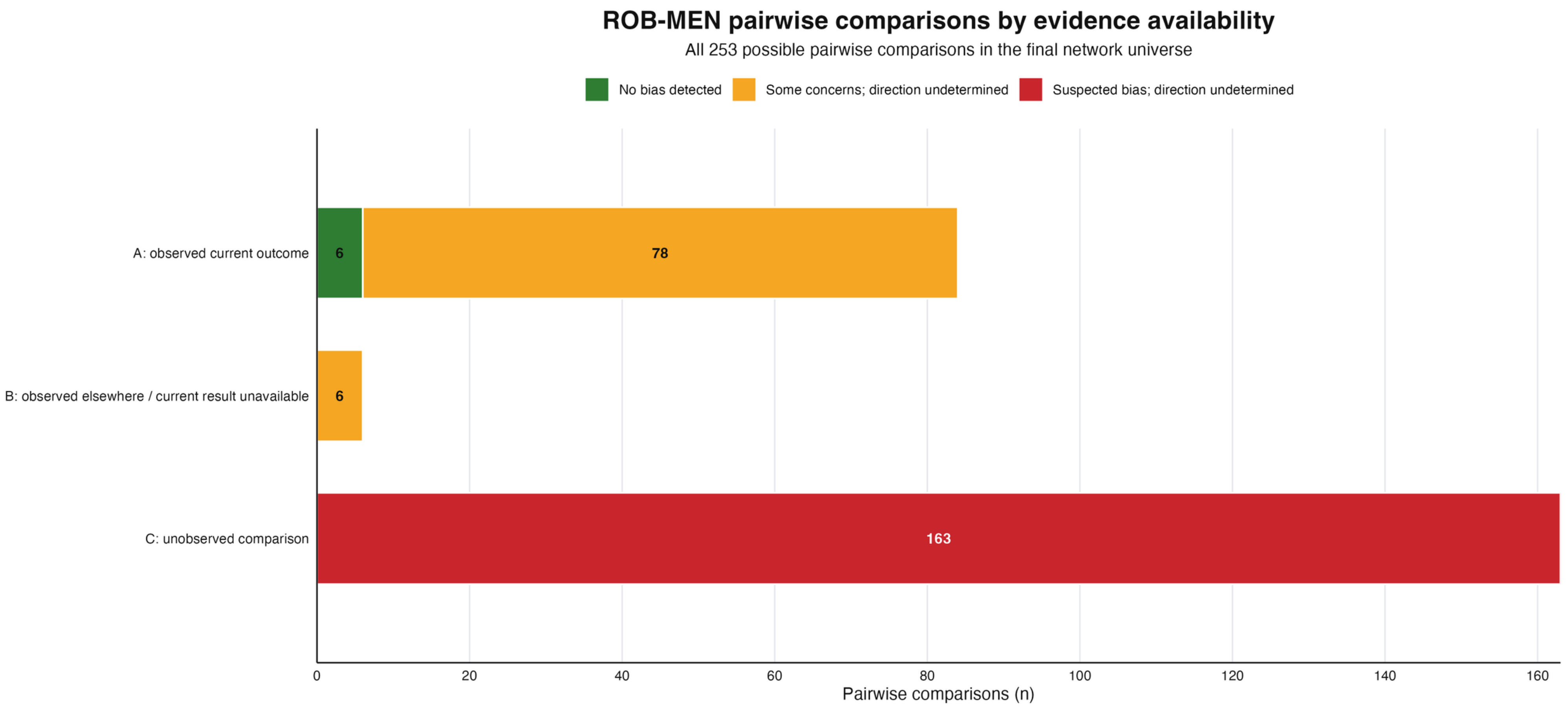

Evidence-availability groups and missing-evidence judgments are shown without inferring a direction where the underlying audit could not establish one.

Figure S30. ROB-MEN pairwise comparisons grouped by evidence availability. The figure describes the reconstructed pairwise evidence universe and is not a treatment ranking.

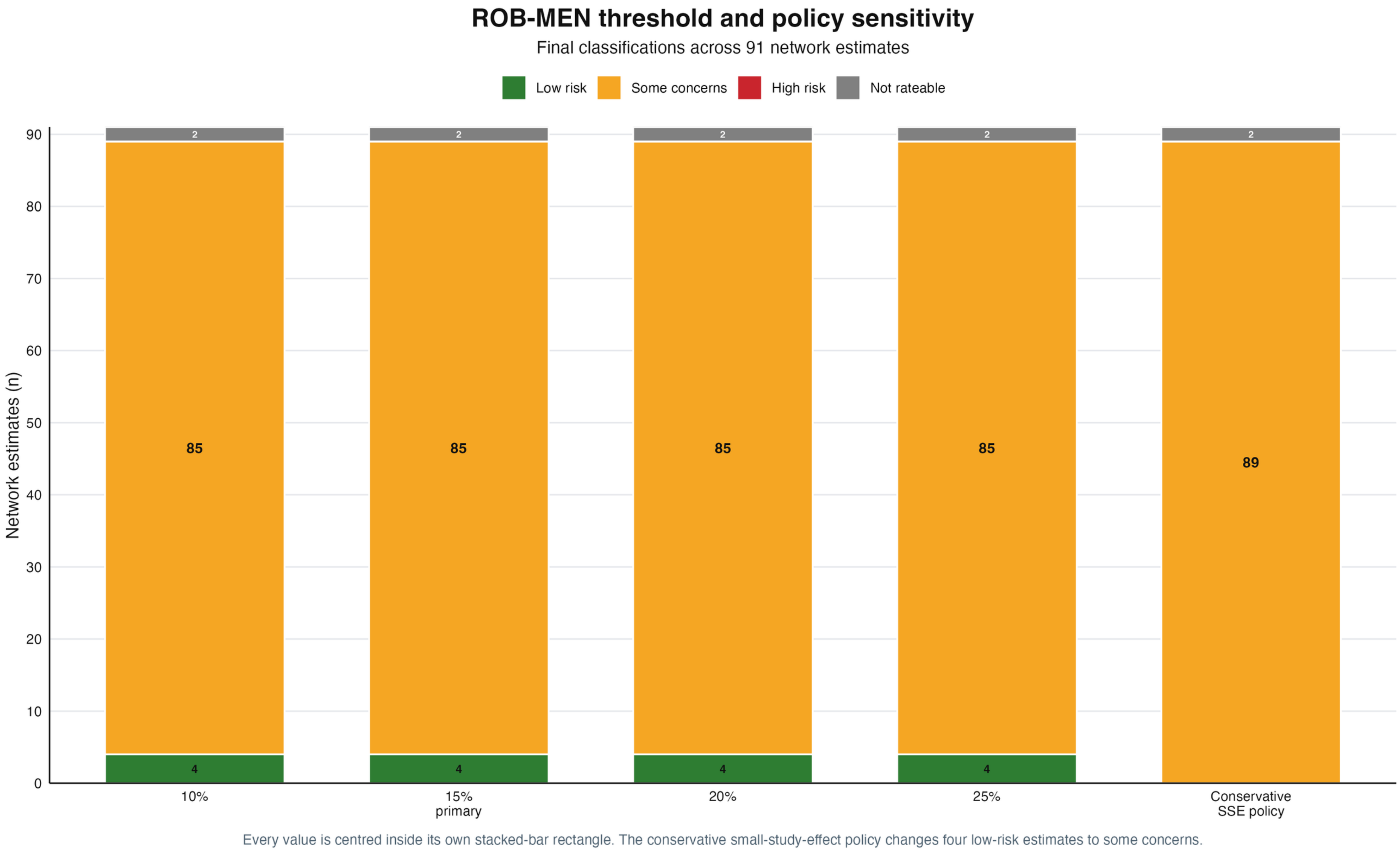

Figure S31. ROB-MEN sensitivity across project-specific contribution indicators and a separate conservative small-study-effect policy. The distribution was unchanged across the 10%, 15%, 20%, and 25% indicators (85 some concerns, four low risk, and two not rateable). Under the conservative policy, the four low-risk judgments shifted to some concerns, while the two not-rateable judgments remained unchanged. None of the contribution indicators is an official ROB-MEN threshold.

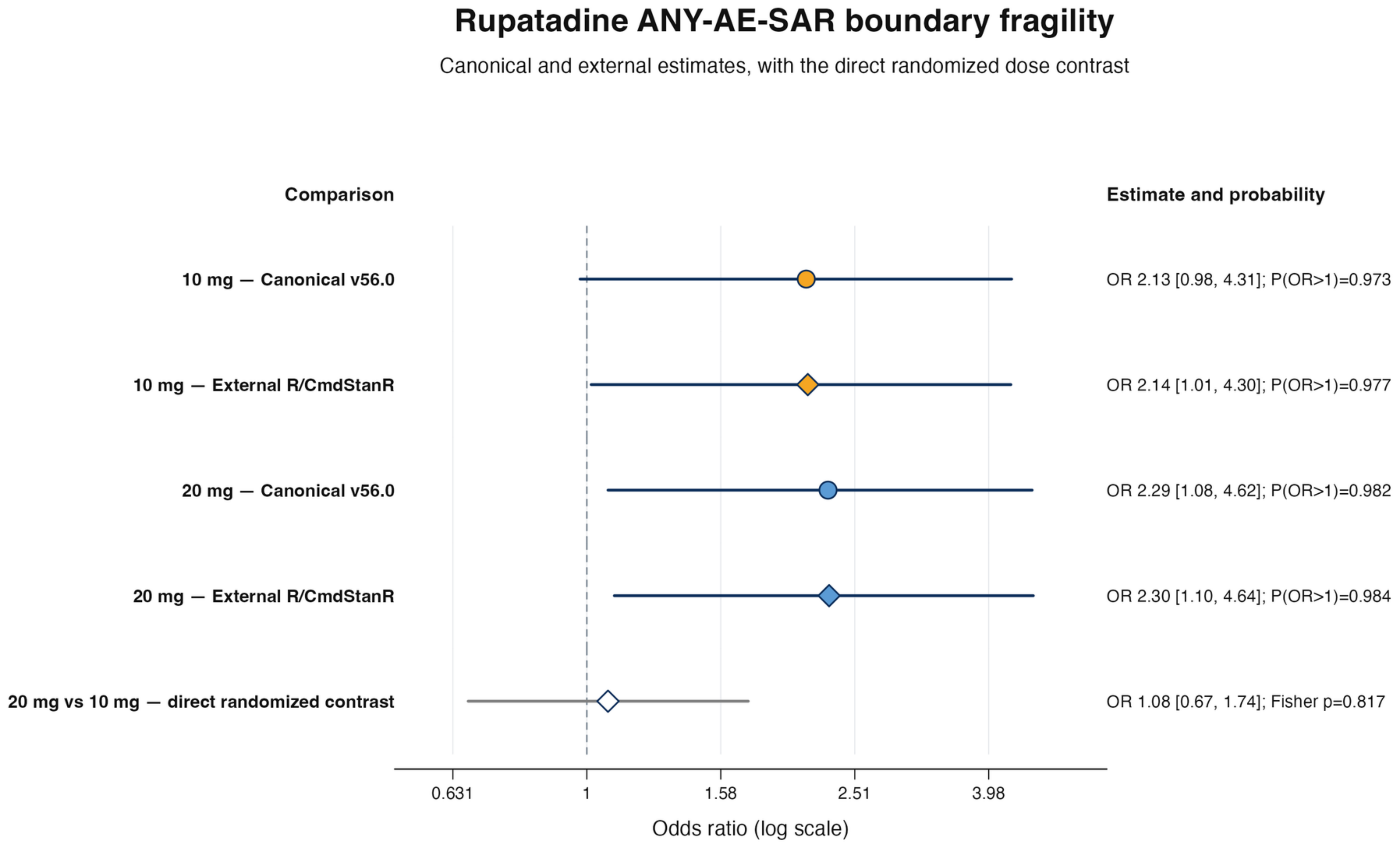

Amber points identify the boundary-indeterminate 10-mg comparison. Exact null-classification agreement remains 3/4; all four source-primary comparisons passed the quantitative magnitude tolerances. The direct 20-mg versus 10-mg contrast does not support a dose-response effect.

Figure S32. Rupatadine Any AE–SAR boundary audit comparing the primary and implementation-independent estimates and the direct randomized 20-mg versus 10-mg contrast. Posterior medians were stable; interval classifications depended on the heterogeneity prior and numerical proximity to the null boundary.
