# Supplementary material for: Oral Second-Generation H1-Antihistamine Regimens for Allergic Rhinitis: Systematic Review and Network Meta-Analysis of Effects and Estimability
Source: J Clin Med. 2026 Sep 15;15(18):7169. doi: 10.3390/jcm15187169 (PMC13607989; doi:10.3390/jcm15187169)
Supplement: Supplementary file 1 [file jcm-15-07169-s001.zip › PRISMA_2020_Abstract_Checklist_v1.1.pdf]

PRISMA 2020 for Abstracts Checklist

Oral Second-Generation H1-Antihistamine Regimens for Allergic Rhinitis: Systematic Review and Network Meta-Analysis of Effects and Estimability

Submission version 1.1 | 24 August 2026

Section and Topic	Item #	Checklist item	Reported (Yes/No)
**TITLE**			
Title	1	Identify the report as a systematic review.	Yes - Manuscript title, p. 1.
**BACKGROUND**			
Objectives	2	Provide an explicit statement of the main objective(s) or question(s) the review addresses.	Yes - Abstract, Background/Objectives, p. 1.
**METHODS**			
Eligibility criteria	3	Specify the inclusion and exclusion criteria for the review.	Partially reported - Abstract, Methods, p. 1: eligible randomized trials, rhinitis categories, natural allergen exposure and eligible oral second-generation H1-antihistamine regimens are specified, but the principal exclusion criteria are not explicitly summarized in the abstract.
Information sources	4	Specify the information sources (e.g. databases, registers) used to identify studies and the date when each was last searched.	Yes - Abstract, Methods, p. 1: all seven information sources are named with execution dates; review coverage through 30 June 2026 is stated.
Risk of bias	5	Specify the methods used to assess risk of bias in the included studies.	Yes - Abstract, Methods, p. 1: RoB 2, CINeMA and ROB-MEN; duplicate independent appraisal is stated.
Synthesis of results	6	Specify the methods used to present and synthesise results.	Yes - Abstract, Methods, p. 1: covariance-aware GLS for continuous outcomes and arm-level Bayesian binomial-logit models for binary harms.
**RESULTS**			
Included studies	7	Give the total number of included studies and participants and summarise relevant characteristics of studies.	Yes - Abstract, Results, p. 1: 61 reports, 66 report-randomization entries, 64 non-overlapping randomized cohorts, 34,831 randomized participants and 35 model-ready randomizations are reported.
Synthesis of results	8	Present results for main outcomes, preferably indicating the number of included studies and participants for each. If meta-analysis was done, report the summary estimate and confidence/credible interval. If comparing groups, indicate the direction of the effect (i.e. which group is favoured).	Partially reported — Abstract, Results, p. 1: principal directions of effect, one fully quantified binary estimate, active–active evidence, estimability and confidence are summarized; complete outcome-specific estimates and intervals are reported in the main text and Supplementary Appendix.
**DISCUSSION**			
Limitations of evidence	9	Provide a brief summary of the limitations of the evidence included in the review (e.g. study risk of bias, inconsistency and imprecision).	Yes - Abstract, Results/Conclusions, p. 1: sparse, placebo-anchored, almost uniformly very-low-confidence evidence and the somnolence evidence gap are stated.
Interpretation	10	Provide a general interpretation of the results and important implications.	Yes - Abstract, Conclusions, p. 1.
**OTHER**			
Funding	11	Specify the primary source of funding for the review.	Yes - Abstract, Conclusions, p. 1: no external funding supported review conduct.
Registration	12	Provide the register name and registration number.	Yes - Abstract, Conclusions, p. 1: retrospective OSF registration, doi:10.17605/OSF.IO/DPT5N.

Checklist wording from Page MJ, McKenzie JE, Bossuyt PM, et al. The PRISMA 2020 statement: an updated guideline for reporting systematic reviews. BMJ. 2021;372:n71. doi:10.1136/bmj.n71. CC BY 4.0.
