# Supplementary material for: Oral Second-Generation H1-Antihistamine Regimens for Allergic Rhinitis: Systematic Review and Network Meta-Analysis of Effects and Estimability
Source: J Clin Med. 2026 Sep 15;15(18):7169. doi: 10.3390/jcm15187169 (PMC13607989; doi:10.3390/jcm15187169)
Supplement: Supplementary file 1 [file jcm-15-07169-s001.zip › PRISMA_2020_Checklist_v1.1.pdf]

PRISMA 2020 Checklist

Oral Second-Generation H1-Antihistamine Regimens for Allergic Rhinitis: Systematic Review and Network Meta-Analysis of Effects and Estimability

Submission version 1.1 | 24 August 2026

Section and Topic	Item #	Checklist item	Location where item is reported
**TITLE**			
Title	1	Identify the report as a systematic review.	Manuscript title, p. 1.
**ABSTRACT**			
Abstract	2	See the PRISMA 2020 for Abstracts checklist.	Manuscript structured Abstract, p. 1; separate PRISMA 2020 for Abstracts checklist.
**INTRODUCTION**			
Rationale	3	Describe the rationale for the review in the context of existing knowledge.	Manuscript Introduction, p. 2.
Objectives	4	Provide an explicit statement of the objective(s) or question(s) the review addresses.	Manuscript Introduction, p. 2.
**METHODS**			
Eligibility criteria	5	Specify the inclusion and exclusion criteria for the review and how studies were grouped for the syntheses.	Manuscript Methods 2.3-2.6, pp. 4-7; Supplement pp. 14-22, Tables S3-S9.
Information sources	6	Specify all databases, registers, websites, organisations, reference lists and other sources searched or consulted to identify studies. Specify the date when each source was last searched or consulted.	Manuscript Methods 2.2, pp. 3-4; Supplement pp. 6-13, Table S2 (4 bibliographic databases and 3 trial registers, with platform/provider and last-search date).
Search strategy	7	Present the full search strategies for all databases, registers and websites, including any filters and limits used.	Supplement pp. 6-13, Table S2 (complete source-specific strategies as executed, including limits/restrictions).
Selection process	8	Specify the methods used to decide whether a study met the inclusion criteria of the review, including how many reviewers screened each record and each report retrieved, whether they worked independently, and if applicable, details of automation tools used in the process.	Manuscript Methods 2.4, pp. 4-5; Results 3.1, pp. 10-12; Figure 1. Rayyan.ai managed records; no automation tool made eligibility decisions.
Data collection process	9	Specify the methods used to collect data from reports, including how many reviewers collected data from each report, whether they worked independently, any processes for obtaining or confirming data from study investigators, and if applicable, details of automation tools used in the process.	Manuscript Methods 2.4, pp. 4–5; Supplement pp. 6 and 16, Table S5. Quantitative extraction was independently duplicated; no investigator contact was undertaken.
Data items	10a	List and define all outcomes for which data were sought. Specify whether all results that were compatible with each outcome domain in each study were sought (e.g. for all measures, time points, analyses), and if not, the methods used to decide which results to collect.	Manuscript Methods 2.5, pp. 5-6; Supplement pp. 17-18, Tables S6-S7.
Data items	10b	List and define all other variables for which data were sought (e.g. participant and intervention characteristics, funding sources). Describe any assumptions made about any missing or unclear information.	Manuscript Methods 2.3-2.6, pp. 4-7; Supplement pp. 15-17, 21-22 and 25-44, Tables S4-S6, S9, S12A-S13.
Study risk of bias assessment	11	Specify the methods used to assess risk of bias in the included studies, including details of the tool(s) used, how many reviewers assessed each study and whether they worked independently, and if applicable, details of automation tools used in the process.	Manuscript Methods 2.8, pp. 8-9; Supplement p. 57 and pp. 82-87, Tables S21/S21A and S30.
Effect measures	12	Specify for each outcome the effect measure(s) (e.g. risk ratio, mean difference) used in the synthesis or presentation of results.	Manuscript Methods 2.5 and 2.7, pp. 5-8 (MDs for continuous outcomes; ORs for binary outcomes; no cross-outcome hierarchy). Supplement p. 88, Table S31 documents secondary binary active-active contrasts.

Section and Topic	Item #	Checklist item	Location where item is reported
Synthesis methods	13a	Describe the processes used to decide which studies were eligible for each synthesis (e.g. tabulating the study intervention characteristics and comparing against the planned groups for each synthesis (item #5)).	Manuscript Methods 2.3-2.6, pp. 4-7; Supplement pp. 14-22, Tables S3-S9.
Synthesis methods	13b	Describe any methods required to prepare the data for presentation or synthesis, such as handling of missing summary statistics, or data conversions.	Manuscript Methods 2.4-2.7, pp. 4-8; Supplement pp. 16-17, Tables S5-S6.
Synthesis methods	13c	Describe any methods used to tabulate or visually display results of individual studies and syntheses.	Manuscript Methods 2.6-2.10, pp. 6-10; main Figures 1-5 and Table 1; Supplement p. 61, Table S24.
Synthesis methods	13d	Describe any methods used to synthesize results and provide a rationale for the choice(s). If meta-analysis was performed, describe the model(s), method(s) to identify the presence and extent of statistical heterogeneity, and software package(s) used.	Manuscript Methods 2.7 and 2.10, pp. 7-10; Supplement p. 24, Table S11; p. 88, Table S31; Supplementary Data S1 and derivation/verification scripts.
Synthesis methods	13e	Describe any methods used to explore possible causes of heterogeneity among study results (e.g. subgroup analysis, meta-regression).	Manuscript Methods 2.6–2.9, pp. 6–9; Supplement pp. 21–23, Tables S9–S10.
Synthesis methods	13f	Describe any sensitivity analyses conducted to assess robustness of the synthesized results.	Manuscript Methods 2.9, p. 9; Supplement p. 23 and pp. 54-56, Tables S10 and S18-S20.
Reporting bias assessment	14	Describe any methods used to assess risk of bias due to missing results in a synthesis (arising from reporting biases).	Manuscript Methods 2.8, pp. 8-9; Results 3.8, p. 21; Supplement p. 57 and pp. 65-71, Tables S21 and S27; ROB-MEN records and contribution matrices.
Certainty assessment	15	Describe any methods used to assess certainty (or confidence) in the body of evidence for an outcome.	Manuscript Methods 2.8, pp. 8-9; Results 3.8, p. 21; Supplement p. 57 and pp. 65-71, Tables S21 and S27.
**RESULTS**			
Study selection	16a	Describe the results of the search and selection process, from the number of records identified in the search to the number of studies included in the review, ideally using a flow diagram.	Manuscript Results 3.1, pp. 10-12; Figure 1; Supplement pp. 25-44, Tables S12-S13.
Study selection	16b	Cite studies that might appear to meet the inclusion criteria, but which were excluded, and explain why they were excluded.	Supplement pp. 30-40, Table S12B (179 report-level exclusion decisions: 176 uniquely excluded reports plus 3 duplicate bibliographic records, with identifiers and reasons).
Study characteristics	17	Cite each included study and present its characteristics.	References [45-105]; Supplement pp. 42-44 and 79-81, Tables S13 and S29.
Risk of bias in studies	18	Present assessments of risk of bias for each included study.	Manuscript Results 3.8, p. 21; Supplement p. 57 and pp. 82-87, Tables S21/S21A and S30.
Results of individual studies	19	For all outcomes, present, for each study: (a) summary statistics for each group (where appropriate) and (b) an effect estimate and its precision (e.g. confidence/credible interval), ideally using structured tables or plots.	Supplement pp. 72-78, Tables S28A-S28B, report all model-ready continuous contrasts and binary arm inputs. Supplement pp. 25-44 (S12-S13) document retained reports/cohorts that did not contribute model-ready outcome data; absence of such data is not presented as a study-level effect estimate.
Results of syntheses	20a	For each synthesis, briefly summarise the characteristics and risk of bias among contributing studies.	Manuscript Results 3.2 and 3.8, pp. 12-16 and p. 21; Table 1; Supplement Tables S8-S9, S13, S21/S21A and S30.
Results of syntheses	20b	Present results of all statistical syntheses conducted. If meta-analysis was done, present for each the summary estimate and its precision (e.g. confidence/credible interval) and measures of statistical heterogeneity. If comparing groups, describe the direction of the effect.	Manuscript Results 3.3-3.6, pp. 16-20; Supplement pp. 45-56 and 63-91, Tables S14-S20 and S26-S33; Supplementary Data S1.
Results of syntheses	20c	Present results of all investigations of possible causes of heterogeneity among study results.	Manuscript Results 3.2, 3.6 and 3.8, pp. 12-16 and 19-21; Supplement Tables S9, S27 and S33.
Results of syntheses	20d	Present results of all sensitivity analyses conducted to assess the robustness of the synthesized results.	Manuscript Results 3.7, pp. 20-21; Supplement p. 23 and pp. 54-56, Tables S10 and S18-S20.

Section and Topic	Item #	Checklist item	Location where item is reported
Reporting biases	21	Present assessments of risk of bias due to missing results (arising from reporting biases) for each synthesis assessed.	Manuscript Results 3.8, p. 21; Supplement p. 57 and pp. 65-71, Tables S21 and S27.
Certainty of evidence	22	Present assessments of certainty (or confidence) in the body of evidence for each outcome assessed.	Manuscript Results 3.8, p. 21; Supplement p. 57 and pp. 65-71, Tables S21 and S27.
**DISCUSSION**			
Discussion	23a	Provide a general interpretation of the results in the context of other evidence.	Manuscript Discussion 4.1-4.5, pp. 22-24.
Discussion	23b	Discuss any limitations of the evidence included in the review.	Manuscript Discussion 4.6, pp. 24-26.
Discussion	23c	Discuss any limitations of the review processes used.	Manuscript Discussion 4.6, pp. 24-26, including retrospective registration, screening-record limitations, quantitative-selection limitations, harmonization, sparse structures and replication scope.
Discussion	23d	Discuss implications of the results for practice, policy, and future research.	Manuscript Discussion 4.5 and 4.7, pp. 24-26; Conclusions, pp. 26-27.
**OTHER INFORMATION**			
Registration and protocol	24a	Provide registration information for the review, including register name and registration number, or state that the review was not registered.	Manuscript structured Abstract, p. 1, and Methods 2.1, p. 3 (retrospective OSF registration: doi:10.17605/OSF.IO/DPT5N).
Registration and protocol	24b	Indicate where the review protocol can be accessed, or state that a protocol was not prepared.	Manuscript Methods 2.1, p. 3; Data Availability Statement, p. 27; OSF doi:10.17605/OSF.IO/DPT5N.
Registration and protocol	24c	Describe and explain any amendments to information provided at registration or in the protocol.	Manuscript Methods 2.1, p. 3; retrospectively registered OSF protocol and methodological audit trail; Supplement pp. 5 and 92, Tables S1 and S34. The review was registered retrospectively, and documented methodological changes and the subsequent submission-version relationship are reported transparently; no prospective-registration claim is made.
Support	25	Describe sources of financial or non-financial support for the review, and the role of the funders or sponsors in the review.	Manuscript Funding statement, p. 27. Review conduct received no external funding; NEXUS PEOPLE, S.L. funded only the APC and had no role in the study.
Competing interests	26	Declare any competing interests of review authors.	Manuscript Conflicts of Interest, p. 28.
Availability of data, code and other materials	27	Report which of the following are publicly available and where they can be found: template data-collection forms; data extracted from included studies; data used for all analyses; analytic code; any other materials used in the review.	Manuscript: Data Availability Statement; Supplement: Tables S25 and S34; Supplementary Data S1. The retrospectively registered protocol and methodological audit trail are publicly available in OSF Registries (doi:10.17605/OSF.IO/DPT5N). Zenodo v1.1 (doi:10.5281/zenodo.22086455) is the current public reproducibility release corresponding to submission version 1.1; Zenodo v1.0.0 (doi:10.5281/zenodo.21780525) remains the archived release corresponding to submission version 1.0. The v1.1 repository package includes machine-readable analytic data and results, analytic and derivation code, retained posterior draws used for the secondary binary active–active derivations, all 147 secondary contrast estimates, the 36-effect placebo-referenced concordance audit, source-file manifests, and the independent validation script, row-level output, and completed validation record. Original database exports, the duplicate-cluster log, and reviewer-specific screening timestamps were not retained. Copyrighted source reports are not redistributed.

Checklist wording from Page MJ, McKenzie JE, Bossuyt PM, et al. The PRISMA 2020 statement: an updated guideline for reporting systematic reviews. BMJ. 2021;372:n71. doi:10.1136/bmj.n71. CC BY 4.0.
