# Supplementary material for: Oral Second-Generation H1-Antihistamine Regimens for Allergic Rhinitis: Systematic Review and Network Meta-Analysis of Effects and Estimability
Source: J Clin Med. 2026 Sep 15;15(18):7169. doi: 10.3390/jcm15187169 (PMC13607989; doi:10.3390/jcm15187169)
Supplement: Supplementary file 1 [file jcm-15-07169-s001.zip › PRISMA_NMA_Checklist_v1.1.pdf]

PRISMA-NMA Checklist

Oral Second-Generation H1-Antihistamine Regimens for Allergic Rhinitis: Systematic Review and Network Meta-Analysis of Effects and Estimability

Submission version 1.1 | 24 August 2026

Section/Topic	Item #	Checklist Item	Reported on Page #
**TITLE**			
Title	1	Identify the report as a systematic review incorporating a network meta-analysis (or related form of meta-analysis).	Manuscript title, p. 1.
**ABSTRACT**			
Structured summary	2	Provide a structured summary including, as applicable: Background: main objectives Methods: data sources; study eligibility criteria, participants, and interventions; study appraisal; and synthesis methods, such as network meta-analysis. Results: number of studies and participants identified; summary estimates with corresponding confidence/credible intervals; treatment rankings may also be discussed. Authors may choose to summarize pairwise comparisons against a chosen treatment included in their analyses for brevity. Discussion/Conclusions: limitations; conclusions and implications of findings. Other: primary source of funding; systematic review registration number with registry name.	Manuscript structured Abstract, p. 1. Eligibility is summarized, but principal exclusion criteria are not explicitly listed in the abstract; see PRISMA 2020 for Abstracts item 3.
**INTRODUCTION**			
Rationale	3	Describe the rationale for the review in the context of what is already known, including mention of why a network meta-analysis has been conducted.	Manuscript Introduction, p. 2.
Objectives	4	Provide an explicit statement of questions being addressed, with reference to participants, interventions, comparisons, outcomes, and study design (PICOS).	Manuscript Introduction, p. 2; Methods 2.3-2.5, pp. 4-6.
**METHODS**			
Protocol and registration	5	Indicate whether a review protocol exists and if and where it can be accessed (e.g., Web address); and, if available, provide registration information, including registration number.	Manuscript Methods 2.1, p. 3; Data Availability Statement, p. 27; retrospective OSF registration doi:10.17605/OSF.IO/DPT5N.
Eligibility criteria	6	Specify study characteristics (e.g., PICOS, length of follow-up) and report characteristics (e.g., years considered, language, publication status) used as criteria for eligibility, giving rationale. Clearly describe eligible treatments included in the treatment network, and note whether any have been clustered or merged into the same node (with justification).	Manuscript Methods 2.3-2.5, pp. 4-6; Supplement pp. 14-18, Tables S3-S7. Regimen-specific nodes are defined in Table S4 and are not merged solely by molecule.
Information sources	7	Describe all information sources (e.g., databases with dates of coverage, contact with study authors to identify additional studies) in the search and date last searched.	Manuscript Methods 2.2, pp. 3-4; Supplement pp. 6-13, Table S2.
Search	8	Present full electronic search strategy for at least one database, including any limits used, such that it could be repeated.	Supplement pp. 7-13, Table S2 (all seven electronic source strategies are reproduced as executed).
Study selection	9	State the process for selecting studies (i.e., screening, eligibility, included in systematic review, and, if applicable, included in the meta-analysis).	Manuscript Methods 2.3-2.4, pp. 4-5; Results 3.1, pp. 10-12; Figure 1.
Data collection process	10	Describe method of data extraction from reports (e.g., piloted forms, independently, in duplicate) and any processes for obtaining and confirming data from investigators.	Manuscript Methods 2.4, pp. 4–5; Supplement pp. 6 and 16, Table S5. Quantitative extraction was independently duplicated; no investigator contact was undertaken.
Data items	11	List and define all variables for which data were sought (e.g., PICOS, funding sources) and any assumptions and simplifications made.	Manuscript Methods 2.3-2.6, pp. 4-7; Supplement Tables S4-S6, S9, S12A and S13.

Section/Topic	Item #	Checklist Item	Reported on Page #
Geometry of the network	S1	Describe methods used to explore the geometry of the treatment network under study and potential biases related to it. This should include how the evidence base has been graphically summarized for presentation, and what characteristics were compiled and used to describe the evidence base to readers.	Manuscript Methods 2.6, pp. 6-7; Table 1; Figure 2; Supplement pp. 19-20, Tables S8A-S8B; Figures S8-S16.
Risk of bias within individual studies	12	Describe methods used for assessing risk of bias of individual studies (including specification of whether this was done at the study or outcome level), and how this information is to be used in any data synthesis.	Manuscript Methods 2.8, pp. 8-9; Supplement p. 57 and pp. 82-87, Tables S21/S21A and S30.
Summary measures	13	State the principal summary measures (e.g., risk ratio, difference in means). Also describe the use of additional summary measures assessed, such as treatment rankings and surface under the cumulative ranking curve (SUCRA) values, as well as modified approaches used to present summary findings from meta-analyses.	Manuscript Methods 2.5 and 2.7, pp. 5-8 (MD and OR; no universal ranking/hierarchy); Supplement p. 88, Table S31 documents secondary within-draw binary active-active contrasts.
Planned methods of analysis	14	Describe the methods of handling data and combining results of studies for each network meta-analysis. This should include, but not be limited to: Handling of multi-arm trials; Selection of variance structure; Selection of prior distributions in Bayesian analyses; and Assessment of model fit.	Manuscript Methods 2.7 and 2.10, pp. 7-10; Supplement p. 24, Table S11; p. 88, Table S31; Supplementary Data S1 and derivation/verification scripts.
Assessment of Inconsistency	S2	Describe the statistical methods used to evaluate the agreement of direct and indirect evidence in the treatment network(s) studied. Describe efforts taken to address its presence when found.	Manuscript Methods 2.7, pp. 7-8; Supplement pp. 21-22, Table S9.
Risk of bias across studies	15	Specify any assessment of risk of bias that may affect the cumulative evidence (e.g., publication bias, selective reporting within studies).	Manuscript Methods 2.8, pp. 8-9; ROB-MEN records and contribution matrices.
Additional analyses	16	Describe methods of additional analyses if done, indicating which were pre-specified. This may include, but not be limited to, the following: Sensitivity or subgroup analyses; Meta-regression analyses; Alternative formulations of the treatment network; and Use of alternative prior distributions for Bayesian analyses (if applicable).	Manuscript Methods 2.9, p. 9; Supplement p. 23 and pp. 54-56, Tables S10 and S18-S20.
**RESULTS**			
Study selection	17	Give numbers of studies screened, assessed for eligibility, and included in the review, with reasons for exclusions at each stage, ideally with a flow diagram.	Manuscript Results 3.1, pp. 10-12; Figure 1; Supplement pp. 25-44, Tables S12-S13.
Presentation of network structure	S3	Provide a network graph of the included studies to enable visualization of the geometry of the treatment network.	Manuscript Figure 2, p. 15; Supplement Figures S8-S16.
Summary of network geometry	S4	Provide a brief overview of characteristics of the treatment network. This may include commentary on the abundance of trials and randomized patients for the different interventions and pairwise comparisons in the network, gaps of evidence in the treatment network, and potential biases reflected by the network structure.	Manuscript Results 3.2, pp. 12-16; Table 1; Supplement pp. 19-20, Tables S8A-S8B.
Study characteristics	18	For each study, present characteristics for which data were extracted (e.g., study size, PICOS, follow-up period) and provide the citations.	References [45-105]; Supplement pp. 42-44 and 79-81, Tables S13 and S29.
Risk of bias within studies	19	Present data on risk of bias of each study and, if available, any outcome level assessment.	Manuscript Results 3.8, p. 21; Supplement p. 57 and pp. 82-87, Tables S21/S21A and S30.
Results of individual studies	20	For all outcomes considered (benefits or harms), present, for each study: 1) simple summary data for each intervention group, and 2) effect estimates and confidence intervals. Modified approaches may be needed to deal with information from larger networks.	Supplement pp. 72-78, Tables S28A-S28B, report all model-ready continuous contrasts and binary arm inputs. Supplement pp. 25-44 (S12-S13) document retained studies/cohorts without model-ready target data; no study-level effect estimate is implied for those cohorts.

Section/Topic	Item #	Checklist Item	Reported on Page #
Synthesis of results	21	Present results of each meta-analysis done, including confidence/credible intervals. In larger networks, authors may focus on comparisons versus a particular comparator (e.g. placebo or standard care), with full findings presented in an appendix. League tables and forest plots may be considered to summarize pairwise comparisons. If additional summary measures were explored (such as treatment rankings), these should also be presented.	Manuscript Results 3.3-3.6, pp. 16-20; Supplement Tables S14-S20 and S26-S33; Supplementary Data S1.
Exploration for inconsistency	S5	Describe results from investigations of inconsistency. This may include such information as measures of model fit to compare consistency and inconsistency models, P values from statistical tests, or summary of inconsistency estimates from different parts of the treatment network.	Manuscript Results 3.6, pp. 19-20; Supplement pp. 21-22 and 65-71, Tables S9 and S27; Incoherence_Network_Status_v1.1.csv. Three global tests were not re-estimated after the final source-verified freeze and this is explicitly reported as a limitation; absence/non-estimability of a test is not treated as evidence of consistency.
Risk of bias across studies	22	Present results of any assessment of risk of bias across studies for the evidence base being studied.	Manuscript Results 3.8, p. 21; Supplement Tables S21 and S27.
Results of additional analyses	23	Give results of additional analyses, if done (e.g., sensitivity or subgroup analyses, meta-regression analyses, alternative network geometries studied, alternative choice of prior distributions for Bayesian analyses, and so forth).	Manuscript Results 3.7, pp. 20-21; Supplement Tables S10 and S18-S20.
**DISCUSSION**			
Summary of evidence	24	Summarize the main findings, including the strength of evidence for each main outcome; consider their relevance to key groups (e.g., healthcare providers, users, and policy-makers).	Manuscript Discussion 4.1-4.2, pp. 22-23.
Limitations	25	Discuss limitations at study and outcome level (e.g., risk of bias), and at review level (e.g., incomplete retrieval of identified research, reporting bias). Comment on the validity of the assumptions, such as transitivity and consistency. Comment on any concerns regarding network geometry (e.g., avoidance of certain comparisons).	Manuscript Discussion 4.3-4.6, pp. 23-26, including transitivity, incoherence, sparse geometry, reporting limitations and the unassessed confidence of secondary binary active-active contrasts.
Conclusions	26	Provide a general interpretation of the results in the context of other evidence, and implications for future research.	Manuscript Discussion 4.4-4.7, pp. 23-26; Conclusions, pp. 26-27.
**FUNDING**			
Funding	27	Describe sources of funding for the systematic review and other support (e.g., supply of data); role of funders for the systematic review. This should also include information regarding whether funding has been received from manufacturers of treatments in the network and/or whether some of the authors are content experts with professional conflicts of interest that could affect use of treatments in the network.	Manuscript Funding statement, p. 27, and Conflicts of Interest, p. 28. Review conduct received no external funding; the APC funder had no study role; relevant author relationships are disclosed.

Checklist wording from Hutton B, Salanti G, Caldwell DM, et al. The PRISMA Extension Statement for Reporting of Systematic Reviews Incorporating Network Meta-analyses of Health Care Interventions. Ann Intern Med. 2015;162:777-784. doi:10.7326/M14-2385.
