# Supplementary material for: Oral Second-Generation H1-Antihistamine Regimens for Allergic Rhinitis: Systematic Review and Network Meta-Analysis of Effects and Estimability
Source: J Clin Med. 2026 Sep 15;15(18):7169. doi: 10.3390/jcm15187169 (PMC13607989; doi:10.3390/jcm15187169)
Supplement: Supplementary file 1 [file jcm-15-07169-s001.zip › PRISMA_S_Checklist_v1.1.pdf]

PRISMA-S Checklist

Oral Second-Generation H1-Antihistamine Regimens for Allergic Rhinitis: Systematic Review and Network Meta-Analysis of Effects and Estimability

Submission version 1.1 | 24 August 2026

Section and Topic	Item #	Checklist item	Location where item is reported
**INFORMATION SOURCES AND METHODS**			
Database name	1	Name each individual database searched, stating the platform for each.	Supplement p. 6, Table S2 (4 bibliographic databases and 3 trial registers; platform/interface and provider stated for each).
Multi-database searching	2	If databases were searched simultaneously on a single platform, state the name of the platform, listing all of the databases searched.	Not applicable - sources were searched separately; Supplement p. 6.
Study registries	3	List any study registries searched.	Manuscript Methods 2.2, p. 3; Supplement p. 6, Table S2 (WHO ICTRP, ClinicalTrials.gov and EU Clinical Trials Register).
Online resources and browsing	4	Describe any online or print source purposefully searched or browsed (e.g., tables of contents, print conference proceedings, web sites), and how this was done.	Not applicable — No additional online or print resource was purposefully searched or browsed as a separate information-source route. Reference-list checking was limited to report linkage and trial-family completeness and is reported under Item 5. Supplement p. 6.
Citation searching	5	Indicate whether cited references or citing references were examined, and describe any methods used for locating cited/citing references (e.g., browsing reference lists, using a citation index, setting up email alerts for references citing included studies).	Supplement p. 6: cited reference lists were examined for linkage/completeness; no forward-citation search or citation alert was undertaken.
Contacts	6	Indicate whether additional studies or data were sought by contacting authors, experts, manufacturers, or others.	Supplement p. 6: no study-investigator, expert or manufacturer contact and no separate search for unpublished data beyond the listed trial registers.
Other methods	7	Describe any additional information sources or search methods used.	Supplement p. 6; Figure 1: no separate other-methods route was used (n = 0).
**SEARCH STRATEGIES**			
Full search strategies	8	Include the search strategies for each database and information source, copied and pasted exactly as run.	Supplement pp. 7-13, Table S2: all seven source-specific strategies are reproduced as executed; only typographical line wrapping/formatting was altered.
Limits and restrictions	9	Specify that no limits were used, or describe any limits or restrictions applied to a search (e.g., date or time period, language, study design) and provide justification for their use.	Supplement pp. 6-13: no language restriction; sources searched from inception to the prespecified review coverage cutoff of 30 June 2026; source-specific syntax/limits are reproduced.
Search filters	10	Indicate whether published search filters were used (as originally designed or modified), and if so, cite the filter(s) used.	Supplement p. 6: no published or externally validated methodological search filter was used.
Prior work	11	Indicate when search strategies from other literature reviews were adapted or reused for a substantive part or all of the search, citing the previous review(s).	Supplement p. 6: strategies were developed specifically for this review and were not adapted from a published review strategy.
Updates	12	Report the methods used to update the search(es) (e.g., rerunning searches, email alerts).	Not applicable - no subsequent search update, rerun or alert was conducted; Supplement p. 6.
Dates of searches	13	For each search strategy, provide the date when the last search occurred.	Manuscript Methods 2.2, p. 3; Supplement pp. 6-13: PubMed/Embase 1 July 2026; Web of Science/CENTRAL 2 July; WHO ICTRP/ClinicalTrials.gov/EU CTR 3 July 2026.
**PEER REVIEW**			
Peer review	14	Describe any search peer review process.	Supplement p. 6: PRESS peer review was not performed.
**MANAGING RECORDS**			
Total records	15	Document the total number of records identified from each database and other information sources.	Supplement p. 6, Table S2; Manuscript Results 3.1, p. 10: source-specific counts total 12,183 records before deduplication.
Deduplication	16	Describe the processes and any software used to deduplicate records from multiple database searches and other information sources.	Manuscript Methods 2.2, pp. 3-4; Supplement p. 6: Rayyan.ai; identical DOI or corroborated title/author/content matching; 6,261 duplicates removed before screening. Final report-level reconciliation later identified three duplicate bibliographic representations among the 240 records assessed for eligibility; the duplicate-cluster log was not retained.

Checklist wording from Rethlefsen ML, Kirtley S, Waffenschmidt S, et al. PRISMA-S: an extension to the PRISMA Statement for Reporting Literature Searches in Systematic Reviews. Syst Rev. 2021;10:39. doi:10.1186/s13643-020-01542-z.
